# Dynamic and Persistent Cyclochirality in Hydrogen-Bonded Derivatives of Medium-Ring Triamines

**DOI:** 10.1021/jacs.3c06570

**Published:** 2023-08-18

**Authors:** David T. J. Morris, Steven M. Wales, Javier Echavarren, Matej Žabka, Giulia Marsico, John W. Ward, Natalie E. Pridmore, Jonathan Clayden

**Affiliations:** School of Chemistry, University of Bristol, Cantock’s Close, Bristol BS8 1TS, U.K.

## Abstract

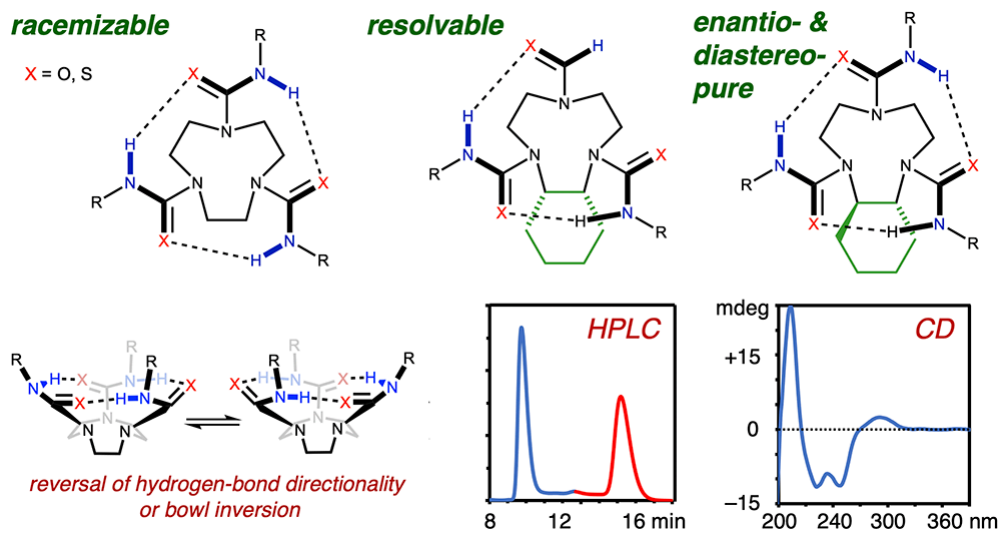

Cyclic triureas derived from 1,4,7-triazacyclononane (TACN) were synthesized; X-ray crystallography showed a chiral bowl-like conformation with each urea hydrogen-bonded to its neighbor with uniform directionality, forming a “cyclochiral” closed loop of hydrogen bonds. Variable-temperature ^1^H NMR, ^1^H-^1^H exchange spectroscopy, Eyring analysis, computational modeling, and studies in various solvents revealed that cyclochirality is dynamic (Δ*G*^‡^_25°C_ = 63–71 kJ mol^–1^ in noncoordinating solvents), exchanging between enantiomers by two mechanisms: bowl inversion and directionality reversal, with the former subject to a slightly smaller enantiomerization barrier. The enantiomerization rate substantially increased in the presence of hydrogen-bonding solvents. Population of only one of the two cyclochiral hydrogen-bond directionalities could be induced by annulating one ethylene bridge with a *trans*-cyclohexane. Alternatively, enantiomerization could be inhibited by annulating one ethylene bridge with a *cis*-cyclohexane (preventing bowl inversion) and replacing one urea function with a formamide (preventing directionality reversal). Combining these structural modifications resulted in an enantiomerization barrier of Δ*G*^‡^_25°C_ = 93 kJ mol^–1^, furnishing a planar-chiral, atropisomeric bowl-shaped structure whose stereochemical stability arises solely from its hydrogen-bonding network.

## Introduction

Hydrogen-bonding networks occur in both natural^1,2^ and artificial oligomeric molecules such as peptides and foldamers^3−5^ and are key to the adoption of stable secondary structural features such as helices, turns, and sheets.^6,7^ Less commonly, hydrogen-bonding networks may form closed loops of multiple hydrogen-bonding units, which cooperatively stabilize the structure. For example, guanine-rich DNA can aggregate, allowing four guanine units to hydrogen-bond to each other in a coplanar fashion, forming the stable cyclic hydrogen-bonded networks that stack to form G-quadruplexes.^8^

**Figure 1:**
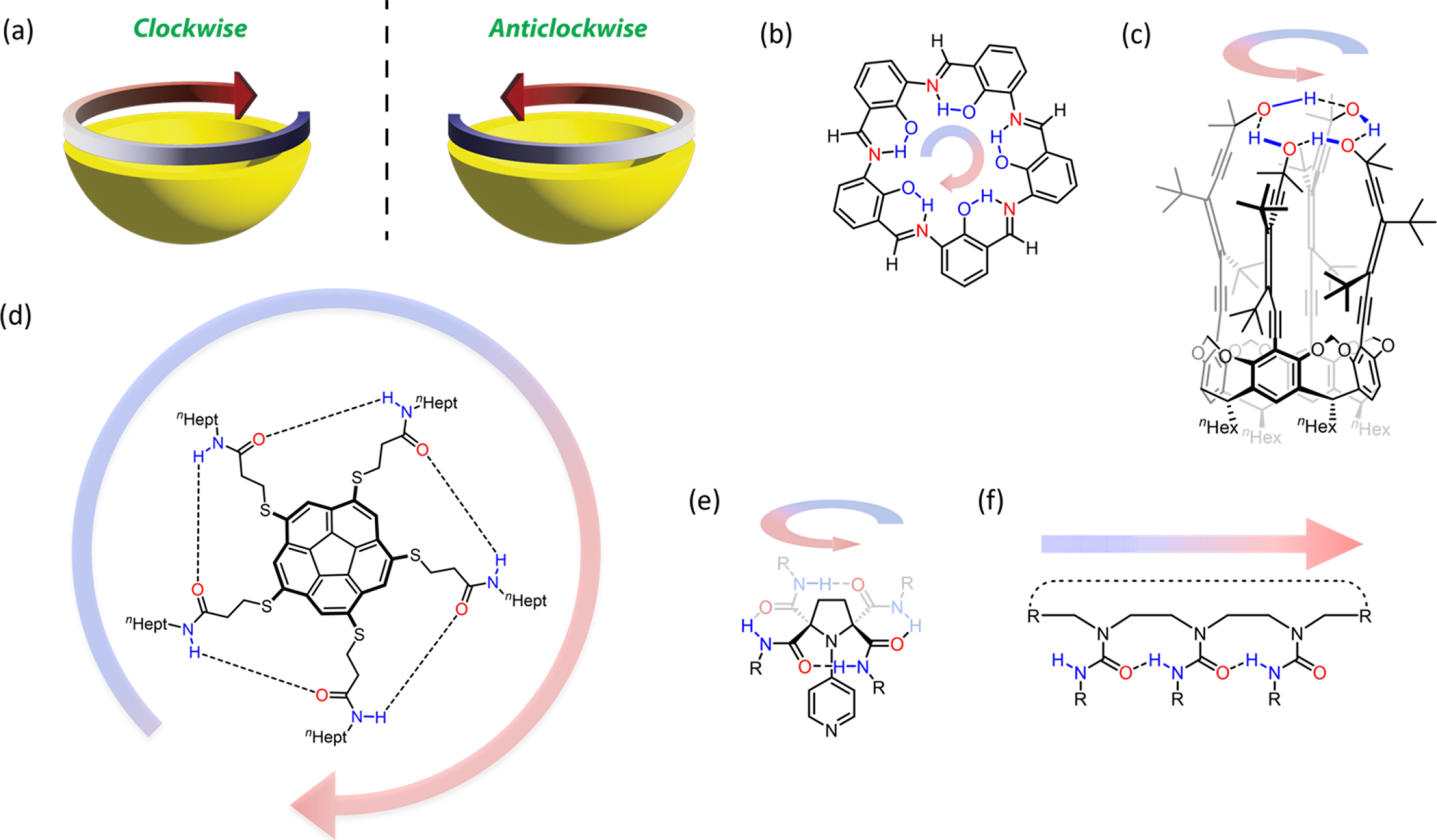
Cyclochirality arising from cyclic unidirectional hydrogen-bonding networks. (a) Cyclochiral molecules that lack a plane of symmetry. (b) Campestarenes. (c) Alleno-acetylenic cages. (d) Cyclochiral corannulenes. (e) Cyclochiral 4-pyrrolidinopyridines. (f) Ethylene-bridged oligoureas with uniform hydrogen-bond directionality.

Cyclic hydrogen-bonding networks are nonetheless rare in synthetic structures and are underexploited. Importantly, continuous cyclic hydrogen-bonded networks of functional groups (such as amides or ureas) that can act both as hydrogen bond acceptors and donors have a uniform hydrogen-bond directionality associated with them: hydrogen-bond acceptors can orient clockwise or anticlockwise (Figure 1a). In planar molecules such as the campestarenes (Figure 1b), these opposing directionalities are degenerate and the overall structure is achiral.^9^ However, in nonplanar molecules where the cyclic hydrogen-bonding network does not lie in a plane of symmetry, chirality ensues from the directionality of the network, and molecules with opposing hydrogen-bond directionalities are enantiomers of each other. This form of planar chirality has been referred to as “cyclochirality”, a term that has evolved from related but distinctly different earlier usage of “cycloenantiomerism” and “cyclostereoisomerism”.

Prelog and Gerlach first used “cycloenantiomerism” in the context of cyclic peptides to describe stereoisomers that possess the same cyclic arrangement of stereocenters but differ only in the direction of the ring.^44^ While this definition requires a cyclic arrangement of stereocenters, the concept of “cyclostereoisomerism” was expanded by Mislow^10,11^ to cover other aspects of isomerism that arise when cyclic arrangements of stereocenters are associated with ring systems. Since 2007,^12^ the term “cyclochirality” has been used in various contexts to describe the (often dynamic) chirality that arises in cyclic arrays where repulsive interactions such as steric hindrance^10,11^ or attractive interactions such as hydrogen bonds^12−16^ govern the coherent formation of alternative isomeric structures. In a few examples, contiguously hydrogen-bonded networks control the cyclochirality of the structure. Diederich and co-workers used axially chiral allenes to attain uniform hydrogen-bond directionality in a cyclic hydrogen-bonding network of four alcohols (Figure 1c).^17^ Similarly, Aida and co-workers slowed down bowl inversion^18−20^ in a helically chiral corannulene where, due to the chirality of the corannulene, uniform hydrogen-bond directionality was observed in a hydrogen-bonding network of five amides (Figure 1d).^21^ These examples constitute cyclic hydrogen-bonding networks whose directionality is biased by other chiral elements. Kawabata and co-workers created a cyclochiral hydrogen-bonding network that was stable toward racemization by appending four amides to a 4-pyrrolidinopyridine (Figure 1e).^22^ The chirality of this scaffold arises solely from the stability of the robust hydrogen-bonding network. The atropisomeric enantiomers were separable and were able to catalyze an asymmetric kinetic resolution of a chiral secondary alcohol.

We have previously shown that linear chains of ureas linked through ethylene bridges adopt a coherent and uniform hydrogen-bond directionality (Figure 1f) with a hydrogen bond-donating terminus and a hydrogen bond-accepting terminus.^23−26^ These molecules pack into cyclic supramolecular structures in the solid state that allow their terminal hydrogen-bonding capacity to be satisfied in an intermolecular manner.^25^ We speculated that linking the termini of an ethylene-bridged scaffold into a ring (Figure 1f, dashed line) could lead to cyclic, fully intramolecularly hydrogen-bonded structures, in which the cyclic hydrogen-bonding network may result in a new class of cyclochiral structure.

## Results and Discussion

**Figure 2:**
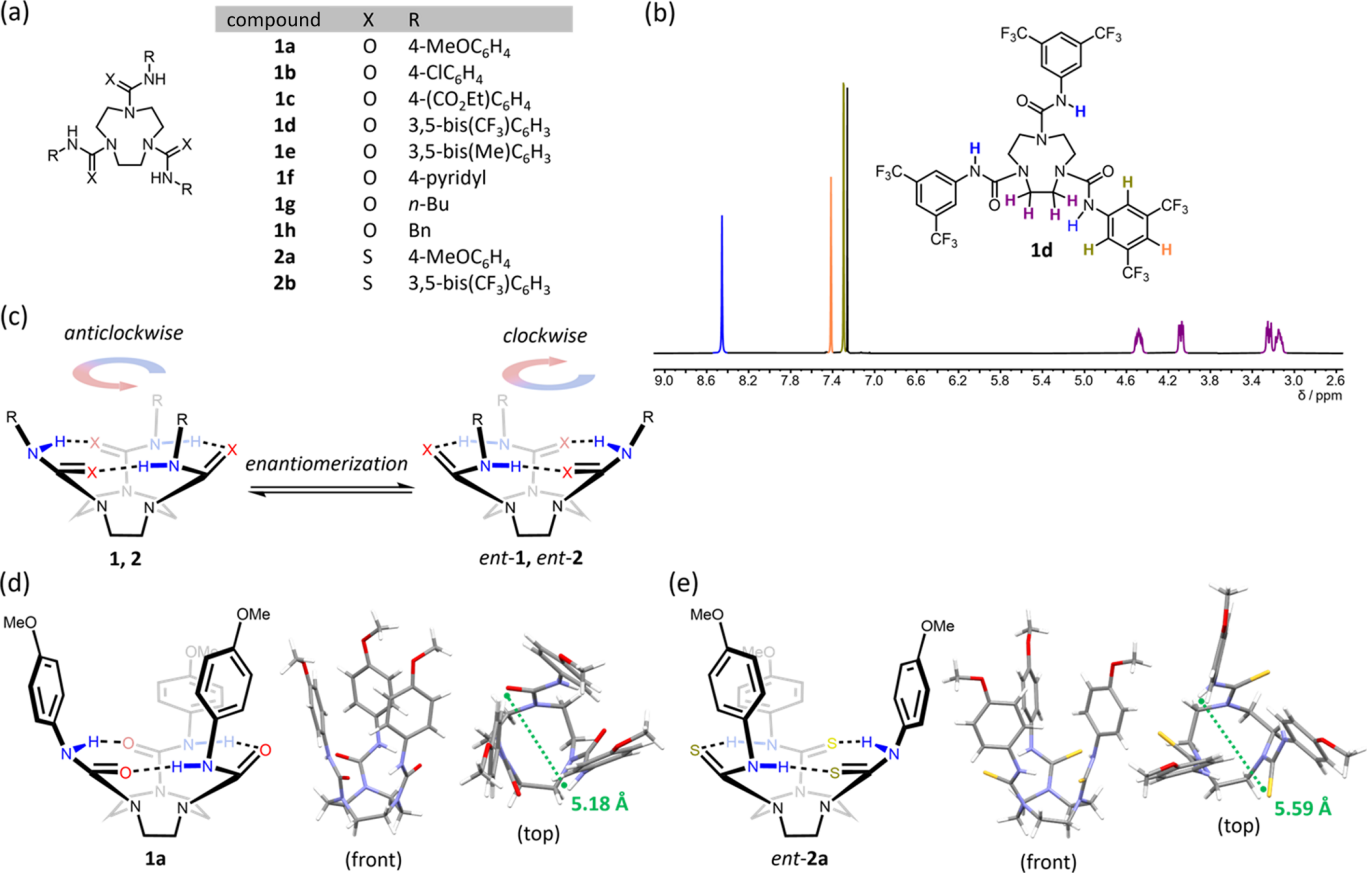
(a) TACN-derived tri(thio)ureas **1a–h** and **2a**,**b**. (b) ^1^H NMR spectrum of **1d** (15 mM, CDCl_3_, 500 MHz). (c) Lowest energy, chiral conformation of **1** and **2** with a cyclic hydrogen-bonding network that can take on either an anticlockwise or clockwise directionality, as viewed from the top of the structure. (d) Front and top views of the X-ray crystal structure of **1a** (CCDC: 2262692). Only one of the two molecules in the asymmetric unit is shown and disorder is omitted for clarity. (e) Front and top views of the X-ray crystal structure of *ent*-**2a** (CCDC: 2262693); an additional crystal structure for **2b** (CCDC: 2262694) is provided in Figure S67.

A variety of triureas **1a**–**e** and trithioureas **2a**–**b** were made from commercially available 1,4,7-triazacyclononane (TACN) and an appropriate aryl iso(thio)cyanate (Figure 2a). Intriguingly, the ^1^H NMR spectra in CDCl_3_ of all compounds at room temperature (exemplified by **1d**, Figure 2b) clearly displayed four resolved diastereotopic proton environments corresponding to the ethylene bridges and a single set of signals for the three arenes. Furthermore, a sharp downfield (δ_Η_ = 8.45 ppm for **1d**) singlet was observed for the three N–H protons, consistent with a hydrogen-bonded environment. Taken together, these initial observations suggested that **1** and **2** possess a cyclic hydrogen-bonding network that enforces folding into a chiral, C_3_-symmetrical, bowl-shaped conformation (Figure 2c),^27,28^ which must enantiomerize only slowly on the NMR timescale to preserve the four nonequivalent TACN proton environments.

X-ray crystal structures of **1a** and **2a** (Figure 2d,e)^29^ confirmed that a cyclochiral conformation is also adopted in the solid state. Both **1a** and **2a** possess 4-methoxyphenyl rings and bear similar structural features in the solid state despite **1a** having urea functions and **2a** having thiourea functions. To position the aryl rings into a bowl shape, each ethylene bridge (N–C–C–N linkage) of **1a** and **2a** adopts a *gauche* conformation,^23^ with a common preference for the hydrogen proximal to the N–H to assume a pseudoaxial orientation on the TACN ring and the hydrogen proximal to the (thio)carbonyl to assume a pseudoequatorial orientation. The directionality of the hydrogen bonds thus seems intimately coupled with the ±60° dihedral angle of each ethylene linker.

Three hydrogen bonds link the (thio)urea functions in each crystal structure (Figure 2d,e). In **1a**, two molecules are present in the asymmetric unit, one of which does not contain disorder on atoms involved in the hydrogen-bonding network; this structure has an average hydrogen bond length of 2.20 ± 0.04 Å (H···O distance) and an average N–H···O angle of 150 ± 3°. Due to the symmetry present in the crystal structure of **2a**, all three hydrogen bonds are identical, measuring 2.53 ± 0.03 Å (H···S distance) and 178 ± 3° (N–H···S angle). While forming approximately linear hydrogen bonds, the longer hydrogen bond lengths in **2a** can be attributed to the greater size of sulfur and longer C=S bonds. Interestingly, the C=S···H angles in **2a** are 82.2 ± 0.7°, compared to an average of 106.2 ± 0.9° for the C=O···H angles in **1a**, suggesting that the hydrogen bonds in **2a** are in fact N–H···π hydrogen bonds. The approximate diameter of the cyclic hydrogen bond network in **1a** (5.18 ± 0.03 Å) is slightly smaller than in **2a** (5.59 ± 0.03 Å), presumably to accommodate the longer hydrogen bonds and C=S bond lengths in **2a**.

**Figure 3:**
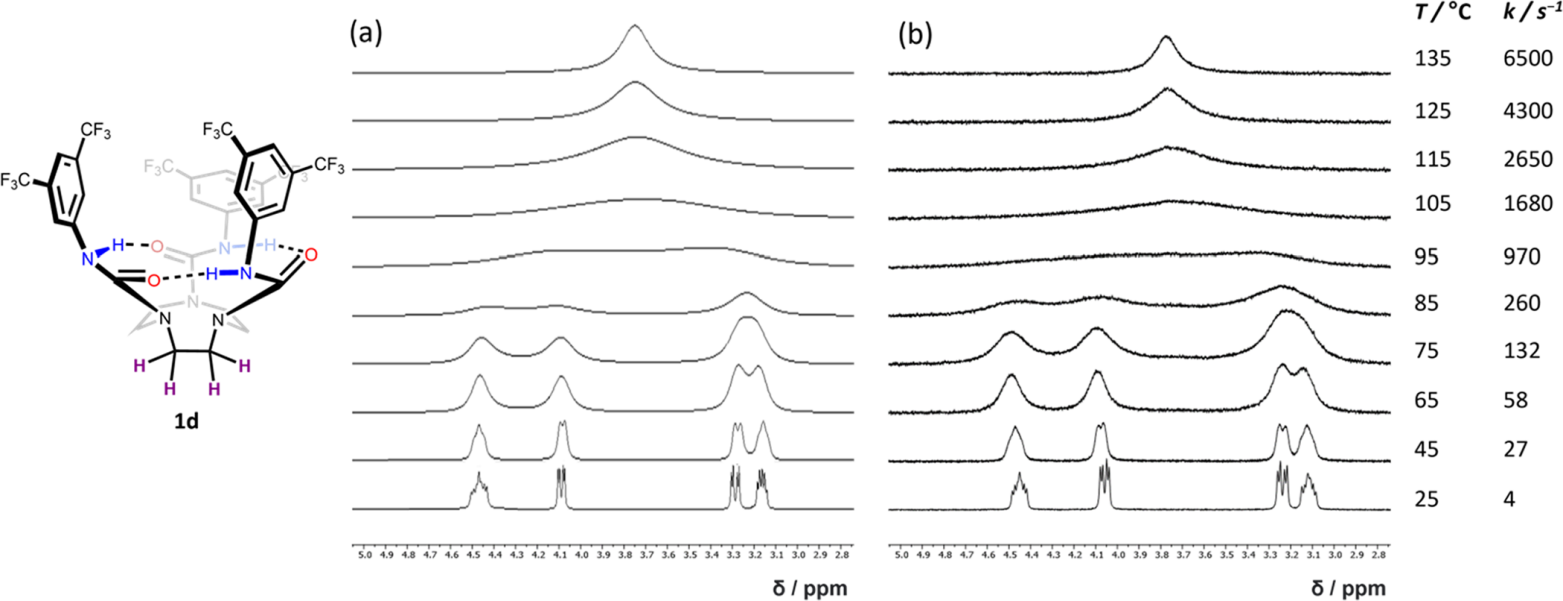
(a) Simulated and (b) experimental VT ^1^H NMR spectra for **1d** in *d*_2_-tetrachloroethane (*d*_2_-TCE) showing the ethylene bridge proton signals (colored purple on the structure).

To investigate the steric and electronic effects on the enantiomerization barrier of **1** and **2**, variable-temperature (VT) ^1^H NMR analysis was performed in nonpolar solvents (Figures S1–S11). In all cases, on raising the temperature, the diastereotopic proton resonances of the ethylene bridges underwent exchange broadening and eventually coalescence to a singlet (as exemplified by **1d**, Figure 3a), indicating that the chirality arising from the cyclic hydrogen-bonding network is dynamic in solution. As each of the experimental signals appeared to broaden at the same temperatures, the spectra were simulated with the initial assumption that the four TACN protons exchange at the same rate, which gave excellent fits with the experimental data (Figure 3b). The extraction of rate constants and Eyring analysis (Tables S1–S11) allowed the determination of the enantiomerization barriers (Δ*G*^‡^_25°C_) listed in Table 1.

**Table 1:**
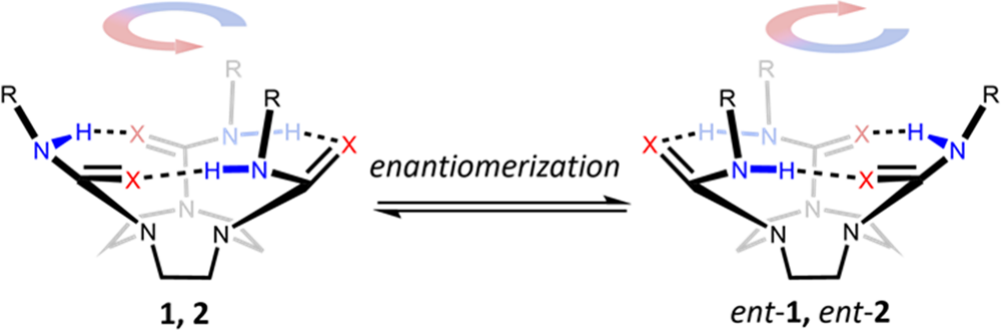
Enantiomerization Barriers of TACN Tri(thio)urea Derivatives **1a**–**g** and **2a,b** with a Cyclic Hydrogen-Bonding Network^a^

entry	compound	X	R	solvent	Δ*G*^‡^_25°C_ (kJ mol^–1^)
1	**1a**	O	4 MeOC_6_H_4_	*d*_2_-TCE	63.2
2	**1a**	O	4-MeOC_6_H_4_	*d*_8_-toluene	65.3
3	**1b**	O	4-ClC_6_H_4_	*d*_8_-toluene	63.2
4	**1c**	O	4-(CO_2_Et)C_6_H_4_	*d*_8_-toluene	65.1
5	**1d**	O	3,5-bis(CF_3_)C_6_H_3_	*d*_8_-toluene	67.5
6	**1d**	O	3,5-bis(CF_3_)C_6_H_3_	*d*_2_-TCE	70.0
7	**1e**	O	3,5-bis(Me)C_6_H_3_	*d*_2_-TCE	64.4
8	**1f**	O	4-pyridyl	*d*_2_-TCE	64.5
9	**1g**	O	*n*-Bu	CDCl_3_	49.0
10	**2a**	S	4-MeOC_6_H_4_	*d*_2_-TCE	62.4
11	**2b**	S	3,5-bis(CF_3_)C_6_H_3_	*d*_2_-TCE	70.5

a Spectral simulations were performed assuming all four diastereotopic TACN protons exchange at the same rate. *d*_2_-TCE = *d*_2_-tetrachloroethane.

The range of enantiomerization barriers for aryl triureas **1a**–**d** was relatively small (Δ*G*^‡^_25°C_ = 63.2–67.5 kJ mol^–1^ in *d*_8_-toluene; Table 1, entries 2–5), with the highest barrier being observed for **1d** bearing 3,5-bis(trifluoromethyl)phenyl (BTMP) ureas. The BTMP substituent, while promoting solubility, raised the barrier (Δ*G*^‡^_25°C_) of **1d** to 67.5 kJ mol^–1^ in *d*_8_-toluene (entry 5) and 70.0 kJ mol^–1^ in *d*_2_-tetrachloroethane (*d*_2_-TCE, entry 6). The higher barrier of **1d** was confirmed to be a result of the electronic deficiency of the BTMP substituent rather than a steric effect, after replacement with a 3,5-dimethylphenyl group in **1e** reduced the barrier to 64.4 kJ mol^–1^ in *d*_2_-TCE (entry 7). An analogue with 4-pyridyl ureas (**1f**) had an enantiomerization barrier of 64.5 kJ mol^–1^ in *d*_2_-TCE (entry 8).

The lowest enantiomerization barrier was determined for **1g** bearing butyl ureas (Δ*G*^‡^_25°C_ = 49.0 kJ mol^–1^, Table 1, entry 9), in which case the TACN protons appeared as a single broad singlet at room temperature that decoalesced into four broad signals on lowering the temperature to −20 °C (Figure S9). An *N*-benzylated triurea derivative **1h** (Figure 2a) also showed a single broad singlet at room temperature corresponding to the TACN protons (Figure S85). These results confirm that *N*-aryl urea substituents impart a greater degree of kinetic stability to the cyclochiral hydrogen-bonding network. Replacing the urea functions in compound **1a** with thioureas (**2a**) did not change the enantiomerization barrier significantly (entry 1 versus entry 10), showing that the aryl group has a greater influence on the enantiomerization rate than the hydrogen bond-accepting atom (O or S). Consistent with this result, BTMP thiourea **2b** (Δ*G*^‡^_25°C_ = 70.5 kJ mol^–1^ in *d*_2_-TCE) had an enantiomerization barrier similar to BTMP urea **1d** (entry 6 versus entry 11). Loss of hydrogen bond-accepting ability on changing from ureas to thioureas is possibly compensated by the hydrogen-bonding geometry (N–H···X angle) being closer to optimal for thioureas and by the greater hydrogen bond-donating ability of a thiourea NH.

**Figure 4:**
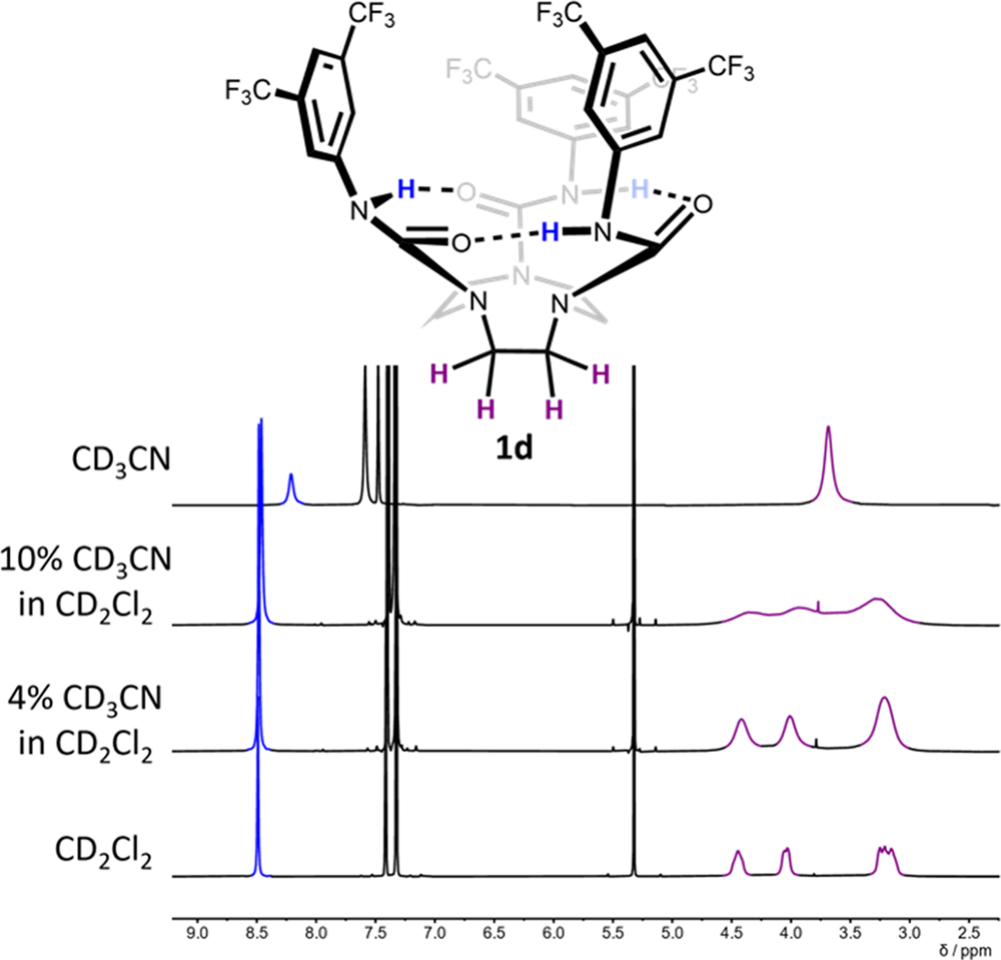
^1^H NMR spectra for **1d** (10 mM, 25 °C, 400 MHz) with increasing amounts of CD_3_CN in CD_2_Cl_2_ (bottom to top). Solvent percentages are by volume.

Even in hydrogen-bonding solvents, the intramolecular hydrogen bond network of **1** and **2** is retained: the chemical shift of the N–H signal of **1d** remains constant across a range of solvents (δ_H_ = 8.18–8.50 ppm at 10–15 mM in *d*_6_-benzene, *d*_2_-TCE, CDCl_3_, CD_2_Cl_2_, CD_3_CN, *d*_6_-acetone, and *d*_3_-MeOH; Figure 2b, Figures 4, S19, S24, and S27). However, a broad singlet is observed for the ethylene bridge protons at room temperature for **1d** in polar and/or hydrogen-bonding solvents (Figure S24), indicating fast exchange between enantiomeric conformers. As an example, Figure 4 shows the ^1^H NMR spectra of **1d** with increasing proportions of CD_3_CN in CD_2_Cl_2_ (0–100% v/v), which progressively lowers the enantiomerization barrier, increasing the rate of proton exchange at 25 °C. Presumably, hydrogen-bonding solvents decrease the enthalpic penalty of breaking the cyclic hydrogen bond network during enantiomerization.

**Figure 5:**
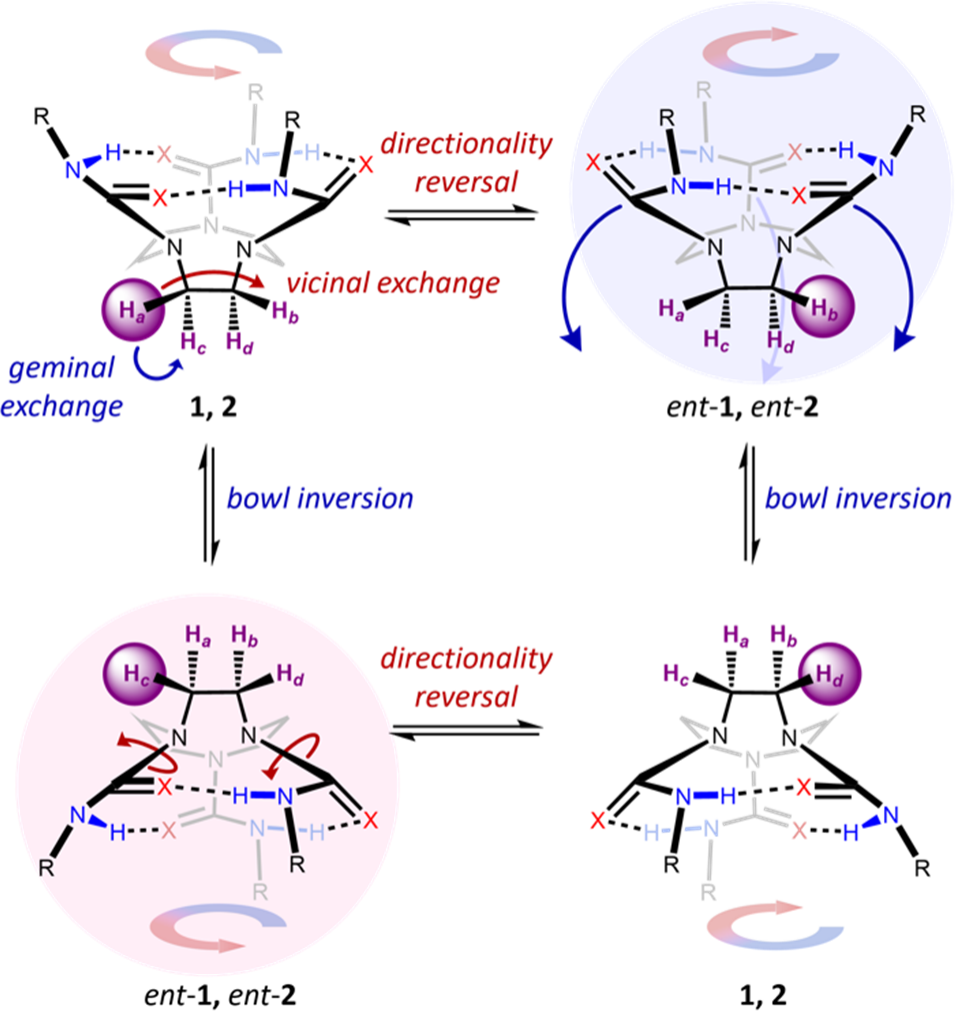
Potential enantiomerization mechanisms for TACN-derived tri(thio)ureas. Each discrete operation of directionality reversal or bowl inversion results in enantiomerization of the molecule. The movement of the proton highlighted by the purple sphere shows the chemical shift exchange processes occurring between the four TACN protons (labeled a, b, c, and d).

The observation that all four diastereotopic ethylene bridge protons of **1** and **2** coalesce to a singlet during the VT NMR and solvent studies shows that each proton must undergo exchange with its geminal partner and with both its *syn* and *anti* vicinal partners. For this to be the case, two mechanisms of enantiomerization must be occurring: (1) bowl inversion,^18−20^ where the ring of hydrogen bonds break and then reform on the opposite face of TACN, and (2) directionality reversal, where the C–N bonds of the ureas rotate through 180° and the hydrogen bonds reform on the same face of TACN, but oriented in the opposite direction.^23^ Although bowl inversion and directionality reversal have the same consequence—that is, they both interconvert one enantiomer into the other—the two mechanisms have different consequences with respect to the protons that exchange: bowl inversion exchanges geminal protons, while directionality reversal exchanges *syn* vicinal protons (Figure 5). Sequential or simultaneous operation of both enantiomerization processes allows net exchange of *anti* vicinal protons, but concerted exchange would result in two proton environments at high temperature and can thus be ruled out (Figure 3).

**Figure 6:**
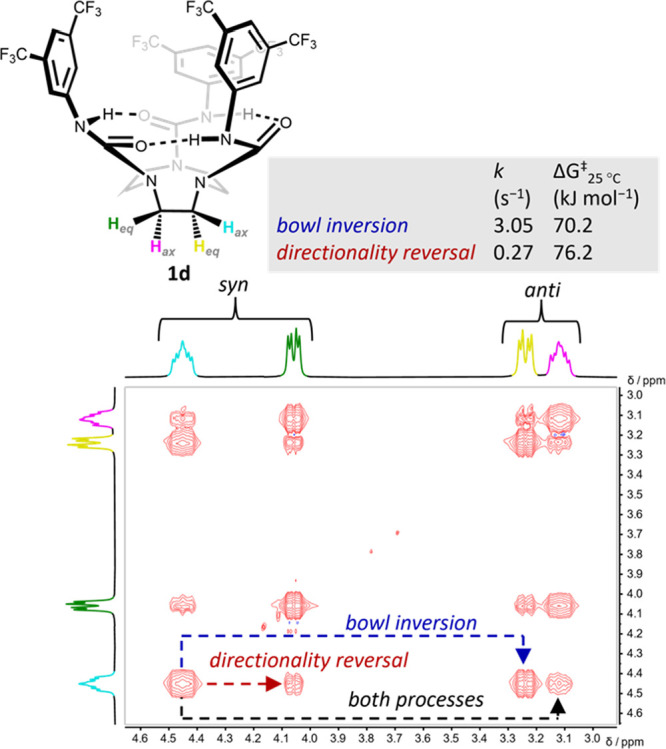
^1^H-^1^H EXSY NMR spectrum of **1d** (10 mM, *d*_2_-TCE, 25 °C, 500 MHz, 300 ms mixing time) showing a full exchange matrix between all the individual spins. The spectrum allowed the extraction of the corresponding exchange rates for bowl and directionality reversal (and their Gibbs free energy of activation) on comparison with a spectrum acquired at “zero” mixing time (Figure S21). Shortening the mixing time to 100 ms gave identical rates. *Syn* and *anti* refer to the orientation of the proton relative to the hydrogen-bonding network. H_ax_ and H_eq_ refer to protons in pseudoaxial and pseudoequatorial positions, respectively.

Compound **1d** displays sharp, well-resolved signals in its ^1^H NMR spectrum in nonpolar solvents at room temperature (Figures 2b and 4) and was chosen as a model to investigate the kinetics of the enantiomerization processes in more detail. The ^1^H NMR signals corresponding to the ethylene bridge protons (colored distinctly in Figure 6) were readily assigned by a combination of COSY, HSQC, NOESY, and coupling constant analysis (Figures S16–S18), establishing that the protons oriented *syn* to the BTMP groups (that make up the bowl-like cavity) appear further downfield than the protons oriented *anti* to the BTMP groups (Figure 6). The scalar coupling is consistent with a rigid gauche conformation for the ethylene bridges (Figure S18), with the turquoise hydrogen (proximal to N–H) and the green hydrogen (proximal to C=O) occupying pseudoaxial and pseudoequatorial positions, respectively, as a result of the defined gauche conformations seen in the X-ray structures of **1a** and **2a** (Figure 2d,e).

Quantitative two-dimensional exchange NMR spectroscopy (^1^H-^1^H EXSY) experiments in *d*_2_-TCE at 25 °C revealed rate constants^30−33^ for the bowl inversion and directionality reversal of **1d** of 3.05 ± 0.10 and 0.27 ± 0.05 s^–1^, respectively, corresponding to energy barriers (Δ*G*^‡^_25°C_) of 70.2 kJ mol^–1^ for bowl inversion and 76.2 kJ mol^–1^ for directionality reversal (Figure 6). The bowl inversion of **1d** is thus an order of magnitude faster than its directionality reversal in *d*_2_-TCE, which is also the case in *d*_6_-benzene, where similar energy barriers were obtained (Table S23). Evidently, an enantiomerization barrier of 70.0 kJ mol^–1^ determined for **1d** by VT NMR in *d*_2_-TCE (Table 1, entry 6), in which the discrete exchange processes cannot be distinguished, is a good representation of the kinetics of the (faster) bowl inversion process.

The fact that bowl inversion preserves the directionality of the hydrogen bonds, despite the fact that hydrogen bonds must be broken during the inversion, may arise because one hydrogen bond remains intact during the inversion, but may also simply be the result of the slow rate of C–N bond rotation. Similar situations where conformation is preserved despite the temporary rupture of hydrogen bonds arise when “faults” form in helical hydrogen-bonded systems.^33b^

**Figure 7:**
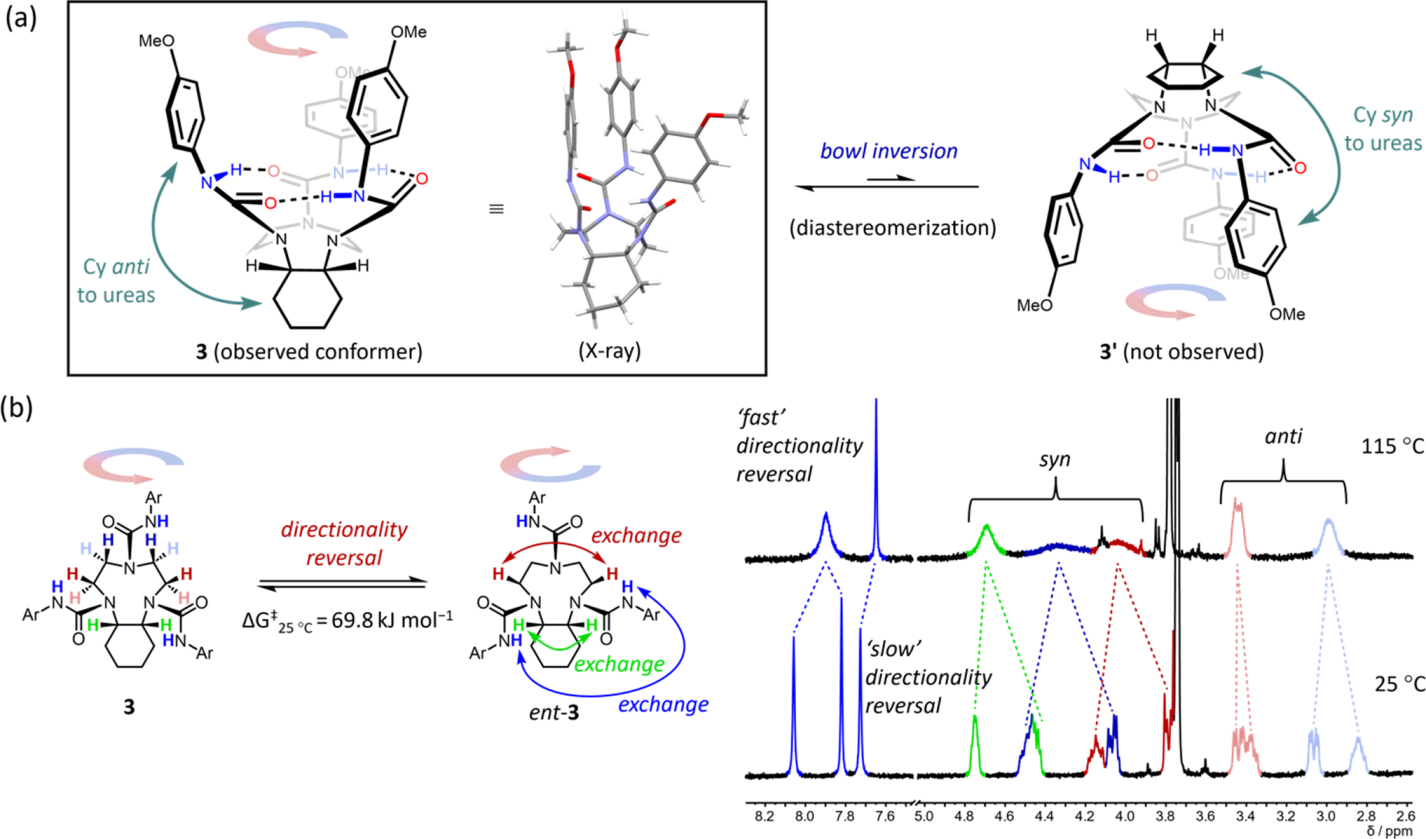
(a) TACN triurea derivative **3** with a *cis*-annulated cyclohexane ring. The observed conformer of **3** is depicted in the box along with its X-ray crystal structure (CCDC: 2262695; solvent molecules omitted for clarity). Another possible conformational diastereomer **3′** was not observed. (b) Enantiomerization of **3** occurs by directionality reversal (Δ*G*^‡^_25°C_ = 69.8 kJ mol^–1^ in *d*_2_-TCE). Selected VT ^1^H NMR spectra of **3** (10 mM, *d*_2_-TCE, 500 MHz) at 25 and 115 °C. *Syn* and *anti* refer to the orientation of the proton relative to the hydrogen-bonding network. Ar = 4-OMeC_6_H_4_.

Breaking the degeneracy of the structures interconverted by bowl inversion could be achieved by introducing two different geminal substituents on the ethylene linkage of the TACN ring, which would prevent enantiomerization by bowl inversion: this process would then lead to a different diastereoisomeric conformer, and appropriate substitution could raise its energy sufficiently to prevent its population.^34,35^ Compound **3** was therefore prepared as an analogue of **1a** with a *cis*-annulated cyclohexane ring (Figure 7a). In **3**, the *meso* configuration serves to maintain the energetic degeneracy of the enantiomeric isomers that result from directionality reversal, while rendering the conformers of the molecule that result from bowl inversion diastereomeric (**3** and **3′**, Figure 7a).

As desired, a single diastereomeric conformer was observed for **3** by NMR in CDCl_3_ at both 25 and −30 °C (Figure S28). ROESY NMR studies (Figures S30–S32) showed that the cyclohexane was oriented *anti* to the aryl ureas (Figure 7a). An X-ray structure confirmed this *anti* conformation in the solid state and revealed a twist-boat conformation of the cyclohexane ring. DFT calculations (B3LYP-D3(BJ)/def2-TZVPP/SMD (MeCN)) predicted an alternative conformation **3′** (Figure 7a) with the cyclohexane and aryl groups in a *syn* orientation to be 10.3 kJ mol^–1^ higher in energy (Δ*G*°_25°C_) than **3**, in agreement with the observation of only a single diastereomeric conformer by NMR.

Ten distinct resonances were observed for the TACN protons of **3** at 25 °C (Figure 7b), as well as three separate N–H signals. Enantiomerization of its *C*_1_-symmetric ground-state conformation must therefore be slow on the NMR timescale at room temperature. Heating to 115 °C in *d*_2_-TCE halved the number of TACN proton resonances, introducing an apparent vertical plane of symmetry bisecting the C–C bond fusing cyclohexane to TACN, giving the structure *C*_v_ symmetry. The (green) signals from the vicinal methine protons (that are part of the cyclohexane ring) and the two (blue) N–H signals proximal to the cyclohexane also coalesced. These observations are consistent with enantiomerization by directionality reversal (Figure 7b), with a barrier of Δ*G*^‡^_25°C_ = 69.8 kJ mol^–1^ (*d*_2_-TCE) determined by line shape and Eyring analysis (Table S12). This value is 6.6 kJ mol^–1^ higher (ΔΔ*G*^‡^_25°C_) than the enantiomerization barrier determined for **1a** (Table 1, entry 1), which lacks the cyclohexane ring and can undergo (more facile) bowl inversion as an alternative enantiomerization pathway. EXSY studies of **1d** (Figure 6) showed a very similar energetic difference between directionality reversal and bowl inversion (ΔΔ*G*^‡^_25°C_ = +6.0 kJ mol^–1^ for directionality reversal), implying that although the *cis*-fused cyclohexane ring prevents bowl inversion, the barrier to directionality reversal is essentially unaffected.

**Figure 8:**
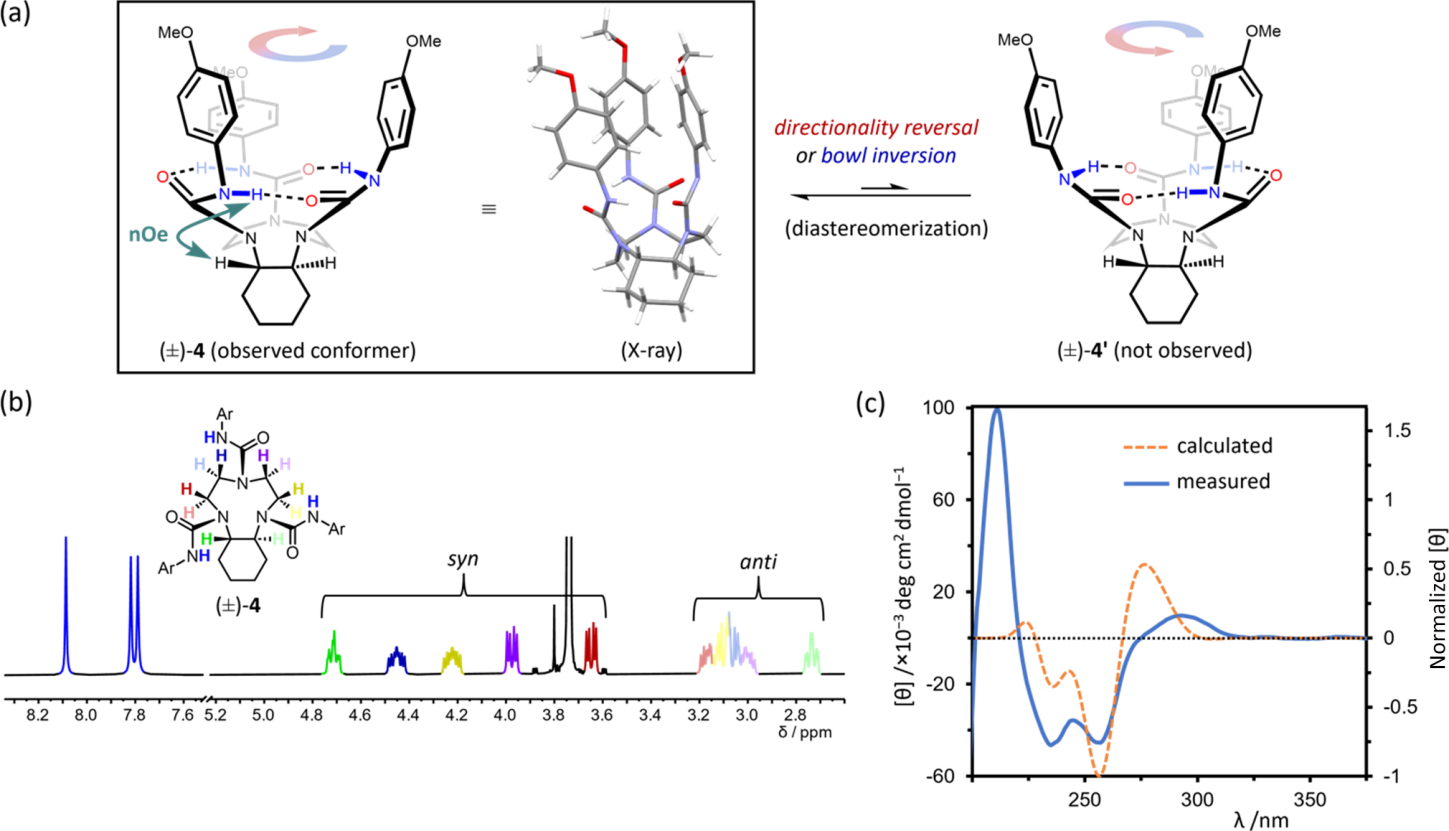
(a) TACN triurea derivative (±)-**4** with a *trans*-annulated cyclohexane ring. The observed conformer of **4** is depicted in the box showing the *S*,*S*-configuration of the stereogenic centers, along with the X-ray structure of the *S*,*S*-enantiomer from a racemic crystal (CCDC: 2262696; disorder omitted for clarity). Another possible conformational diastereomer **4′** was not observed. (b) ^1^H NMR spectrum of **4** (9 mM, CDCl_3_, 25 °C, 500 MHz). *Syn* and *anti* refer to the orientation of the proton relative to the hydrogen-bonding network. Ar = 4-OMeC_6_H_4_. (c) Measured and calculated (PBE0/def2-TZVPP/CPCM(MeCN)) circular dichroism (CD) spectra of enantiopure (*S*,*S*)-**4** in MeCN (experimental conditions: 0.25 mM, 25 °C, *l* = 1 mm). The measured spectrum is plotted with classical units of molar ellipticity (left *y*-axis) and the calculated spectrum is plotted with normalized molar ellipticity (right *y*-axis).

Introducing an element of chirality into one of the ethylene bridges of **1** would mean that both bowl inversion and directionality reversal would lead to energetically nondegenerate diastereoisomeric conformers and could induce a single chiral hydrogen-bonded conformation across the entire structure. The *trans*-annulated cyclohexane derivative (±)-**4** was prepared (Figure 8a). The local C_2_ symmetry of the *trans*-fused cyclohexane ring is a strategic design feature that prevents interconversion between + and – gauche conformers and therefore limits the system to just two possible diastereomeric conformers **4** and **4′** with oppositely polarized cyclic hydrogen-bonding networks.

The ^1^H NMR spectra of **4** in CDCl_3_ at room temperature (Figure 8b) and from −30 to 60 °C (Figure S34) showed no broadening or decoalescence indicative of multiple conformers. Compound **4** therefore exists in a single diastereomeric conformation, demonstrating that the configuration of the *trans*-fused cyclohexane controls completely the cyclochirality of the adjacent hydrogen-bonding network—a notable result considering that other cyclochiral hydrogen-bonding networks are less sensitive to adjacent chiral elements.^22^

X-ray analysis of crystals grown from (±)-**4** (Figure 8a) revealed the relative stereochemical relationship between the fixed stereocenters and the cyclochiral hydrogen-bonding network. The *S*,*S*-stereochemistry at the ring junction (as drawn) defines a C–C dihedral angle that enforces the hydrogen-bonding directionality where the C=O···H–N linkages turn clockwise as seen from the top of the structure. This conformational preference is the same in CDCl_3_, where a strong NOE is apparent between the axial methine hydrogen lying above the plane of the ring and the N–H proximal to the cyclohexane (Figure S36). It thus appears that the directionality of the hydrogen-bonding network places the axial methine hydrogen proximal to a N–H instead of a carbonyl group. This also echoes the conformational preferences discussed for compounds **1** and **2**, where the hydrogens proximal to the N–Hs occupy a pseudoaxial position on the TACN ring instead of a pseudoequatorial position.

The same diastereomer of **4** was also exclusively observed in the hydrogen bond-accepting solvent CD_3_CN (as confirmed by NOESY, Figure S40). Earlier studies showed that the TACN protons of **1d** resonate as a broad singlet in CD_3_CN, owing to an increased rate of enantiomerization (Figure 4), but the 10 TACN protons of **4** remain sharp and well resolved in CD_3_CN (Figure S39), indicating that dynamic conformational interconversions no longer take place. These results further show that the hydrogen-bonding network and associated bowl-like conformation of **1**–**4** is maintained in hydrogen-bonding solvents and that the ability to induce a single sense of cyclochirality using an asymmetrically substituted ethylene bridge should extend across a wide solvent range.

An alternative diastereomer **4′** (Figure 8a) where the *S*,*S*-configured cyclohexane (as drawn) is associated with an anticlockwise directionality of the hydrogen bond network was predicted by DFT calculations in MeCN to be >30 kJ mol^–1^ higher in energy (Δ*G*°_25°C_) than the observed lowest energy conformation of **4** (Table S29). These calculations reveal stronger hydrogen bonding in **4** relative to **4′** (Tables S27 and S28). Most notably, the hydrogen bond that spans the ring-fused cyclohexane is shorter in **4** (H···O distance of 1.89 Å) than in **4**′ (H···O distance of 2.21 Å) and is significantly more linear in **4** (N–H···O angle of 161.2°) than in **4**′ (N–H···O angle of 133.3°).

The UV–vis spectrum of **4** was recorded in MeCN and shows broad but distinct maxima at 290 and 240 nm (Figure S57). The calculated absorption spectrum and natural transition orbital analysis (PBE0/def2-TZVPP/CPCM(MeCN)) predicts the peaks at 278 and 234 nm, respectively, to be due to HOMO–LUMO transitions with admixtures of other orbitals (mainly HOMO – 1 and LUMO + 1). Most of these orbitals are localized over two urea fragments. However, the LUMO is delocalized over three aromatic rings with electron density also in between the rings (Figures S58–S60). The CD spectrum of enantiopure (*S*,*S*)-**4** (Figure 8c) displays multiple maxima probably due to the frontier orbital localization on two out of three urea fragments. The calculated CD spectrum closely matches the experimental spectrum in MeCN (Figure 8c).

**Figure 9:**
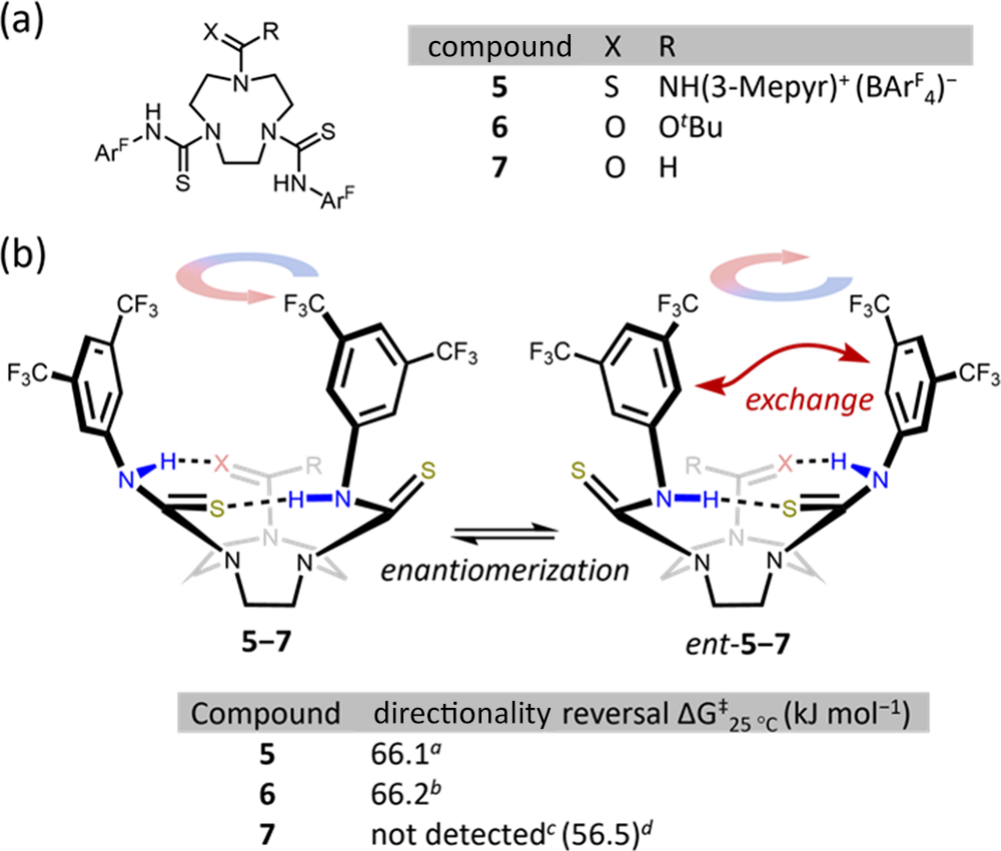
(a) Structures of TACN thiourea derivatives **5**–**7**. (b) Directionality reversal enantiomerization barriers of compounds **5**–**7** (*d*_2_-TCE, 9–13 mM) determined by VT ^1^H NMR and Eyring analysis. ^a^ Barrier determined by coalescence of BTMP thiourea N–H proton resonances; ^b^ barrier determined by coalescence of *ortho* BTMP proton resonances; ^c^ BTMP resonances remained resolved at 115 °C (see Figure 10); and ^d^ barrier for bowl inversion, determined by coalescence of geminal ethylene bridge protons. 3-Mepyr = 3-methylpyridinium. Ar^F^ = 3,5-bis(CF_3_)C_6_H_3_.

Although a mechanism to shut down bowl inversion had been identified in **3** (Figure 7), rapid reversal of directionality precluded the resolution of its enantiomers. To address this challenge, further structural and kinetic insight into the directionality reversal process was sought by systematically modifying the hydrogen-bonding network of trithiourea **2b**, which was the most stable toward enantiomerization of the simple TACN derivatives tested (Table 1, entry 11). Compounds **5**–**7** were prepared, each related to **2b** but with one of the BTMP thioureas replaced with a different hydrogen-bonding group (Figure 9a). Although enantiomerization by bowl inversion is still feasible in these model systems (**5**–**7**), the break in *C*_3_ symmetry allows kinetic information on directionality reversal to be extracted directly by following the rotational exchange of the (nonequivalent) BTMP groups (or BTMP thiourea N–Hs) by VT NMR. This is because the chemical environments of the BTMP groups in **5**–**7** are exchanged by directionality reversal but not by bowl inversion.

Replacement of BTMP thioureas with cationic, 3-methylpyridinium thioureas (and a noncoordinating BAr^F^_4_ counterion) has been shown to increase the activity of hydrogen-bond donor catalysts.^36,37^ One of the thiourea groups of **2b** was modified in this way to give **5** (Figure 9a). The barrier to directionality reversal of **5** in *d*_2_-TCE was determined to be Δ*G*^‡^_25°C_ = 66.1 kJ mol^–1^ (Figure 9b). Since the barrier for enantiomerization of **2b** is necessarily ≥70.5 kJ mol^–1^ (Table 1, entry 11; the VT NMR method for **1**–**2** does not distinguish bowl inversion and directionality reversal), swapping one BTMP thiourea for a cationic thiourea evidently has the undesired effect of increasing the rate of directionality reversal.

A BTMP thiourea was replaced with a *tert*-butyloxycarbonyl group (**6**, Figure 9a), capable only of functioning as a hydrogen-bond acceptor. Despite this change breaking the continuous cyclic hydrogen-bonding network, the directionality reversal barrier was maintained at Δ*G*^‡^_25°C_ = 66.2 kJ mol^–1^ (Figure 9b), indicating that directionality reversal is likely a nonconcerted process, not unlike the inversion of hydrogen bond chains in analogous linear polyureas.^23^ Encouraged by this result, we questioned whether a hydrogen bond-accepting group such as an amide with an innately higher barrier to N–C(O) bond rotation would increase even further the barrier to directionality reversal. Indeed, several dynamic, cyclochiral compounds are based on secondary amide hydrogen bond networks that run around the rim of their bowl-like structures,^21,22^ but directionality reversal of such systems is not associated with inversion of the local amide geometry.^3,4,38−40^ The opportunity to integrate amide N–C(O) bond rotation with directionality reversal appears to be unique to the present system.

**Figure 10:**
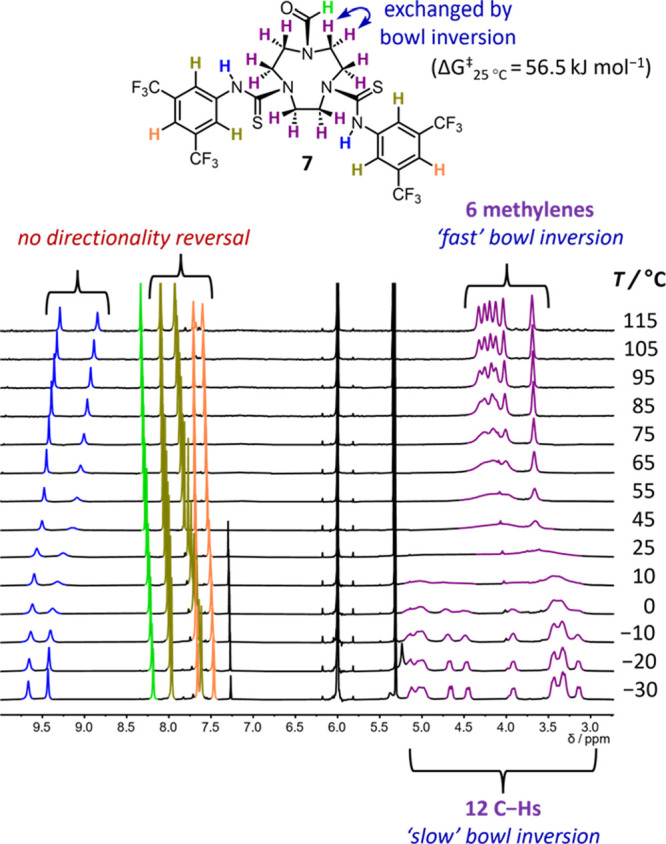
VT ^1^H NMR of compound **7** (13 mM, *d*_2_-TCE, 500 MHz). The absence of broadening or coalescence of the BTMP thiourea resonances (blue (N–Hs), gold (*ortho* ArHs), and orange (*para* ArHs)) indicates that no directionality reversal is occurring on the ^1^H NMR timescale at all temperatures studied.

Tertiary formamides exhibit relatively high rotational barriers about their C–N bonds^41^ and their associated *cis* and *trans* isomers can even be separated at ambient temperature in certain cases.^42^ Accordingly, we incorporated a formamide into the hydrogen bond network to give **7** (Figure 9a). Unlike **5** and **6**, VT ^1^H NMR analysis of **7** in *d*_2_-TCE up to 115 °C showed no signs of broadening or coalescence of the signals of the BTMP rings, nor their adjacent N–Hs (Figure 10), confirming a substantially increased barrier to directionality reversal relative to **1**–**3**, **5**, and **6**. In contrast, significant broadening of the TACN proton signals at 25 °C suggested a relatively low bowl inversion barrier, which is consistent with the expectation that bowl inversion does not require N–C(O) bond rotation.

Because directionality reversal and associated vicinal proton exchange in **7** are slow on the NMR time scale at the temperatures employed, in this case the bowl inversion kinetics could be extracted from the VT NMR data by following the exchanging geminal protons of one of the TACN methylene groups (Figure 10). At slow exchange, below 0 °C, the 12 different chemical environments of the TACN protons were evident, while at fast exchange (115 °C), each pair of diastereotopic methylene resonances coalesced, giving six broad triplets. The bowl inversion barrier calculated for **7** was Δ*G*^‡^_25°C_ = 56.5 kJ mol^–1^ (Figure 9b and Table S15), notably smaller than for **2b** (≥70.5 kJ mol^–1^) and all other triaryl (thio)ureas studied (Table 1). Collectively, these results substantiate our earlier proposal that bowl inversion and directionality reversal are fundamentally discrete processes.

**Figure 11:**
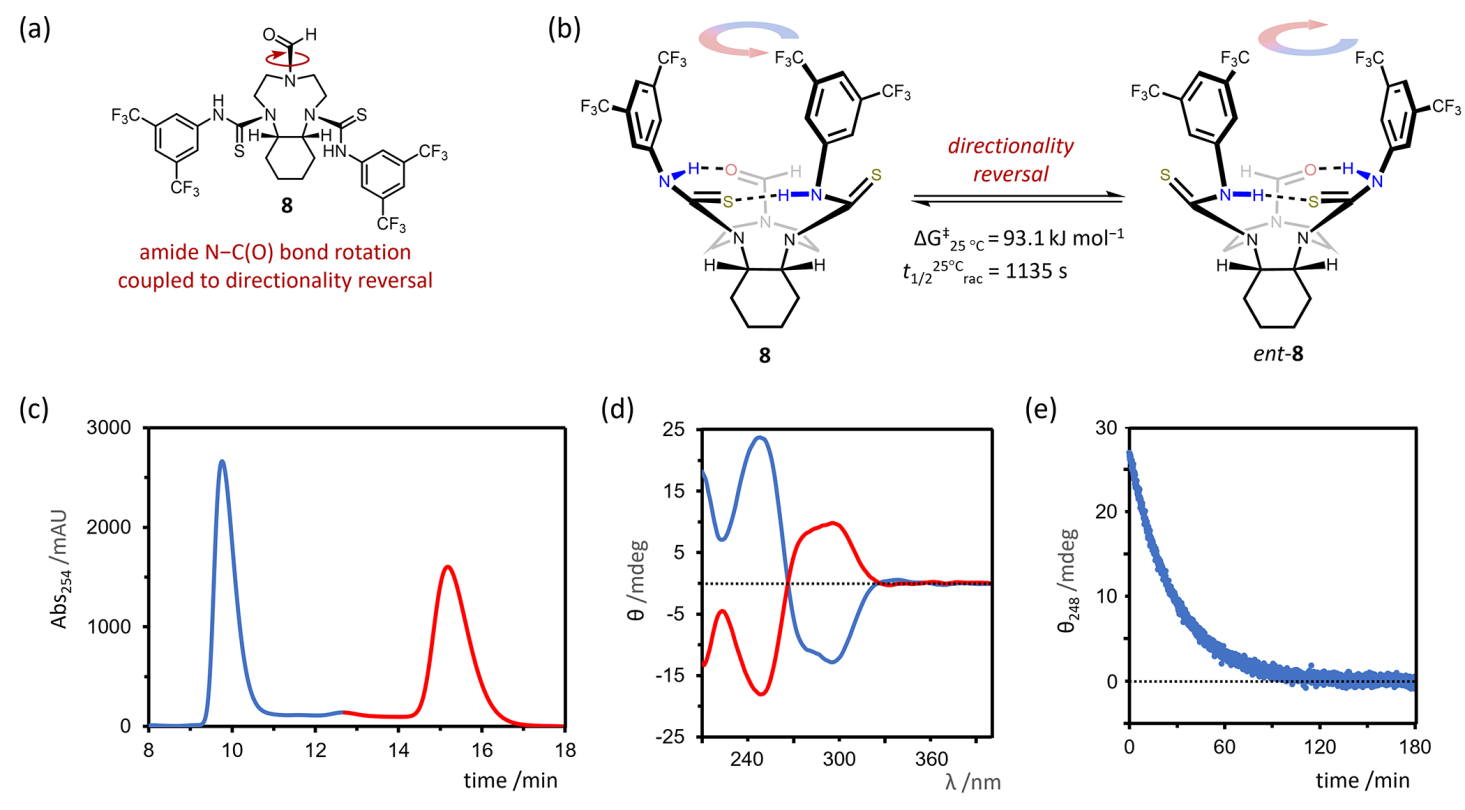
(a) Structure of TACN thiourea derivative **8** with a *cis*-annulated cyclohexane ring. (b) Lowest energy, atropisomeric conformation of **8**, which enantiomerizes slowly by directionality reversal (Δ*G*^‡^_25°C_ = 93.1 kJ mol^–1^ in 20% *i*-PrOH/hexane, *t*_1/2_^25^°^C^_rac_ = 1135 s). (c) HPLC chromatogram of (±)-**8** at a wavelength of 254 nm (OD-H chiral stationary phase, 20% *i*-PrOH/hexane, 1 mL min^–1^). (d) CD spectra of enantioenriched samples of **8** and *ent*-**8** (20% *i*-PrOH/hexane, 25 °C, *l* = 10 mm). The samples were obtained directly from fractions eluted from a chiral HPLC column (see Figure 11c) and are approximately 30 μM. The spectrum depicted in blue corresponds to the enantiomer with the shorter HPLC retention time. (e) Time course decay of the CD signal of an enantioenriched sample of **8** at a wavelength of 248 nm (∼33 μM, 20% *i*-PrOH/hexane, 25 °C, *l* = 10 mm). The sample is enriched initially in the enantiomer with the shorter HPLC retention time.

Having established that the formamide in **7** shuts down hydrogen-bond directionality reversal and that a *cis*-annulated cyclohexane in **3** shuts down bowl inversion, we incorporated both structural elements into compound **8** (Figure 11a). The cyclohexane ring positioned on the ethylene bridge between the BTMP thioureas preserves the *meso* stereochemistry and ensures sufficient steric interactions between the cyclohexane ring and the BTMP thioureas to favor the *anti* conformation. Indeed, like **3**, a single diastereomeric conformer was observed for **8** by NMR in CDCl_3_, with the intramolecular hydrogen bond network maintained and the cyclohexyl group disposed *anti* to the BTMP thioureas (Figures 11b and S41–S44). The lowest energy conformation of **8** identified by GFN2-xTB metadynamics and DFT calculations was predicted to be >20 kJ mol^–1^ (Δ*G*°_25°C_) more stable in MeCN than alternative conformer(s) **8′** with the cyclohexane ring *syn* to the hydrogen bond network (Table S31).

Encouragingly, no coalescence of the BTMP urea or TACN proton signals of **8** were observed by VT ^1^H NMR analysis up to 125 °C (Figures S45 and S46), and no exchange cross peaks were detected by EXSY at 52 °C with a 500 ms mixing time (Figure S43), indicating a high barrier to enantiomerization by directionality reversal. Enantioenriched samples of **8** and *ent*-**8** were obtained by semipreparative HPLC on an OD-H chiral stationary phase (Figure 11c) and the CD spectrum of each enantiomer was recorded in 20% isopropanol/hexane (Figure 11d). Monitoring the decay of the CD signal intensity over time (Figure 11e) allowed an enantiomerization barrier of Δ*G*^‡^_25°C_ = 93.1 kJ mol^–1^ (20% isopropanol/hexane) to be determined, corresponding to a racemization half-life of 1135 s at 25 °C and indicating that **8** is a chiral, atropisomeric structure under these conditions.

Similar enantiomerization barriers were determined for **8** in CHCl_3_ (Δ*G*^‡^_25°C_ = 92.5 kJ mol^–1^, Figure S50) and DMSO (Δ*G*^‡^_25°C_ = 93.2 kJ mol^–1^, Figure S51), showing that coupling amide N–C(O) bond rotation to directionality reversal in this system allows the kinetics of enantiomerization to be tuned independently of solvent polarity and hydrogen-bonding capacity. Eyring analyses confirmed that enthalpy was the major contributor to the enantiomerization barrier, with a negative entropy of activation in the hydrogen-bonding solvent 20% isopropanol/hexane and a positive entropy of activation in chloroform (Table S25).

**Figure 12:**
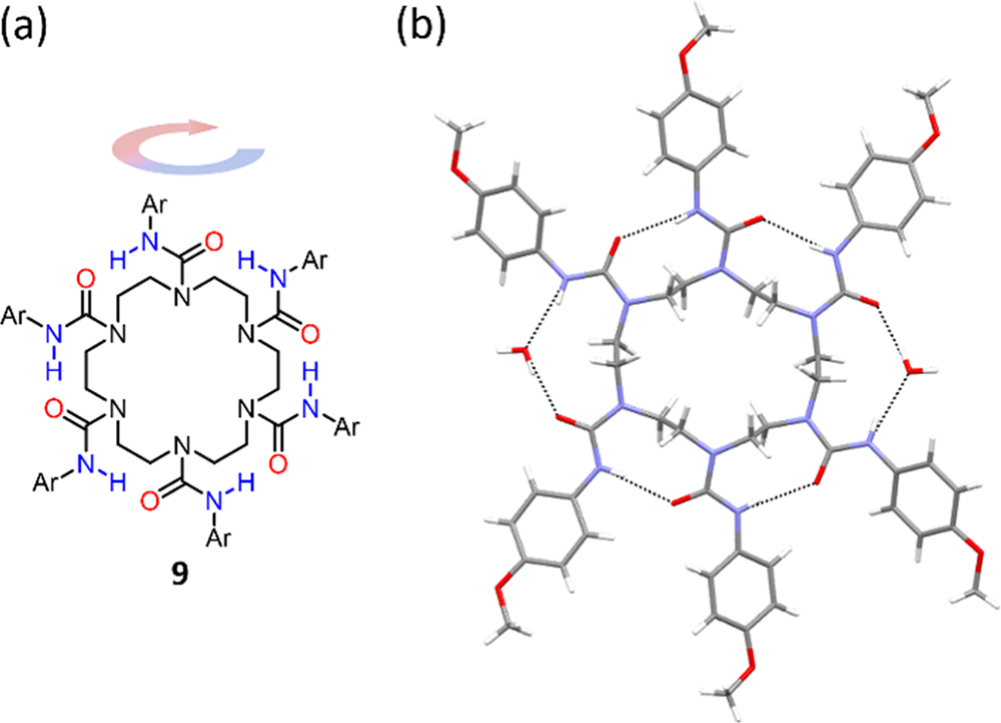
(a) Structure of hexacyclen hexaurea derivative **9**. (b) X-ray crystal structure of **9** (CCDC: 2262697). Shown are two molecules of water, which bridge the intramolecular cyclic hydrogen bond network of **9**. Other solvent molecules in the crystal are omitted for clarity. Ar = 4-OMeC_6_H_4_.

We also sought to determine whether cyclochirality was evident in homologous tetra- or hexaureas. In contrast to the TACN-derived ureas **1**, their larger-ring homologues did not appear to take on chiral ground-state conformations. Cyclen-derived tetraureas showed no diastereotopic signals by ^1^H NMR in CDCl_3_ or CD_2_Cl_2_ at a range of temperatures, while a hexacyclen derivative **9** (Figure 12a) crystallized in an achiral, “unfolded” conformation with an inversion center (Figure 12b)—reminiscent of the reported crystal structure of cyclen bearing four butyl ureas.^43^ These crystal structures (Figure 12b, ref (43)) reveal that the ethylene bridges of these more flexible, higher homologues adopt partially or completely the *anti* conformation,^23^ which leads to unfolding. Despite the unfolded structure of these cyclen and hexacyclen derivatives, coherent and contiguous hydrogen bonding between ureas (bridged in part by solvent molecules) is evident in the solid state,^25^ which may form the basis for further studies on induced hydrogen bond directionality in more complex cyclochiral systems.

## Conclusions

In summary, various cyclochiral tri(thio)ureas and their derivatives have been synthesized and their two enantiomerization mechanisms have been investigated in the solution and solid states by VT NMR, ^1^H-^1^H EXSY, Eyring analysis, computational modeling, and X-ray crystallography. This mechanistic understanding allowed structural modifications that exploited symmetry to control the conformational dynamics of the system by selectively preventing either or both inversion mechanisms from operating. The control exerted by these modifications allows selection of the dynamic process by which the cyclochiral structure racemizes—the structure can retain a persistent bowl-like structure that can reverse its hydrogen-bond directionality, or it can exhibit a dynamic bowl-like structure that retains coherent and unidirectional hydrogen-bond directionality. Employing both modifications substantially raised the enaniomerization barrier, introducing persistent cyclochirality to the resulting structure. Future modifications may allow the application of the stable cyclochiral structures in asymmetric catalysis or in enantioselective host–guest binding.

## Supporting Information Available

Synthetic schemes, experimental procedures and compound characterization data, details of conformational analysis, VT NMR spectra, Eyring plots, HPLC traces, CD spectra and decay plots, computational methods, crystallography data, and NMR spectra of novel compounds (PDF)
