# Supplementary material for: Dynamic and Persistent Cyclochirality in Hydrogen-Bonded Derivatives of Medium-Ring Triamines
Source: J Am Chem Soc. 2023 Aug 18;145(34):19030–41. doi: 10.1021/jacs.3c06570 (PMC10472504; doi:10.1021/jacs.3c06570)
Supplement: Supplementary file 1 — ja3c06570_si_001.pdf [file ja3c06570_si_001.pdf]

Dynamic and Persistent Cyclochirality in Hydrogen-Bonded Derivatives of Medium-Ring Triamines

David T. J. Morris,[‡] Steven M. Wales,[‡] Javier Echavarren, Matej Žabka, Giulia Marsico, John W. Ward, Natalie E. Pridmore and Jonathan Clayden*

Email: j.clayden@bristol.ac.uk

[‡]These authors contributed equally.

Contents

General Information 2

Synthetic Schemes 3

Experimental Procedures 7

Variable-Temperature ^{1}H NMR Studies of **1–3**, **5–7** 23

Eyring Analyses of **1–3**, **5–7** 38

NMR Studies of **1d** in $CDCl_3$ 55

EXSY Studies of **1d** in TCE-d_2 57

NMR Solvent Studies of **1d** 63

NMR H/D Exchange Kinetics of **1d** 64

NMR Studies of **3** 66

NMR Studies of **4** 72

NMR Studies of **8** 79

Stereochemical Stability of **8** using Circular Dichroism (CD) Spectroscopy 85

Contrasting Conformations of **1a** and **9** 92

Computational Details 94

- Computed Energies of **3** / **3'** 94
- Computed Geometric Properties of the H-Bonds in **4** / **4'** 95
- Computed Energies of **4** / **4'** and UV-Vis/CD Spectra of (*S*,*S*)-**4** 96
- HOMO/LUMO Visualizations for **4** 100
- Computed Energies of **8** / **8'** 103
- Coordinates of Optimized Structures 104

Crystallography 132

NMR Spectra of Novel Compounds 137

References 161

General Information

Where specified, procedures were performed under an atmosphere of nitrogen. Air and moisture-sensitive liquids/solutions were transferred to reaction vessels by syringe under an atmosphere of nitrogen. Solvents and reagents were purchased from commercial suppliers and were used without further purification unless otherwise specified. Agitation was achieved using Teflon coated stirrer bars by magnetic induction. All thin layer chromatography (TLC) experiments were conducted on pre-coated plastic plates (Macherey-Nagel polygram SIL G/UV_{254}) and visualized using ultraviolet light (254 nm) or staining. Flash chromatography was performed on an automated Biotage Isolera™ Spektra Four using gradient elution on pre-packed silica gel Sfär Duo columns. Solvent systems for TLC and flash chromatography are reported in solvent:solvent volume ratios. All variable-temperature NMR experiments were conducted using a Bruker AVANCE III HD 500 MHz NMR Spectrometer with 5 mm DCH ^{13}C–^{1}H/D Cryo Probe (500 MHz) or a Varian 500 MHz Spectrometer. All room temperature NMR experiments were conducted using a JEOL ECZ 400 MHz Spectrometer, a Bruker Nano 400 MHz Spectrometer or a Bruker AVANCE III HD 500 MHz NMR Spectrometer with a 5 mm DCH ^{13}C–^{1}H/D Cryo Probe, with chemical shifts reported (δ in ppm) relative to the specified deuterated solvent. All NMR characterization experiments were performed at 25 °C and 1 atm unless otherwise specified. Multiplicity is reported as follows – s = singlet, d = doublet, t = triplet, q = quartet, m = multiplet. All spin-spin coupling constants (J) are reported in hertz (Hz) to the nearest 0.1 Hz. All ^{13}C NMR signals are reported as singlets unless otherwise stated. High-resolution mass spectrometry experiments (HR-MS) were performed on a Bruker micrOTOF Spectrometer using electrospray ionization, positive ion mode or a Bruker Ultraflex using MALDI with only molecular ion ($[M+H]^+$ or $[M+Na]^+$) peaks being reported. Chiral HPLC was performed with a Chiralcel OD-H column on an Agilent 1260 Infinity HPLC system equipped with a fraction collector. Circular dichroism (CD) spectroscopy was performed on a Jasco J-815 CD spectrometer equipped with and a Jasco CDF-426S/15 Peltier module and a Julabo AWC100 cooler. All CD spectra are corrected by subtracting the 'blank' spectrum of the solvent from the spectrum containing the analyte.

Synthetic Schemes

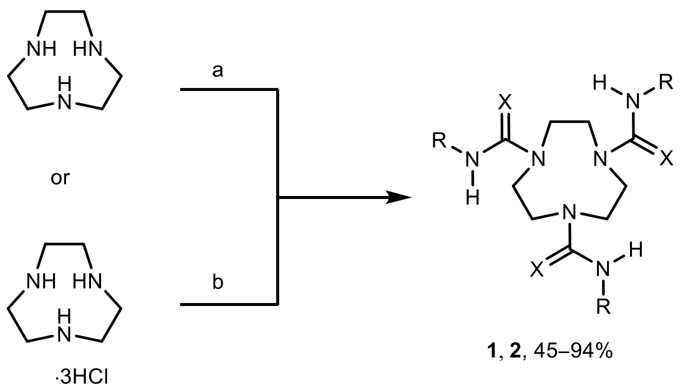

Scheme S1. Synthesis of TACN-derived tri-ureas **1** (X = O) and tri-thioureas **2** (X = S). Reagents and conditions: (a) RNCX (3.0−4.5 equiv), CH_2Cl_2 (or $CHCl_3$), 0 °C−RT, 0.5−16 h; (a) RNCX (3.0−4.5 equiv), *i*-Pr_2EtN (3.0−3.3 equiv), CH_2Cl_2, RT, 16−21.5 h.

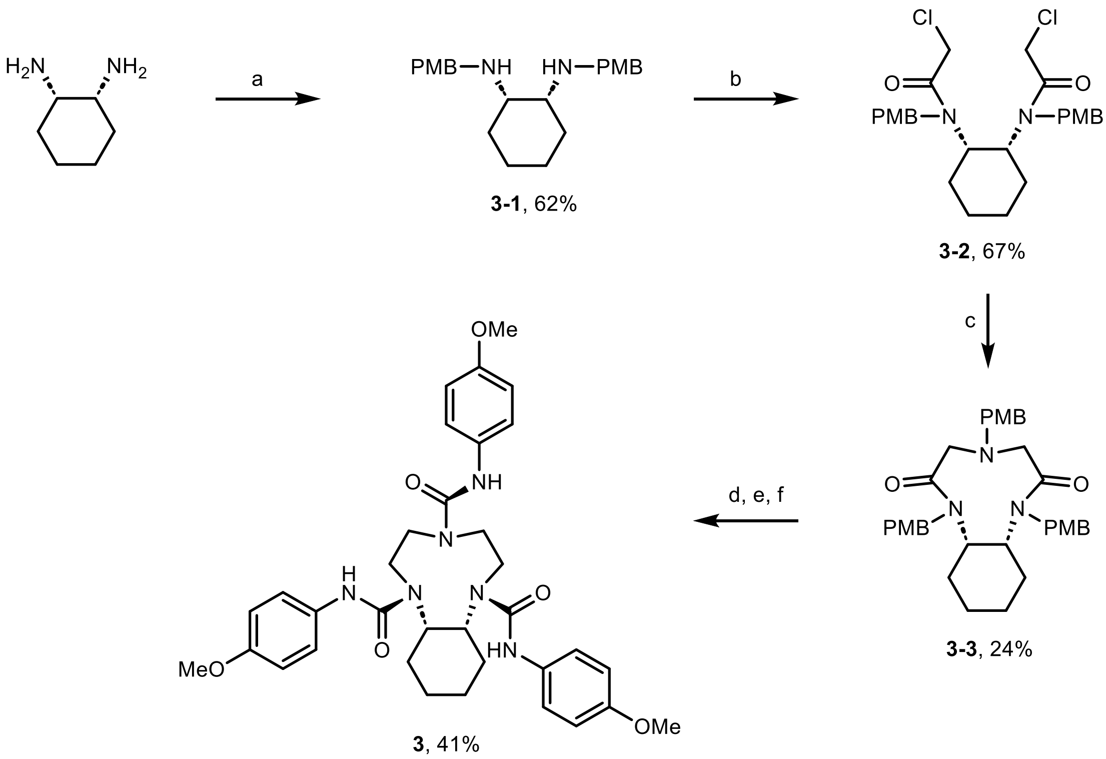

Scheme S2. Synthesis of *cis*-cyclohexane-fused derivative **3**. Reagents and conditions: (a) (i) *p*-anisaldehyde (2.0 equiv), MeOH, 65 °C, 22 h; (ii) $NaBH_4$, RT, 26 h; (b) chloroacetyl chloride (2.2 equiv), K_2CO_3 (5.0 equiv), H_2O, $CHCl_3$, 0−10 °C, 1.5 h; (c) *p*-methoxybenzylamine (1.0 equiv), LiBr (2.1 equiv), Na_2CO_3, MeCN, 80 °C, 25 h; (d) $LiAlH_4$, THF, 70 °C, 16 h; (e) Pd/C, H_2, AcOH, MeOH, 50 °C, 4 h; (f) 4-methoxyphenyl isocyanate (4.5 equiv), *i*-Pr_2EtN, CH_2Cl_2, RT, 16 h. PMB = *p*-methoxybenzyl.

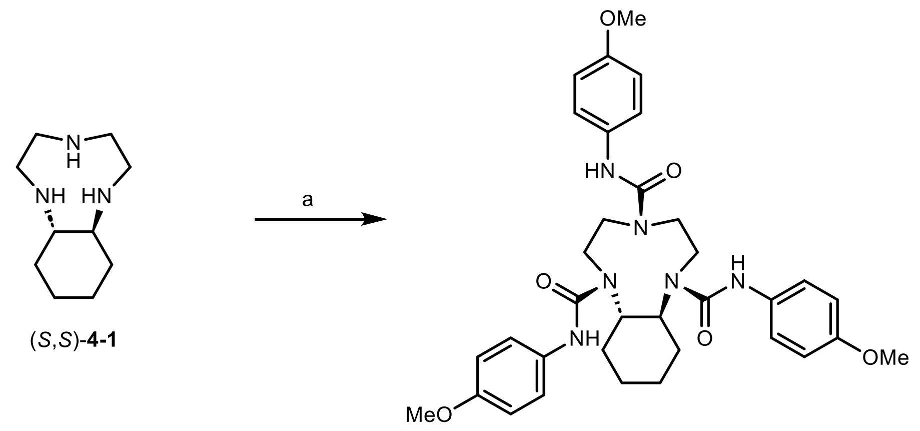

(*S*,*S*)-**4-1**

(*S*,*S*)-**4**, 47%

Scheme S3. Synthesis of *(S,S)-trans*-cyclohexane-fused derivative **4**. Reagents and conditions: (a) 4-methoxyphenyl isocyanate (3.1 equiv), CH_2Cl_2, RT, 5 h.

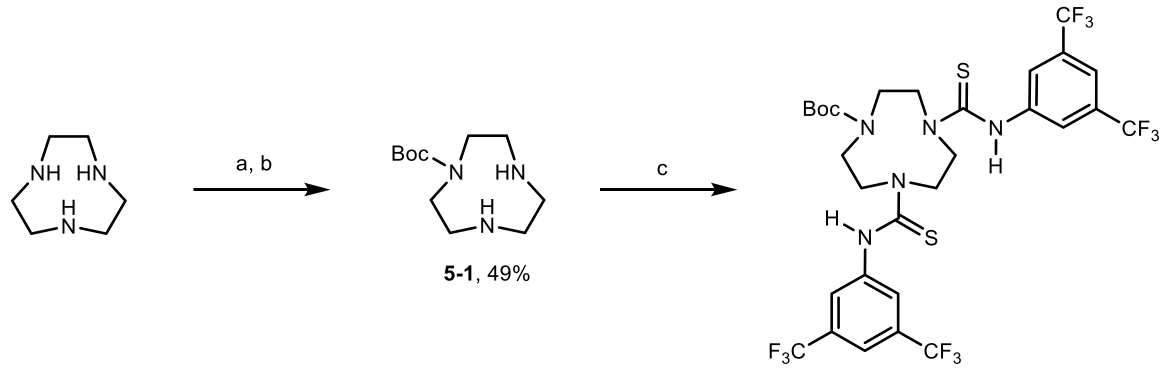

5-1, 49%

6, 89%

Scheme S4. Synthesis of Boc-protected, thiourea **6**. Reagents and conditions: (a) (i) PhCHO (1.0 equiv), $MgSO_4$, $CDCl_3$, 40 °C, 2.5 h; (ii) Boc_2O (1.0 equiv), $CHCl_3$, RT, 2 h; (b) $TsNHNH_2$ (1.0 equiv), MeOH, RT, 2.5 h; (c) 3,5-bis(trifluoromethyl)phenyl isothiocyanate (2.0 equiv), CH_2Cl_2, RT, 2.5 h.

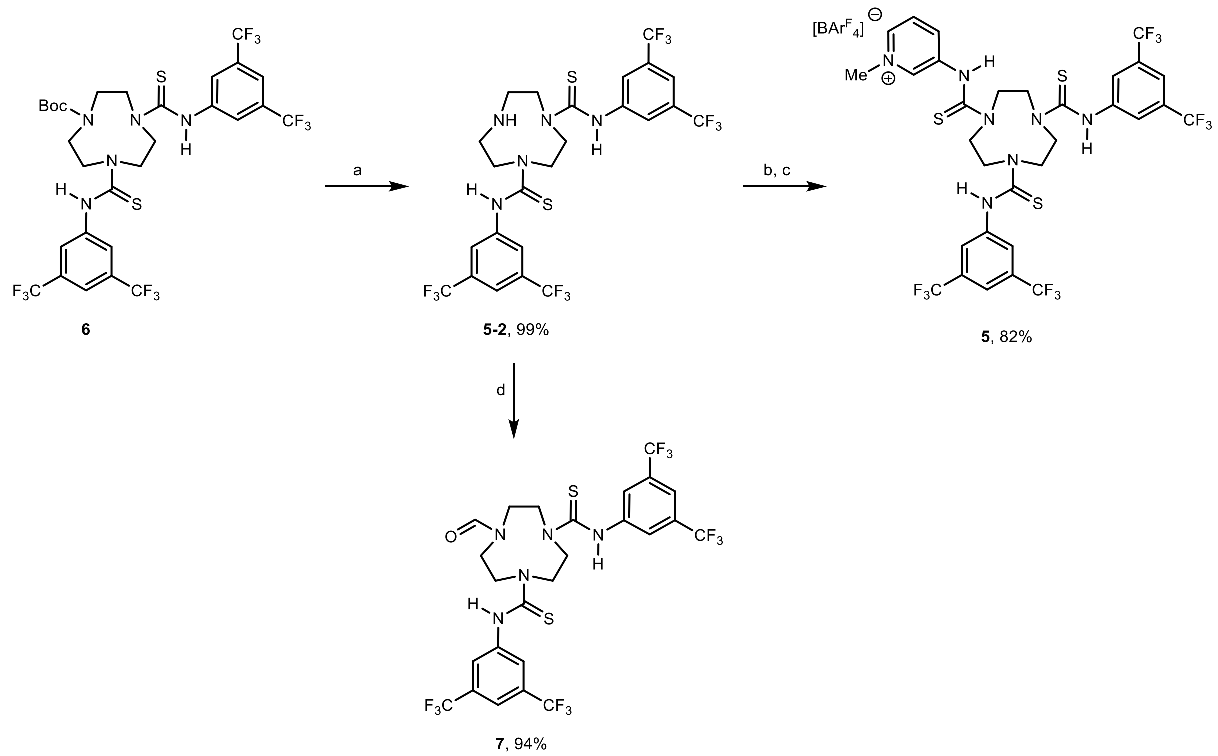

Scheme S5. Synthesis of pyridinium thiourea **5** and formamide **7**. Reagents and conditions: (a) trifluoroacetic acid, CH_2Cl_2, RT, 21.5 h; (b) 3-isothiocyanato-1-methylpyridin-1-ium iodide[S1] (1.0 equiv), MeCN, RT, 20 min; (c) sodium tetrakis[(3,5-bis(trifluoromethyl)phenyl]borate (1.0 equiv), CH_2Cl_2, RT, 2 h; (d) 2,2,2-trifluoroethyl formate, CH_2Cl_2, RT, 19 h. $Ar^F = 3,5\text{-bis}(CF_3)_2C_6H_3$.

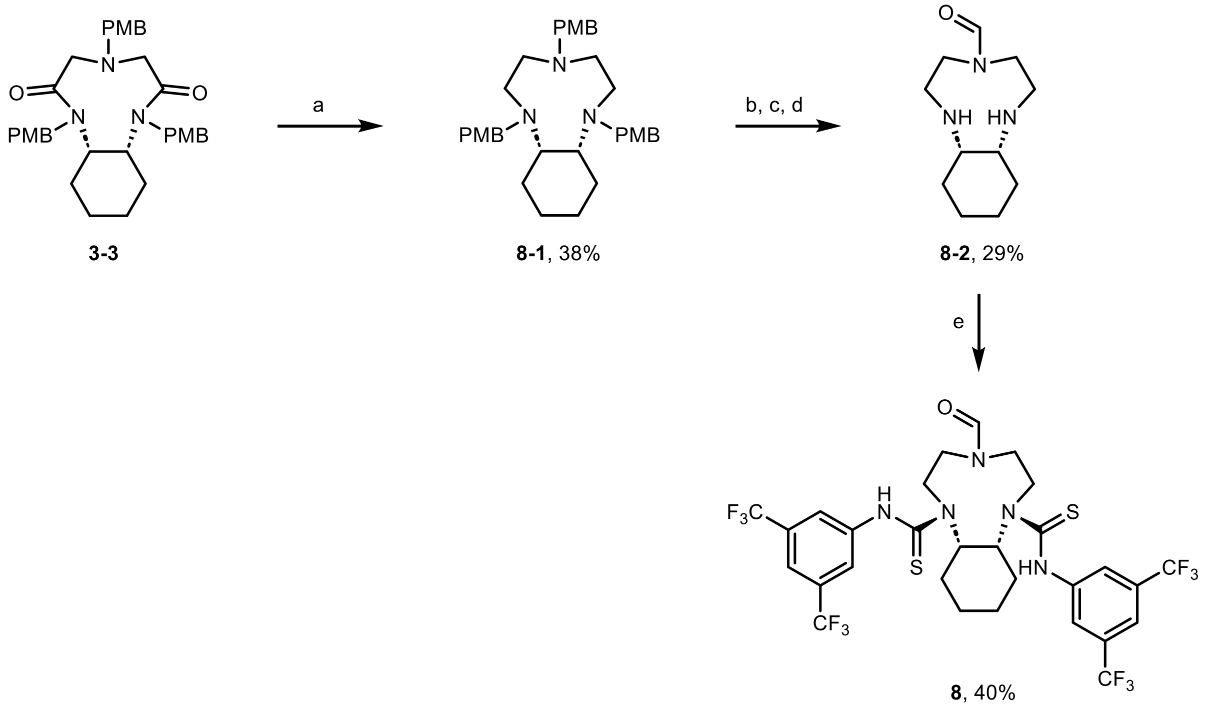

Scheme S6. Synthesis of formamide **8**. Reagents and conditions: (a) $LiAlH_4$, THF, RT, 19.5 h; (b) (i) 1-chloroethyl chloroformate (1.0 equiv), 1,2-dichloroethane, 70 °C, 19.5 h; (ii) MeOH, 60 °C, 1.5 h; (c) 2,2,2-trifluoroethyl formate, CH_2Cl_2, RT, 1.5 h; (d) Pd/C, H_2, AcOH, MeOH, 50 °C, 2 h; (e) 3,5-bis(trifluoromethyl)phenyl isothiocyanate (3.7 equiv), 1,2-dichloroethane, 40−50 °C, 93.5 h. PMB = *p*-methoxybenzyl.

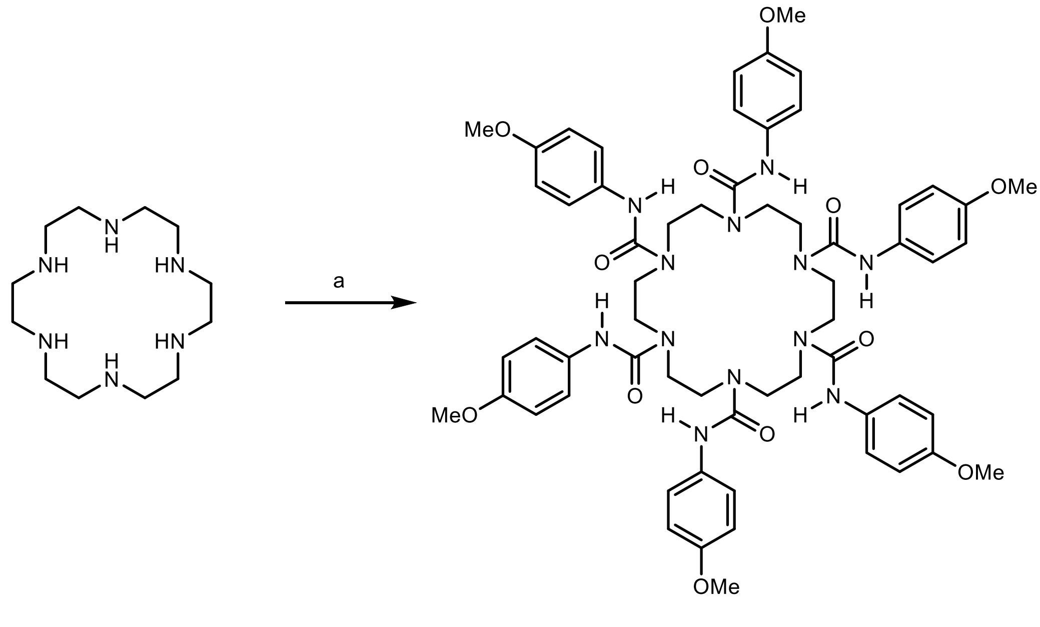

9, 34%

Scheme S7. Synthesis of hexacyclen derivative **9**. Reagents and conditions: (a) 4-methoxyphenyl isocyanate (6.3 equiv), CH_2Cl_2, RT, 40 min.

Experimental Procedures

General Procedure A – (Thio)Carboxamidation of 1,4,7-triazacyclononane (TACN) using iso(thio)cyanates.

Under a dry, inert atmosphere, a solution of TACN (1.0 equiv, 0.10 M) in anhydrous CH_2Cl_2 was cooled to 0 °C. To this was added iso(thio)cyanate (4.5 equiv) dropwise (if a liquid) or over three portions (if a solid). The resulting solution was allowed to warm to room temperature and stirred for 16 h before being concentrated *in vacuo* to give the crude (thio)urea. Purification was performed as described within.

1,4,7-Tris(4-methoxyanilinylcarbonyl)-1,4,7-triazacyclononane, 1a

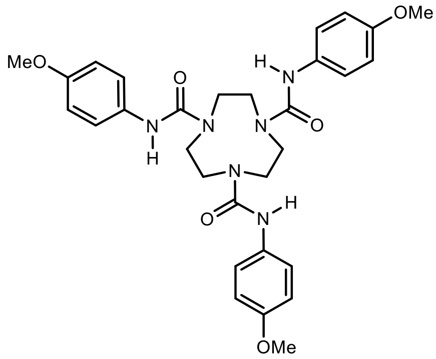

Synthesised according to *General Procedure A* using TACN (20 mg, 0.15 mmol) and 4-methoxyphenyl isocyanate (0.03 mL, 0.70 mmol). The crude product was purified by column chromatography (1-10% MeOH:CH_2Cl_2) to yield the title triurea as a foamy white solid (78 mg, 0.14 mmol, 90%). **TLC** – R_f = 0.17 (SiO_2, 5:95 MeOH:CH_2Cl_2). **^{1}H NMR** (500 MHz, $CDCl_3$) δ_H 2.96-3.16 (m, 6H, 3 x NCH^A, 3 x NCH^B), 3.74 (s, 9H, 3 x OCH_3), 3.93-4.01 (m, 3H, 3 x NCH^A), 4.33-4.45 (m, 3H, 3 x NCH^B), 6.49 (d, J = 9.0, 6H, 6 x Ar*H*), 6.63 (d, J = 9.0, 6H, 6 x Ar*H*), 7.84 (s, 3H, 3 x N*H*). **^{13}C NMR** (126 MHz, $CDCl_3$) δ_C 45.0 (3 x N*C*H_2), 47.0 (3 x N*C*H_2), 55.6 (3 x O*C*H_3), 113.6 (6 x Ar*C*H), 122.7 (6 x Ar*C*H), 132.3 (3 x Ar*C*N), 155.4 (3 x Ar*C*O), 159.0 (3 x *C*O). **HR-MS** (ESI, positive ion mode) – *m/z* for $[C_{30}H_{36}N_6O_6+Na]^+$ = 599.2589. Found 599.2618.

Crystals of **1a** suitable for X-ray diffraction were grown by slow diffusion of Et_2O into a solution of **1a** in $CHCl_3$ (CCDC: 2262692).

1,4,7-Tris(4-chloroanilinylcarbonyl)-1,4,7-triazacyclononane, 1b

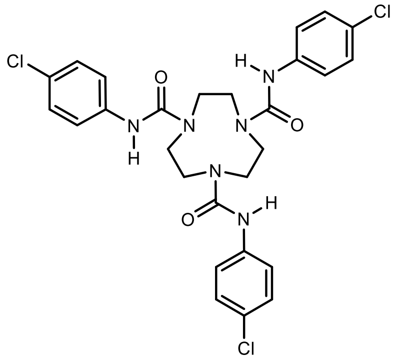

Synthesised according to *General Procedure A* using TACN (50 mg, 0.39 mmol) and 4-chlorophenyl isocyanate (267 mg, 1.74 mmol). The title triurea was isolated as a white solid (267 mg, 0.18 mmol, 45%). **TLC** – R_f = 0.36 (SiO_2, 10:90 MeOH:CH_2Cl_2). **^{1}H NMR** (400 MHz, $CDCl_3$) δ_H 3.02-3.04 (m, 3H, 3 x NCH^A), 3.11-3.15 (m, 3H, 3 x NCH^B), 3.89-3.91 (m, 3H, 3 x NCH^A), 4.34-4.37 (m, 3H, 3 x NCH^B), 6.58 (d, J = 8.9, 6H, 6 x Ar*H*), 6.85 (d, J = 8.9, 6H, 6 x Ar*H*), 7.90 (s, 3H, 3 x N*H*). **^{13}C NMR** (101 MHz, $CDCl_3$)

δ_C 44.9 (3 x N*C*H$_2$), 46.9 (3 x N*C*H$_2$), 122.3 (6 x Ar*C*H), 128.3 (3 x Ar*C*Cl), 128.4 (6 x Ar*C*H), 137.4 (3 x Ar*C*N), 158.7 (3 x *C*O). **HR-MS** (MALDI) – *m/z* for $[C_{27}H_{27}Cl_3N_6O_3+Na]^+$ = 611.1108. Found 611.1102.

1,4,7-Tris(4-(ethylcarboxy)anilinylcarbonyl)-1,4,7-triazacyclononane, 1c

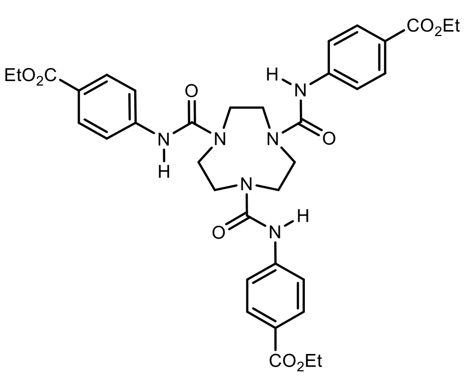

Synthesised according to *General Procedure A* using TACN (50 mg, 0.37 mmol) and ethyl 4-isocyanatobenzoate (333 mg, 1.74 mmol). The residue was washed with cold acetonitrile and air-dried to give the title triurea as a white solid (272 mg, 0.30 mmol, 80%). **TLC** – R_f = 0.35 (SiO_2, 5:95 MeOH:CH_2Cl_2). **^{1}H NMR** (400 MHz, $CDCl_3$) δ_H 1.37 (t, J = 7.1, 9H, 3 x CH$_2$C*H*$_3$), 3.08-3.15 (m, 6H, 3 x NC*H*^{A}, 3 x NC*H*^{B}), 4.00-4.02 (m, 3H, 3 x NC*H*^{A}), 4.32 (q, J = 7.1, 6H, 3 x OC*H*$_2$), 4.44-4.47 (m, 3H, 3 x NC*H*^{B}), 6.91 (d, J = 8.9, 6H, 6 x Ar*H*), 7.61 (d, J = 8.9, 6H, 6 x Ar*H*), 8.19 (s, 3H, 3 x N*H*). **^{13}C NMR** (101 MHz, $CDCl_3$) δ_C 14.4 (3 x *C*H$_3$), 44.9 (3 x N*C*H$_2$), 46.9 (3 x N*C*H$_2$), 60.8 (3 x O*C*H$_2$), 119.5 (6 x Ar*C*H), 124.9 (3 x Ar*C*H), 130.2 (6 x Ar*C*N), 143.0 (3 x Ar*C*C), 158.3 (3 x N*C*O), 166.3 (3 x *C*OO). **HR-MS** (ESI, positive ion mode) – *m/z* for $[C_{36}H_{42}N_6O_9+Na]^+$ = 725.2911. Found 725.2901.

1,4,7-Tris(3,5-bis(trifluoromethyl)anilinylcarbonyl)-1,4,7-triazacyclononane, 1d

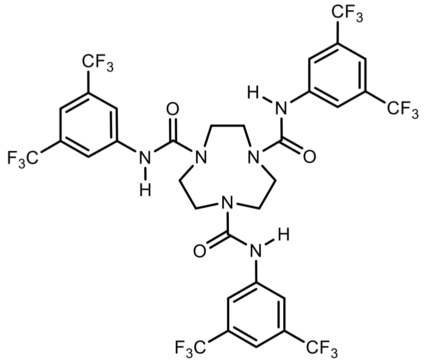

To a solution of TACN (12.9 mg, 0.10 mmol, 1.0 equiv) in lab grade CH_2Cl_2 (0.25 mL) under air was added a solution of 3,5-bis(trifluoromethyl)phenyl isocyanate (76.5 mg, 0.30 mmol, 3.0 equiv) in CH_2Cl_2 (0.75 mL) and the solution was stirred at room temperature for 3 h. MeOH (1 mL) was added and stirring continued for 25 min. The solvent was removed *in vacuo*. Flash chromatography (Biotage, 5 g Sfär Duo column, MeOH/CH_2Cl_2 gradient from 0:100 to 2:98) gave the product (80.0 mg, 89%) as a white solid. **TLC** – R_f = 0.47 (SiO_2, 2.5:97.5 MeOH:CH_2Cl_2). **^{1}H NMR** (500 MHz, $CDCl_3$) δ_H 3.10-3.19 (m, 3H, 3 x NC*H*^{A}), 3.21-3.27 (m, 3H, 3 x NC*H*^{B}), 4.04-4.11 (m, 3H, 3 x NC*H*^{A}), 4.43-4.53 (m, 3H, 3 x NC*H*^{B}), 7.30 (d, J = 1.5, 6H, 6 x Ar*H*), 7.42 (t, J = 1.5, 3H, 3 x Ar*H*), 8.45 (s, 3H, 3 x N*H*). **^{13}C NMR** (126 MHz, $CDCl_3$) δ_C 44.9 (3 x N*C*H$_2$), 47.0 (3 x N*C*H$_2$), 116.9 (3 x Ar*C*H), 119.4 (6 x Ar*C*H), 122.9 (q, J = 271.8, 6 x *C*F$_3$), 132.3 (q, J = 34.1, 6 x Ar*C*CF$_3$), 139.9 (3 x Ar*C*N), 158.3 (3 x *C*O). **^{19}F NMR** (376 MHz, $CDCl_3$) δ_F −63.5. **HR-MS** (ESI, positive ion mode) – *m/z* for $[C_{33}H_{24}F_{18}N_6O_3+H]^+$ = 895.1695. Found 895.1657.

1,4,7-Tris(3,5-dimethylanilinylcarbonyl)-1,4,7-triazacyclononane, 1e

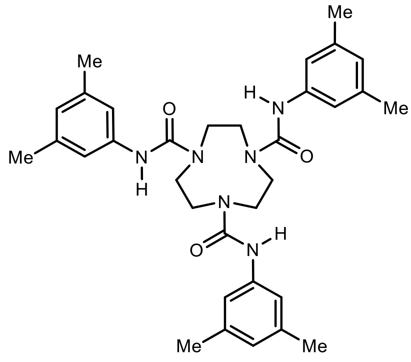

To a solution of TACN (12.9 mg, 0.10 mmol, 1.0 equiv) in lab grade CH_2Cl_2 (0.25 mL) under air was added a solution of 3,5-dimethylphenyl isocyanate (44.2 mg, 0.30 mmol, 3.0 equiv) in CH_2Cl_2 (0.75 mL) and the solution was stirred at room temperature for 3 h. MeOH (1 mL) was added and stirring continued for 35 min. The solvent was removed *in vacuo*. Flash chromatography (Biotage, 5 g Sfär Duo column, MeOH/CH_2Cl_2 gradient from 0:100 to 2:98) gave the product (53.9 mg, 94%) as a white solid. **TLC** – R_f = 0.33 (SiO_2, 2.5:97.5 MeOH:CH_2Cl_2). **^{1}H NMR** (500 MHz, $CDCl_3$) δ_H 1.98 (s, 18H), 2.90 – 3.25 (m, 6H), 3.87 – 4.08 (m, 3H), 4.32 – 4.56 (m, 3H), 6.47 (s, 6H), 6.54 (s, 3H), 7.97 (s, 3H). **^{13}C NMR** (126 MHz, $CDCl_3$) δ_C 21.1, 44.8, 47.0, 118.9, 124.9, 137.8, 138.8, 158.8. **HR-MS** (ESI, positive ion mode) – *m/z* for $[C_{33}H_{42}N_6O_3+H]^+$ = 571.3391. Found 571.3413.

1,4,7-Tris(4-pyridylaminocarbonyl)-1,4,7-triazacyclononane, 1f

This compound was prepared using the corresponding phenyl carbamate as the acylating agent instead of an isocyanate. Preparation of phenyl N-(4-pyridyl)carbamate: Under a dry, inert atmosphere, a solution of 4-aminopyridine (1.00 g, 10.6 mmol, 1.0 equiv, 0.1 M) in anhydrous CH_2Cl_2 (60 mL) was cooled to 0 °C. To this solution was added phenyl chloroformate (1.47 mL, 11.7 mmol, 1.1 equiv) dropwise followed by anhydrous Et_3N (2.07 mL, 14.9 mmol, 1.4 equiv) in one portion. The resulting colourless solution was warmed to room temperature and stirred for 2 h, after which time saturated aqueous $NaHCO_3$ (60 mL) was added slowly. The aqueous phase was extracted with CH_2Cl_2 (3 x 50 mL) and the combined organic extracts were dried ($MgSO_4$), filtered and concentrated *in vacuo* to yield phenyl *N*-(4-pyridyl)carbamate (1.30 g, 57%) as a white solid. **TLC** – R_f = 0.25 (SiO_2, 1:99 MeOH:CH_2Cl_2). **^{1}H NMR** (400 MHz, $CDCl_3$) δ_H 7.19 (d, J = 8.1, 2H, 2 x Ar*H*), 7.26-7.29 (m, 1H, Ar*H*), 7.39-7.44 (m, 4H, 4 x Ar*H*), 7.99 (s, 1H, N*H*), 8.51 (d, J = 8.1, 2H, 2 x Ar*H*). Spectroscopic data matched that previously reported.[S2]

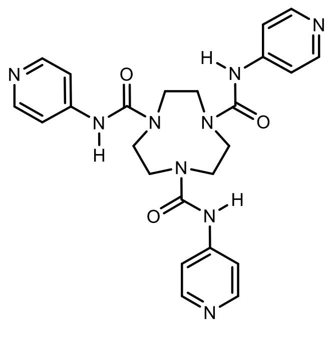

Synthesised according to *General Procedure A* using TACN (50 mg, 0.39 mmol) and phenyl *N*-(4-pyridyl)carbamate (373 mg, 1.74 mmol). The title triurea was isolated as a white solid (132 mg, 0.27 mmol, 70%). **TLC** – R_f = 0.08 (SiO_2, 10:90:1 MeOH:DCM:NH_4OH). **^{1}H NMR** (400 MHz, $CDCl_3$) δ_H 3.09 (brs, 3H), 3.19 (brs, 3H), 4.03 (brs, 3H), 4.44 (brs, 3H), 6.83 (d, J = 4.8, 6H), 8.16 (d, J = 4.8, 6H), 8.18 (bs, 3H). **^{13}C NMR** (101 MHz, $CDCl_3$) δ_C 44.8, 46.8, 114.2, 145.9, 150.2, 158.0. **HR-MS** (ESI, positive ion mode) – *m/z* for $[C_{24}H_{27}N_9O_3+Na]^+$ = 512.2135. Found 512.2129.

1,4,7-Tris(butylaminocarbonyl)-1,4,7-triazacyclononane, 1g

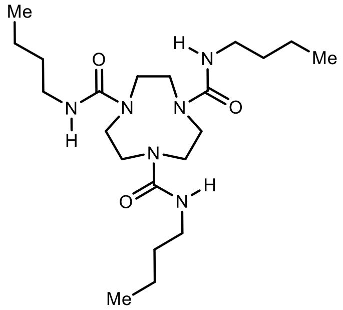

To solid TACN·3HCl (23.9 mg, 0.10 mmol, 1.0 equiv) was added a solution of *N,N*-diisopropylethylamine (42.7 mg, 0.33 mmol, 3.3 equiv) in lab grade CH_2Cl_2 (1 mL) and the suspension was stirred at room temperature under air for 5 min. A solution of butyl isocyanate (29.7 mg, 0.30 mmol, 3.0 equiv) in CH_2Cl_2 (1 mL) was added and the mixture was stirred at room temperature for 21.5 h. The solvent was removed *in vacuo*. Flash chromatography (Biotage, 10 g Sfär Duo column, MeOH/CH_2Cl_2 gradient from 0:100 to 5:95) gave the product (34.4 mg, 81%) as a white solid. **^{1}H NMR** (500 MHz, $CDCl_3$) δ_H 0.91 (t, 9H, 3 x C*H*$_3$), 1.30-1.38 (m, 6H, 3 x C*H*$_2$CH$_3$), 1.43-1.50 (m, 6H, 3 x NCH$_2$C*H*$_2$), 3.10 (q, J = 5.9, 6H, 3 x NC*H*$_2$), 3.41 (s, 12H, 6 x NC*H*$_2$), 5.62 (s, 3H, 3 x N*H*). **^{13}C NMR** (126 MHz, $CDCl_3$) δ_C 14.0 (3 x *C*H$_3$), 20.4 (3 x *C*H$_2$CH$_3$), 32.4 (3 x NCH$_2$*C*H$_2$), 40.7 (3 x N*C*H$_2$), 47.4 (6 x N*C*H$_2$), 160.1 (3 x *C*O). **HR-MS** (ESI, positive ion mode) – *m/z* for $[C_{21}H_{42}N_6O_3+H]^+$ = 427.3397. Found 427.3393.

1,4,7-Tris(benzylaminocarbonyl)-1,4,7-triazacyclononane, 1h

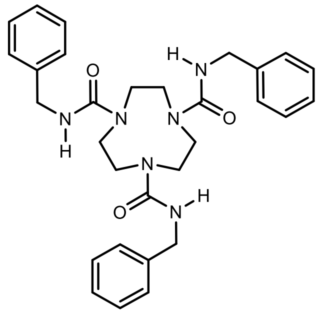

Synthesised according to *General Procedure A* using TACN (20 mg, 0.15 mmol) and benzyl isocyanate (0.08 mL, 0.68 mmol). The crude product was purified by column chromatography (1-10% MeOH:DCM) to yield the title triurea as a foamy white solid (47 mg, 0.09 mmol, 60%). **TLC** – R_f = 0.13 (SiO_2, 1:99 MeOH:DCM). **^{1}H NMR** (400 MHz, $CDCl_3$) δ_H 3.48 (s, 6 x NC*H*$_2$, 12H), 4.20 (d, J = 5.1, 3 x ArC*H*$_2$, 6H), 5.94 (s, 3H, 3 x N*H*), 7.23-7.34 (m, 15 x Ar*H*, 15H). **^{13}C NMR** (101 MHz, $CDCl_3$) δ_C 44.6 (3 x Ar*C*H$_2$), 47.6 (6 x N*C*H$_2$), 127.1 (3 x Ar*C*), 127.6 (6 x Ar*C*), 128.5 (6 x Ar*C*), 139.5 (3 x Ar*C*), 159.6 (3 x *C*O). **HR-MS** (ESI, positive ion mode) – *m/z* for $[C_{30}H_{36}N_6O_3+Na]^+$ = 551.2747. Found 551.2745.

1,4,7-Tris(4-methoxyanilinylthiocarbonyl)-1,4,7-triazacyclononane, 2a

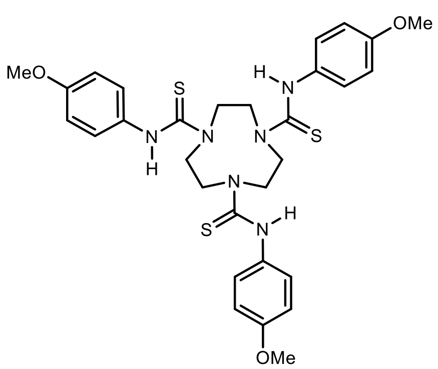

To a solution of TACN (25.8 mg, 0.20 mmol, 1.0 equiv) in lab grade $CHCl_3$ (0.5 mL) under air was added a solution of 4-methoxyphenyl isothiocyanate (99.1 mg, 0.60 mmol, 3.0 equiv) in $CHCl_3$ (1.5 mL) and the solution was stirred at room temperature for 1.5 h. EtOH (0.5 mL) was added and stirring continued for 45 min. The solvent was removed *in vacuo*. Flash chromatography (Biotage, 10 g Sfär Duo column, MeOH/CH_2Cl_2 gradient from 0:100 to 2:98) gave the product (80.4 mg, 64%) as a white solid. **TLC** – R_f = 0.50 (SiO_2, 2.5:97.5 MeOH:CH_2Cl_2). **^{1}H NMR** (500 MHz, $CDCl_3$) δ_H 3.23-3.65 (m, 6H, 3 x NC*H*^{A}, 3 x NC*H*^{B}), 3.79 (s, 9H, 3 x OC*H*$_3$), 4.67-4.89 (m, 3H, 3 x

NCH^A), 5.26-5.46 (m, 3H, 3 x NCH^B), 6.75 (d, J = 9.0, 6H, 6 x Ar*H*), 7.14 (d, J = 9.0, 6H, 6 x Ar*H*), 9.57 (s, 3H, 3 x N*H*). **^{13}C NMR** (126 MHz, $CDCl_3$) δ_C 49.0 (3 x NCH_2), 52.5 (3 x NCH_2), 55.6 (3 x OCH_3), 113.7 (6 x Ar*C*H), 127.0 (6 x Ar*C*H), 132.2 (3 x Ar*C*N), 157.7 (3 x Ar*C*O), 184.2 (3 x *C*S). **HR-MS** (ESI, positive ion mode) – *m/z* for $[C_{30}H_{36}N_6O_3S_3+Na]^+$ = 647.1903. Found 647.1896.

Crystals of **2a** suitable for X-ray analysis were obtained by slow diffusion of petroleum ether into a solution of **2a** in 1,2-dichloroethane (CCDC: 2262693).

1,4,7-Tris(3,5-bis(trifluoromethyl)anilinylthiocarbonyl)-1,4,7-triazacyclononane, 2b

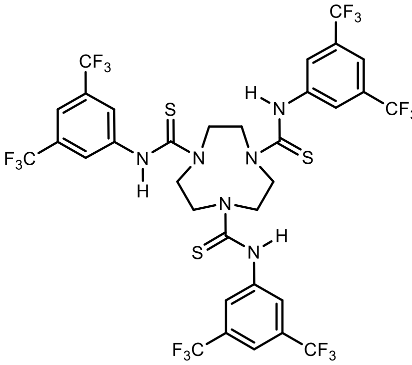

To a suspension of TACN·3HCl (23.9 mg, 0.10 mmol, 1.0 equiv) in lab grade CH_2Cl_2 (1 mL) under N_2 at room temperature was added sequentially *N,N*-diisopropylethylamine (52 µL, 0.30 mmol, 3.0 equiv) and 3,5-bis(trifluoromethyl)phenyl isothiocyanate (82 µL, 0.45 mmol, 4.5 equiv) and the mixture was stirred at room temperature for 16 h. MeOH (0.5 mL) was added and then the solvents were removed *in vacuo*. The residue was re-dissolved in CH_2Cl_2 and the solution was washed sequentially with saturated NH_4Cl, water and brine. The organic layer was dried (Na_2SO_4) and concentrated *in vacuo*. Flash chromatography (Biotage, Sfär Duo column, CH_2Cl_2/petroleum ether gradient from 50:50 to 80:20) gave the product (78 mg, 83%) as a white solid. **TLC** – R_f = 0.75 (SiO_2, 1.5:98.5 MeOH:CH_2Cl_2). **^{1}H NMR** (500 MHz, $CDCl_3$) δ_H 3.44-3.51 (m, 3H, 3 x NCH^A), 3.58-3.66 (m, 3H, 3 x NCH^B), 4.78-4.86 (m, 3H, 3 x NCH^B), 5.34-5.45 (m, 3H, 3 x NCH^A), 7.59 (s, 3H, 3 x Ar*H*), 7.78 (s, 6H, 6 x Ar*H*), 10.01 (s, 3H, 3 x N*H*). **^{13}C NMR** (126 MHz, $CDCl_3$) δ_C 48.9 (3 x NCH_2), 52.4 (3 x NCH_2), 119.2 (3 x Ar*C*H), 122.8 (q, J = 272.3, 6 x CF_3), 123.6 (6 x Ar*C*H), 132.0 (q, J = 33.8, 6 x Ar*C*CF_3), 140.0 (3 x Ar*C*N), 183.5 (3 x *C*S). **^{19}F NMR** (376 MHz, $CDCl_3$) δ_F −63.5. **HR-MS** (Nanospray, positive ion mode) – *m/z* for $[C_{33}H_{24}F_{18}N_6S_3+H]^+$ = 943.1015. Found 943.1013.

Crystals of **2b** (Figure S67) suitable for X-ray analysis were obtained by slow diffusion of petroleum ether into a solution of **2b** in 1,2-dichloroethane (CCDC: 2262694).

(1*R*,2*S*)-*N*,*N′*-Bis(4-methoxybenzyl)cyclohexane-1,2-diamine, 3-1

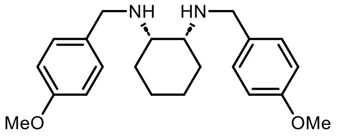

To a solution of *cis*-1,2-diaminocyclohexane (1.142 g, 10.00 mmol, 1.0 equiv) in lab grade MeOH (10 mL) was added *p*-anisaldehyde (2.43 mL, 20.00 mmol, 2.0 equiv) and the mixture was heated to 65 °C under air for 22 h (round bottom flask with glass stopper). After cooling to room temperature, solid $NaBH_4$ (908.0 mg, 24.00 mmol, 2.4 equiv) was added portionwise over 75 min with the flask open to the air (H_2 evolution!), then stirring was continued at room temperature for a further

26 h. MeOH (30 mL) and 10% w/w NaOH (25 mL) were added and the mixture was stirred for 45 min at room temperature. Most of the MeOH was removed by rotary evaporation, then water (20 mL) was added. The product was extracted with CH_2Cl_2 (50 mL, then 25 mL), and the combined organic extracts were dried (Na_2SO_4) and concentrated. Flash chromatography (Biotage, 50 g Sfär Duo column, $MeOH/CH_2Cl_2$ gradient from 0:100 to 10:90) gave the product along with minor, higher R_f impurities. To purify the (neat) product further, 1 M HCl (50 mL) and water (25 mL) were added and the resulting aqueous solution (containing the product as the dihydrochloride salt) was washed with CH_2Cl_2 (40 mL, then 25 mL). The aqueous phase was cooled in an ice bath with stirring and made basic by the slow addition of KOH pellets (4.2 g). The product freebase was extracted with CH_2Cl_2 (50 mL, then 25 mL) and the combined extracts were dried (Na_2SO_4) and concentrated to give the pure product (1.993 g) as a light brown/orange oil. Additional product was obtained by back-extracting the combined CH_2Cl_2 washings (that is, the CH_2Cl_2 used to wash the acidic solution of the dihydrochloride salt) with water (75 mL), basifying the aqueous phase with 10% w/w NaOH (20 mL), then extracting the generated product freebase with CH_2Cl_2 (30 mL). This CH_2Cl_2 extract was dried (Na_2SO_4) and combined with the original sample of pure product. Concentration *in vacuo* gave the pure product (2.208 g, 62%) as a light brown/orange oil. **TLC** – R_f = 0.34 (SiO_2, 5:1:94 MeOH/[35% aqueous NH_3]/CH_2Cl_2). **^{1}H NMR** (400 MHz, $CDCl_3$) δ_H 1.25 – 1.38 (m, 4H), 1.52 – 1.81 (m, 6H), 2.69 – 2.77 (m, 2H), 3.54 (d, J = 13.0 Hz, 2H), 3.67 (d, J = 13.0 Hz, 2H), 3.80 (s, 6H), 6.85 (d, J = 8.3 Hz, 4H), 7.22 (d, J = 8.3 Hz, 4H). **^{13}C NMR** (101 MHz, $CDCl_3$) δ_C 22.5, 28.0, 50.6, 55.4, 55.8, 113.8, 129.3, 133.5, 158.6. **HR-MS** (ESI, positive ion mode) – *m/z* for $[C_{22}H_{30}N_2O_2+H]^+$ = 355.2380. Found 355.2383.

***N,N′*-((1*R*,2*S*)-Cyclohexane-1,2-diyl)bis(2-chloro-N-(4-methoxybenzyl)acetamide), 3-2**

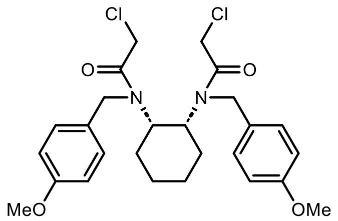

To a solution of **3-1** (3.086 g, 8.71 mmol, 1.0 equiv) in lab grade $CHCl_3$ (23 mL) at 0 °C under air was added dropwise over 6 min a solution of chloroacetyl chloride (1.53 mL, 19.15 mmol, 2.2 equiv) in $CHCl_3$ (17.5 mL) and a solution of K_2CO_3 (6.016 g, 43.53 mmol, 5.0 equiv) in water (17.5 mL) (the two solutions were added simultaneously and at approximately equal rates). After complete addition, the mixture was stirred in the ice bath for 1.5 h, during which time the temperature of the bath increased to 10 °C. The mixture was diluted with CH_2Cl_2 (50 mL) and water (30 mL) and the organic layer was dried (Na_2SO_4) and concentrated. The residue was taken up in CH_2Cl_2 (5 mL) and then HCl (2 M in Et_2O, 2.0 mL, 4.0 mmol) was added (to protonate a cyclisation side product resulting from reaction of **3-1** with 1 equiv of chloroacetyl chloride, thus enabling **3-2** to be separated by chromatography). The mixture was concentrated *in vacuo*. Flash chromatography (Biotage, 50 g Sfär Duo column, Et_2O/petroleum ether gradient from 20:80 to 100:0) gave the product (2.963 g, 67%) as an off-white solid. Note that a mixture of amide rotamers was

observed by NMR at room temperature. The NMR data listed here corresponds to the spectra recorded at 110 °C where the rotamers are in fast exchanage and peak splitting does not occur. Both the 25 °C and 110 °C spectra are provided in the 'NMR Spectra of Novel Compounds' section. **TLC** – R_f = 0.25 (SiO_2, 75:25 Et_2O:petroleum ether). **^{1}H NMR** (500 MHz, $C_2D_2Cl_4$, 110 °C) δ_H 1.38 – 1.49 (m, 2H), 1.67 – 1.84 (m, 4H), 2.18 – 2.33 (m, 2H), 3.86 (s, 6H), 4.03 (d, *J* = 12.6 Hz, 2H), 4.13 (d, *J* = 12.6 Hz, 2H), 4.52 (bs, 2H), 4.71 (bs, 4H), 6.96 (d, *J* = 8.7 Hz, 4H), 7.16 (d, *J* = 8.7 Hz, 4H). **^{13}C NMR** (126 MHz, $C_2D_2Cl_4$, 110 °C) δ_C 22.5, 26.4, 42.2, 50.2, 55.3, 56.0, 114.6, 126.9, 129.5, 159.1, 168.0. **HR-MS** (ESI, positive ion mode) – *m/z* for $[C_{26}H_{32}(^{35}Cl)_2N_2O_4+H]^+$ = 507.1812. Found 507.1813.

(7a*R*,11a*S*)-1,4,7-Tris(4-methoxybenzyl)decahydro-2*H*-benzo[*b*][1,4,7]triazonine-2,6(3*H*)-dione, 3-3

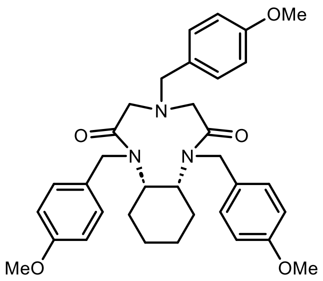

A 500 mL round bottom flask equipped with a reflux condenser was charged with **3-2** (2.952 g, 5.82 mmol, 1.0 equiv) and the system was purged wth N_2. Lab grade MeCN (218 mL) was added, followed sequentially by solid LiBr (1.061 g, 12.22 mmol, 2.1 equiv) and Na_2CO_3 (6.166 g, 58.17 mmol, 10 equiv). The system was again purged wth N_2, then heated to 80 °C with stirring. When the temperature of the heating block had reached 80 °C (after ca 45 min), a solution of 4-methoxybenzylamine (798.0 mg, 5.82 mmol, 1.0 equiv) in MeCN (15 mL) was added over 1 min, then the mixture was heated at 80 °C for 25 h. After cooling to room temperature, the suspension was gravity filtered and the filter cake and reaction flask were rinsed with further MeCN (50 mL). The filtrate was concentrated *in vacuo*. Flash chromatography (Biotage, 50 g Sfär Duo column, EtOAc/petroleum ether gradient from 40:60 to 90:10) gave the product (2.175 g) contaminated with significant lower R_f side products. Further flash chromatography (Biotage, 100 g Sfär Duo column, EtOAc/petroleum ether gradient from 50:50 to 100:0) partially upgraded the product (1.434 g) to 61% w/w purity as quantified by ^{1}H NMR analysis. Further flash chromatography − this time changing the solvent system − (Biotage, 100 g Sfär Duo column, MeOH/CH_2Cl_2 gradient from 1:99 to 2:98) gave the pure product (809.1 mg, 24%) as a white solid. **TLC** – R_f = 0.45 (SiO_2, 85:15 EtOAc:petroleum ether). **^{1}H NMR** (400 MHz, $CDCl_3$) δ_H 1.29 – 1.44 (m, 2H), 1.49 – 1.61 (m, 2H), 1.72 – 1.83 (m, 2H), 1.83 – 2.00 (m, 2H), 3.29 (d, *J* = 14.8 Hz, 2H), 3.57 (bs, 2H), 3.73 – 3.88 (m, 13H, including singlets from 1×OMe and 2×OMe'), 4.76 – 4.86 (m, 2H), 5.21 (d, *J* = 15.1 Hz, 2H), 6.79 (d, *J* = 8.7 Hz, 4H), 6.88 (d, *J* = 8.6 Hz, 2H), 7.13 (d, *J* = 8.7 Hz, 4H), 7.18 (d, *J* = 8.6 Hz, 2H). **^{13}C NMR** (101 MHz, $CDCl_3$) δ_C 19.0, 24.2, 47.7, 55.3, 55.4, 58.8, 62.1, 62.3, 113.8, 114.2, 128.2, 129.0, 130.7, 131.4, 158.5, 159.5, 170.4. **HR-MS** (ESI, positive ion mode) – *m/z* for $[C_{34}H_{41}N_3O_5+H]^+$ = 572.3119. Found 572.3123.

(7a*R*,11a*S*)-*N*[1],*N*[4],*N*[7]-Tris(4-methoxyphenyl)dodecahydro-1*H*-benzo[*b*][1,4,7]triazonine-1,4,7-tricarboxamide, 3

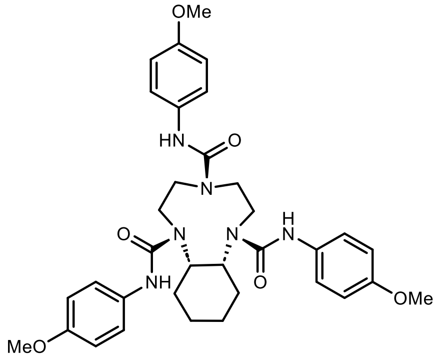

Note: The reduction method desribed in *Step 1* is based on the use of a commerical solution of $LiAlH_4$ in THF. We subsequently found that more consistent and repeatable results were obtained using $LiAlH_4$ powder, although the yield was still modest (see the synthesis and isolation of **8-1** in the preparation of formamide **8**).

***Step 1** (reduction):* To a solution of **3-3** (100 mg, 0.18 mmol) in dry THF (2 mL) under N_2 at room temperature was added a commercial solution of $LiAlH_4$ (1 M in THF, 2.0 mL, 2.00 mmol, 11.1 equiv). The mixture was heated at 70 °C for 16 h. After cooling in an ice bath, water (0.1 mL) was added dropwise (H_2 evolution!). The resulting suspension was filtered and the filter cake and reaction flask were rinsed/washed with THF. The filtrate was concentrated *in vacuo*. The residue was redissolved in $CHCl_3$ and the solution was dried (Na_2SO_4) and again concentrated *in vacuo*. ***Step 2** (hydrogenolysis):* Under N_2, the crude material from *Step 1* (88 mg, 0.15 mmol if pure) was dissolved in dry MeOH (12 mL, pre-degassed by bubbling through N_2) and AcOH (2 drops) was added. Palladium on carbon (10% w/w, 70 mg total, 0.066 mmol of Pd, 0.45 equiv of Pd) was carefully added, then H_2 (from a balloon) was bubbled through the suspension for 15 min, before the mixture was heated at 50 °C for 4 h under H_2. After cooling to room temperature, the H_2 balloon was removed and the vessel was purged with N_2. The suspension was filtered through celite with the aid of additional MeOH. The filtrate was concentrated *in vacuo*. ***Step 3** (urea formation):* The crude material from *Step 2* (0.15 mmol based on full conversion) was dissolved in lab grade CH_2Cl_2 (2 mL) and *N*,*N*-diisopropylethylamine (1 drop) and 4-methoxyphenyl isocyanate (86 µL, 0.67 mmol, 4.5 equiv) were sequentially added. The mixture was stirred under air at room temperature for 16 h. MeOH was added and stirring continued for 2 min. The solvent was removed in *vacuo*. The residue was re-dissolved in CH_2Cl_2 and the solution was washed sequentially with saturated NH_4Cl (2 × 5mL) and brine (5 mL). The organic layer was dried (Na_2SO_4) and concentrated *in vacuo*. Flash chromatography (Biotage, Sfär Duo column, MeOH/CH_2Cl_2 gradient from 0.25:99.75 to 1.5:98.5) gave the product (45 mg, 41% over three steps) as a white solid. **TLC** – R_f = 0.31 (SiO_2, 1:99 MeOH:CH_2Cl_2). **^{1}H NMR** (500 MHz, $CDCl_3$; signals assigned as H_{syn} refer to protons with a syn relationship to the ureas. Signals assigned as H_{anti} refer to protons with an anti relationship to the ureas; signals assigned as C^{cyclo} refer to carbons of the cyclohexane ring) δ_H 1.53-1.58 (m, 1H, $C^{cyclo}H$), 1.61-1.71 (m, 2H, $C^{cyclo}H_2$), 1.96-2.02 (m, 3H, 1 x $C^{cyclo}H_2$, 1 x $C^{cyclo}H$), 2.03-2.12 (m, 2H, 2 x $C^{cyclo}H$), 2.82-2.90 (m, 1H, NCH_{anti}), 3.06 (dd, J = 6.1, 15.2, 1H, NCH_{anti}), 3.36-3.48 (m, 2H, 2 x NCH_{anti}), 3.73 (s, 3H, OCH_3), 3.74 (s, 6H, 2 x OCH_3), 3.80 (dd, J = 5.3, 14.1, 1H, NCH_{syn}), 4.10 (dd, J = 6.2, 14.1, 1H, NCH_{syn}), 4.16-4.24 (m, 1H, NCH_{syn}), 4.45-4.57 (m, 2H, 1 x NCH_{syn}, 1 x $NC^{cyclo}H$), 4.79 (q, J = 7.1, 1H, $NC^{cyclo}H$), 6.46 (d, J = 9.0, 2H, 2 x Ar*H*), 6.48-

6.52 (m, 4H, 4 x Ar*H*), 6.57 (d, J = 9.0, 2H, 2 x Ar*H*), 6.60 (d, J = 9.0, 2H, 2 x Ar*H*), 6.72 (d, J = 9.0, 2H, 2 x Ar*H*), 7.74 (s, 1H, N*H*), 7.85 (s, 1H, N*H*), 8.09 (s, 1H, N*H*). **^{13}C NMR** (126 MHz, $CDCl_3$) δ_C 21.6 (C^{cyclo}), 24.2 (C^{cyclo}), 25.5 (C^{cyclo}), 29.4 (C^{cyclo}), 41.4 (N*C*H_2), 43.3 (N*C*H_2), 48.0 (N*C*H_2), 48.3 (N*C*H_2), 51.5 (NC^{cyclo}), 54.5 (NC^{cyclo}), 55.5 (O*C*H_3), 55.6 (2 x O*C*H_3), 113.5 (2 x Ar*C*H), 113.6 (4 x Ar*C*H), 122.5 (2 x Ar*C*H), 122.7 (2 x Ar*C*H), 122.9 (2 x Ar*C*H), 132.2 (Ar*C*N), 132.5 (Ar*C*N), 132.6 (Ar*C*N), 155.4 (Ar*C*O), 155.4 (Ar*C*O), 155.4 (Ar*C*O), 159.2 (*C*O), 159.3 (*C*O), 159.5 (*C*O). **HR-MS** (ESI, positive ion mode) – *m/z* for $[C_{34}H_{42}N_6O_6+Na]^+$ = 653.3058. Found 653.3039.

Crystals of **3** suitable for X-ray analysis were obtained by slow diffusion of petroleum ether into a solution of **3** in 1,2-dichloroethane (CCDC: 2262695).

(7a*S*,11a*S*)-Dodecahydro-1*H*-benzo[*b*][1,4,7]triazonine, (*S*,*S*)-4-1

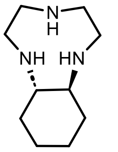

4-1 is a known compound, prepared via the reported reaction of the di-tosyl derivative of (*S*,*S*)-1,2-diaminocyclohexane with the tri-tosyl deriative of diethanolamine to give $(Ts)_3$-(*S*,*S*)-**4-1**,[S3] which was deprotected with sodium amalgam as described.[S4]

(7a*S*,11a*S*)-*N*^{1},*N*^{4},*N*^{7}-Tris(4-methoxyphenyl)dodecahydro-1*H*-benzo[*b*][1,4,7]triazonine-1,4,7-tricarboxamide, (*S*,*S*)-4

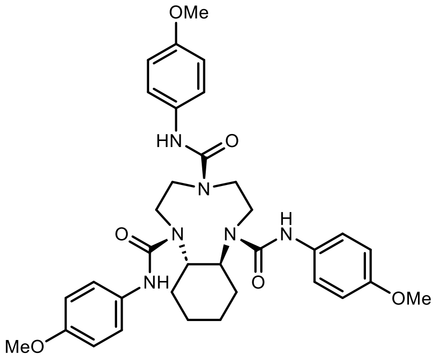

To a solution of (*S*,*S*)-**4-1** (11.0 mg, 0.060 mmol, 1.0 equiv) in lab grade CH_2Cl_2 (0.2 mL) at room temperature under air was added a solution of 4-methoxyphenyl isocyanate (27.9 mg, 0.19 mmol, 3.1 equiv) in CH_2Cl_2 (1 mL) [note that the isocyanate solution was passed through a pipette cotton plug upon addition to the reaction vessel to remove a trace of a solid impurity]. The solution was stirred at room temperature under air for 5 h. The reaction was quenched with MeOH (0.5 mL) and stirred for a further 5 min, before the solvent was removed *in vacuo*. Flash chromatography (Biotage, 5 g Sfär Duo column, MeOH/CH_2Cl_2 gradient from 0:100 to 3:97) gave the product (17.7 mg, 47%) as a white solid. $[\alpha]^{22}_D$ −96.6 (*c* 1.24 g/100 mL, CH_2Cl_2). **TLC** – R_f = 0.34 (SiO_2, 2.5:97.5 MeOH:CH_2Cl_2). **^{1}H NMR** (500 MHz, $CDCl_3$; signals assigned as H_{syn} refer to protons with a syn relationship to the ureas. Signals assigned as H_{anti} refer to protons with an anti relationship to the ureas; signals assigned as C^{cyclo} refer to carbons of the cyclohexane ring) δ_H 1.17-1.27 (m, 1H, C^{cyclo}*H*), 1.41-1.50 (m, 2H, 2 x C^{cyclo}*H*), 1.80 (d, J = 10.8, 1H, C^{cyclo}*H*), 1.89 (d, J = 13.2, 1H, C^{cyclo}*H*), 1.93-2.01 (m, 2H, 2 x C^{cyclo}*H*), 2.54 (dq, J = 4.0, 13.1, 1H, C^{cyclo}*H*), 2.74 (dt, J = 3.7, 11.3, 1H, N$C^{cyclo}$$H_{anti}$), 2.96-3.21 (m, 4H, 4 x NCH_{anti}), 3.65 (dd, J = 5.7, 14.4, 1H, NCH_{syn}), 3.73 (s, 3H, OCH_3), 3.74 (s, 3H, OCH_3), 3.75 (s, 3H, OCH_3), 3.98 (dd, J = 6.0, 14.2, 1H, NCH_{syn}), 4.22 (ddd, J = 6.2, 11.2, 15.8, 1H, NCH_{syn}), 4.45 (ddd, J = 5.7, 11.5, 15.4, 1H,

NCH_{syn}), 4.68-4.75 (m, 1H, $NC^{cyclo}H_{syn}$), 6.46 (d, J = 9.0, 2H, 2 x Ar*H*), 6.48 (d, J = 9.0, 2H, 2 x Ar*H*), 6.49 (d, J = 9.0, 2H, 2 x Ar*H*), 6.58 (d, J = 9.0, 2H, 2 x Ar*H*), 6.62 (d, J = 9.0, 2H, 2 x Ar*H*), 6.63 (d, J = 9.0, 2H, 2 x Ar*H*), 7.79 (s, 1H, N*H*), 7.82 (s, 1H, N*H*), 8.09 (s, 1H, N*H*). **^{13}C NMR** (126 MHz, $CDCl_3$) δ_C 25.4 (C^{cyclo}), 26.2 (C^{cyclo}), 28.7 (C^{cyclo}), 32.3 (C^{cyclo}), 39.5 (NCH_2), 44.6 (NCH_2), 45.5 (NCH_2), 47.4 (NCH_2), 55.5 (2 x OCH_3), 55.6 (OCH_3), 57.3 (NC^{cyclo}), 61.1 (NC^{cyclo}), 113.5 (2 x Ar*C*H), 113.6 (4 x Ar*C*H), 122.6 (2 x Ar*C*H), 122.6 (2 x Ar*C*H), 123.1 (2 x Ar*C*H), 132.2 (Ar*C*N), 132.3 (Ar*C*N), 132.5 (Ar*C*N), 155.3 (Ar*C*O), 155.4 (Ar*C*O), 155.4 (Ar*C*O), 158.7 (*C*O), 158.8 (*C*O), 159.1 (*C*O). **HR-MS** (ESI, positive ion mode) – *m/z* for $[C_{34}H_{42}N_6O_6+Na]^+$ = 653.3058. Found 653.3027.

Crystals of (±)-**4** suitable for X-ray analysis were obtained by slow diffusion of Et_2O into a solution of (±)-**4** in $CHCl_3$ (CCDC: 2262696).

***Tert*-butyl 1,4,7-triazonane-1-carboxylate, 5-1**

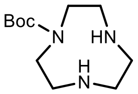

Step 1 *(aminal formation*[S5] *and mono-Boc protection):* Following a literature procedure with some modifications,[S5] to solid TACN (200.0 mg, 1.55 mmol, 1.0 equiv) was added $MgSO_4$ (279 mg) followed by a solution of benzaldehyde (164.3 mg, 1.55 mmol, 1.0 equiv) in $CDCl_3$ (1.5 mL; note that $CDCl_3$ was used so that aliquots could be directly analysed by NMR to monitor conversion) and the suspension was heated at 40 °C under air for 2.5 h. After cooling to room temperature, a solution of Boc_2O (337.8 mg, 1.55 mmol, 1.0 equiv) in $CHCl_3$ (1.5 mL) was added (CO_2 evolution!) and the mixture was stirred at room temperature open to the air for 2h. The suspension was diluted with $CHCl_3$ (20 mL) and filtered by gravity, then the filtrate was concentrated *in vacuo*. ***Step 2*** *(aminal removal by hydrazone formation):* The crude material from *Step 1* (434.8 mg, 88% of theoretical) was dissolved in lab grade MeOH (1.5 mL) and solid *p*-toluenesulfonyl hydrazide (288.7 mg, 1.55 mmol, 1.0 equiv relative to the initial quantity of TACN) was added. The solution was stirred at room temperature under air for 2.5 h, before the solvent was removed *in vacuo*. Flash chromatography (Biotage, 10 g Sfär Duo column, MeOH/[35% aqueous NH_3]/CH_2Cl_2 gradient from 0:0:100 to 10:2:88) gave the product (173.5 mg, 49% over two steps) as a pale yellow oil. The ^{1}H NMR spectum was consistent with the data reported previously.[S6]

***Tert*-butyl 4,7-bis((3,5-bis(trifluoromethyl)phenyl)carbamothioyl)-1,4,7-triazonane-1-carboxylate, 6**

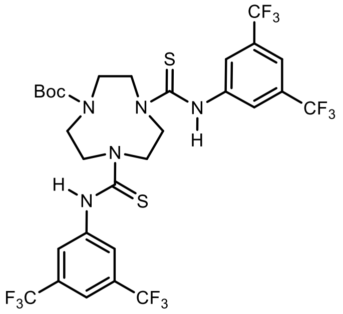

To a solution of **5-1** (69.3 mg, 0.30 mmol, 1.0 equiv) in lab grade CH_2Cl_2 (1 mL) at room temperature under air was added a solution of 3,5-bis(trifluoromethyl)phenyl isothiocyanate (163.9 mg, 0.60 mmol, 2.0 equiv) in CH_2Cl_2 (2 mL) and the solution was stirred for 2.5 h. MeOH (0.5 mL) was added and stirring continued for 5 min, before the solvent was removed *in vacuo*. Flash chromatography (Biotage, 10 g Sfär Duo column, MeOH/CH_2Cl_2 gradient from 0:100 to 2:98) gave the product (207.0 mg, 89%) as a white solid. **TLC** – R_f = 0.31 (SiO_2, 1:99 MeOH:CH_2Cl_2). ^{13}C NMR signals reported as ranges are broad signals due to rotamer exchange. **^{1}H NMR** (500 MHz, $CDCl_3$) δ_H 1.42 (s, 9H, C(C*H*$_3$)$_3$), 3.48-4.48 (m, 12H, 6 x NC*H*$_2$), 7.60 (s, 1H, Ar*H*), 7.63 (s, 1H, Ar*H*), 7.83 (s, 2H, 2 x Ar*H*), 8.12 (s, 2H, 2 x Ar*H*), 8.62 (s, 1H, N*H*), 9.23 (s, 1H, N*H*). **^{13}C NMR** (126 MHz, $CDCl_3$) δ_C 28.4 (C(*C*H$_3$)$_3$), 46.6-48.0 (2 x N*C*H$_2$), 50.8-53.9 (4 x N*C*H$_2$), 82.5 (*C*(CH$_3$)$_3$), 118.2-118.5 (Ar*C*H), 118.6-119.0 (Ar*C*H), 123.0-123.5 (2 x Ar*C*H), 123.1 (q, J = 272.5, 2 x *C*F$_3$), 123.2 (q, J = 272.5, 2 x *C*F$_3$), 123.8 (4 x Ar*C*H), 124.6-125.0 (2 x Ar*C*H), 131.3-132.4 (4 x Ar*C*CF$_3$), 140.7-141.0 (2 x Ar*C*N), 158.2 (*C*O) 183.1-183.4 (2 x C*S*). **^{19}F NMR** (376 MHz, $CDCl_3$) δ_F −63.1, −63.0. **HR-MS** (ESI, positive ion mode) – *m/z* for $[C_{29}H_{29}F_{12}N_5O_2S_2+H]^+$ = 772.1649. Found 772.1644.

***N*^{1},*N*^{4}-Bis(3,5-bis(trifluoromethyl)phenyl)-1,4,7-triazonane-1,4-bis(carbothioamide), 5-2**

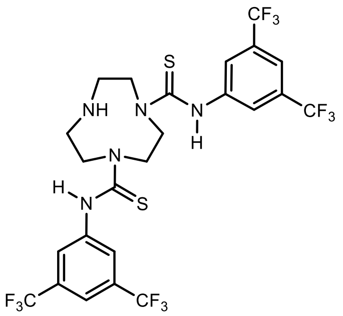

To a solution of **6** (151.9 mg, 0.20 mmol, 1.0 equiv) in lab grade CH_2Cl_2 (2 mL) at room temperature under air was added trifluoroacetic acid (0.23 mL, 2.95 mmol, 15 equiv) and the solution was stirred for 21.5 h. After cooling in an ice bath, saturated $NaHCO_3$ (8 mL) was slowly added over 10 min with stirring. Upon complete addition, the ice bath was removed and stirring continued for 5 min. 10% w/w NaOH was added until pH 9 was reached, then water (10 mL) was added. The product was extracted with CH_2Cl_2 (2 × 20 mL) and the combined organic extracts were dried (Na_2SO_4) and concentrated to give the product (130.7 mg, 99%) as a white solid. **TLC** – R_f = 0.28 (SiO_2, 2.5:97.5 MeOH:CH_2Cl_2). **^{1}H NMR** (500 MHz, $CDCl_3$) δ_H 3.24 (s, 4H, 2 x NC*H*$_2$), 3.82, (s, 4H, 2 x NC*H*$_2$), 4.36 (s, 4H, 2 x NC*H*$_2$), 7.61 (s, 2H, 2 x Ar*H*), 7.84 (s, 4H, 4 x Ar*H*), 8.90 (s, 2H, 2 x N*H*). **^{13}C NMR** (126 MHz, $CDCl_3$) δ_C 47.6 (2 x N*C*H$_2$), 53.3 (2 x N*C*H$_2$), 56.7 (2 x N*C*H$_2$), 118.2 (2 x Ar*C*H), 123.2 (q, J = 272.9, 4 x *C*F$_3$), 123.8 (4 x Ar*C*H), 131.9 (q, J = 33.7, 4 x Ar*C*CF$_3$), 141.1 (2 x Ar*C*N), 183.5 (2 x C*S*). **^{19}F NMR** (376 MHz, $CDCl_3$) δ_F −62.8. **HR-MS** (ESI, positive ion mode) – *m/z* for $[C_{24}H_{21}F_{12}N_5S_2+H]^+$ = 672.1125. Found 672.1118.

3-(4,7-Bis((3,5-bis(trifluoromethyl)phenyl)carbamothioyl)-1,4,7-triazonane-1-carbothioamido)-1-methylpyridin-1-ium tetrakis(3,5-bis(trifluoromethyl)phenyl)borate, 5

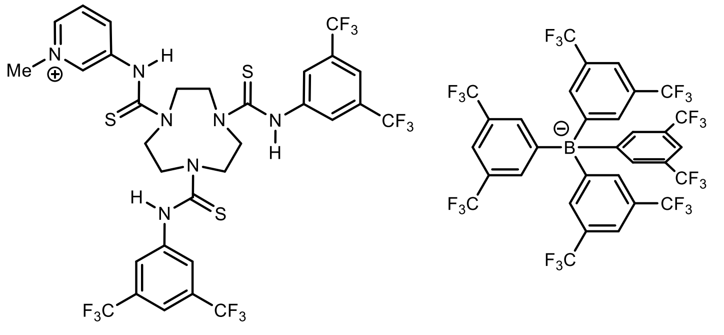

***Step 1** (thiourea formation):* 3-Isothiocyanato-1-methylpyridin-1-ium iodide[S1] (27.1 mg, 0.10 mmol, 1.0 equiv) and **5-2** (65.5 mg, 0.10 mmol, 1.0 equiv) were added to a reaction vial. Dry MeCN (2 mL) was added and the resulting yellow solution was stirred under air at room temperature for 20 min during which time a white precipitate formed. The solvent was removed *in vacuo*. Et_2O (10 mL) was added and the suspension placed in a sonicator for 1 min, then filtered by gravity. The reaction vial and filter cake were rinsed/washed with further Et_2O (2 × 10 mL) and the filter cake was air dried to give the product (77.0 mg, 83%) as a white solid. ***Step 2** (anion exchange):* To a portion of the product from *Step 1* (14.8 mg, 0.016 mmol, 1.0 equiv) was added sequentially sodium tetrakis[(3,5-bis(trifluoromethyl)phenyl]borate (13.8 mg, 0.016 mmol, 1.0 equiv) and lab grade CH_2Cl_2 (0.7 mL). The mixture was stirred at room temperature under air for 2 h before being filtered through a cotton plug inside a pipette. The reaction vial and plug were washed with further CH_2Cl_2 (5 mL) and the filtrate was concentrated *in vacuo* to give the product (26.0 mg, 99% or 82% over two steps) as a white solid. **^{1}H NMR** (500 MHz, $CDCl_3$) δ_H 3.37-3.55 (m, 3H, 3 x NCH^A), 3.58-3.71 (m, 3H, 3 x NCH^A), 4.01 (s, 3H, N^+CH_3), 4.54-4.63 (m, 1H, NCH^B), 4.67-4.75 (m, 1H, NCH^B), 4.77-4.83 (m, 1H, NCH^B), 5.16-5.26 (m, 1H, NCH^B), 5.28-5.41 (m, 2H, 2 x NCH^B), 7.49 (s, 2H, 2 x Ar*H*), 7.52 (s, 4H, 4 x Ar*H*), 7.58 (dd, J = 6.1, 8.5, 1H, Ar*H*), 7.65 (s, 2H, 2 x Ar*H*), 7.67-7.72 (m, 10H, 10 x Ar*H*), 7.89 (d, J = 5.9, 1H, Ar*H*), 8.28 (d, J = 8.5, 1H, Ar*H*), 8.99 (s, 1H, Ar*H*), 9.43 (s, 1H, N*H*), 10.04 (s, 1H, N*H*), 10.83 (s, 1H, N*H*). **^{13}C NMR** (126 MHz, $CDCl_3$) δ_C 48.7 (N*C*H_2), 49.0 (N*C*H_2), 49.1 (N^+CH_3), 49.4 (N*C*H_2), 51.2 (N*C*H_2), 52.7 (N*C*H_2), 53.0 (N*C*H_2), 117.7 (pentet, J = 4.0, 4 x Ar*C*), 119.6 (Ar*C*H), 119.8 (Ar*C*H), 122.7 (q, J = 273.3, 2 x *C*F_3), 122.9 (q, J = 273.2, 2 x *C*F_3), 123.9 (2 x Ar*C*H), 124.7 (q, J = 272.8, 8 x *C*F_3), 125.2 (2 x Ar*C*H), 128.0 (Ar*C*), 129.1 (qdd, J = 2.7, 5.5, 31.4, 8 x Ar*C*), 131.9 (q, J = 33.4, 2 x Ar*C*CF_3), 132.3 (q, J = 33.4, 2 x Ar*C*CF_3), 134.9 (8 x Ar*C*), 137.6 (Ar*C*), 138.0 (Ar*C*), 138.2 (Ar*C*), 140.1 (Ar*C*N), 140.3 (Ar*C*N), 141.7 (Ar*C*N), 161.8 (q, J = 50.1, 4 x Ar*C*B), 183.1 (*C*S), 183.7 (*C*S), 183.8 (*C*S). **^{19}F NMR** (376 MHz, $CDCl_3$) δ_F −63.3, −63.2, −62.2. **HR-MS** (ESI, positive ion mode) – *m/z* for $[C_{31}H_{28}F_{12}N_7S_3]^+$ = 822.1377. Found 822.1375.

***N*[1],*N*[4]-Bis(3,5-bis(trifluoromethyl)phenyl)-7-formyl-1,4,7-triazonane-1,4-bis(carbothioamide), 7**

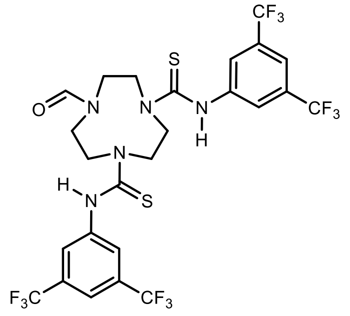

To a solution of **5-2** (20.1 mg, 0.030 mmol, 1.0 equiv) in lab grade CH_2Cl_2 (0.3 mL) under air at room temperature was added 2,2,2-trifluoroethyl formate (18 μL, 0.19 mmol, 6.2 equiv) and the solution was stirred under air at room temperature for 19 h. The solution was concentrated *in vacuo* to give the product (19.7 mg, 94%) as a white solid. **TLC** – R_f = 0.58 (SiO_2, 5:95 MeOH:CH_2Cl_2). **^{1}H NMR** (500 MHz, $CDCl_3$) δ_H 3.11-3.59 (m, 12H, 6 x NC*H*$_2$), 7.52 (s, 1H, Ar*H*), 7.67 (s, 1H, Ar*H*), 7.77 (s, 2H, 2 x Ar*H*), 8.01 (s, 2H, 2 x Ar*H*), 8.27 (s, 1H, C*H*O), 9.20 (s, 1H, N*H*), 9.55 (s, 1H, N*H*). **^{13}C NMR** (126 MHz, $CDCl_3$) δ_C 43.7 (N*C*H$_2$), 46.4 (N*C*H$_2$), 48.6-49.2 (2 x N*C*H$_2$), 51.4 (N*C*H$_2$), 52.2 (N*C*H$_2$), 118.4-118.7 (2 x Ar*C*H), 123.0 (q, J = 273.6, 2 x *C*F$_3$), 123.1 (2 x Ar*C*H), 123.2 (q, J = 273.6, 2 x *C*F$_3$), 124.2 (2 x Ar*C*H), 131.6 (q, J = 33.7, 2 x Ar*C*CF$_3$), 132.0 (q, J = 33.7, 2 x Ar*C*CF$_3$), 123.8 (4 x Ar*C*H), 131.9 (q, J = 33.7, 4 x Ar*C*CF$_3$), 140.6 (Ar*C*N), 140.7 (Ar*C*N), 167.1 (*C*HO), 183.4 (*CS*), 184.1 (*CS*). **^{19}F NMR** (376 MHz, $CDCl_3$) δ_F −63.2, −63.0. **HR-MS** (ESI, positive ion mode) – *m/z* for $[C_{25}H_{21}F_{12}N_5OS_2+H]^+$ = 700.1074. Found 700.1088.

(7a*R*,11a*S*)-1,4,7-Tris(4-methoxybenzyl)dodecahydro-1*H*-benzo[*b*][1,4,7]triazonine, 8-1

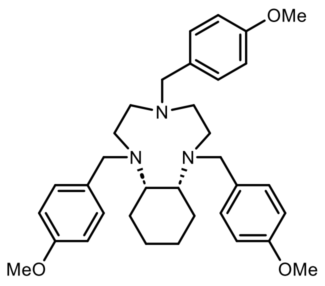

To a solution of **3-3** (651.1 mg, 1.14 mmol, 1.0 equiv) in dry THF (20 mL) at room temperature under N_2 in a multi-neck round bottom flask was added over 2 min in portions solid $LiAlH_4$ powder (864.5 mg, 22.78 mmol, 20.0 equiv) through one neck under a positive pressure of N_2. The resulting suspension was stirred at room temperature for 19.5 h. After cooling in an ice bath, water (0.86 mL) was slowly added through one neck under a positive pressure of N_2 (H_2 evolution!). The ice bath was removed and the suspension was diluted with lab grade THF (30 mL), followed by the addition of 15% w/w NaOH (0.86 mL) and further water (2.59 mL). The mixture was stirred at room temperature for 45 min, then Na_2SO_4 (several spatulas worth) was added as a drying agent. After 2 min of further stirring, the suspension was gravity filtered and the filter cake and reaction flask were rinsed with further THF (50 mL). The filtrate was concentrated *in vacuo*. Flash chromatography (Biotage, 25 g Sfär Duo column, MeOH/[35% aqueous NH_3]/CH_2Cl_2 gradient from 0:0:100 to 10:2:88) gave the product (233.1 mg, 38%) as a colourless gum. **TLC** – R_f = 0.17 (SiO_2, 7.5:1.5:91 MeOH/[35% aqueous NH_3]/CH_2Cl_2). **^{1}H NMR** (500 MHz, $CDCl_3$, note that one proton signal of the TACN ring of 2H integration is too broad to be observed) δ_H 1.29 – 1.40 (m, 2H), 1.50 – 1.63 (m, 2H), 1.72 – 1.86 (m, 2H), 1.95 – 2.14 (m, 4H), 2.46 – 2.60 (m, 2H), 2.74 – 2.90 (m, 2H), 2.98 – 3.18 (m, 2H), 3.37 (d, *J* = 13.7 Hz, 2H), 3.46 (s, 2H), 3.80 (s, 3H), 3.81 (s, 6H), 3.99 (d, *J* = 13.7 Hz, 2H), 6.80 – 6.88 (m, 6H), 7.17 (d, *J* = 8.1 Hz, 2H), 7.22 (d, *J* = 8.2 Hz, 4H). **^{13}C NMR** (126 MHz, $CDCl_3$, note that the expected 2×methoxy signals are not resolved) δ_C 23.8 (broad),

26.5 (broad), 51.9 (very broad), 55.35, 55.41 (broad), 56.0 (broad), 63.0 (very broad), 64.1, 113.5, 113.6, 129.8, 130.2, 133.0, 133.5, 158.3, 158.5. **HR-MS** (ESI, positive ion mode) – *m/z* for $[C_{34}H_{45}N_3O_3+H]^+$ = 544.3534. Found 544.3526.

(7a*R*,11a*S*)-Dodecahydro-4*H*-benzo[*b*][1,4,7]triazonine-4-carbaldehyde, 8-2

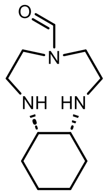

Step 1 (*selective mono-PMB cleavage*)*:* To neat **8-1** (233.1 mg, 0.43 mmol, 1.0 equiv) under air at room temperature was added a solution of 1-chloroethyl chloroformate (61.3 mg, 0.43 mmol, 1.0 equiv) in lab grade 1,2-dichloroethane (0.86 mL). The solution was heated to 70 °C (round bottom flask with glass stopper) under air and maintained at this temperature for 19.5 h. The resulting brown solution was concentrated *in vacuo*. To the residue was added lab grade MeOH (2 mL) and the solution was heated to 60 °C (round bottom flask with glass stopper) under air and maintained at this temperature for 1.5 h. The MeOH was removed *in vacuo*. CH_2Cl_2 (20 mL) was added and the solution was washed with 10% w/w NaOH (10 mL). The aqeuous layer was back extracted with CH_2Cl_2 (10 mL) and the organic extracts combined, dried (Na_2SO_4) and concentrated. ^{1}H NMR spectroscopic analysis showed the desired mono-deprotected product along with unreacted starting material in a molar ratio of approximately 2.7:1, as well as the expected 4-methoxybenzyl methy ether by-product; this mixture was used directly in the next step. ***Step 2*** (*formylation*)*:* The mixture obtained from *Step 1* was taken up in lab grade CH_2Cl_2 (0.86 mL) and 2,2,2-trifluoroethyl formate (0.21 mL, 2.14 mmol, 5.0 equiv relative to the amount of **8-1** used originally) was added. The solution was stirred under air at room temperature (round bottom flask with glass stopper) for 1.5 h. The solution was concentrated *in vacuo* and the residue was re-evaporated from CH_2Cl_2 (ca 10 mL) to ensure complete removal of 2,2,2-trifluoroethyl formate (which could potentially react with NH_3 during subsequent chromatography). Flash chromatography (Biotage, 10 g Sfär Duo column, MeOH/[35% aqueous NH_3]/CH_2Cl_2 gradient from 0:0:100 to 2:0.4:97.6) gave the formylated product (99.5 mg, 51% over two steps) as a yellow gum with approximately 20−30 mol% unknown impurities as judged by NMR spectroscopy;* this mixture was used in the next step without further purification. *Further elution from the chromatography column (MeOH/[35% aqueous NH_3]/CH_2Cl_2 gradient from 2:0.4:97.6 to 6.4:1.3:92.3) gave the unreacted starting material **8-1** (38.2 mg, 16%). ***Step 3*** (*hydrogenolysis*)*:* A two-neck, 10 mL round bottom flask was charged under air with palladium on carbon (10% w/w, 68.6 mg total, 0.064 mmol of Pd, 0.5 equiv of Pd). The flask was then purged with N_2. In a separate 5 mL round bottom flask, a portion of the impure, formylated product from *Step 2* (58.2 mg, 0.13 mmol, 1.0 equiv) was taken up in lab grade MeOH (3 mL) under air and to this was added acetic acid (15.5 mg, 0.26 mmol, 2.0 equiv). The flask was fitted with a septum and N_2 (from a balloon) was bubbled through the solution for 5 min. The solution was then slowly added to the flask containing palladium on carbon under N_2. Once the addition was complete, the flask was evacuated and refilled with N_2 three times, then, under a positive pressure of N_2, one of the necks was fitted with a three-way tap containing a H_2-filled balloon (the H_2 was closed off to the reaction initially). The system

was evacuated and refilled with N_2, then evacuated and refilled with H_2 three times, before being heated to 50 °C (DrySyn block) for 2 h. After cooling to room temperature, the system was evacuated and refilled with N_2 twice, before the reaction mixture was filtered through a pad of celite with the aid of CH_2Cl_2 (15 mL). The reaction flask and celite pad were rinsed with further CH_2Cl_2 (15 mL) and the filtrate was concentrated *in vacuo.* The residue was dissolved in CH_2Cl_2 (25 mL) and washed with 1 M K_2CO_3 (15 mL). The aqueous layer was back-extracted with CH_2Cl_2 (15 mL) and the combined organic layers were dried (Na_2SO_4) and concentrated *in vacuo*. Flash chromatography (Biotage, 5 g Sfär Duo column, MeOH/[35% aqueous NH_3]/CH_2Cl_2 gradient from 0:0:100 to 10:2:88) gave the product (15.1 mg, 56% or 29% over three steps) as a colourless gum with approximately 5 mol% of an unknown impurity as judged by NMR spectroscopy. **TLC** – R_f = 0.12 (SiO_2, 7.5:1.5:91 MeOH/[35% aqueous NH_3]/CH_2Cl_2). **^{1}H NMR** (500 MHz, $CDCl_3$) δ_H 1.20 – 1.61 (m, 8H), 2.56 – 2.61 (m, 1H), 2.65 – 2.74 (m, 2H), 2.81 (ddd, *J* = 14.6, 7.9, 3.0 Hz, 1H), 3.10 (ddd, *J* = 13.9, 7.9, 3.0 Hz, 1H), 3.15 – 3.27 (m, 3H), 3.50 (dt, *J* = 13.7, 3.0 Hz, 1H), 3.63 (ddd, *J* = 13.9, 6.2, 3.0 Hz, 1H), 8.15 (s, 1H). **^{13}C NMR** (126 MHz, $CDCl_3$) δ_C 21.7 (broad), 22.8 (broad), 29.4, 29.7, 45.3, 47.0 (broad), 49.1 (broad), 52.8, 55.5 (very broad), 57.4 (broad), 164.3. **HR-MS** (ESI, positive ion mode) – *m/z* for $[C_{11}H_{21}N_3O+H]^+$ = 212.1757. Found 212.1757.

(7a*R*,11a*S*)-*N*^{1},*N*^{7}-Bis(3,5-bis(trifluoromethyl)phenyl)-4-formyldecahydro-1*H*-benzo[*b*][1,4,7]triazonine-1,7(2*H*)-bis(carbothioamide), 8

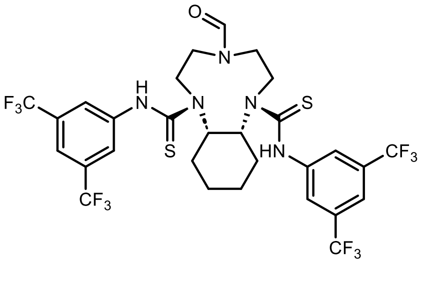

To neat **8-2** (6.6 mg, 0.031 mmol, 1.0 equiv) in a 10 mL round bottom flask under air was added a solution of 3,5-bis(trifluoromethyl)phenyl isothiocyanate (21.2 mg, 0.078 mmol, 2.5 equiv) in lab grade 1,2-dichloroethane (0.62 mL) and the solution was heated at 45 °C for 2.5 h, then at 40 °C for 64.5 h, at which point TLC analysis (MeOH/CH_2Cl_2 2.5:97.5) showed complete consumption of **8-2** and the formation of **8**, but a significant amount of the mono-functionalised intermediate (which itself appears as two close spots on TLC due to non-degenerate formamide rotamers). Additional 3,5-bis(trifluoromethyl)phenyl isothiocyanate (10.5 mg, 0.039 mmol, 1.2 equiv) was added as a solution in 1,2-dichloroethane (0.2 mL) and the mixture was heated at 50 °C for a further 5.5 h, then at 45 °C for another 21 h. The solvent was removed *in vacuo*. Flash chromatography (Biotage, 5 g Sfär Duo column, MeOH/CH_2Cl_2 gradient from 0:100 to 1.4:98.6) initially gave the product with some co-elution of the mono-functionalised intermediate. The fractions containing **8** were concentrated. Further flash chromatography – this time with a lower polarity gradient – (Biotage, 5 g Sfär Duo column, MeOH/CH_2Cl_2 gradient from 0:100 to 0.7:99.3) gave the product (9.3 mg, 40%) as a colourless gum. **TLC** – R_f = 0.49 (SiO_2, 2.5:97.5 MeOH:CH_2Cl_2). **^{1}H NMR** (500 MHz, $CDCl_3$) δ_H 1.62 – 1.79 (m, 3H), 1.99 – 2.07 (m, 2H), 2.12 – 2.28 (m, 3H), 3.16 (dd, *J* = 14.5, 4.6 Hz, 1H), 3.26 – 3.36 (m, 1H), 3.71 – 3.78 (m, 1H), 3.85 (dd, *J* = 16.7, 6.9 Hz, 1H), 4.01 (dd, *J* = 14.8, 6.6

Hz, 1H), 4.47 (dd, J = 14.6, 4.7 Hz, 1H), 4.71 – 4.80 (m, 1H), 5.15 – 5.23 (m, 1H), 5.54 – 5.61 (m, 1H), 5.72 – 5.77 (m, 1H), 7.50 (s, 1H), 7.68 (s, 1H), 7.81 (s, 2H), 7.95 (s, 2H), 8.23 (s, 1H), 9.37 (s, 1H), 9.56 (bs, 1H). **^{13}C NMR** (126 MHz, $CDCl_3$) δ_C 21.2, 24.1, 25.9, 29.2, 45.7, 46.0, 46.2, 47.3, 56.2, 58.9, 118.2 (pentet, J = 3.8 Hz), 118.8 (pentet, J = 3.8 Hz), 119.8 – 126.5 (4×overlapping quartets), 131.2 – 132.3 (2×overlapping quartets), 141.0, 141.1, 166.8, 184.76, 184.83. **^{19}F NMR** (376 MHz, $CDCl_3$) δ_F −63.2, −63.0. **HR-MS** (Nanospray with HCO_2H, positive ion mode) – *m/z* for $[C_{29}H_{27}F_{12}N_5OS_2+H]^+$ = 754.1544. Found 754.1540.

1,4,7,10,13,16-Hexakis(4-methoxyanilinylcarbonyl)-1,4,7,10,13,16-hexaazacyclooctadecane, 9

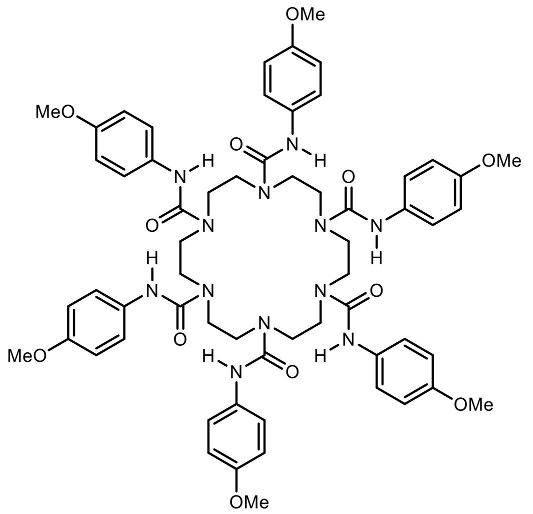

To a suspension of hexacyclen (25.8 mg, 0.10 mmol, 1.0 equiv) in lab grade CH_2Cl_2 (0.5 mL) at room temperature under air was added a solution of 4-methoxyphenyl isocyanate (94.0 mg, 0.63 mmol, 6.3 equiv) in CH_2Cl_2 (1.5 mL) [note that the isocyanate solution was passed through a pipette cotton plug upon addition to the reaction vessel to remove a trace of a solid impurity]. The solution was stirred at room temperature under air (vial with screw cap) for 40 min. The reaction was quenched with MeOH (1 mL) and stirred at room temperature for a further 1 h, before the solvent was removed *in vacuo*. To the white solid residue in the vial was added Et_2O (7 mL) and the suspension was sonicated for 2 min, then gravity filtered. Residual solid in the vial was rinsed on to the filter paper with further Et_2O (7 mL) and the filter cake was air dried to give the product (67.8 mg) of approximate 90 mol% purity as judged by ^{1}H NMR analysis. To purify further, this was dissolved in CH_2Cl_2 (15 mL) and MeOH (7 mL) was added. The solution was subjected to rotary evaporation (40 °C, ≥500 mbar) to selectively remove the CH_2Cl_2 and the resulting precipitate was collected by gravity filtration and washed seqentially with MeOH (10 mL) and petroleum ether (20 mL), then air dried to give the product (39.4 mg, 34%) as a white powder. **TLC** – R_f = 0.29 (SiO_2, 5:95 MeOH:CH_2Cl_2). **^{1}H NMR** (500 MHz, $C_2D_2Cl_4$) δ_H 3.52 (s, 12H, 12 x CH^AH^B), 3.62 (s, 12H, 12 x C$H^A$$H^B$), 3.77 (s, 18H, 18 x OC$H_3$), 6.77 (d, J = 9.1, 12H, 12 x Ar*H*), 7.42 (d, J = 9.1, 12H, 12 x Ar*H*), 8.70 (s, 6H, 6 x N*H*). **^{13}C NMR** (126 MHz, $C_2D_2Cl_4$) δ_C 49.0 (6 x N*C*H_2), 49.7 (6 x N*C*H_2) 55.5 (6 x O*C*H_3), 113.8 (12 x Ar*C*H), 121.6 (12 x Ar*C*H), 132.1 (6 x Ar*C*N), 155.2 (6 x Ar*C*O), 157.3 (3 x *C*O). **HR-MS** (ESI, positive ion mode) – *m/z* for $[C_{60}H_{72}N_{12}O_{12}+Na]^+$ = 1175.5290. Found 1175.5286.

Crystals of **9** suitable for X-ray analysis were obtained by slow diffusion of water into a solution of **9** in DMF (CCDC: 2262697).

Variable-Temperature ^{1}H NMR Studies of **1**–**3**, **5**–**7**

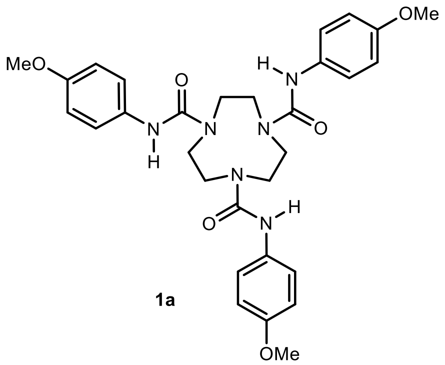

1a

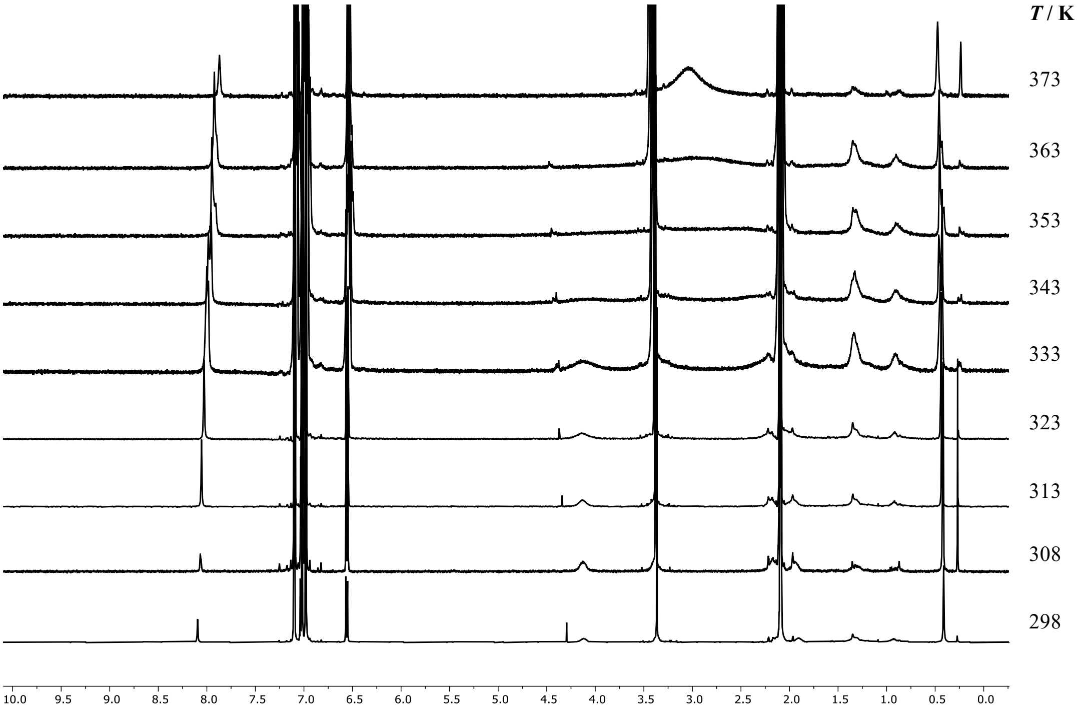

Figure S1 – VT-NMR stack plot for **1a** (500 MHz, d_8-toluene).

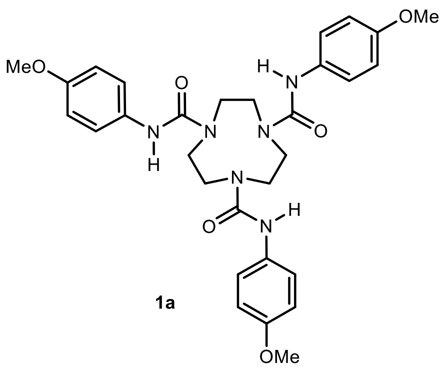

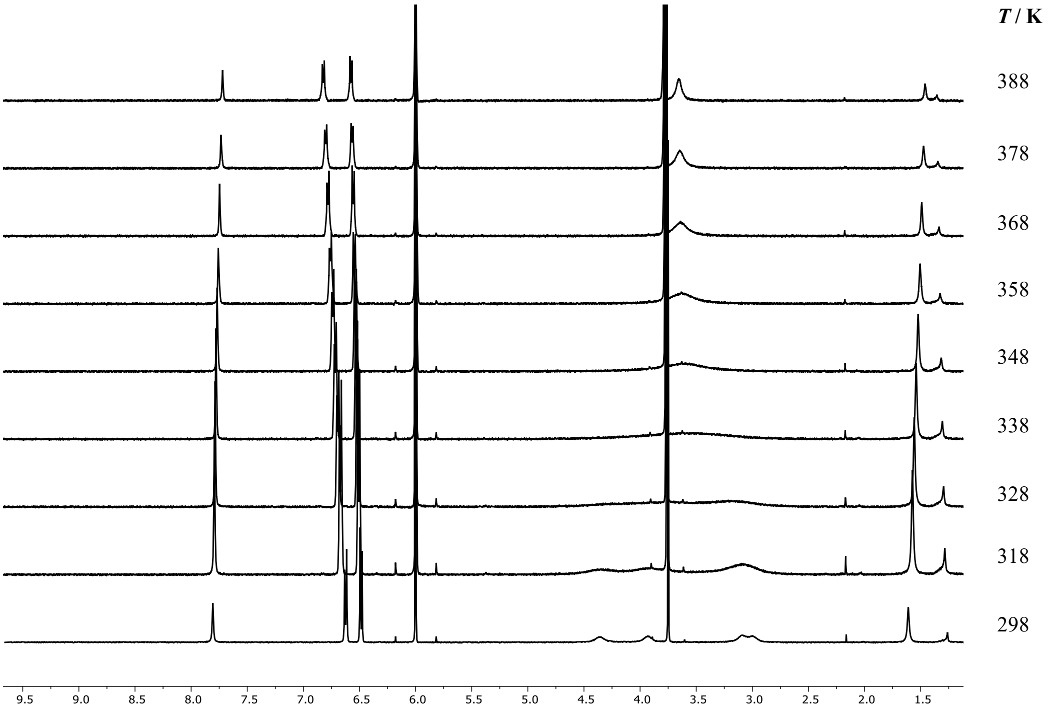

Figure S2 – VT-NMR stack plot for **1a** (500 MHz, d_2-TCE).

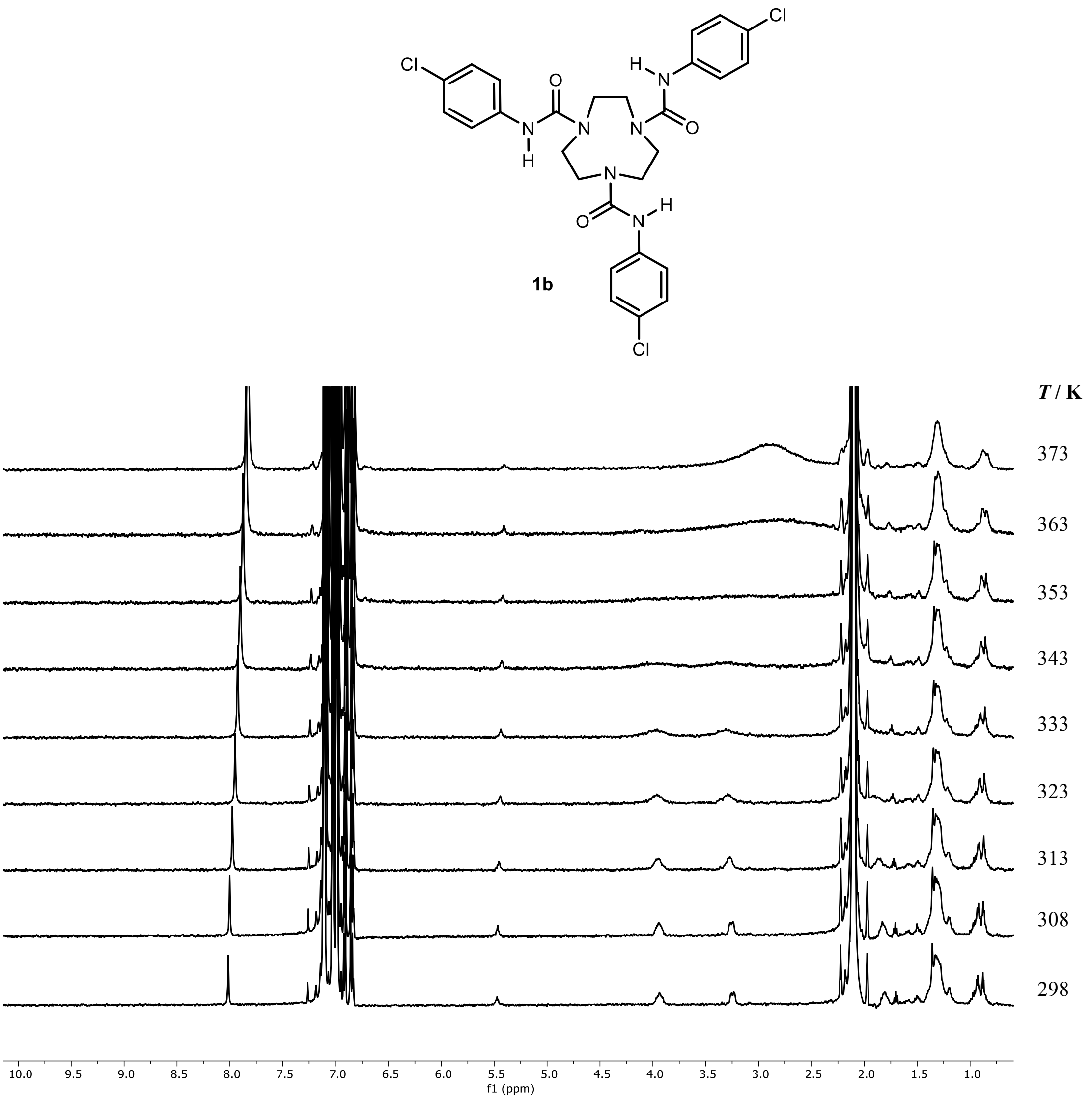

Figure S3 – VT-NMR stack plot for **1b** (500 MHz, d_8-toluene).

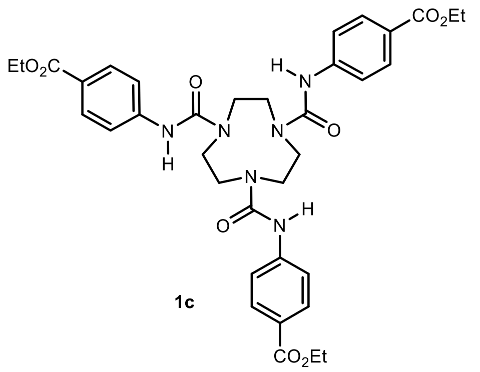

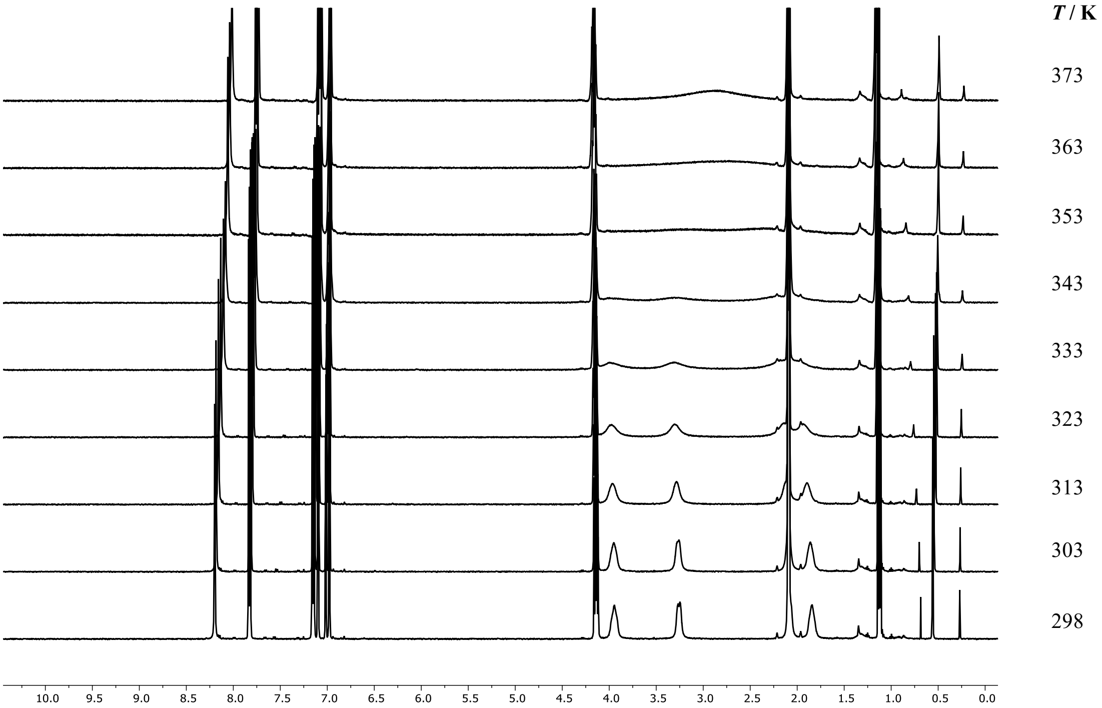

Figure S4 – VT-NMR stack plot for **1c** (500 MHz, d_8-toluene).

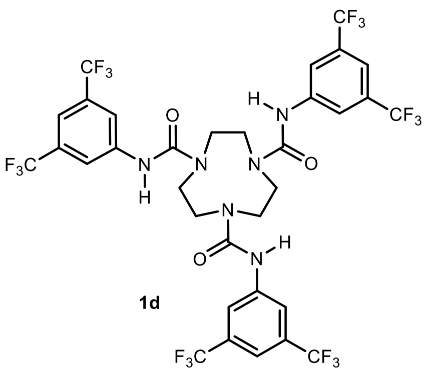

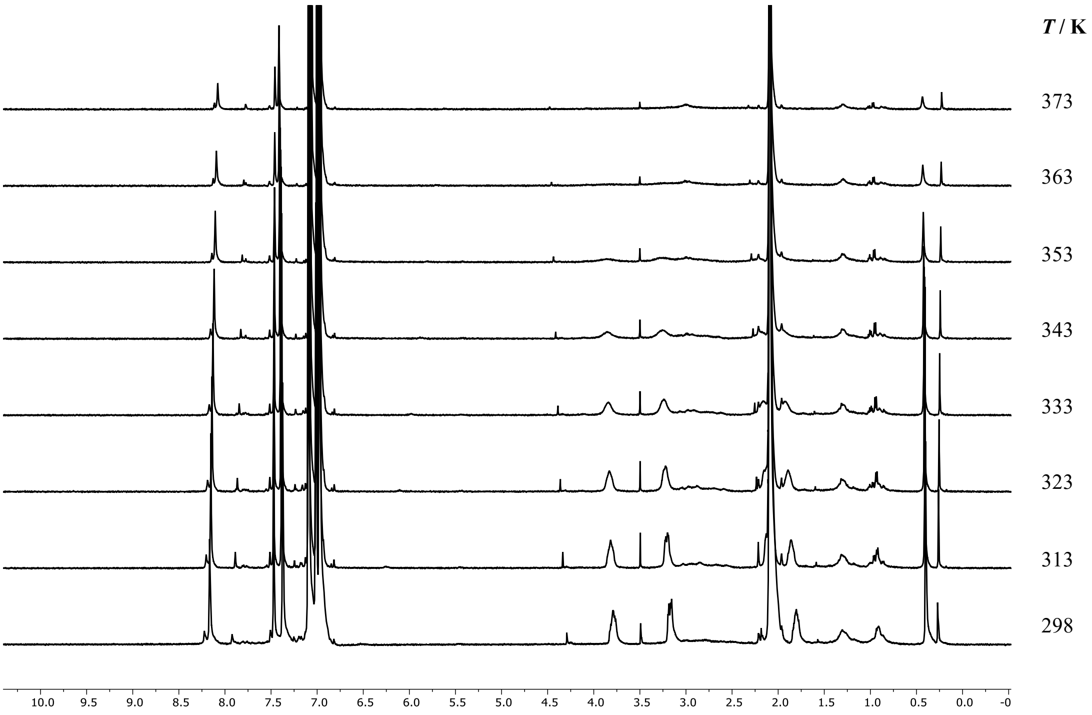

Figure S5 – VT-NMR stack plot for **1d** (500 MHz, *d*8-toluene).

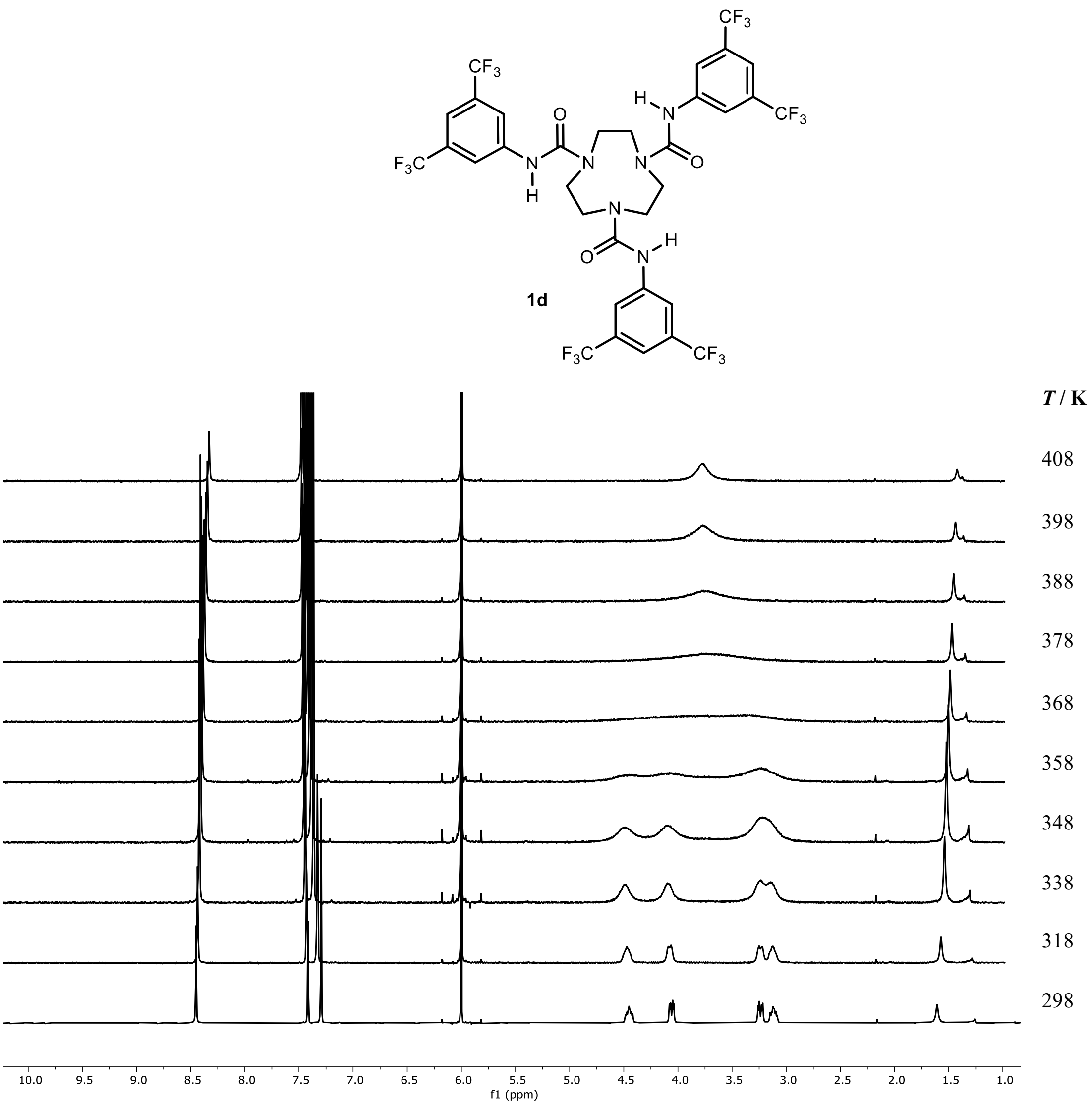

Figure S6 – VT-NMR stack plot for **1d** (500 MHz, d_2-TCE).

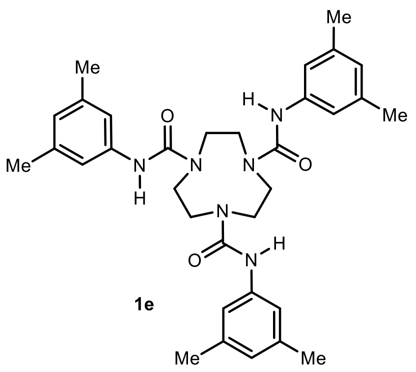

1e

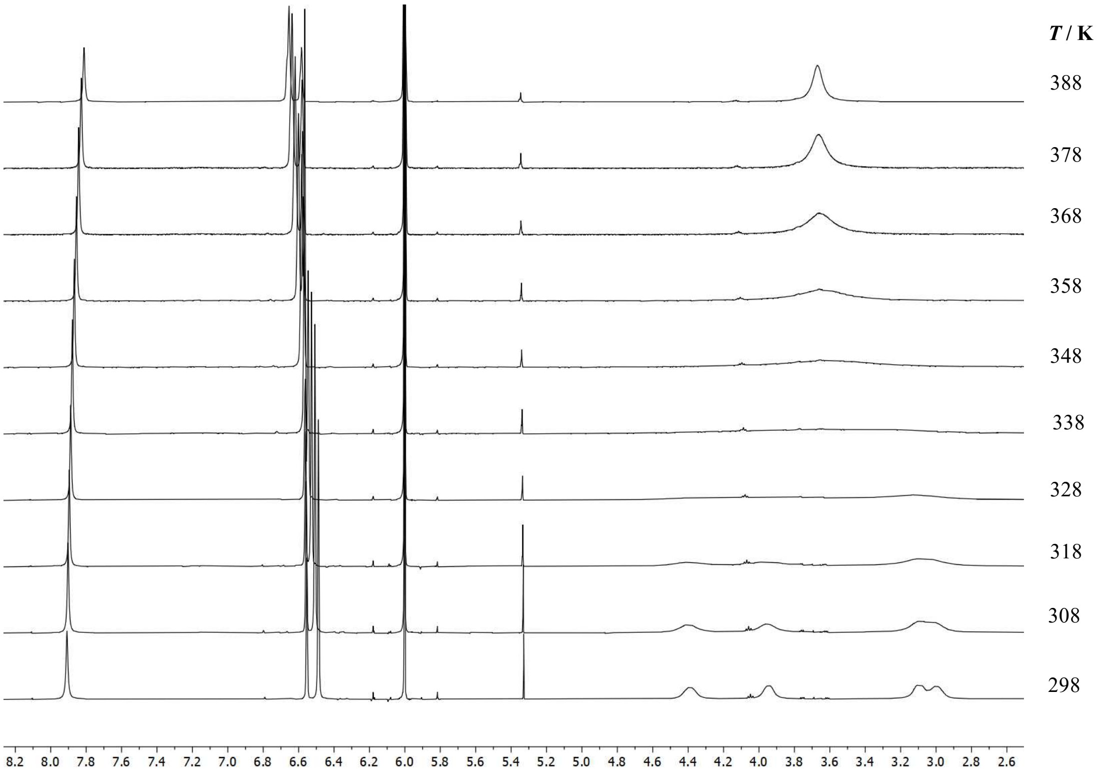

Figure S7 – VT-NMR stack plot for **1e** (500 MHz, d_2-TCE).

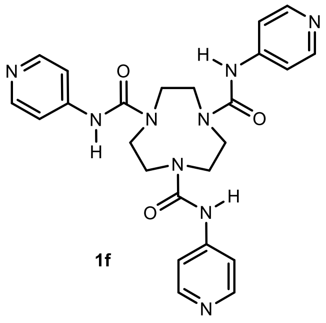

1f

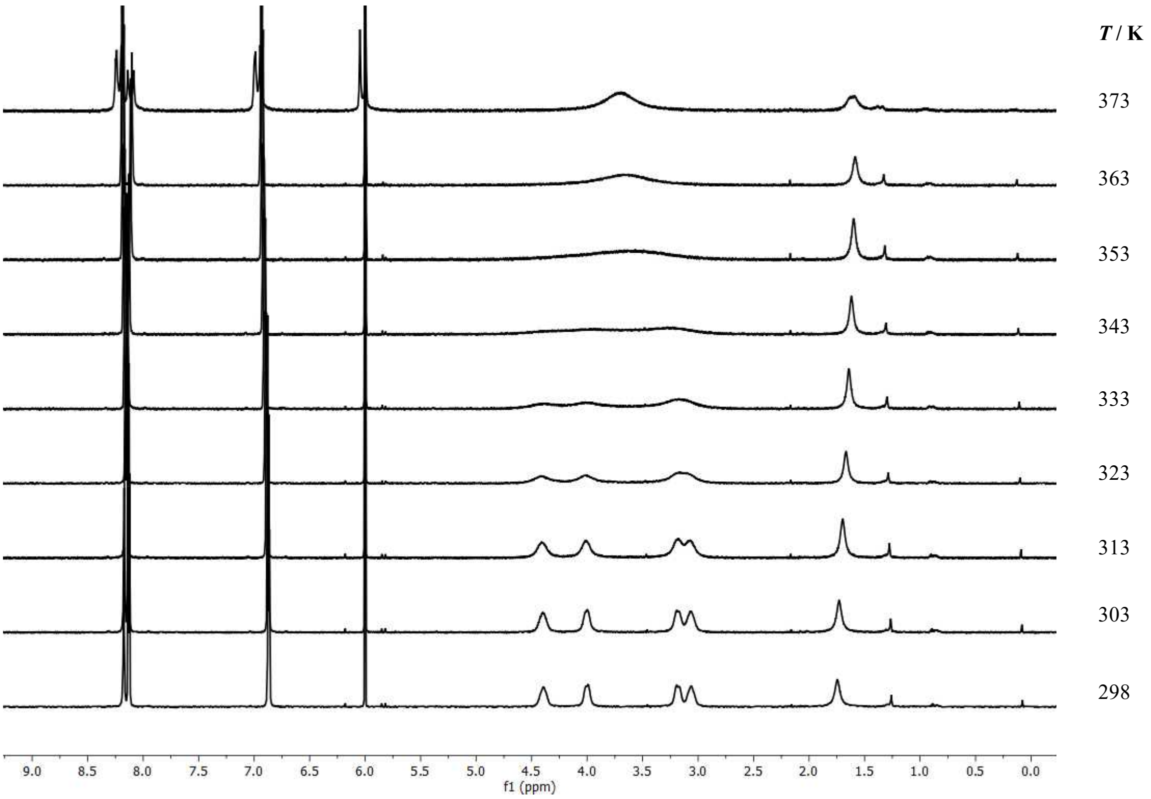

Figure S8 – VT-NMR stack plot for **1f** (500 MHz, d_2-TCE).

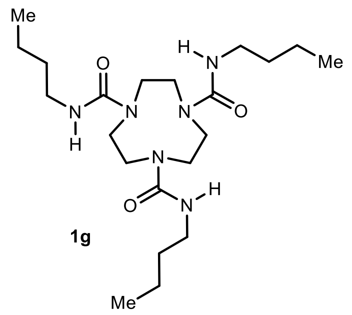

1g

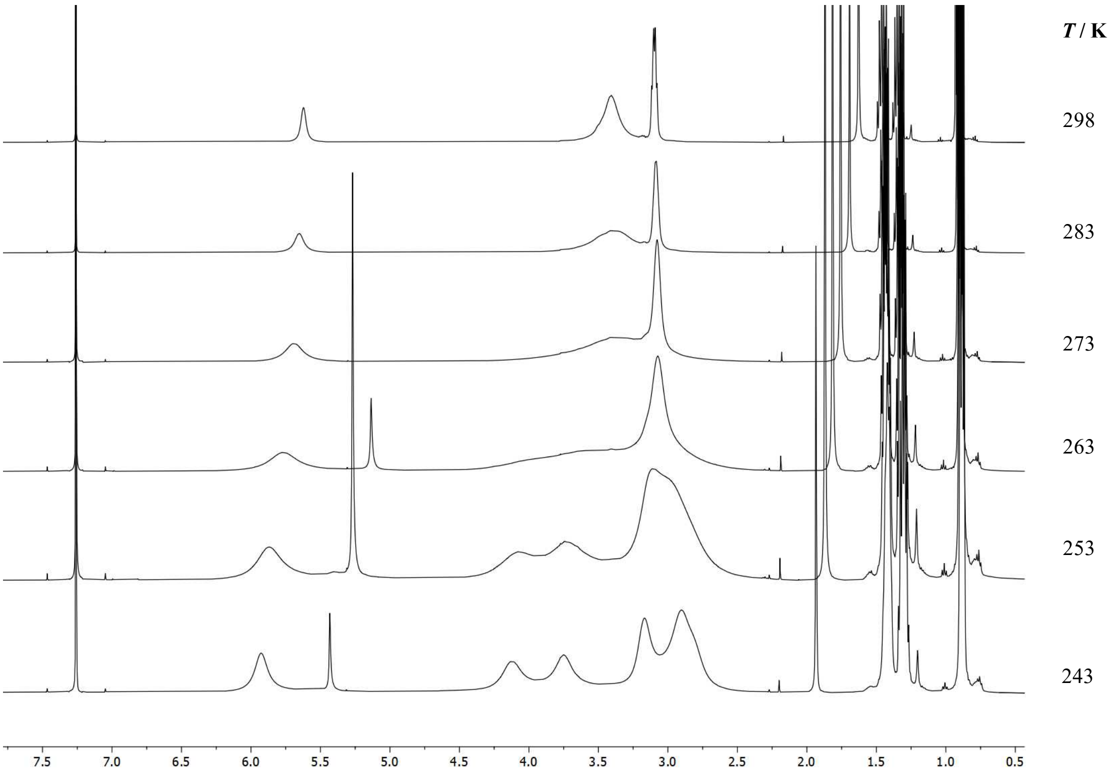

Figure S9 – VT-NMR stack plot for **1g** (500 MHz, $CDCl_3$).

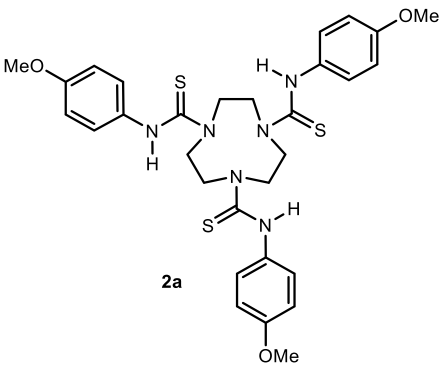

2a

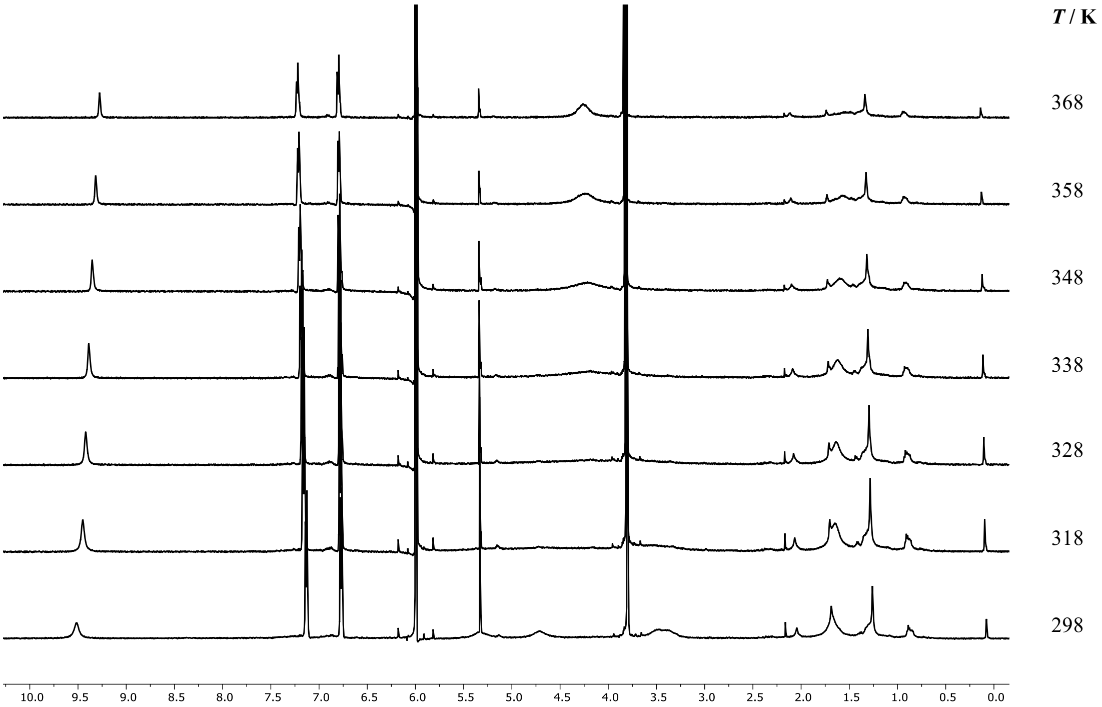

Figure S10 – VT-NMR stack plot for **2a** (500 MHz, d_2-TCE).

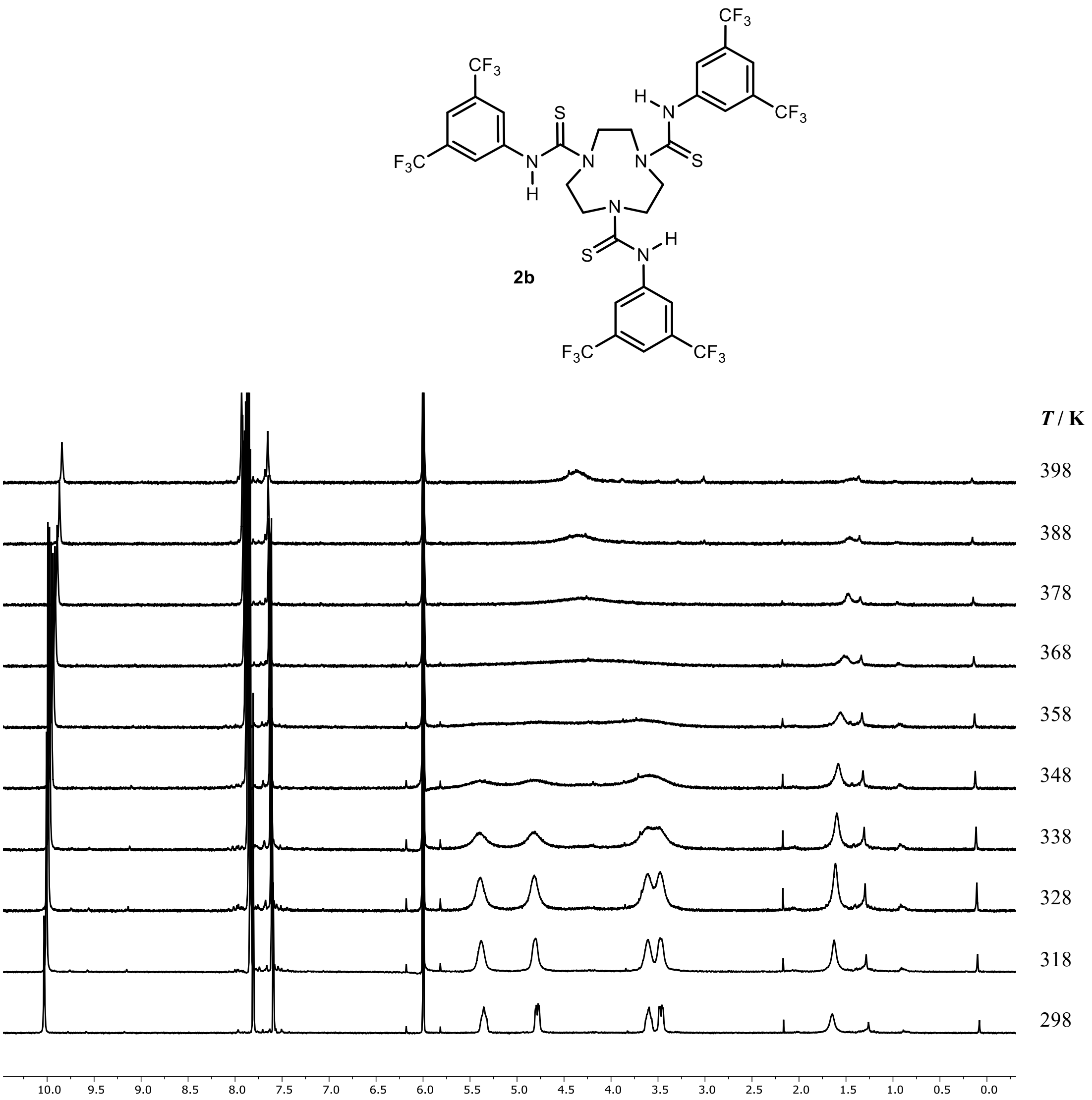

Figure S11 – VT-NMR stack plot for **2b** (500 MHz, d_2-TCE).

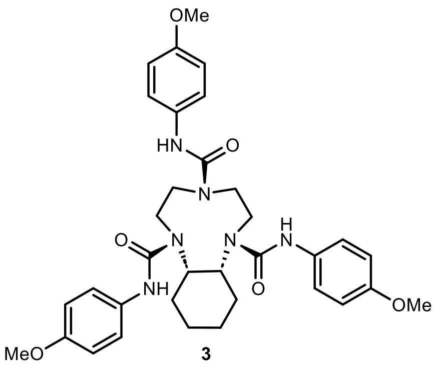

3

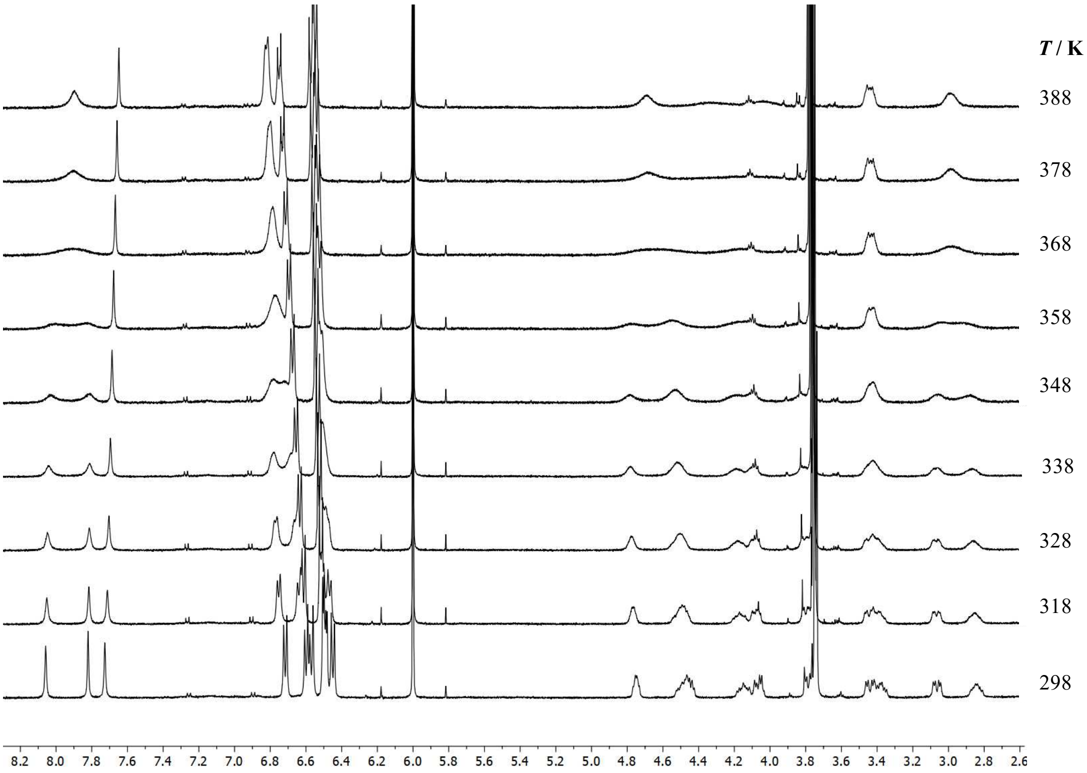

Figure S12 – VT-NMR stack plot for **3** (500 MHz, d_2-TCE).

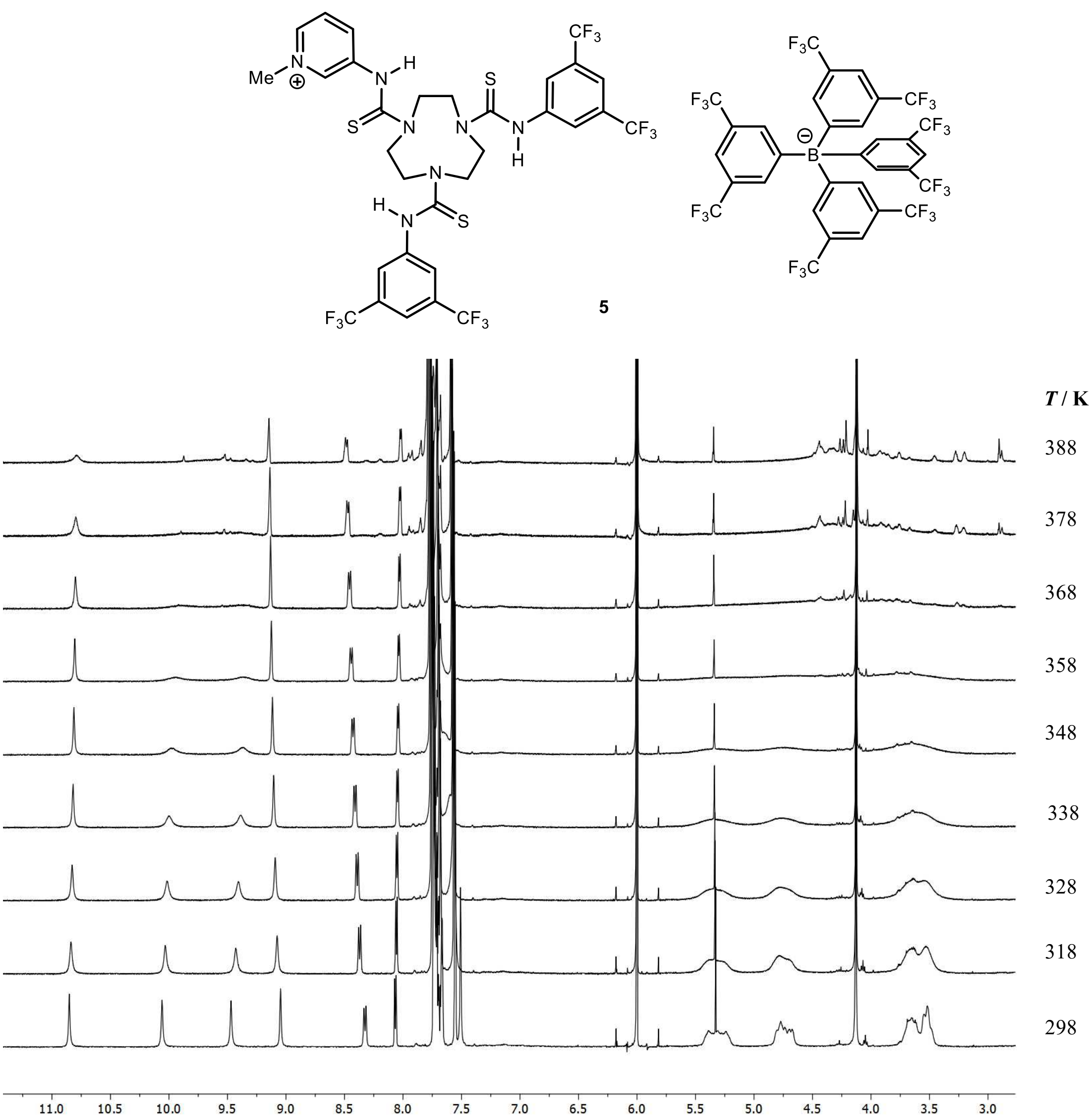

Figure S13 – VT-NMR stack plot for **5** (500 MHz, d_2-TCE).

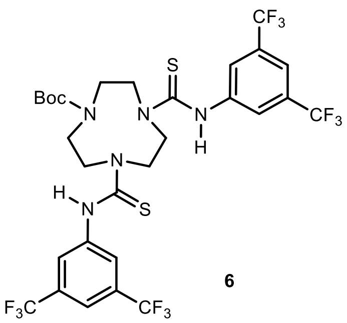

6

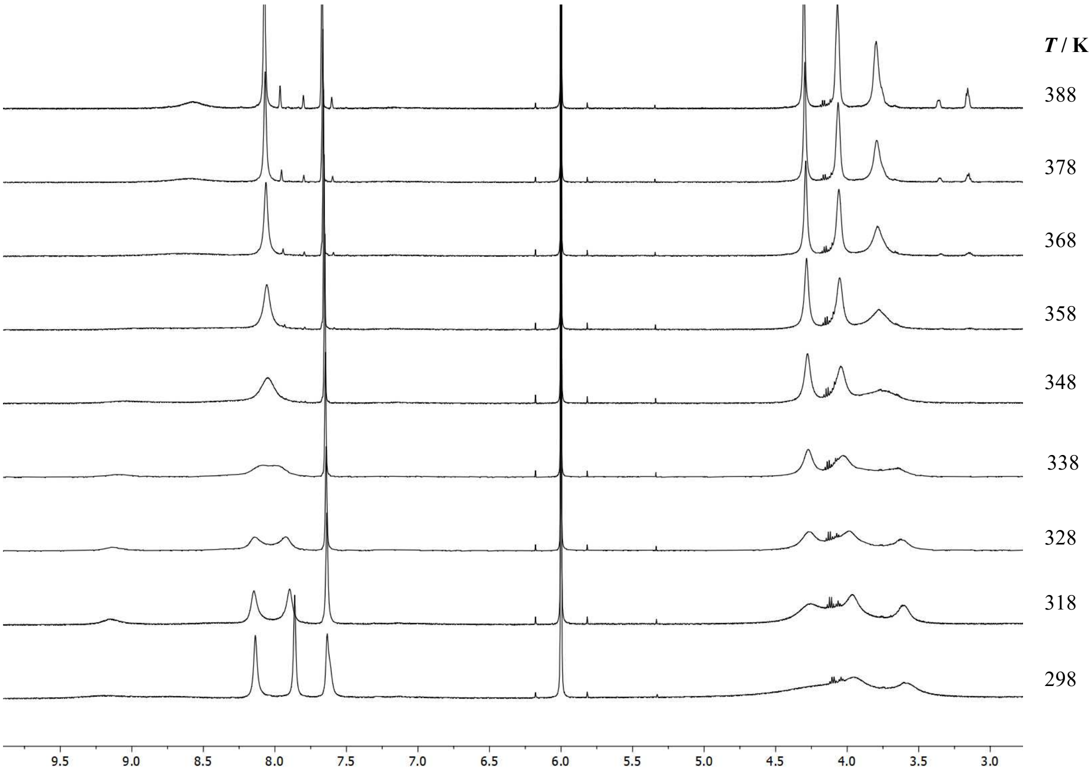

Figure S14 – VT-NMR stack plot for **6** (500 MHz, d_2-TCE).

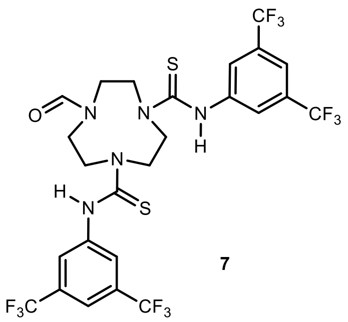

7

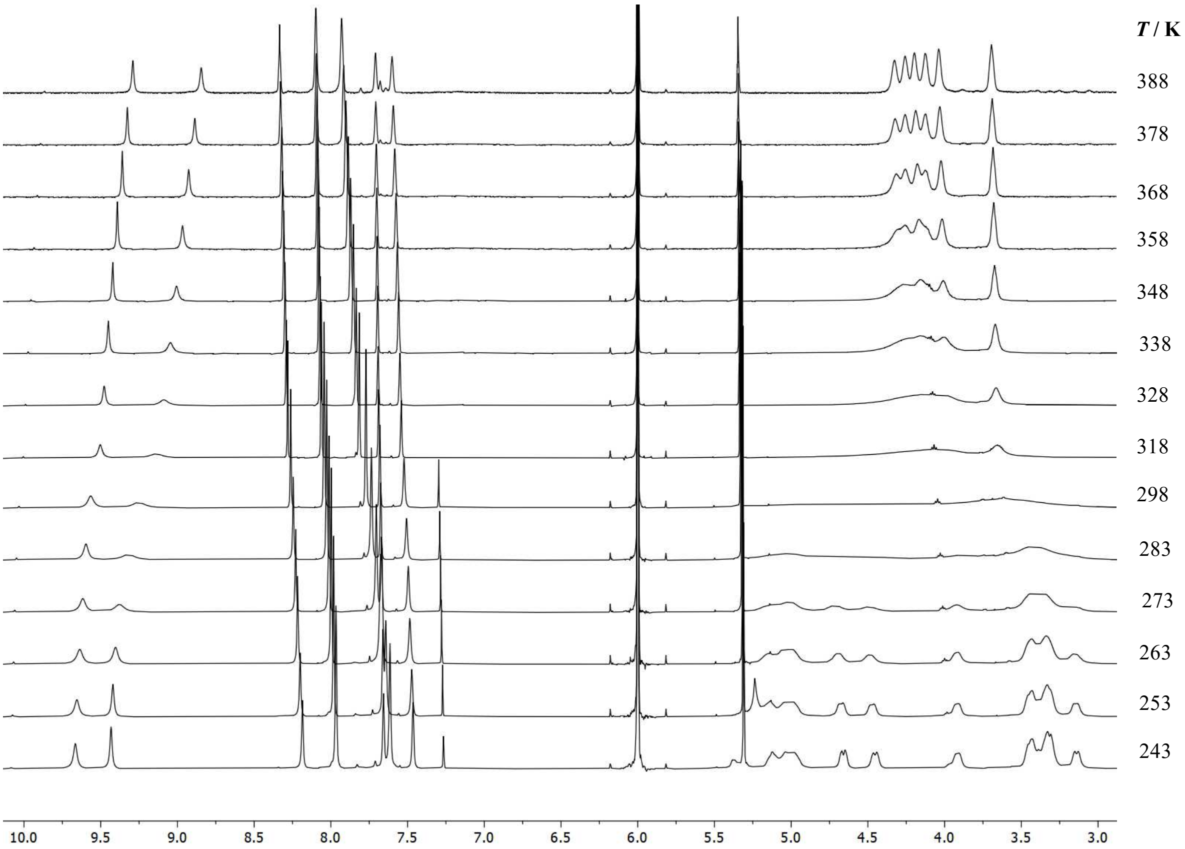

Figure S15 – VT-NMR stack plot for **7** (500 MHz, d_2-TCE).

Eyring Analyses of **1−3**, **5−7**

All Eyring plots were constructed using rate constants that were attained by line shape fitting of experimental NMR spectra at different temperatures. Spectra were simulated using SpinWorks 4 NMR processing software (available at ftp://davinci.chem.umanitoba.ca/pub/marat/SpinWorks/) with appropriate exchange vectors. For each temperature, the corresponding rate of enantiomerization was estimated using the dynamic NMR simulation module DNMR3 by comparing full-width half-maxima and multiplicity resolution with experimental data. The linear form of the Eyring-Polanyi equation was used to give an equation of the form y = mx + c such that a plot of *ln(k/T)* against *1/T* gives a straight line of gradient $\frac{-\Delta H^{\ddagger}}{R}$ and intercept $ln\frac{k_B}{h}+\frac{\Delta S^{\ddagger}}{R}$. These data were used with the Gibbs free energy equation to extrapolate $\Delta G^{\ddagger}$ at 298 K. An exemplar simulation is given in Figure 3 of the manuscript. Scalar couplings were omitted from simulations whose experimental counterparts were in fast enough exchange at room temperature such that splitting was not measurable.

Table S1 – Kinetics data and Eyring analysis of **1a** in d_8-toluene. Rate constants for enantiomerization were determined from the exchange of all four ethylene bridge protons.

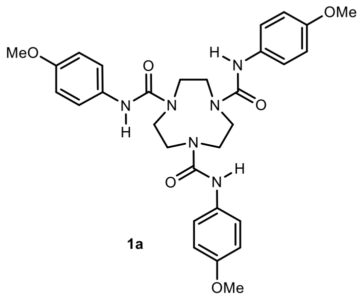

1a

$\Delta G^{\ddagger}_{298\ K}$ = 65.3 kJ mol^{-1}

(d_8-Toluene)

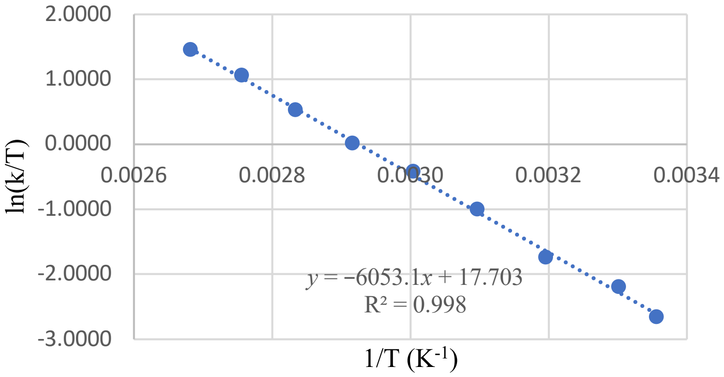

Temperature (K)	Rate Constant, *k* (s^{-1})	1/T (K^{-1})	ln(*k*/T)	ΔG‡ (kJ mol^{-1})	$t_{1/2}$ (s)
298	21	0.0034	-2.6526	65.3357	0.0165
303	34	0.0033	-2.1874	65.5875	0.0102
313	55	0.0032	-1.7389	66.0911	0.0063
323	120	0.0031	-0.9902	66.5947	0.0029
333	220	0.0030	-0.4145	67.0983	0.0016
343	350	0.0029	0.0202	67.6019	0.0010
353	600	0.0028	0.5305	68.1055	0.0006
363	1050	0.0028	1.0621	68.6091	0.0003
373	1600	0.0027	1.4562	69.1128	0.0002

Table S2 – Kinetics data and Eyring analysis of **1a** in d_2-TCE. Rate constants for enantiomerization were determined from the exchange of all four ethylene bridge protons.

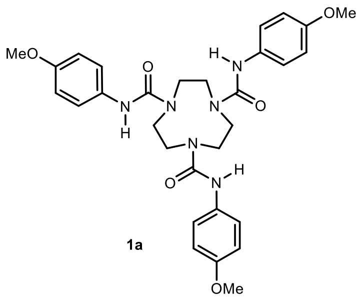

1a

$\Delta G^{\ddagger}_{298\ K}$ = 63.2 kJ mol^{-1}

(d_2-TCE)

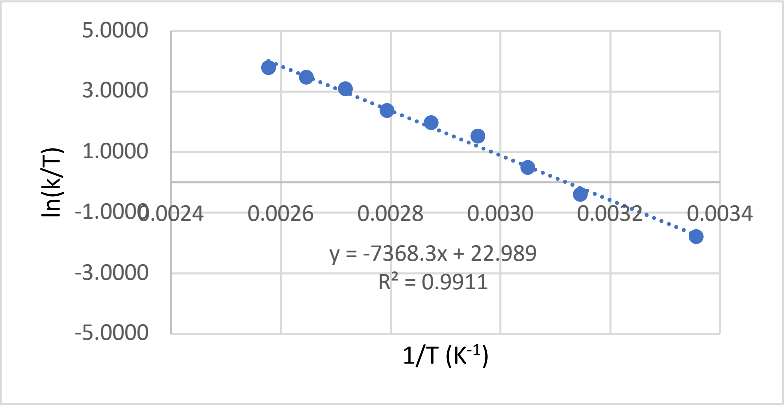

Temperature (K)	Rate Constant, k (s^{-1})	1/T (K^{-1})	ln(k/T)	ΔG‡ (kJ mol^{-1})	$t_{½}$ (s)
298	50	0.0034	-1.7851	63.1737	0.0069
318	215	0.0031	-0.3914	63.3019	0.0016
328	530	0.0030	0.4799	63.3660	0.0007
338	1550	0.0030	1.5230	63.4301	0.0002
348	2500	0.0029	1.9718	63.4942	0.0001
358	3800	0.0028	2.3622	63.5583	0.0001
368	8000	0.0027	3.0791	63.6224	0.0000
378	12000	0.0026	3.4578	63.6865	0.0000
388	17000	0.0026	3.7800	63.7506	0.0000

Table S3 – Kinetics data and Eyring analysis of **1b** in d_8-toluene. Rate constants for enantiomerization were determined from the exchange of all four ethylene bridge protons.

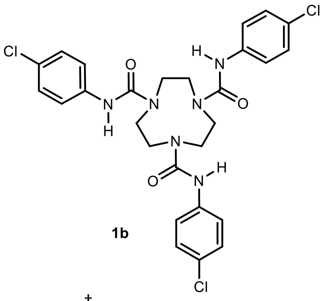

1b

$\Delta G^{\ddagger}_{298\,K} = 63.2$ kJ mol^{-1}

(d_8-Toluene)

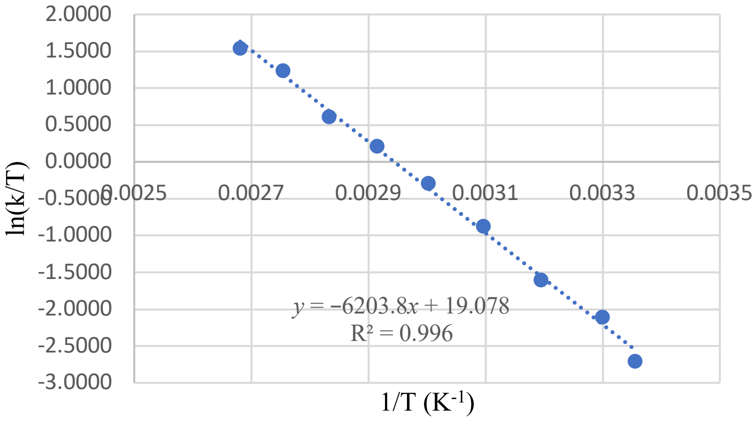

Temperature (K)	Rate Constant, k (s^{-1})	1/T (K^{-1})	ln(k/T)	$\Delta G^{\ddagger}$ (kJ mol^{-1})	$t_{1/2}$ (s)
298	20	0.0034	-2.7014	63.1811	0.0173
303	37	0.0033	-2.1028	63.3757	0.0094
313	63	0.0032	-1.6031	63.7650	0.0055
323	135	0.0031	-0.8724	64.1542	0.0026
333	250	0.0030	-0.2867	64.5435	0.0014
343	425	0.0029	0.2144	64.9328	0.0008
353	650	0.0028	0.6105	65.3220	0.0005
363	1250	0.0028	1.2365	65.7113	0.0003
373	1750	0.0027	1.5458	66.1005	0.0002

Table S4 – Kinetics data and Eyring analysis of **1c** in d_8-toluene. Rate constants for enantiomerization were determined from the exchange of all four ethylene bridge protons.

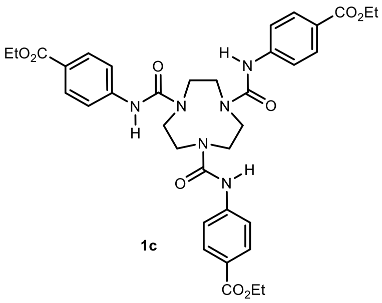

1c

$\Delta G^{\ddagger}_{298\ K}$ = 65.1 kJ mol^{-1}

(d_8-Toluene)

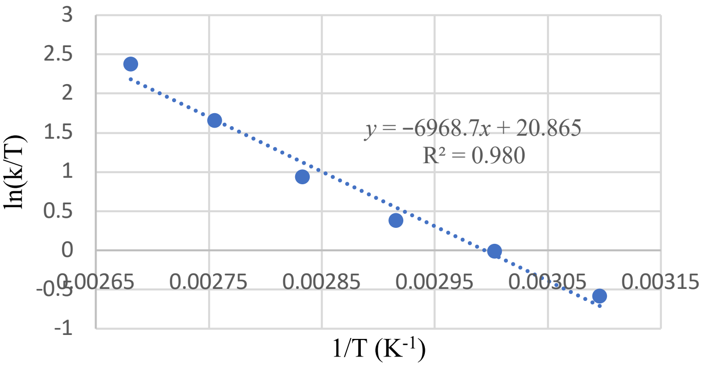

Temperature (K)[a]	Rate Constant, k (s^{-1})	1/T (K^{-1})	ln(k/T)	ΔG‡ (kJ mol^{-1})	t$_{½}$ (s)
323	180	0.0031	-0.5847	65.7157	0.0019
333	330	0.0030	-0.0090	65.9564	0.0011
343	500	0.0029	0.3769	66.1971	0.0007
353	900	0.0028	0.9359	66.4378	0.0004
363	1900	0.0028	1.6552	66.6785	0.0002
373	4000	0.0027	2.3725	66.9192	0.0001

[a] The experimental spectrum at 298 K was not simulated.

Table S5 – Kinetics data and Eyring analysis of **1d** in *d*$_8$-toluene. Rate constants for enantiomerization were determined from the exchange of all four ethylene bridge protons.

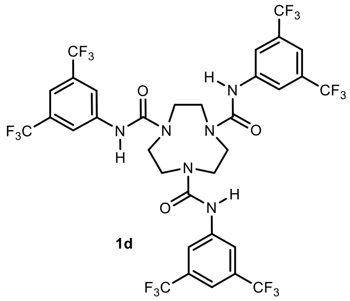

$\Delta G^{\ddagger}_{298\ K}$ = 67.5 kJ mol^{-1}

(*d*$_8$-Toluene)

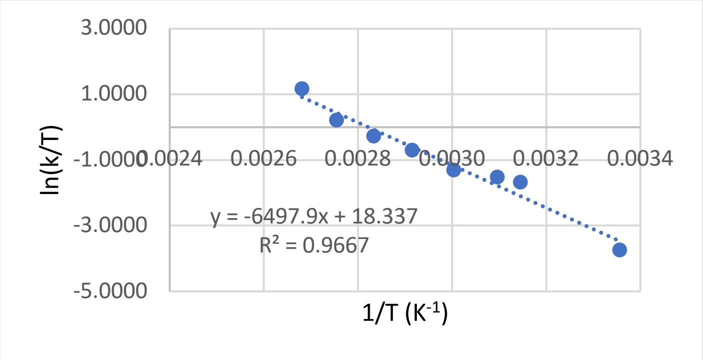

Temperature (K)	Rate Constant, *k* (s^{-1})	1/T (K^{-1})	ln(*k*/T)	$\Delta G^{\ddagger}$ (kJ mol^{-1})	t$_{½}$ (s)
298	7	0.0034	-3.7512	67.4631	0.0495
318	60	0.0031	-1.6677	68.3649	0.0058
323	70	0.0031	-1.5292	68.5903	0.0050
333	90	0.0030	-1.3083	69.0412	0.0039
343	170	0.0029	-0.7019	69.4921	0.0020
353	270	0.0028	-0.2680	69.9430	0.0013
363	450	0.0028	0.2148	70.3939	0.0008
373	1200	0.0027	1.1685	70.8448	0.0003

Table S6 – Kinetics data and Eyring analysis of **1d** in d_2-TCE. Rate constants for enantiomerization were determined from the exchange of all four ethylene bridge protons.

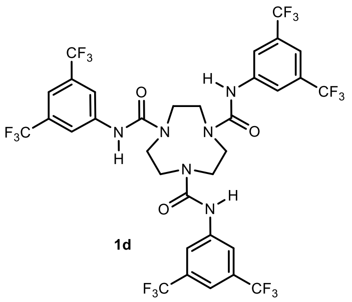

1d

$\Delta G^{\ddagger}_{298\,K}$ = 70.0 kJ mol^{-1}

(d_2-TCE)

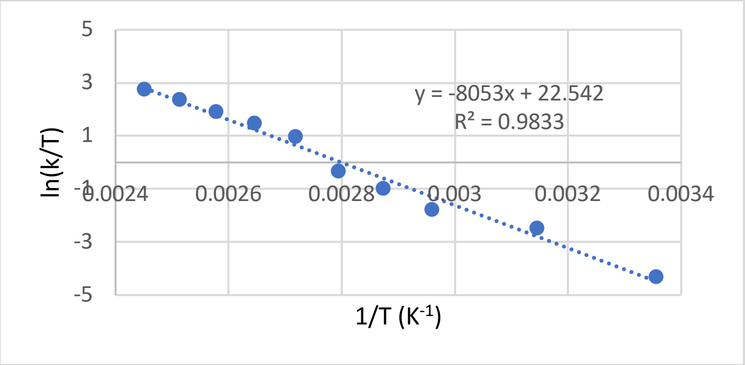

**Temperature (K)**	**Rate Constant, *k* (s^{-1})**	**1/T (K^{-1})**	**ln(*k*/T)**	**ΔG‡ (kJ mol^{-1})**	**t$_{½}$ (s)**
298	4	0.0034	-4.3108	69.9742	0.0866
318	27	0.0031	-2.4662	70.1767	0.0128
338	58	0.0030	-1.7626	70.3792	0.0060
348	132	0.0029	-0.9694	70.4805	0.0026
358	260	0.0028	-0.3199	70.5818	0.0013
368	970	0.0027	0.9692	70.6830	0.0004
378	1680	0.0026	1.4917	70.7843	0.0002
388	2650	0.0026	1.9213	70.8856	0.0001
398	4300	0.0025	2.3799	70.9869	0.0001
408	6500	0.0025	2.7683	71.0881	0.0001

Table S7 – Kinetics data and Eyring analysis of **1e** in d_2-TCE. Rate constants for enantiomerization were determined from the exchange of all four ethylene bridge protons.

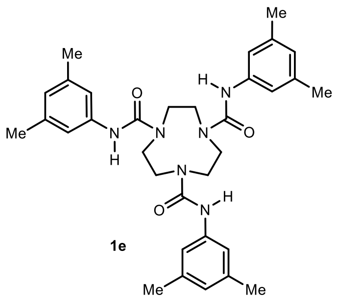

1e

$\Delta G^{\ddagger}_{298\,K}$ = 64.4 kJ mol^{-1}

(d_2-TCE)

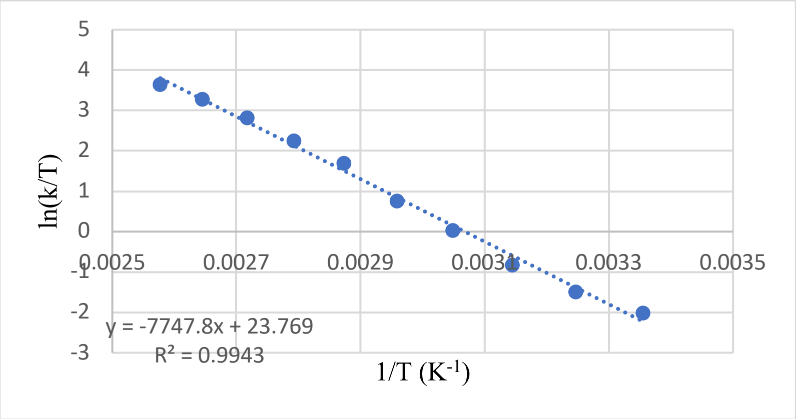

**Temperature (K)**	**Rate Constant, *k* (s^{-1})**	**1/T (K^{-1})**	**ln(*k*/T)**	**ΔG‡ (kJ mol^{-1})**	**t$_{½}$ (s)**
298	40	0.003356	-2.00821	64.39644	0.008664
308	70	0.003247	-1.4816	64.39569	0.004951
318	140	0.003145	-0.82041	64.39494	0.002476
328	340	0.003049	0.035932	64.39419	0.001019
338	730	0.002959	0.769999	64.39345	0.000475
348	1900	0.002874	1.697407	64.3927	0.000182
358	3400	0.002793	2.250998	64.39195	0.000102
368	6200	0.002717	2.824222	64.3912	5.59E-05
378	10000	0.002646	3.275446	64.39045	3.47E-05
388	15000	0.002577	3.6548	64.3897	2.31E-05

Table S8 – Kinetics data and Eyring analysis of **1f** in d_2-TCE. Rate constants for enantiomerization were determined from the exchange of all four ethylene bridge protons.

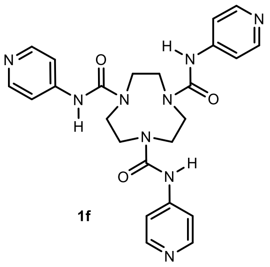

1f

$\Delta G^{\ddagger}_{298\,K}$ = 64.5 kJ mol^{-1}

(d_2-TCE)

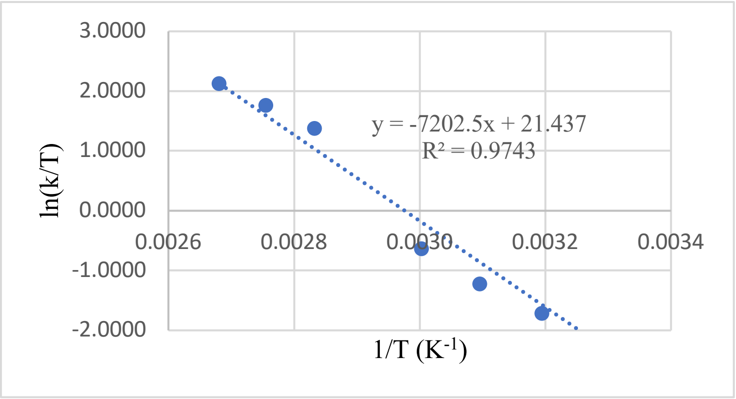

Temperature (K)	Rate Constant, k (s^{-1})	1/T (K^{-1})	ln(k/T)	ΔG‡ (kJ mol^{-1})	$t_{½}$ (s)
298	26	0.0034	-2.4390	64.4869	0.0133
303	35	0.0033	-2.1584	64.5003	0.0099
313	56	0.0032	-1.7209	64.5271	0.0062
323	95	0.0031	-1.2238	64.5539	0.0036
333	175	0.0030	-0.6434	64.5807	0.0020
353	1390	0.0028	1.3706	64.6342	0.0002
363	2100	0.0028	1.7553	64.6610	0.0002
373	3100	0.0027	2.1176	64.6878	0.0001

Table S9 – Kinetics data and Eyring analysis of **1g** in $CDCl_3$. Rate constants for enantiomerization were determined from the exchange of all four ethylene bridge protons.

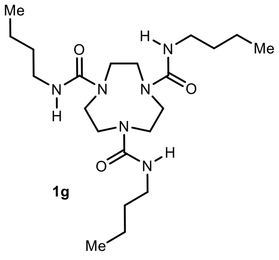

$\Delta G^{\ddagger}_{298\,K}$ = 49.0 kJ mol^{-1}

($CDCl_3$)

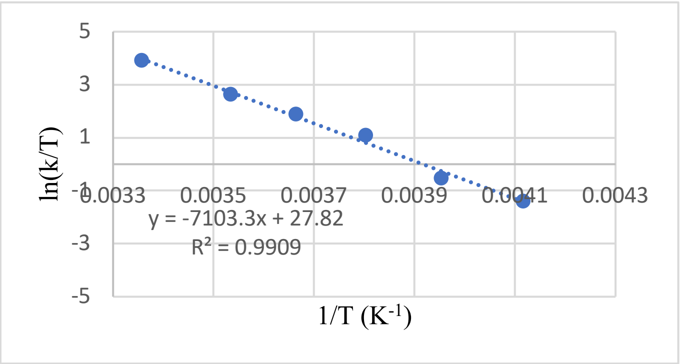

Temperature (K)	Rate Constant, *k* (s^{-1})	1/T (K^{-1})	ln(*k*/T)	$\Delta G^{\ddagger}$ (kJ mol^{-1})	$t_{½}$ (s)
243	60	0.004115	-1.39872	50.8572	0.005776
253	150	0.003953	-0.52275	50.51963	0.00231
263	800	0.003802	1.112458	50.18206	0.000433
273	1800	0.003663	1.88607	49.84449	0.000193
283	4000	0.003534	2.648603	49.50693	8.66E-05
298	15000	0.003356	3.918712	49.00057	2.31E-05

Table S10 – Kinetics data and Eyring analysis of **2a** in d_2-TCE. Rate constants for enantiomerization were determined from the exchange of all four ethylene bridge protons.

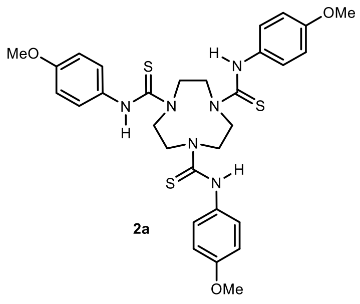

$\Delta G^{\ddagger}_{298\ K}$ = 62.4 kJ mol^{-1}

(d_2-TCE)

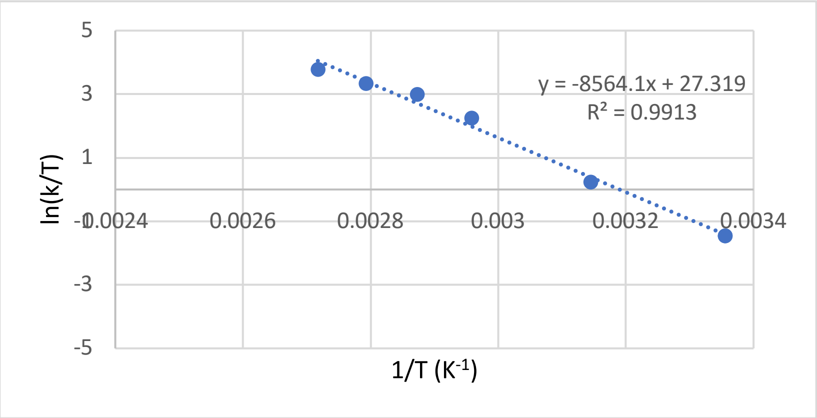

**Temperature (K)**	**Rate Constant, *k* (s^{-1})**	**1/T (K^{-1})**	**ln(*k*/T)**	**ΔG‡ (kJ mol^{-1})**	**t$_{½}$ (s)**
298	70	0.003356	-1.4486	62.38767	0.004951
318	400	0.003356	-1.4486	62.38767	0.004951
328	1400	0.003145	0.229413	61.79584	0.000866
338	3200	0.002959	2.24786	61.20402	0.000108
348	7000	0.002874	3.001463	60.90811	0.000050
358	10000	0.002793	3.329807	60.61219	0.000035
368	16000	0.002717	3.772261	60.31628	0.000022

Table S11 – Kinetics data and Eyring analysis of **2b** in d_2-TCE. Rate constants for enantiomerization were determined from the exchange of all four ethylene bridge protons.

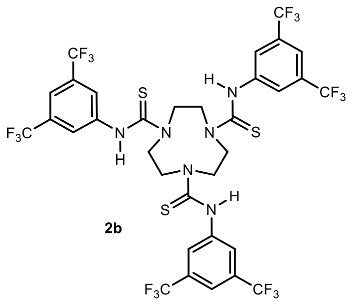

2b

$\Delta G^{\ddagger}_{298\ K} = 70.5\ kJ\ mol^{-1}$

(d_2-TCE)

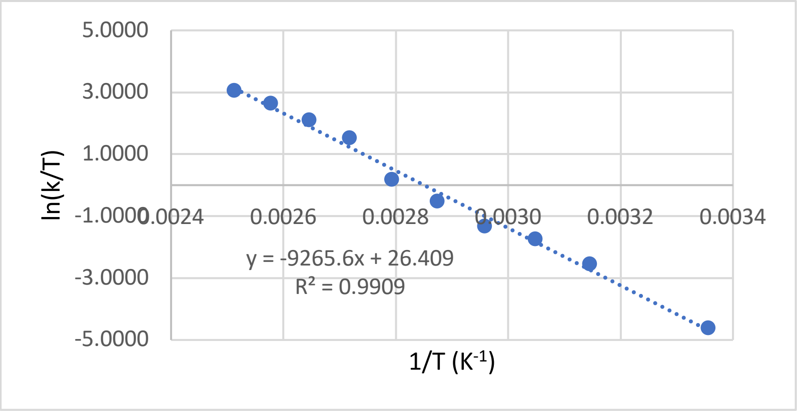

Temperature (K)	Rate Constant, *k* (s^{-1})	1/T (K^{-1})	ln(*k*/T)	$\Delta G^{\ddagger}$ (kJ mol^{-1})	$t_{1/2}$ (s)
298	3	0.0034	-4.5985	70.4750	0.11552
318	25	0.0031	-2.5432	70.0345	0.01386
328	58	0.0030	-1.7326	69.8142	0.00598
338	90	0.0030	-1.3232	69.5940	0.00385
348	210	0.0029	-0.5051	69.3737	0.00165
358	430	0.0028	0.1833	69.1535	0.00081
368	1700	0.0027	1.5303	68.9332	0.00020
378	3100	0.0026	2.1043	68.7130	0.00011
388	5500	0.0026	2.6515	68.4927	0.00006
398	8600	0.0025	3.0731	68.2725	0.00004

Table S12 – Kinetics data and Eyring analysis of **3** in d_2-TCE. Rate constants for enantiomerization by directionality reversal were determined from the exchange of ethylene bridge protons.

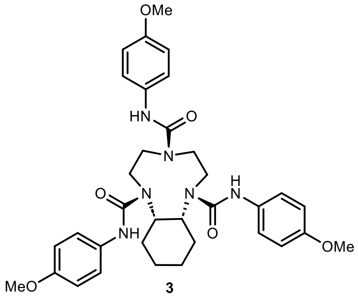

3

$\Delta G^{\ddagger}_{298\,K}$ = 69.8 kJ mol^{-1}

(d_2-TCE)

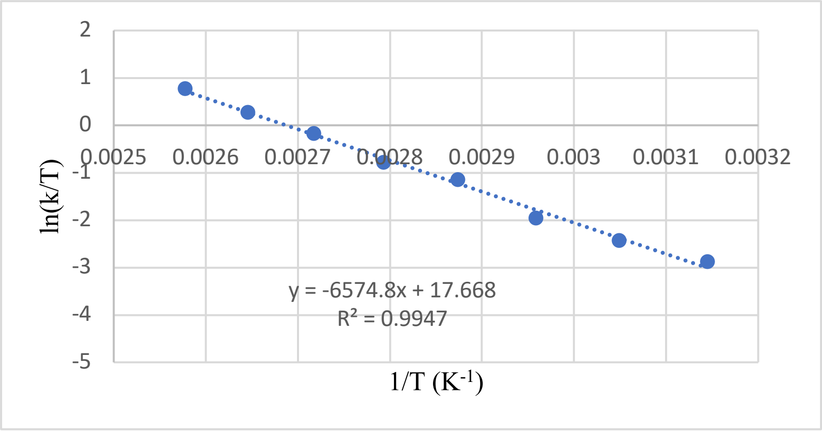

Temperature (K)	Rate Constant, k (s^{-1})	1/T (K^{-1})	ln(k/T)	$\Delta G^{\ddagger}$ (kJ mol^{-1})	$t_{½}$ (s)
298	12	0.003356	-3.21219	69.76008	0.028881
318	18	0.003145	-2.87168	70.77312	0.019254
328	29	0.003049	-2.42572	71.27963	0.011951
338	48	0.002959	-1.95184	71.78615	0.00722
348	110	0.002874	-1.15172	72.29266	0.003151
358	165	0.002793	-0.77459	72.79918	0.0021
368	310	0.002717	-0.17151	73.3057	0.001118
378	500	0.002646	0.279714	73.81221	0.000693
388	840	0.002577	0.772397	74.31873	0.000413

Table S13 – Kinetics data and Eyring analysis of **5** in d_2-TCE. Rate constants for enantiomerization by directionality reversal were determined from the exchange of the BTMP thiourea NH resonances.

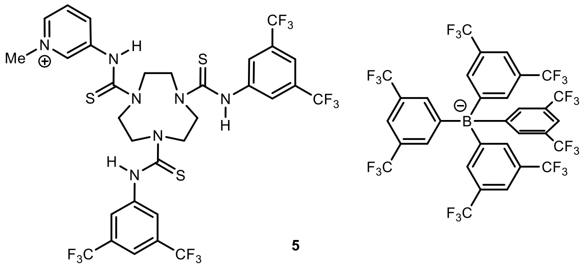

5

$\Delta G^{\ddagger}_{298\,K}$ = 66.1 kJ mol^{-1}

(d_2-TCE)

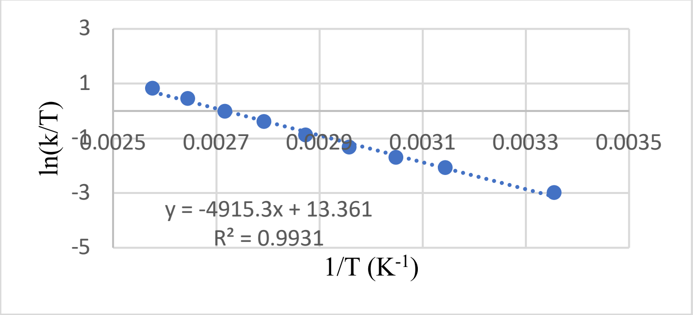

Temperature (K)	Rate Constant, k (s^{-1})	1/T (K^{-1})	ln(k/T)	ΔG‡ (kJ mol^{-1})	t$_{½}$ (s)
298	15	0.003356	-2.98904	66.14314	0.023105
318	40	0.003145	-2.07317	68.03069	0.008664
328	60	0.003049	-1.69867	68.97446	0.005776
338	90	0.002959	-1.32324	69.91824	0.003851
348	145	0.002874	-0.87547	70.86201	0.00239
358	240	0.002793	-0.39989	71.80578	0.001444
368	360	0.002717	-0.02198	72.74956	0.000963
378	590	0.002646	0.445228	73.69333	0.000587
388	880	0.002577	0.818917	74.6371	0.000394

Table S14 – Kinetics data and Eyring analysis of **6** in d_2-TCE. Rate constants for enantiomerization by directionality reversal were determined from the exchange of the BTMP *ortho* resonances.

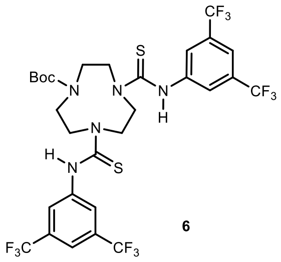

6

$\Delta G^{\ddagger}_{298\ K}$ = 66.2 kJ mol^{-1}

(d_2-TCE)

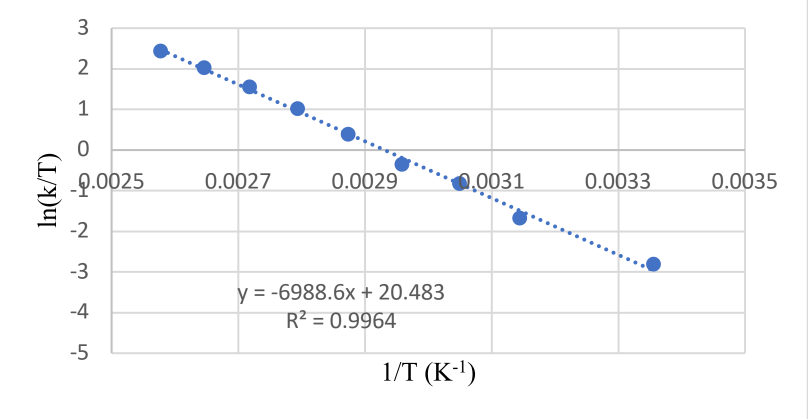

Temperature (K)	Rate Constant, *k* (s^{-1})	1/T (K^{-1})	ln(*k*/T)	$\Delta G^{\ddagger}$ (kJ mol^{-1})	$t_{½}$ (s)
298	18	0.003356	-2.80672	66.22586	0.019254
318	60	0.003145	-1.66771	66.77078	0.005776
328	145	0.003049	-0.81628	67.04325	0.00239
338	240	0.002959	-0.34241	67.31571	0.001444
348	520	0.002874	0.401626	67.58818	0.000666
358	1000	0.002793	1.027222	67.86064	0.000347
368	1750	0.002717	1.559288	68.1331	0.000198
378	2900	0.002646	2.037572	68.40557	0.00012
388	4500	0.002577	2.450827	68.67803	7.7E-05

Table S15 – Kinetics data and Eyring analysis of **7** in d_2-TCE. Rate constants for enantiomerization by bowl inversion were determined from the exchange of geminal ethylene bridge protons.

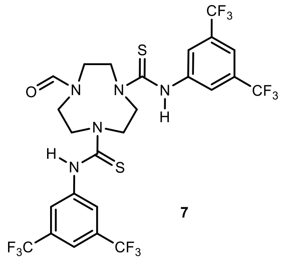

7

$\Delta G^{\ddagger}_{298\ K}$ = 56.5 kJ mol^{-1}

(d_2-TCE)

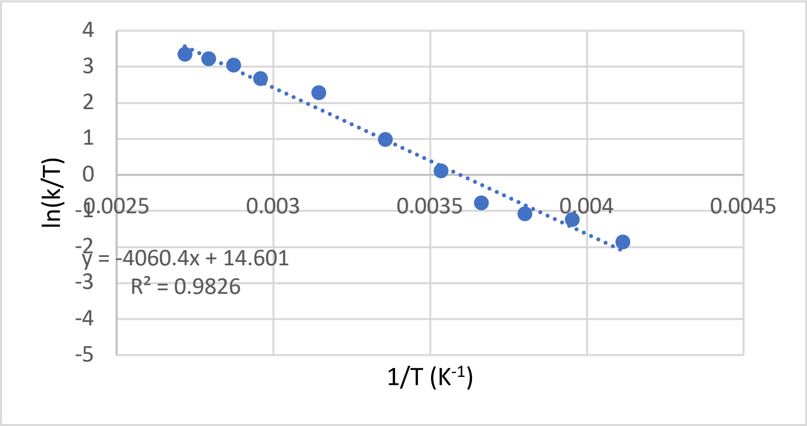

Temperature (K)	Rate Constant, k (s^{-1})	1/T (K^{-1})	ln(k/T)	$\Delta G^{\ddagger}$ (kJ mol^{-1})	$t_{1/2}$ (s)
243	38	0.004115	-1.85548	52.26498	0.00912
253	73	0.003953	-1.24293	53.0265	0.004748
263	90	0.003802	-1.07234	53.78802	0.003851
273	125	0.003663	-0.78116	54.54954	0.002773
283	320	0.003534	0.122874	55.31106	0.001083
298	800	0.003356	0.987518	56.45334	0.000433
318	3100	0.003145	2.277106	57.97638	0.000112
338	4900	0.002959	2.673945	59.49942	7.07E-05
348	7400	0.002874	3.057033	60.26094	4.68E-05
358	9000	0.002793	3.224447	61.02246	3.85E-05
368	10500	0.002717	3.351048	61.78398	3.3E-05

Table S16 – Summary of enantiomerization barriers and their enthalpic and entropic contributions for compounds **1**–**3** and **5**–**7**, as determined by ^{1}H NMR lineshape and Eyring analysis.

**Compound**	**Solvent**	**$\Delta G^{\ddagger}_{298\,K}$** (kJ mol^{-1})	**$\Delta H^{\ddagger}$** (kJ mol^{-1})	**$\Delta S^{\ddagger}$** (J mol^{-1} K^{-1})
**1a**	d_8-toluene	65.33^a	50.33	−50.36
**1a**	d_2-TCE	63.17^a	61.26	−6.41
**1b**	d_8-toluene	63.18^a	51.58	−38.93
**1c**	d_8-toluene	65.11^a	57.94	−24.07
**1d**	d_8-toluene	67.46^a	54.03	−45.09
**1d**	d_2-TCE	69.97^a	66.96	−10.13
**1e**	d_2-TCE	64.40^a	64.42	0.07
**1f**	d_2-TCE	64.49^a	59.88	−5.76
**1g**	$CDCl_3$	49.00^a	59.06	33.76
**2a**	d_2-TCE	62.39^a	71.21	25.59
**2b**	d_2-TCE	70.48^a	77.04	22.03
**3**	d_2-TCE	69.76^b	54.67	−50.65
**5**	d_2-TCE	66.13^b	40.87	−86.46
**6**	d_2-TCE	66.23^b	58.11	−27.25
**7**	d_2-TCE	56.45^c	33.76	−76.15

^{a} Directionality reversal and bowl inversion not distinguished. ^{b} Discrete directionality reversal barrier. ^{c} Discrete bowl inversion barrier.

NMR Studies of **1d** in $CDCl_3$

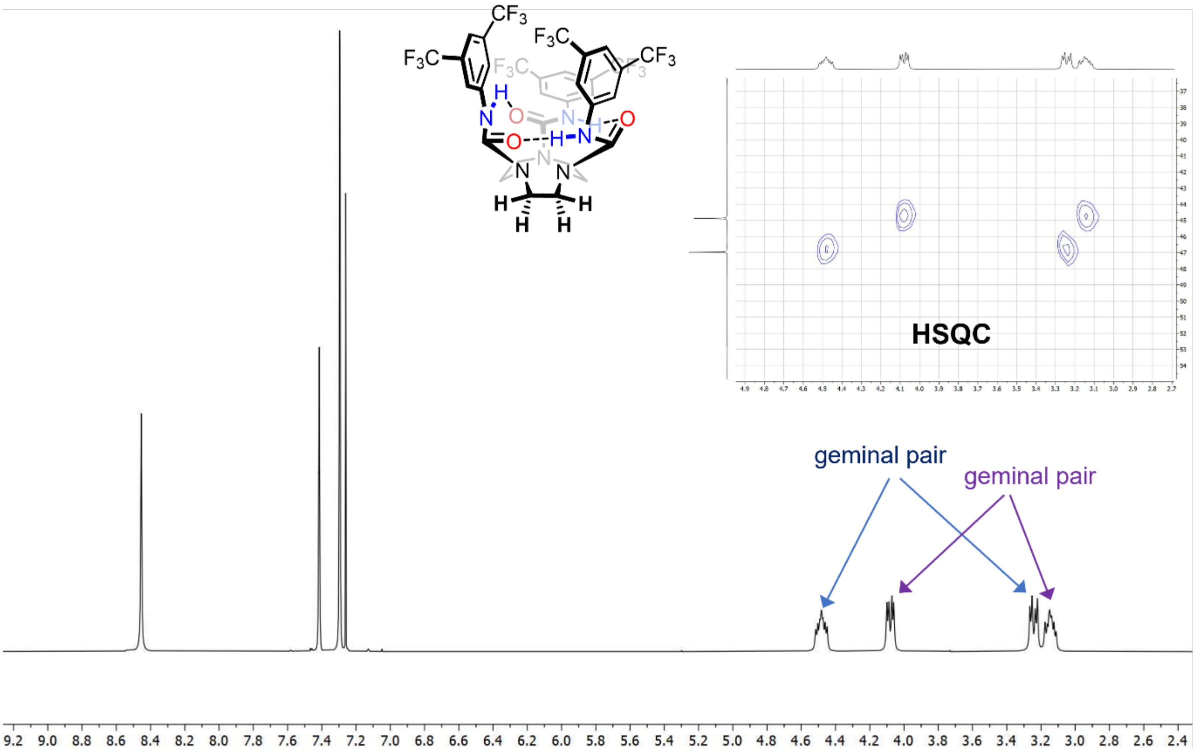

Figure S16 – ^{1}H NMR spectrum of **1d** (25 °C, 15 mM, $CDCl_3$, 500 MHz) and a portion of the HSQC spectrum (in $CDCl_3$) showing carbon correlations of the ethylene bridge protons. Geminal ethylene bridge proton pairs are labelled on the ^{1}H NMR spectrum.

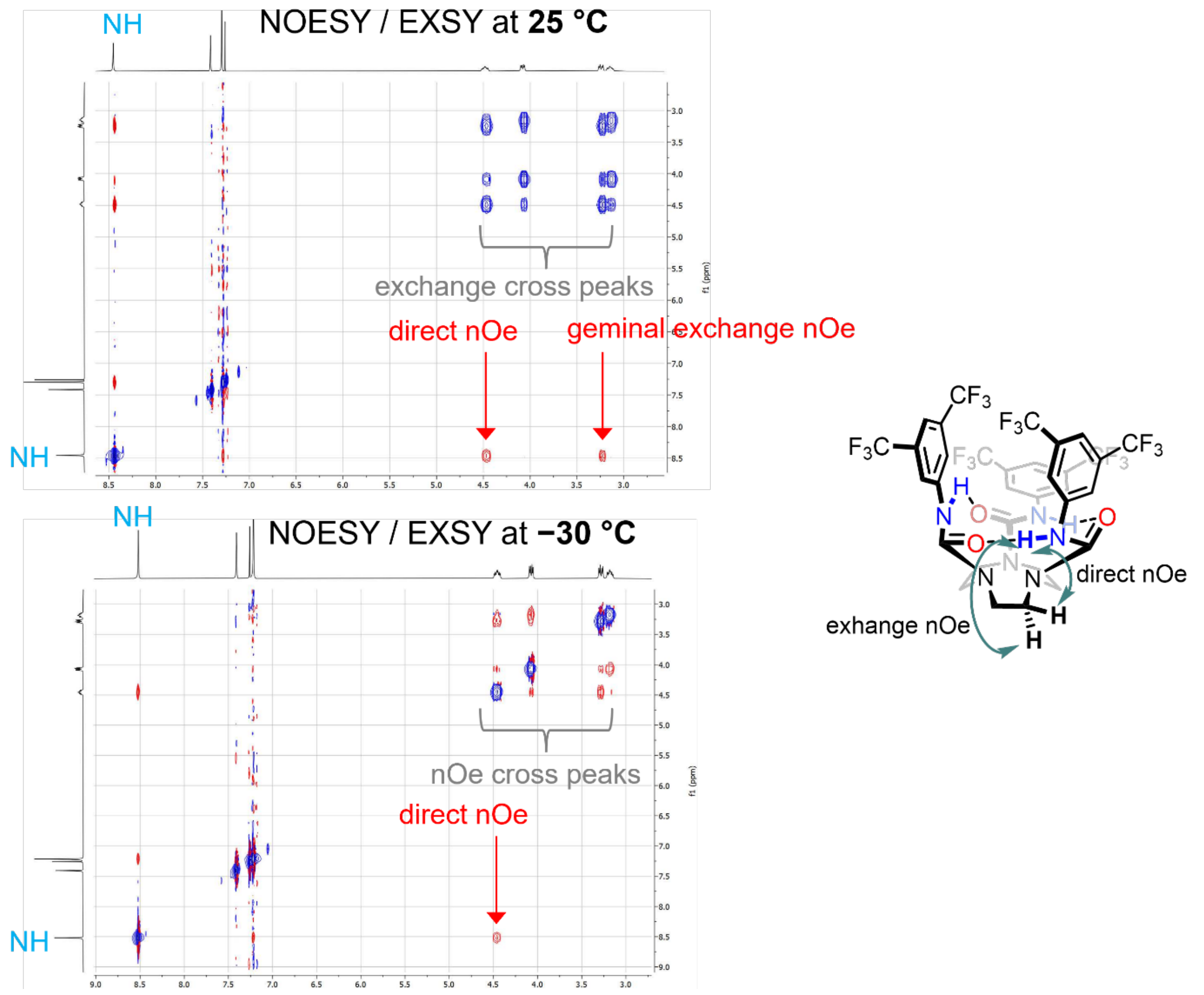

Figure S17 – NOESY / EXSY spectra of **1d** (15 mM, $CDCl_3$, 500 MHz) at 25 °C (top) and −30 °C (bottom). A geminal exchange nOe with the NH is observed at 25 °C (but not vicinal exchange nOe's) because bowl inversion is faster than directionality reversal. Exchange nOe's are 'filtered out' at −30 °C and only the direct nOe's remain. Direct nOe's between the ethylene bridge protons become apparent at −30 °C (red cross peaks) because their exchange by enantiomerization is slowed down significantly.

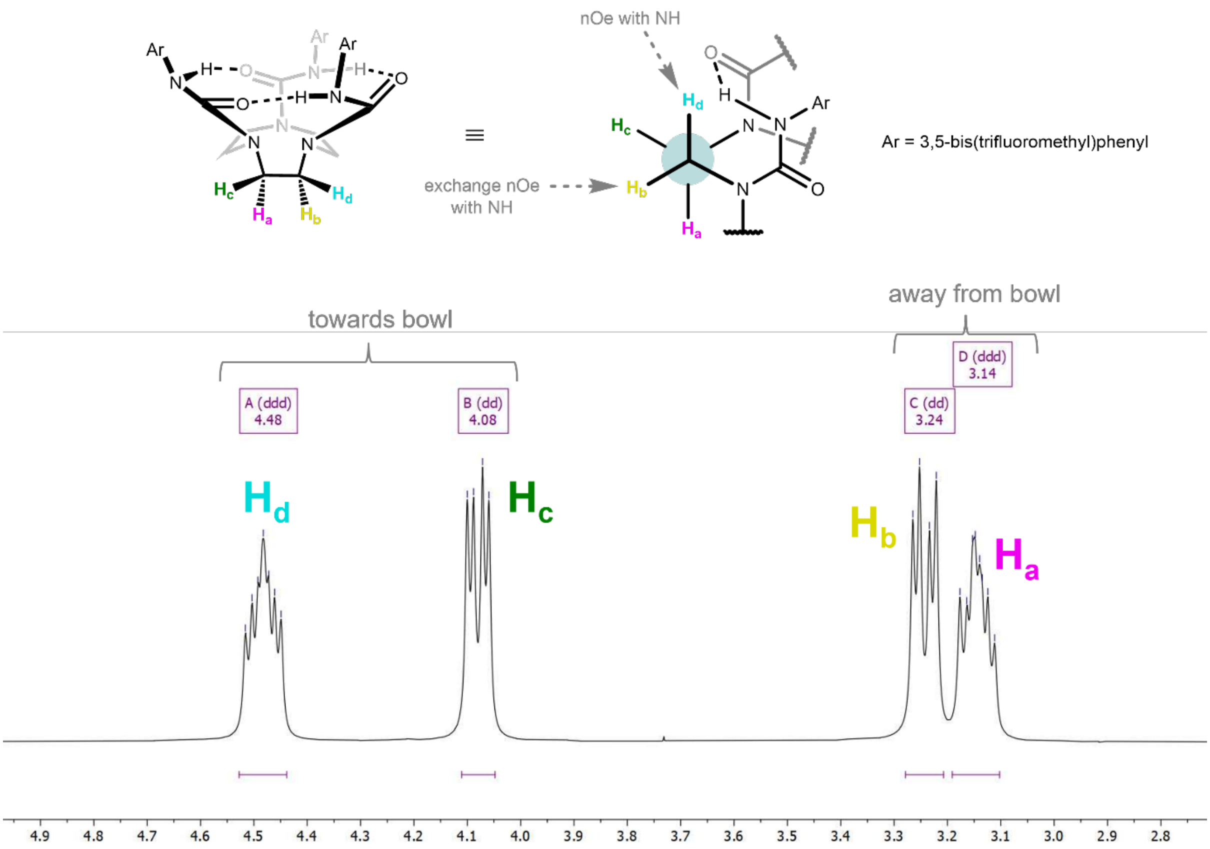

Figure S18 – Portion of the ^{1}H NMR spectrum of **1d** (25 °C, 15 mM, $CDCl_3$, 500 MHz) showing the ethylene bridge proton assignments based on HSQC and NOESY NMR data. The coupling pattern is consistent with a gauche conformation of the ethylene bridge, with protons H_a and H_d occupying the pseudo-axial positions.

EXSY Studies of **1d** in TCE-d_2

For further investigation of the exchange processes in molecule **1d**, we conducted 2D EXSY NMR experiments. These experiments give access to rate constants of all the occurring exchange processes provided sufficient signal separation in the slow-exchange regime.[S7–S9]

The spectra were recorded in TCE-d_2 (500 MHz, 298 K) using noesygpph standard Bruker pulse sequence. Relaxation delay of 5 s was used. The mixing time was set to 300 ms (corresponding to $1/k_{ex}$) and 100 ms, respectively. Due to the short mixing times and molecule size, exchange dominated the spectra and only very weak NOE crosspeak was detected. A reference "zero mixing time" spectrum was recorded with 2.6 ms mixing time. In addition to the dominating diagonal peaks, only weak COSY-type antiphase crosspeaks were detected with net zero integral. Overall, these experiments rule out significant NOE or zero-quantum coherence artifacts.

The data were phased, baseline-corrected and zero-filled to 4 K x 1 K in MestreNova 14.2.1. The spectra were integrated, and the exchange rates calculated by EXSYCalc 1.0 program from Mestrelab Research. Integrals of the diagonal and cross-peaks provided the rate constants.

^{1}H NMR (500 MHz, TCE-d_2) δ 4.45 (ddd, J = 16.8, 11.7, 6.1 Hz, 1H, peak **D**), 4.06 (dd, J = 14.5, 5.9 Hz, 1H, peak **C**), 3.24 (dd, J = 15.7, 6.3 Hz, 1H, peak **B**), 3.17 – 3.07 (m, 1H, peak **A**).

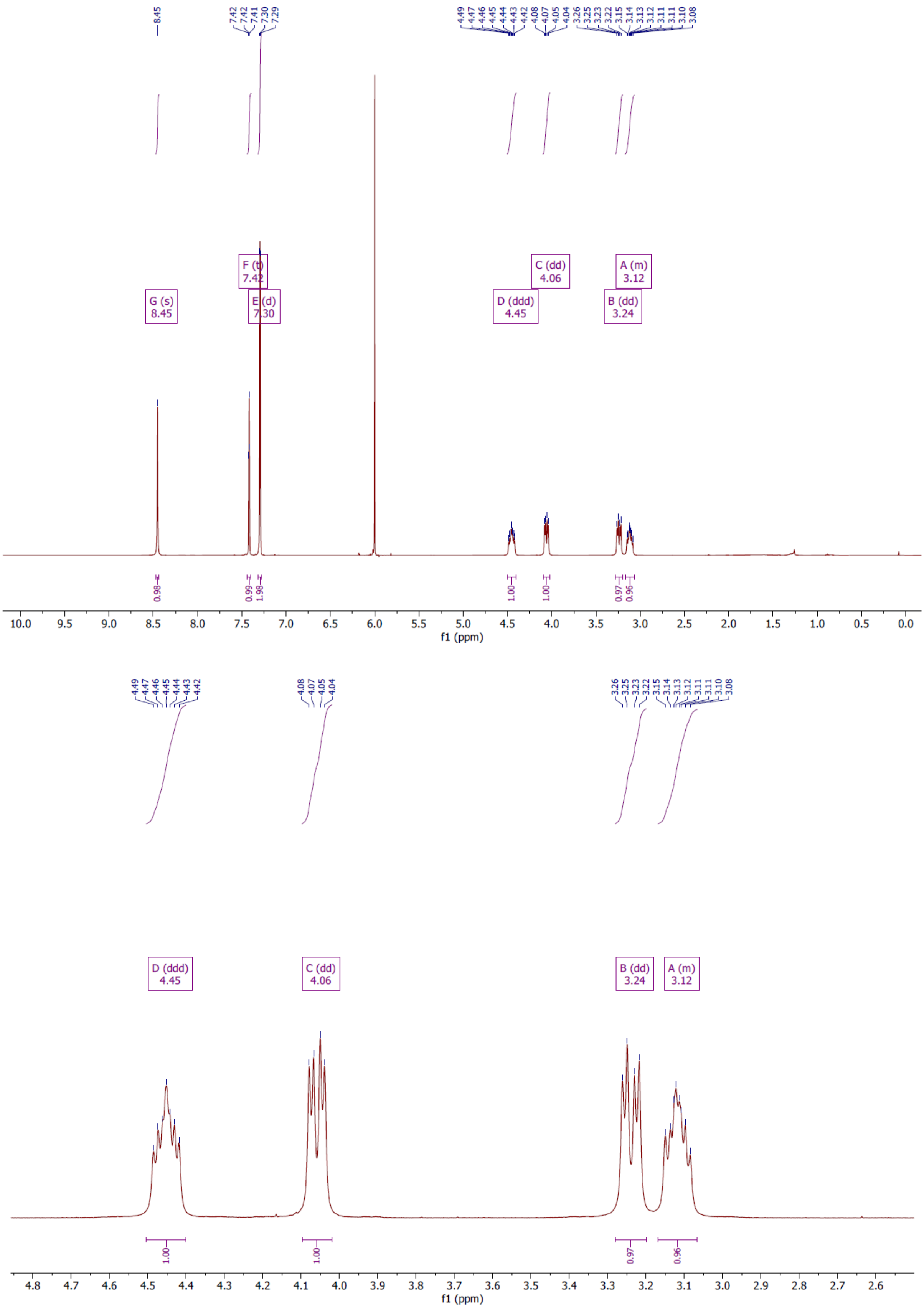

Figure S19 – ^{1}H NMR spectrum (500 MHz, TCE-d_2, 10 mM) of **1d** at 298 K.

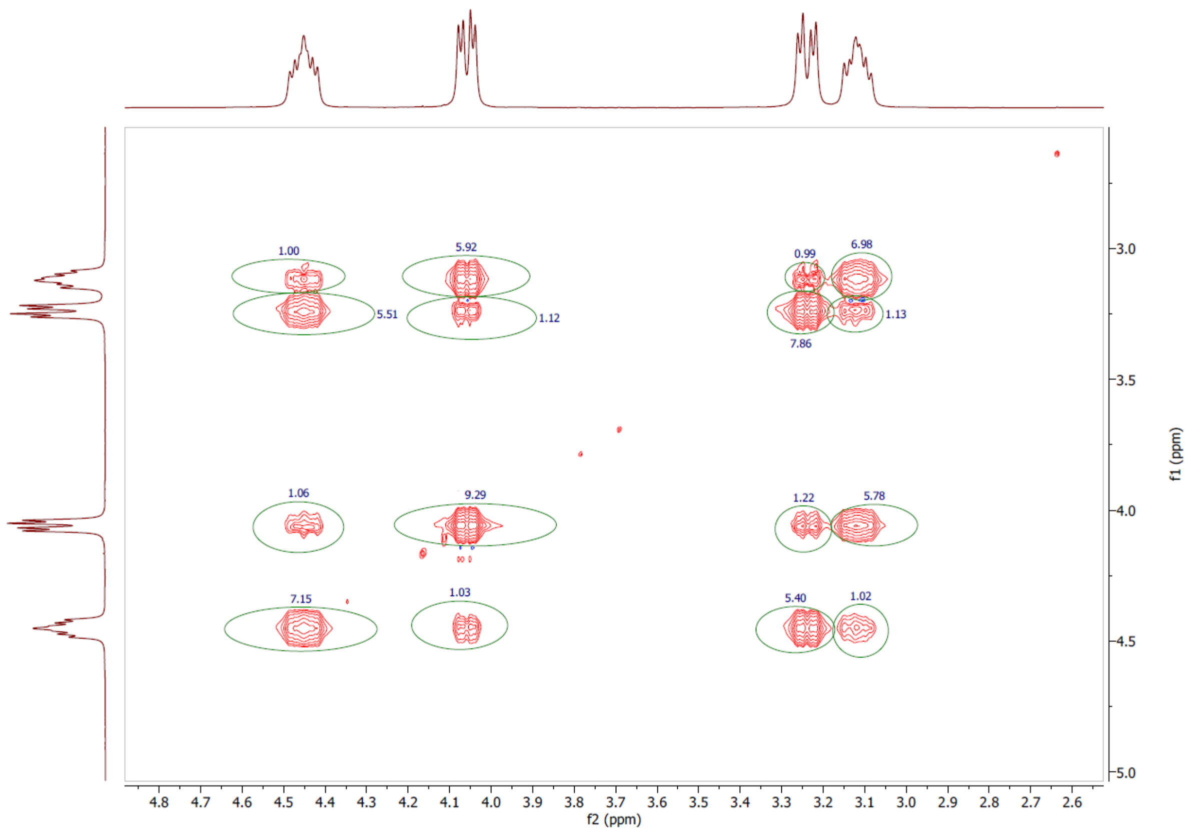

Figure S20 – ^{1}H,^{1}H NOESY spectrum (500 MHz, TCE-d_2, 10 mM) of **1d** at 298 K with 300 ms mixing time.

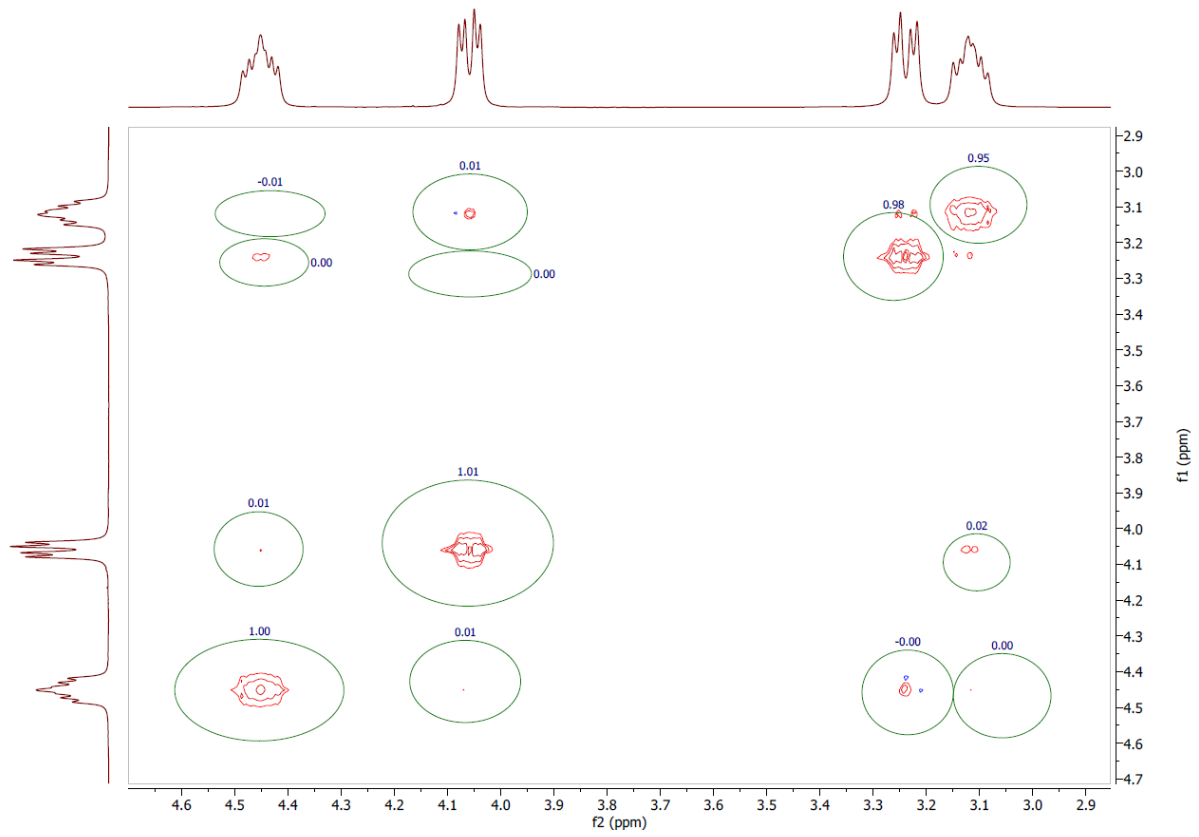

Figure S21 – ^{1}H,^{1}H NOESY spectrum (500 MHz, TCE-d_2, 10 mM) of **1d** at 298 K with 2.6 ms mixing time (d8).

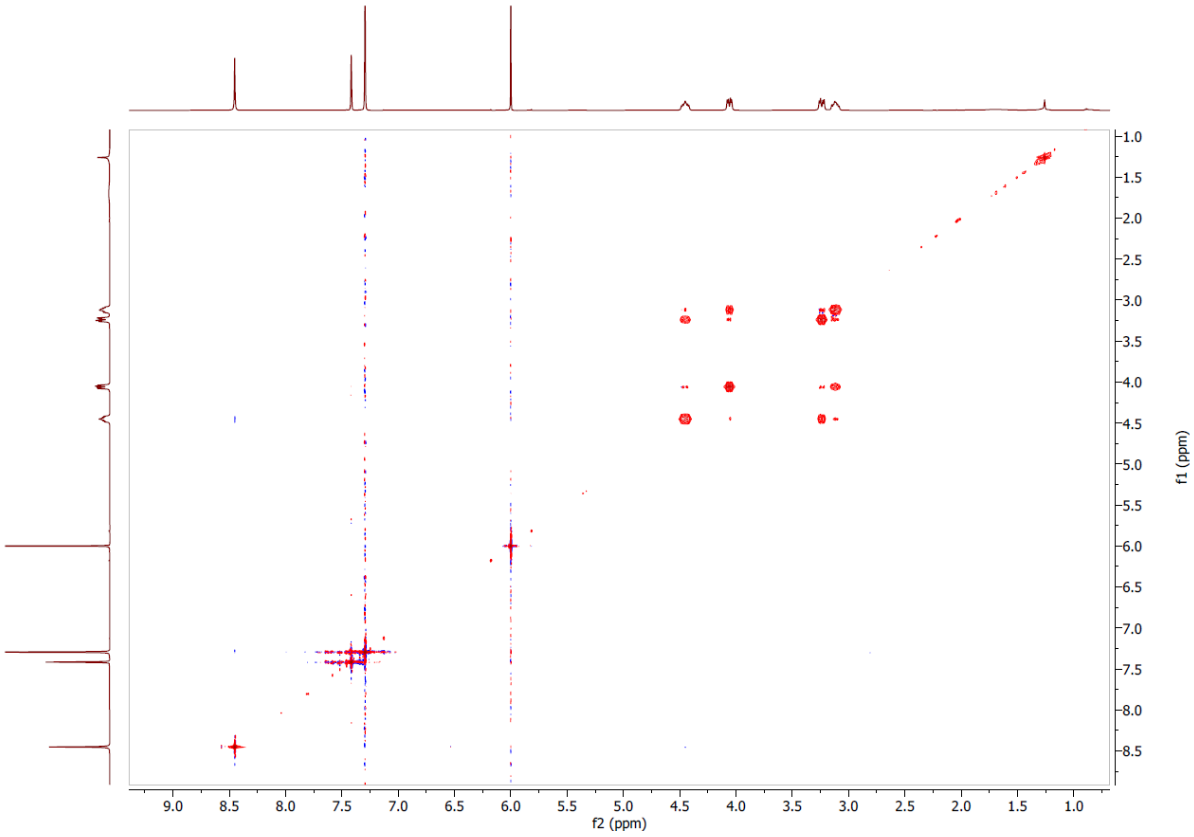

Figure S22 – ^{1}H, ^{1}H NOESY spectrum (500 MHz, TCE-d_2, 10 mM) of **1d** at 298 K with 100 ms mixing time showing the exclusive exchange peaks and only a very weak NOE crosspeak.

Table S17 – Exchange and diagonal peak integrals at 300 ms mixing time.

	**A**	**B**	**C**	**D**
**A**	6503	923	5515	931
**B**	1056	7324	1048	5129
**C**	5381	1132	8655	986
**D**	954	5029	961	6662

Table S18 – Diagonal peak integrals at 2.6 ms mixing time.

**diagonal peak**	**integral**
**AA**	42005
**BB**	43416
**CC**	44669
**DD**	44176

Table S19 – Exchange rate matrix (s^{-1}) extracted from data with 300 ms and 2.6 ms mixing time. The diagonal elements are a combination of exchange rates and longitudinal relaxation rates (e.g. $–R_1$-k_1).

	**A**	**B**	**C**	**D**
**A**	-7.592	0.328	2.907	0.275
**B**	0.211	-7.159	0.336	2.996
**C**	3.178	0.246	-6.636	0.228
**D**	0.321	3.107	0.201	-7.63

Table S20 – Exchange and diagonal peak integrals at 100 ms mixing time (zero-filling 4 K x 2 K).

	**A**	**B**	**C**	**D**
**A**	45081	2114	14604	1825
**B**	2458	49076	1937	14421
**C**	14392	2052	52749	1684
**D**	1949	14307	1573	47614

Table S21 – Diagonal peak integrals at 2.6 ms mixing time (zero-filling 4 K x 2 K; separate experiment).

**diagonal peak**	**integral**
**AA**	93788
**BB**	95940
**CC**	98193
**DD**	98028

Table S22 – Exchange rate matrix (s^{-1}) extracted from data with 100 ms and 2.6 ms mixing time. The diagonal elements are a combination of exchange rates and longitudinal relaxation rates (e.g. $–R_1$-k_1).

	**A**	**B**	**C**	**D**
**A**	-7.819	0.431	2.962	0.315
**B**	0.363	-7.17	0.314	3.01
**C**	3.15	0.288	-6.666	0.222
**D**	0.309	3.099	0.243	-7.699

If we assume the forward and reverse exchange processes in **1d** between A-C and B-D ($k_1^{AC} = k_{-1}^{AC} = k_1^{BD} = k_{-1}^{BD}$) to be equivalent, we can average all the corresponding values to give the rate constants.

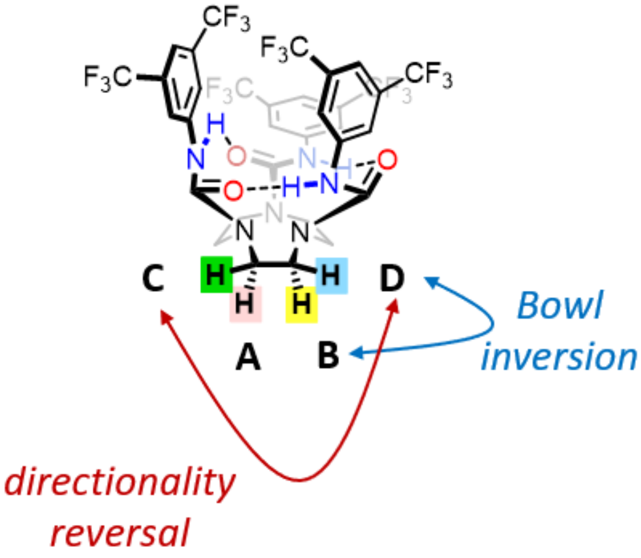

Bowl inversion (processes A-C and B-D)

Directionality reversal (all other processes)

Table S23 – Rate constants (s^{-1}) of bowl inversion and directionality reversal exchange processes of **1d** in different solvents and mixing time at 298 K.

Solvent, mixing time	Bowl inversion (s^{-1})	Directionality reversal (s^{-1})
TCE-d_2, 300 ms	3.05±0.10	0.27±0.05
TCE-d_2, 100 ms	3.06±0.07	0.31±0.05
C_6D_6, 300 ms	3.59±0.26	0.08±0.03

NMR Solvent Studies of **1d**

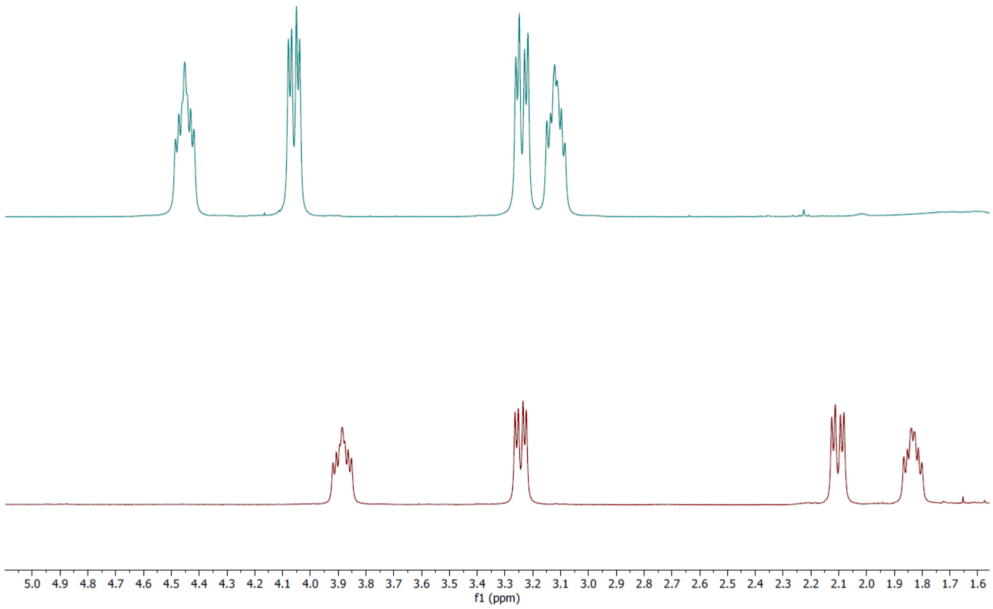

Figure S23 – ^{1}H NMR spectrum (500 MHz, 10 mM) of **1d** at 298 K in TCE-d_2 (top) and C_6D_6 (bottom).

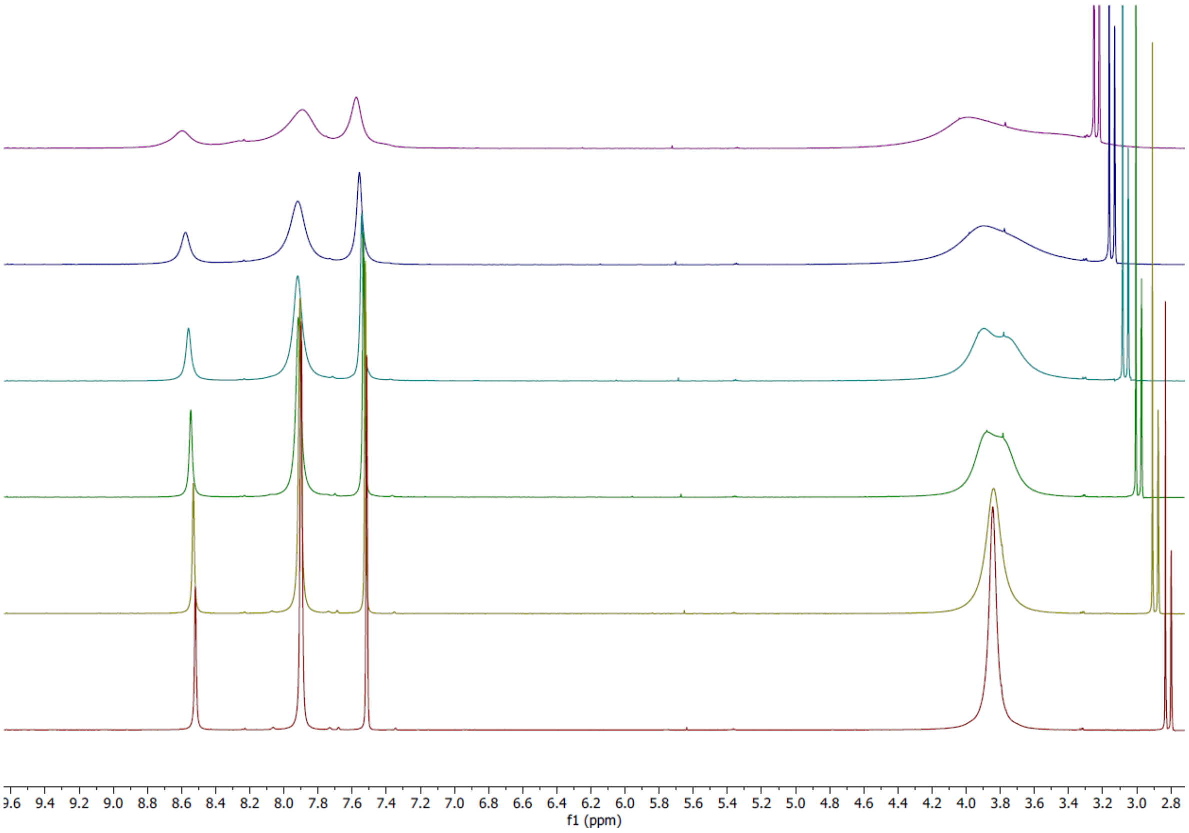

Figure S24 – Variable temperature ^{1}H NMR spectrum (500 MHz, 10 mM) of **1d** in acetone-d_6 between 298 K (bottom) and 248 K (top) in 10 K increments. The hydrogen bond (δ_H 8.50 ppm) remains intact even in a competing solvent such as acetone.

NMR H/D Exchange Kinetics of **1d**

An H/D exchange experiment[S10] was conducted with **1d**. NMR tube was charged with **1d**, dissolved in $CDCl_3$ (0.45 mL, 10 mM), which had been filtered through neutral alumina. CD_3OD (50 µL) from a freshly opened ampoule was added to make 10 % v/v solution, which ensures pseudo-first order kinetics. The sample was loaded into the NMR spectrometer (500 MHz) and the ^1H spectra recorded at 298 K. The decay of the NH signal over time was monitored and fitted to an exponential decay function in Mestrenova. The extracted rate constant was divided by 3 to give *k* due to the three exchanging equivalent NH protons.

Table S24 – Rate constants and half-lives of the H/D exchange of **1d** at different CD_3OD concentrations.

% CD_3OD	k (s^{-1})	$t_{1/2}$ (s)
10	0.0386	17.9
5	0.0040	175.0

The H/D exchange rate is faster than the rates reported for a reported amide compound (see Figure S25 – Half-lives of H/D exchange for a reported compound.[S10]). The protons in the H-bond donating groups exchange slowly than other types of NH. The fast rate of **1d** might potentially be due to the conformational exchange disrupting the H-bond donor/acceptor system.

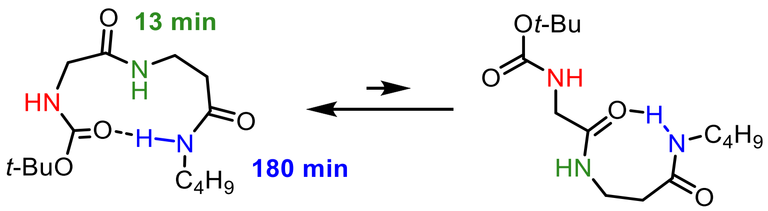

Figure S25 – Half-lives of H/D exchange for a reported compound.[S10]

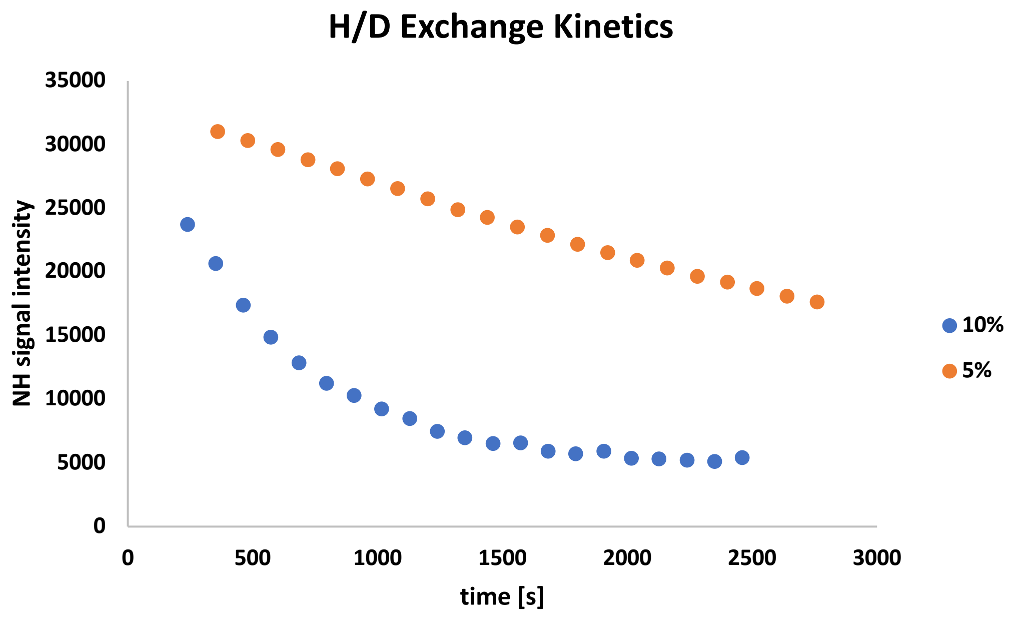

Figure S26 – Decay of NH signal of **1d** due to H/D exchange at different CD_3OD concentrations.

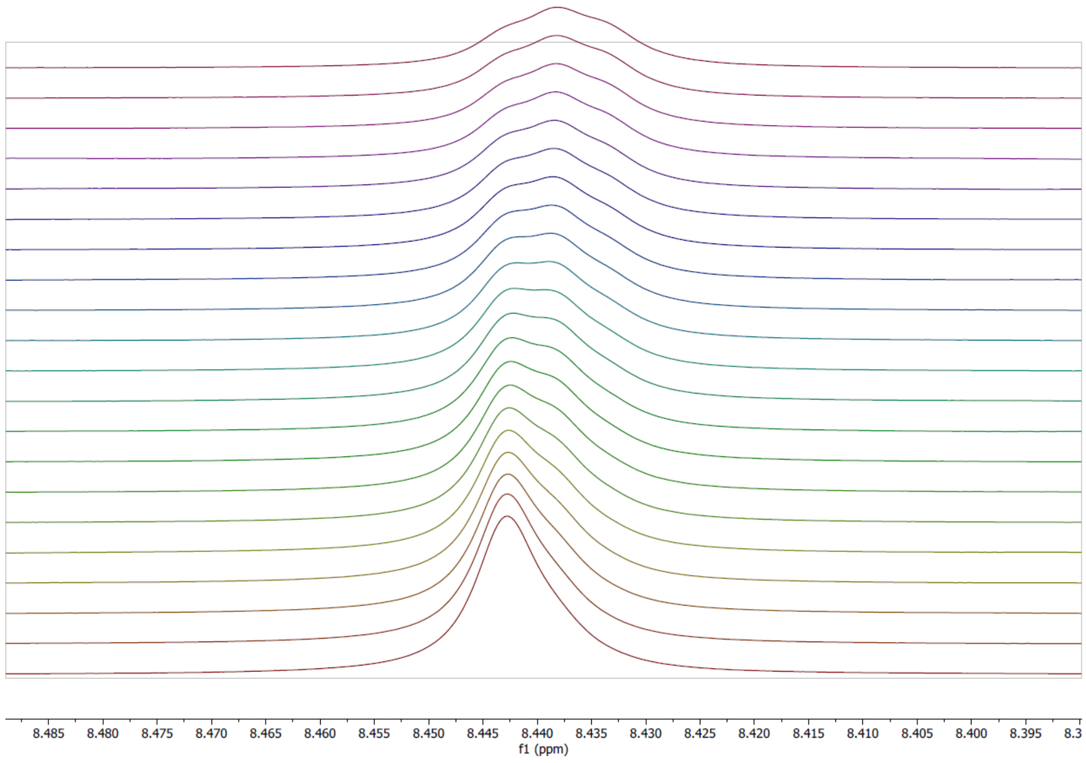

Figure S27 – Stacked ^{1}H NMR spectra (NH signal, 500 MHz, 5 % CD_3OD in $CDCl_3$, 298 K) of **1d** over time (bottom to top). The splitting of the signal is due to the exchanging NHs protons for deuteriums. This process breaks the symmetry of the molecule by producing *HHH*, *HHD* and *HDD* isotopomers.

NMR Studies of **3**

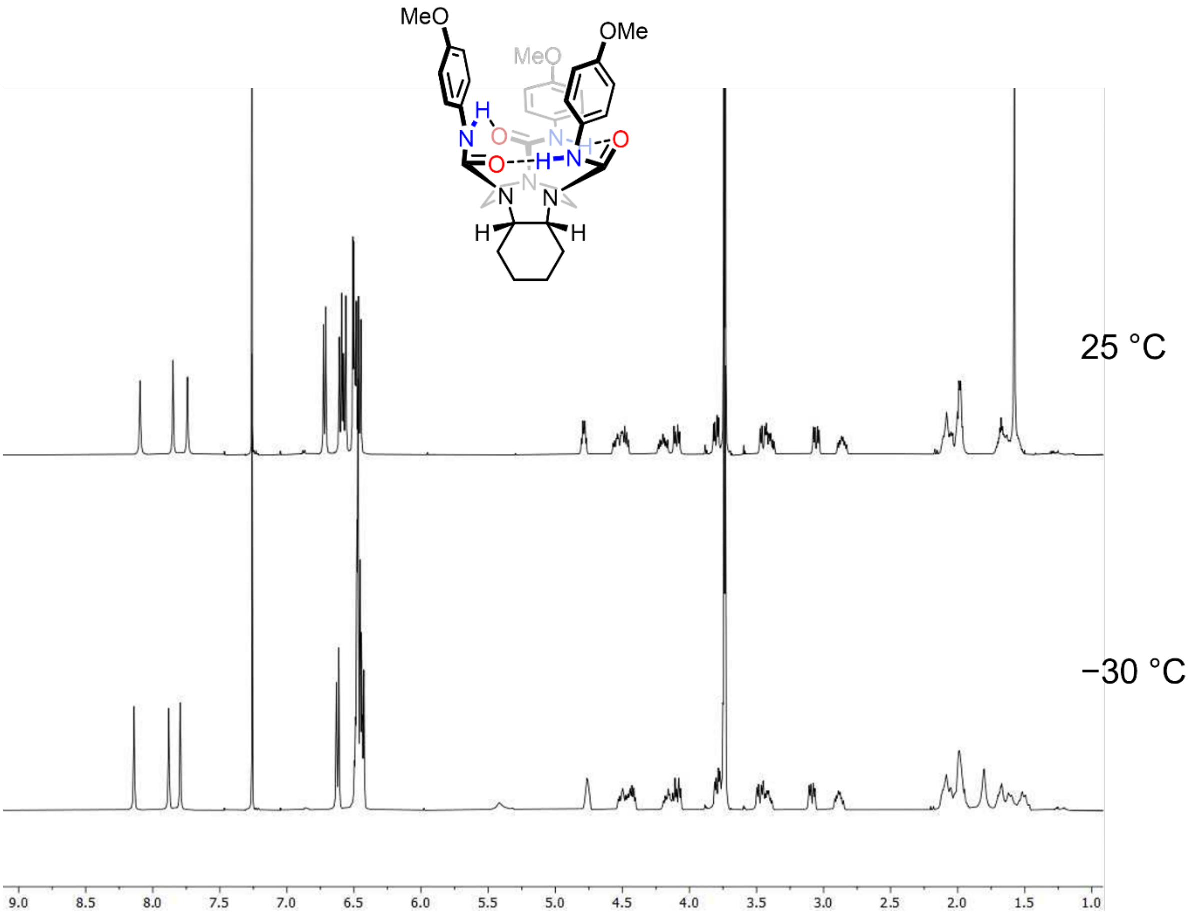

Figure S28 – VT-NMR stack plot for **3** (15 mM, 500 MHz, $CDCl_3$).

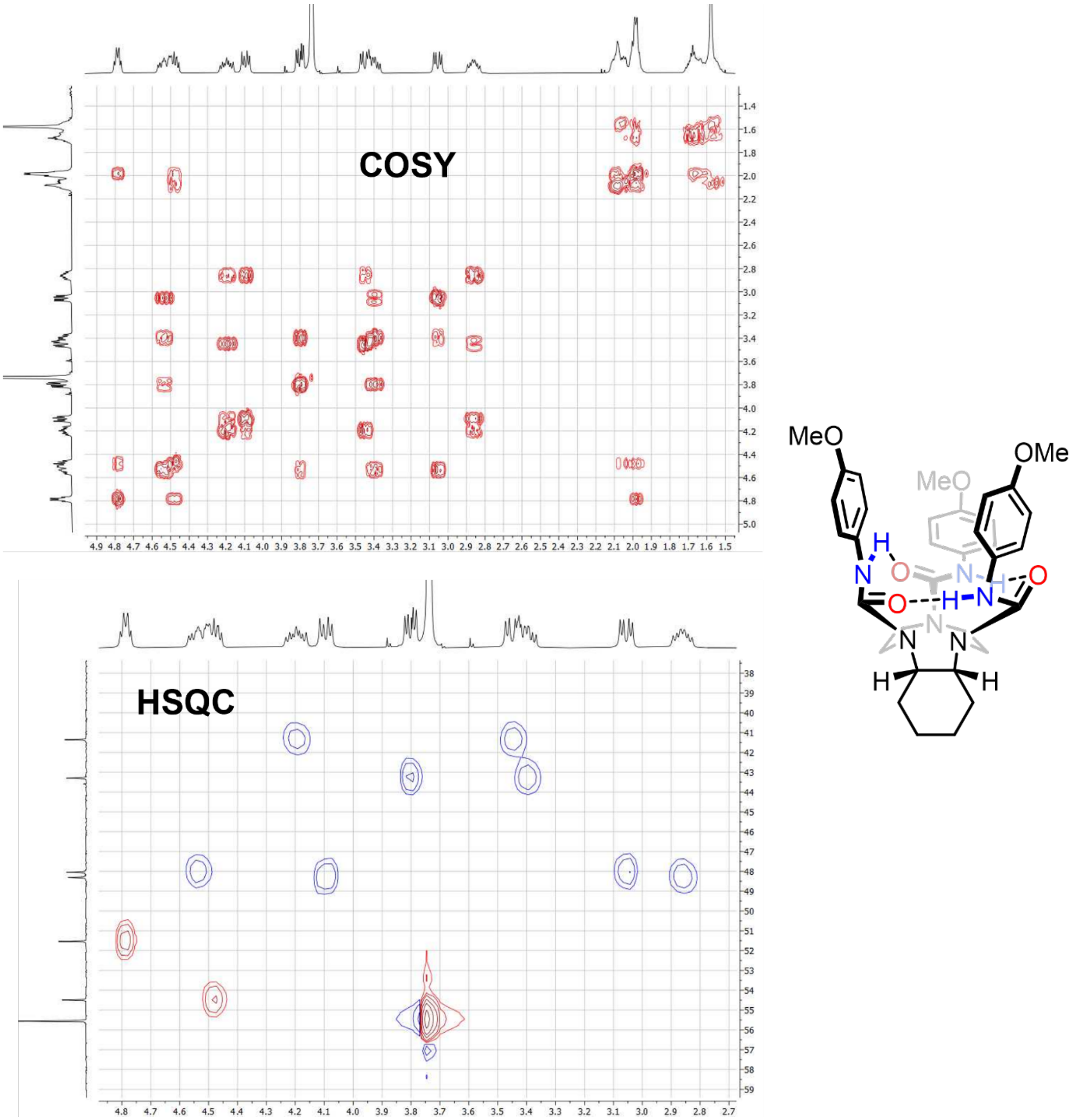

Figure S29 – Portions of the COSY and HSQC spectra of **3** (15 mM, 500 MHz, $CDCl_3$, 25 °C) showing the ethylene bridge and cyclohexyl proton regions (COSY) and carbon correlations of the ethylene bridge protons (HSQC).

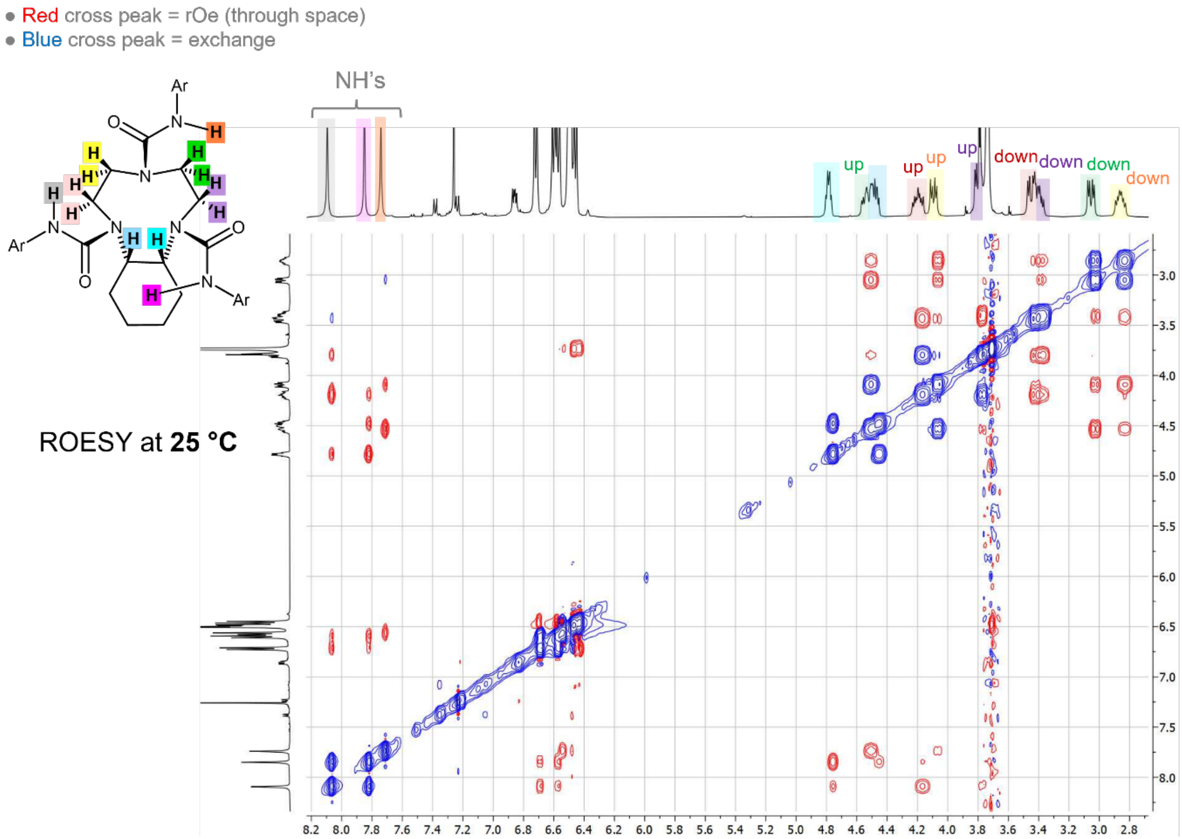

Figure S30 – A portion of the ROESY spectrum of **3** (20 mM, 500 MHz, $CDCl_3$, 25 °C) showing assignments of the TACN ring protons and the NH protons by color (based on COSY, HSQC and ROESY data). Ethylene bridge protons labelled as 'up' are orientated syn to the hydrogen bond network. Ethylene bridge protons labelled as 'down' are orientated anti to the hydrogen bond network. Due to enantiomerization by directionality reversal, rotational exchange cross peaks (blue) are observed, as well as both direct and exchange rOe cross peaks (red).

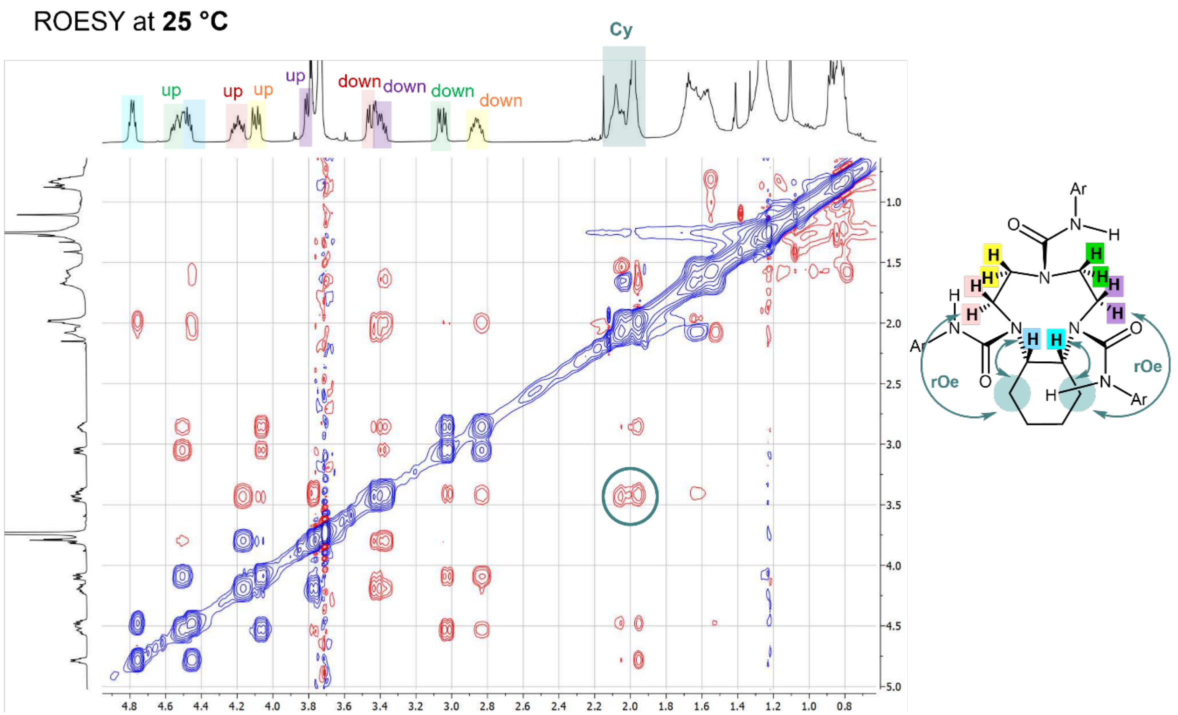

Figure S31 – A portion of the ROESY spectrum of **3** (20 mM, 500 MHz, $CDCl_3$, 25 °C) highlighting the through space rOe cross peaks (circled in green on the spectrum) between the 'pink down' and 'purple down' TACN ring protons with the methylene protons of the cyclohexyl ring. These results confirm that in $CDCl_3$ the cyclohexyl group is disposed anti to the hydrogen bond network (as observed in the crystal structure).

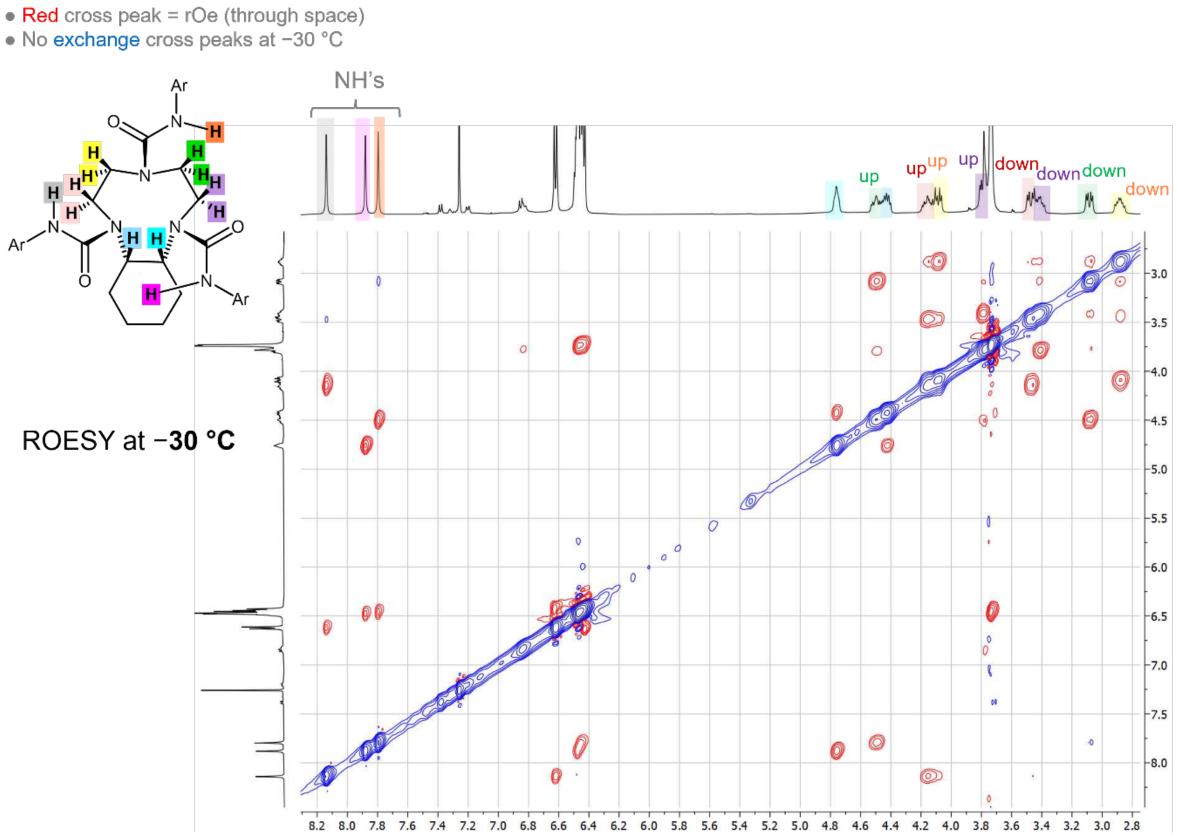

Figure S32 – A portion of the ROESY spectrum of **3** (20 mM, 500 MHz, $CDCl_3$, −30 °C) showing assignments of the TACN ring protons and the NH protons by color (based on COSY, HSQC and ROESY data). Ethylene bridge protons labelled as 'up' are orientated syn to the hydrogen bond network. Ethylene bridge protons labelled as 'down' are orientated anti to the hydrogen bond network. Since enantiomerization by directionality reversal is slowed down significantly at −30 °C, rotational exchange cross peaks (blue) are not observed, and only direct rOe cross peaks are observed (red).

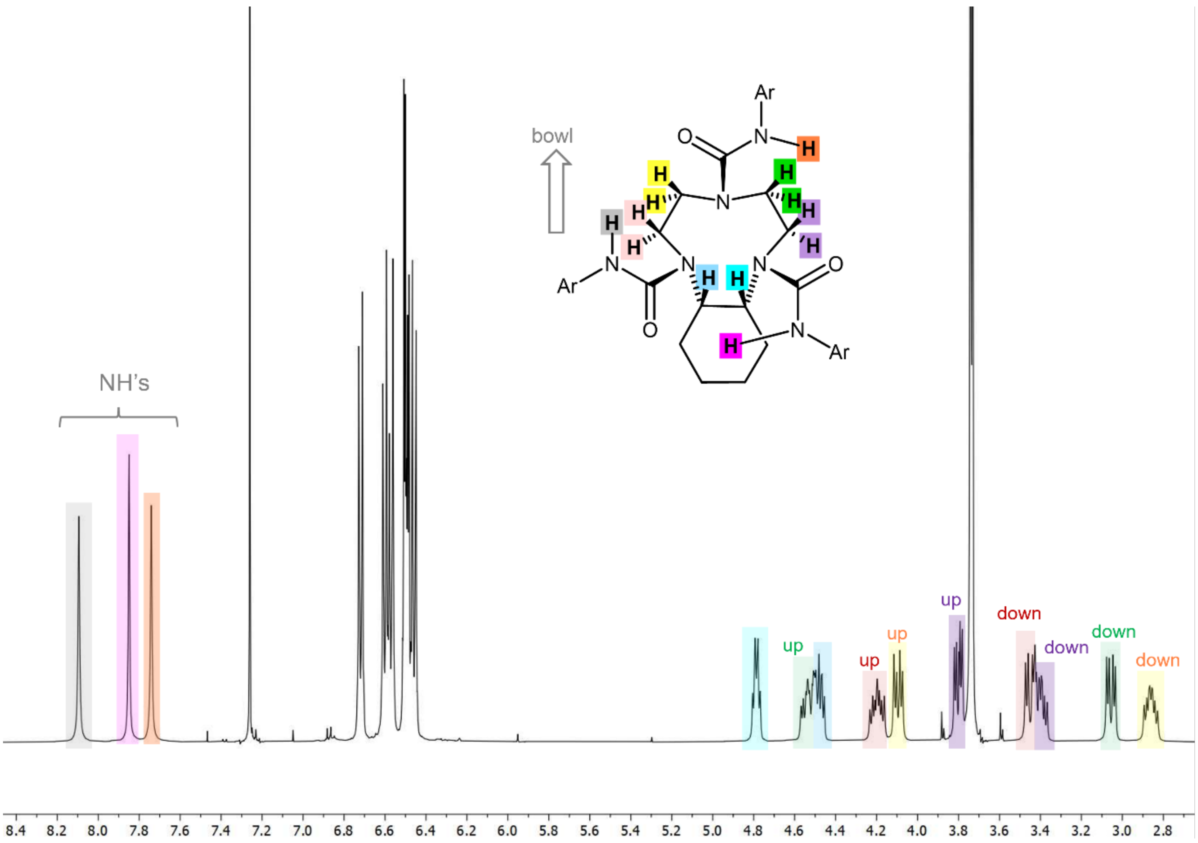

Figure S33 – A portion of the ^{1}H NMR spectrum of **3** (15 mM, 500 MHz, $CDCl_3$, 25 °C) showing assignments of the TACN ring protons and the NH protons by color (based on COSY, HSQC and ROESY data). Ethylene bridge protons labelled as 'up' are orientated syn to the hydrogen bond network. Ethylene bridge protons labelled as 'down' are orientated anti to the hydrogen bond network.

NMR Studies of **4**

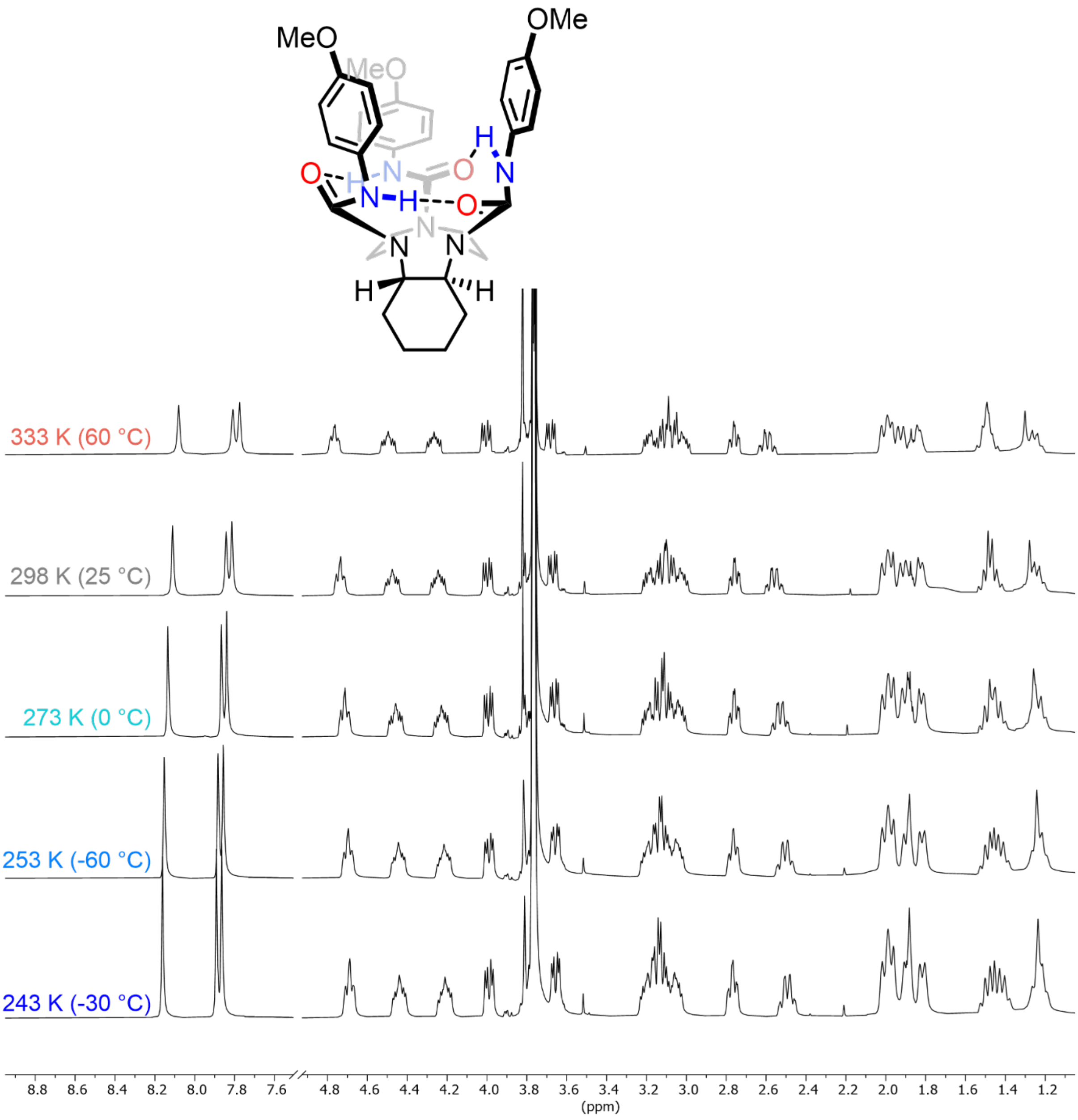

Figure S34 – VT-NMR stack plot for **4** (500 MHz, $CDCl_3$).

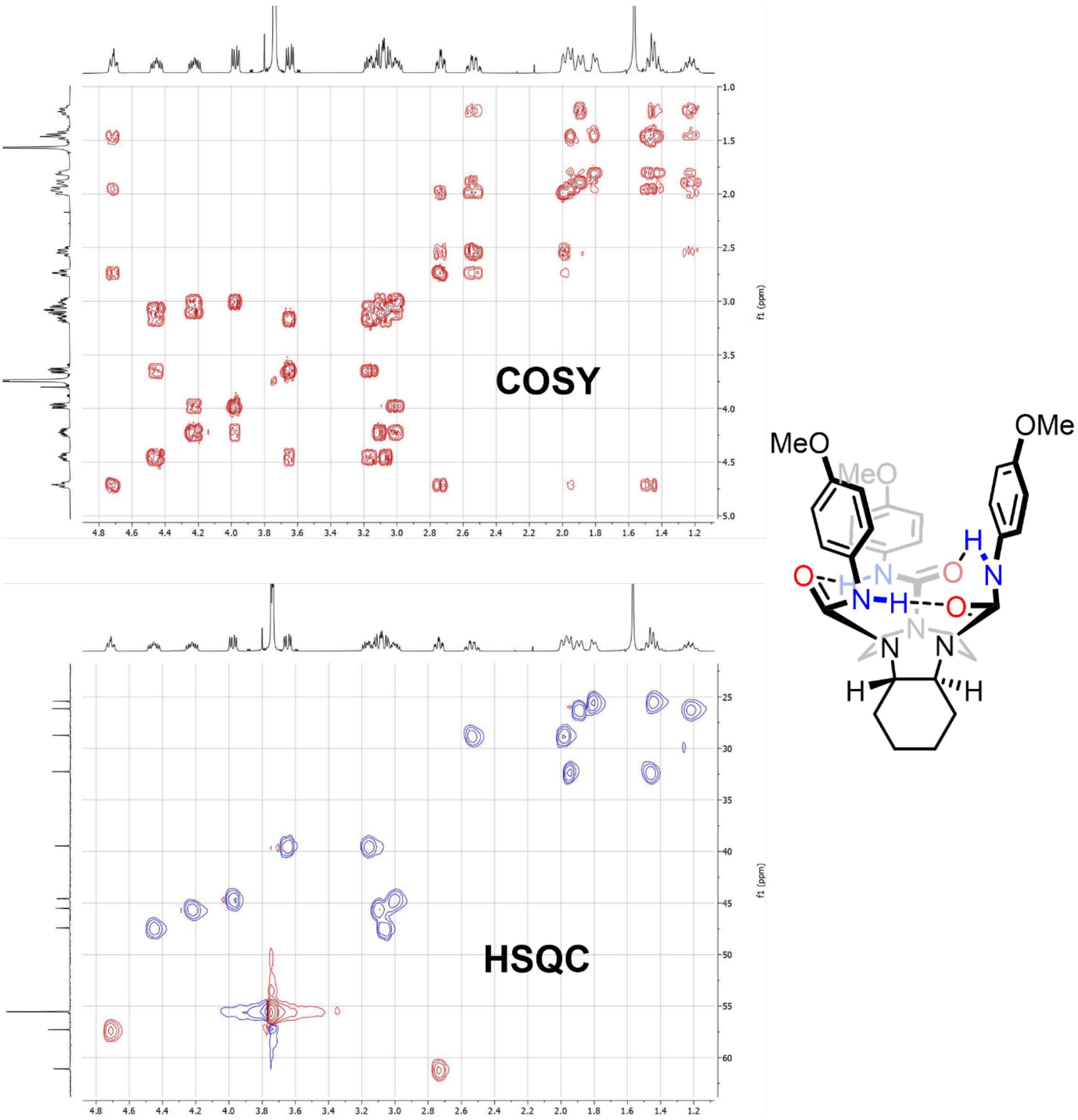

Figure S35 – Portions of the COSY and HSQC spectra of **4** (9 mM, 500 MHz, $CDCl_3$, 25 °C) showing the ethylene bridge and cyclohexyl proton regions.

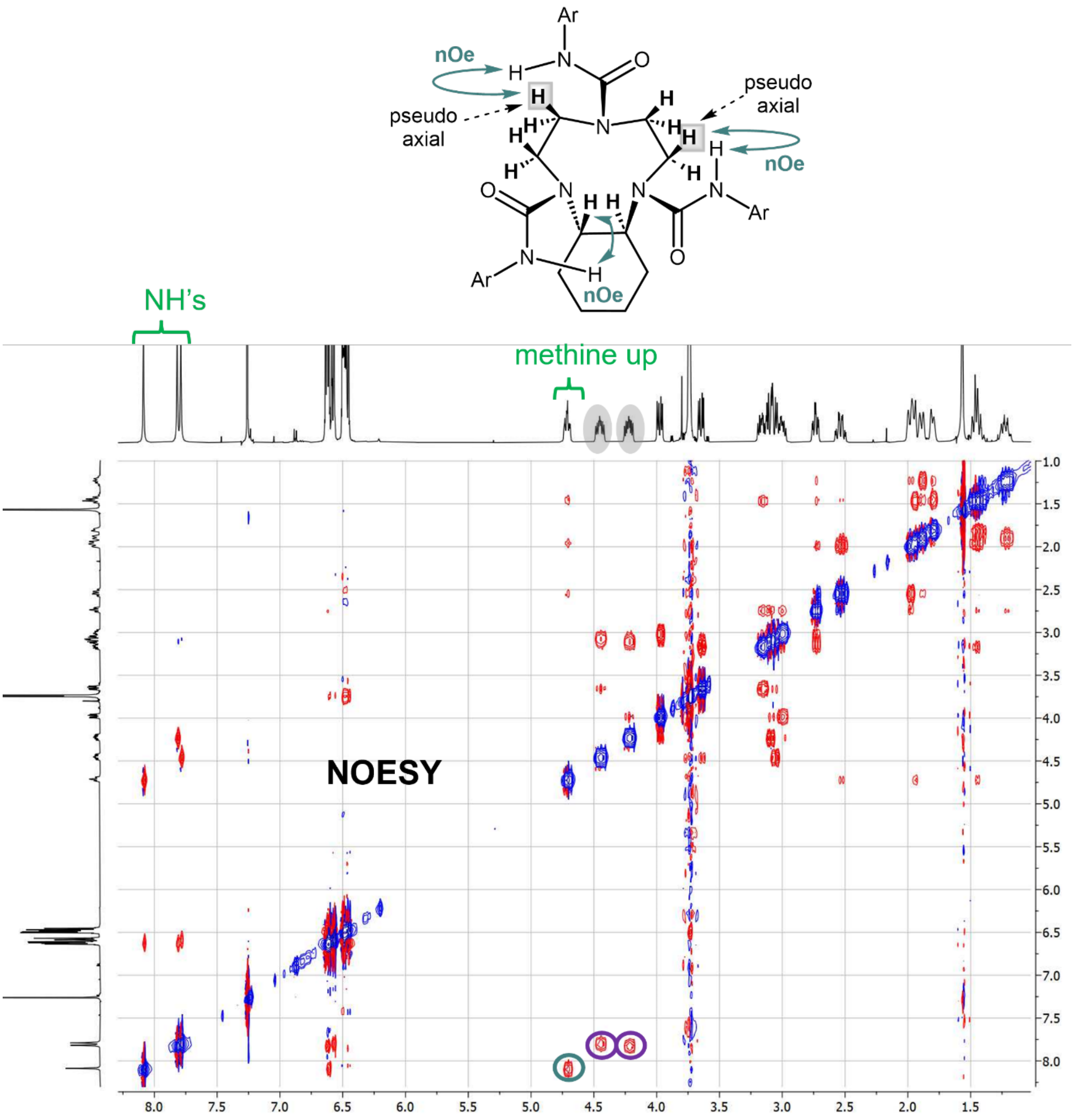

Figure S36 – NOESY spectrum of **4** (9 mM, 500 MHz, $CDCl_3$, 25 °C) showing nOe correlations (circled purple on the spectrum) between the pseudo axial protons on the TACN backbone (shaded grey) with their proximal NH's. A strong nOe between the methine proton labelled as 'up' and the NH proton proximal to the cyclohexyl group is also observed (circled in green), establishing that the relative stereochemical preference between the fixed stereocentres and the directionality of the hydrogen bond network (depicted as the *S*,*S*/clockwise enantiomer here) is the same as seen in the crystal structure.

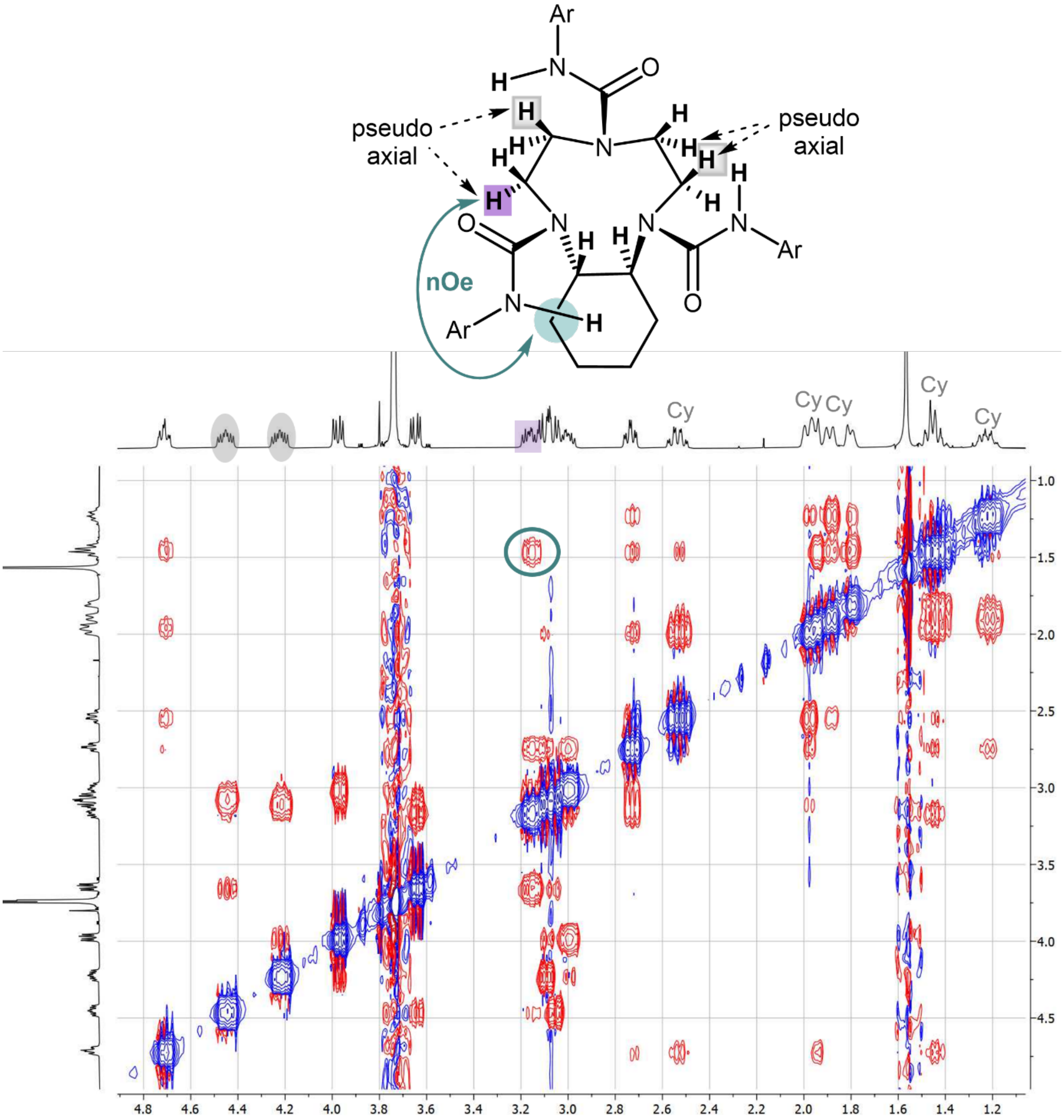

Figure S37 – A portion of the NOESY spectrum of **4** (9 mM, 500 MHz, $CDCl_3$, 25 °C) showing the ethylene bridge and cyclohexyl proton regions. An nOe correlation (circled green on the spectrum) is observed between the 'downwards' pseudo axial proton on the TACN backbone (shaded purple) and a methylene group of the cyclohexyl ring. The identification of this proton (shaded purple) allows precise assignment of the two pseudo axial protons proximal to the NH's (shaded grey) from COSY and HSQC data, and in turn, complete assignment of the NH and TACN protons (see below).

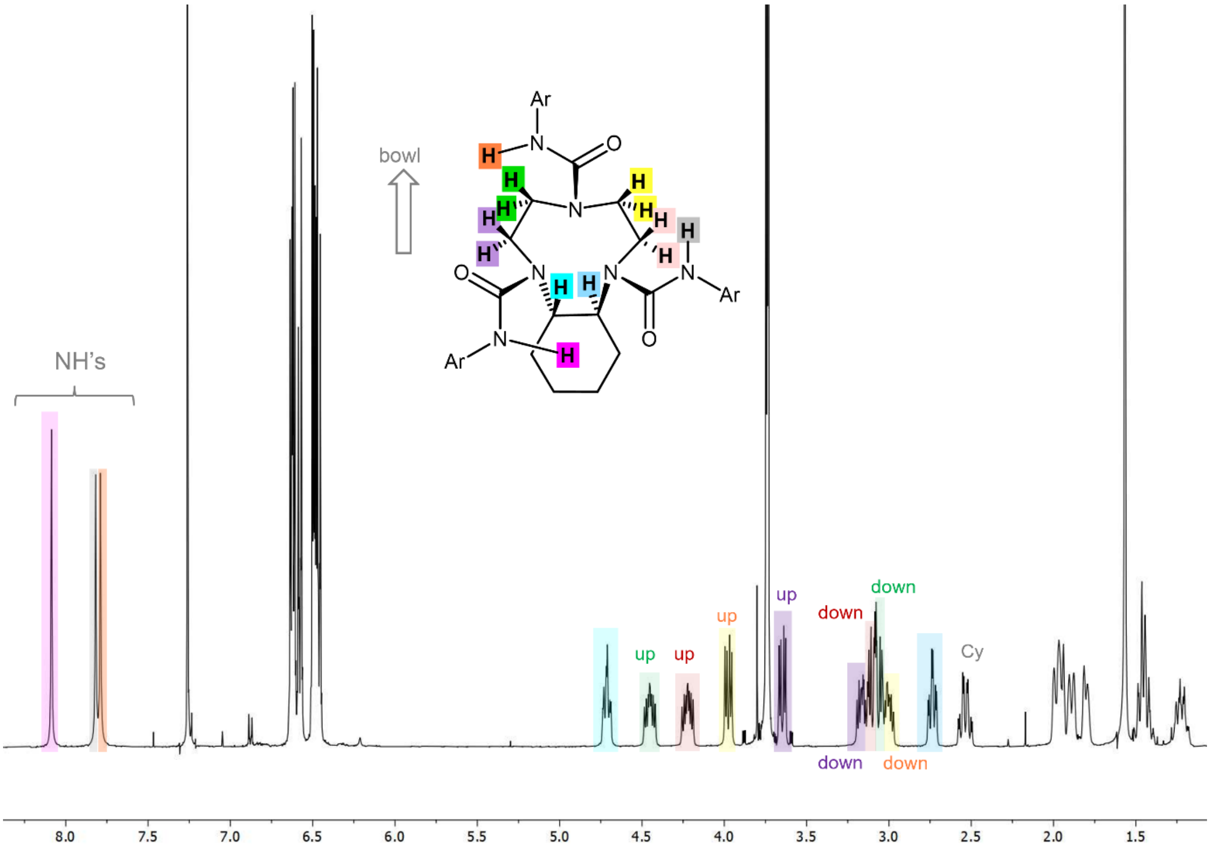

Figure S38 – ^{1}H NMR spectrum of **4** (9 mM, 500 MHz, $CDCl_3$, 25 °C) showing assignments of the TACN ring protons and the NH protons by color (based on COSY, HSQC and NOESY data). Ethylene bridge protons labelled as 'up' are orientated syn to the hydrogen bond network. Ethylene bridge protons labelled as 'down' are orientated anti to the hydrogen bond network.

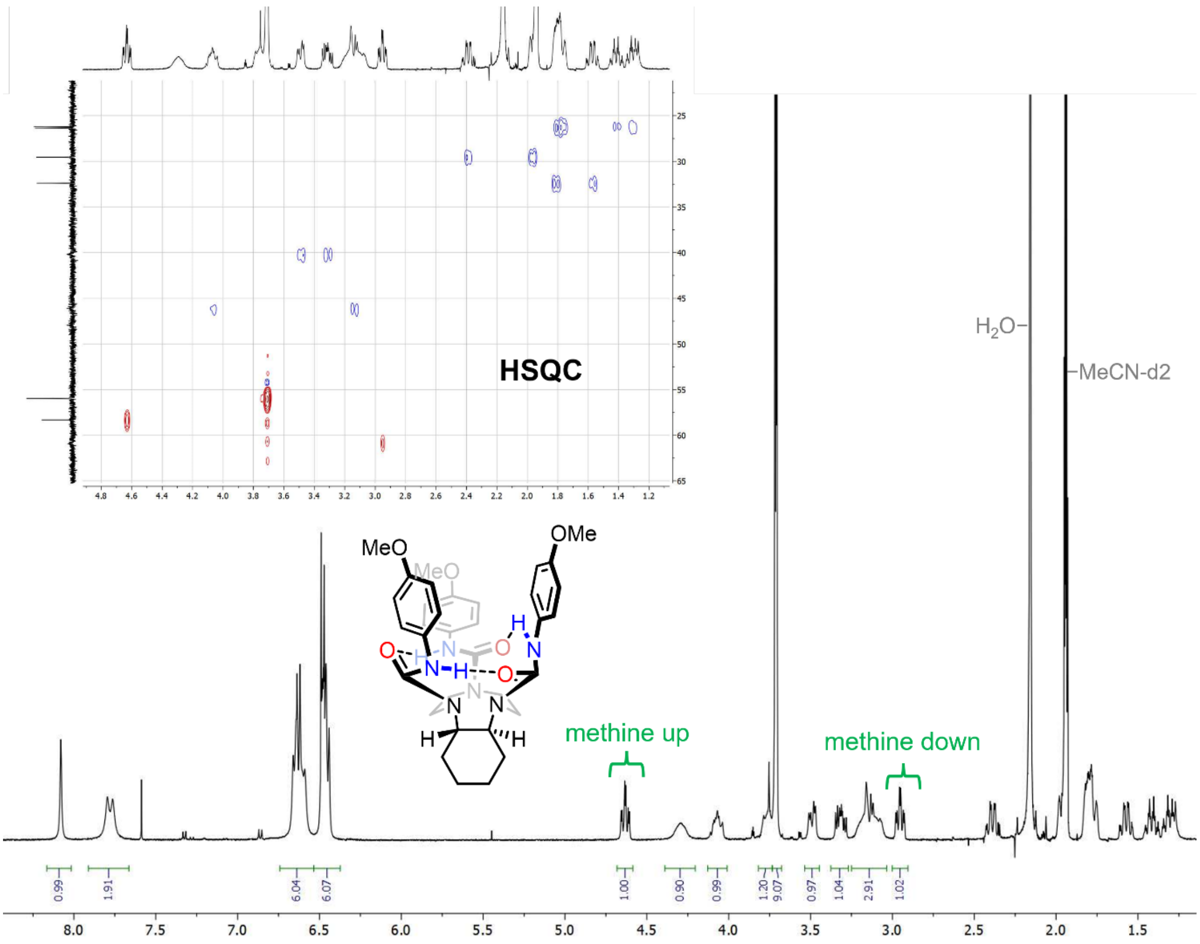

Figure S39 – ^{1}H NMR spectrum of **4** in CD_3CN (25 °C, 10 mM, 500 MHz) and a portion of the HSQC spectrum (in CD_3CN) showing carbon correlations of the ethylene bridge and cyclohexyl protons. The methine proton labelled as ‘up’ is orientated syn to the hydrogen bond network. The methine proton labelled as ‘down’ is orientated anti to the hydrogen bond network.

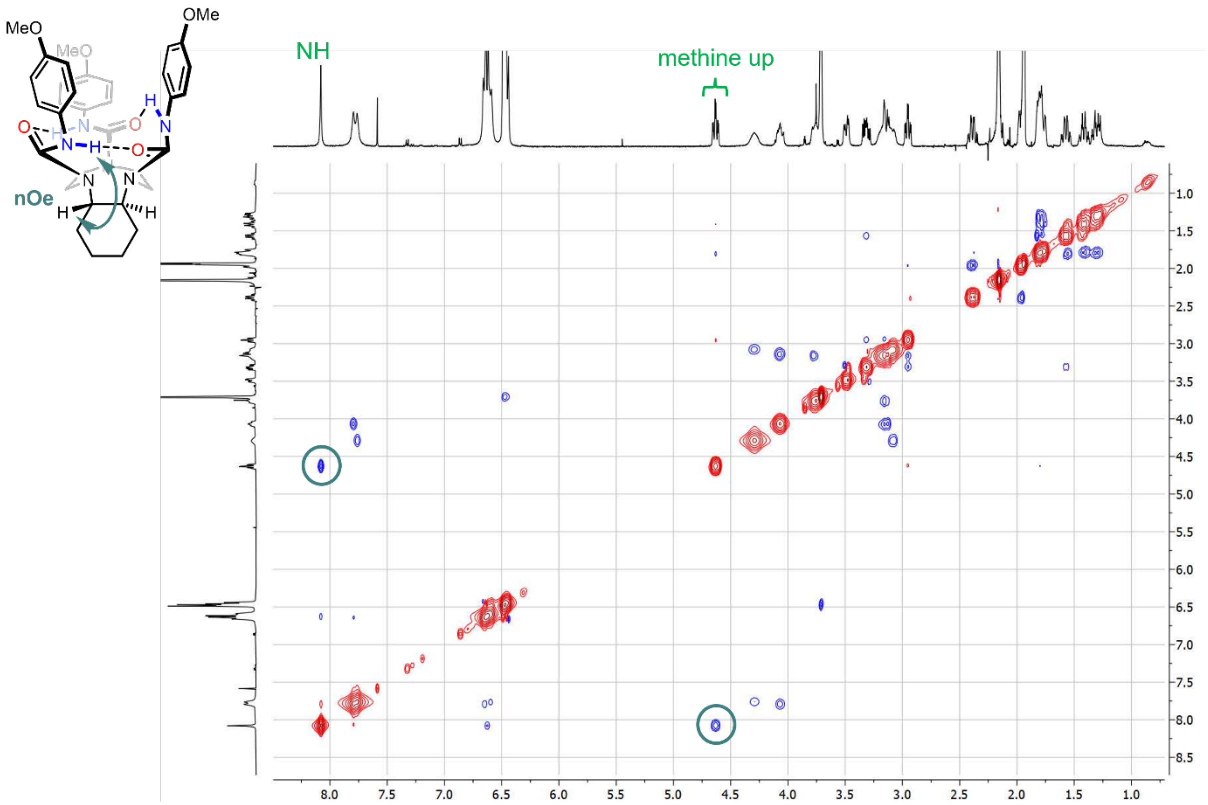

Figure S40 – NOESY spectrum of **4** in CD_3CN (25 °C, 10 mM, 500 MHz). A strong nOe between the methine proton labelled as 'up' and the NH proximal to the cyclohexyl group is observed (cross peaks circled in green), confirming that the relative stereochemical preference between the fixed stereocentres and the directionality of the hydrogen bond network is the same as in $CDCl_3$ (depicted as the *S*,*S*/clockwise enantiomer here).

NMR Studies of **8**

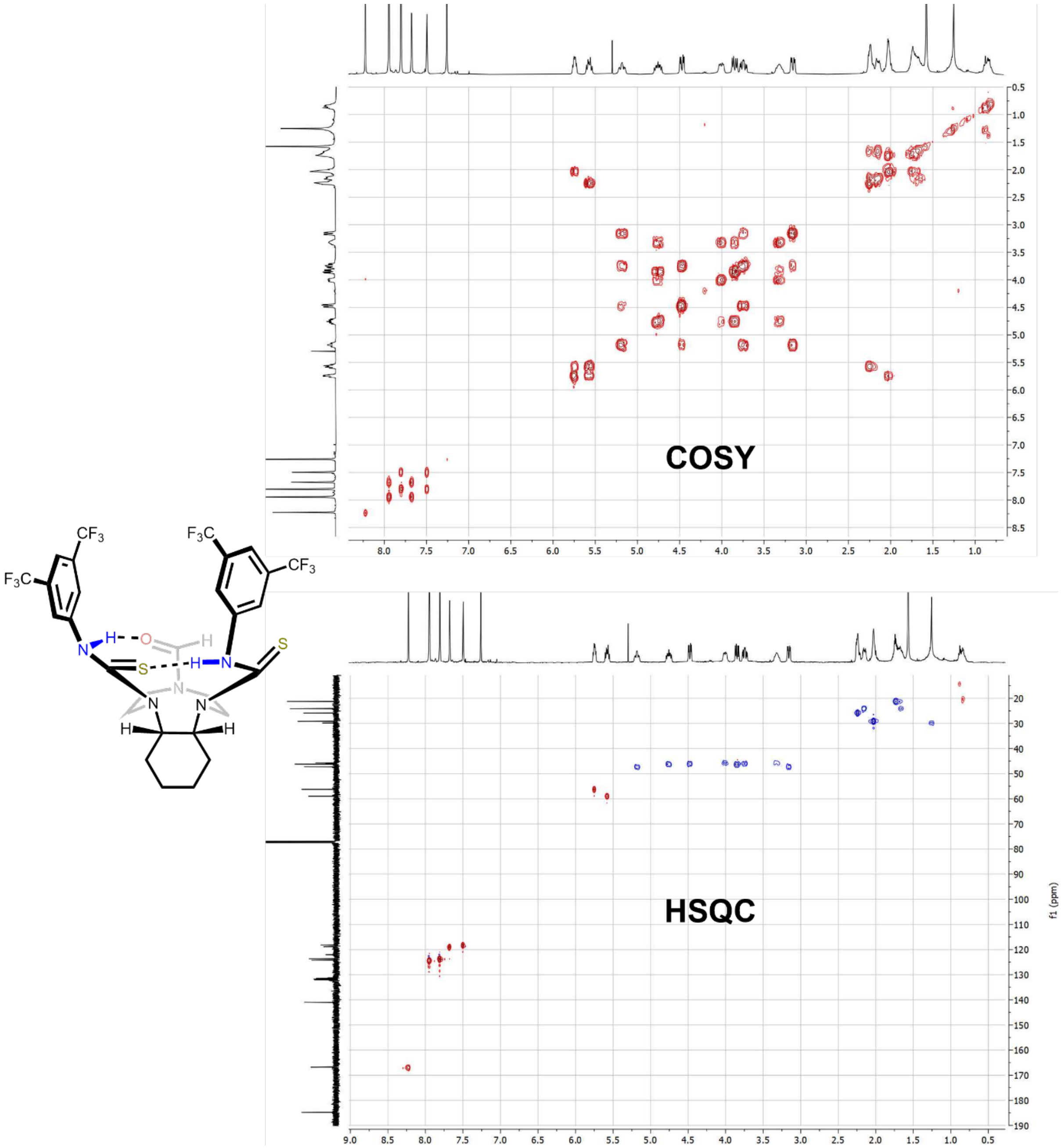

Figure S41 – COSY (400 MHz) and HSQC (500 MHz) spectra of **8** (both 25 mM, $CDCl_3$, 25 °C).

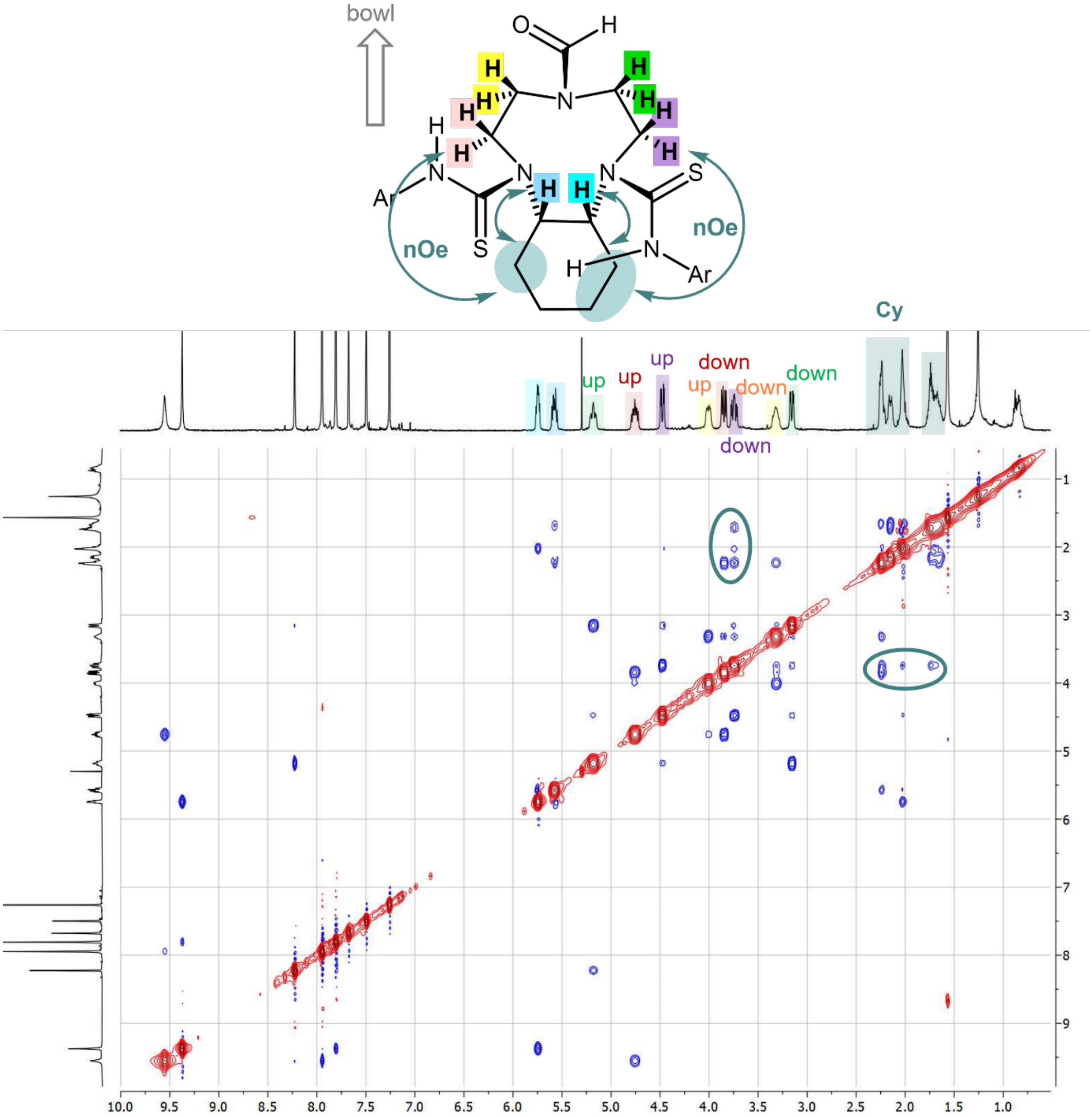

Figure S42 –NOESY spectrum of **8** (25 mM, 500 MHz, $CDCl_3$, 25 °C, 200 ms mixing time) showing assignments of the TACN ring protons by color (based on COSY, HSQC and NOESY data). Ethylene bridge protons labelled as 'up' are orientated syn to the hydrogen bond network. Ethylene bridge protons labelled as 'down' are orientated anti to the hydrogen bond network. Through space nOe cross peaks (circled in green on the spectrum) between the 'pink down' and 'purple down' TACN ring protons with the methylene protons of the cyclohexyl ring confirm that the cyclohexyl group is disposed anti to the hydrogen bond network.

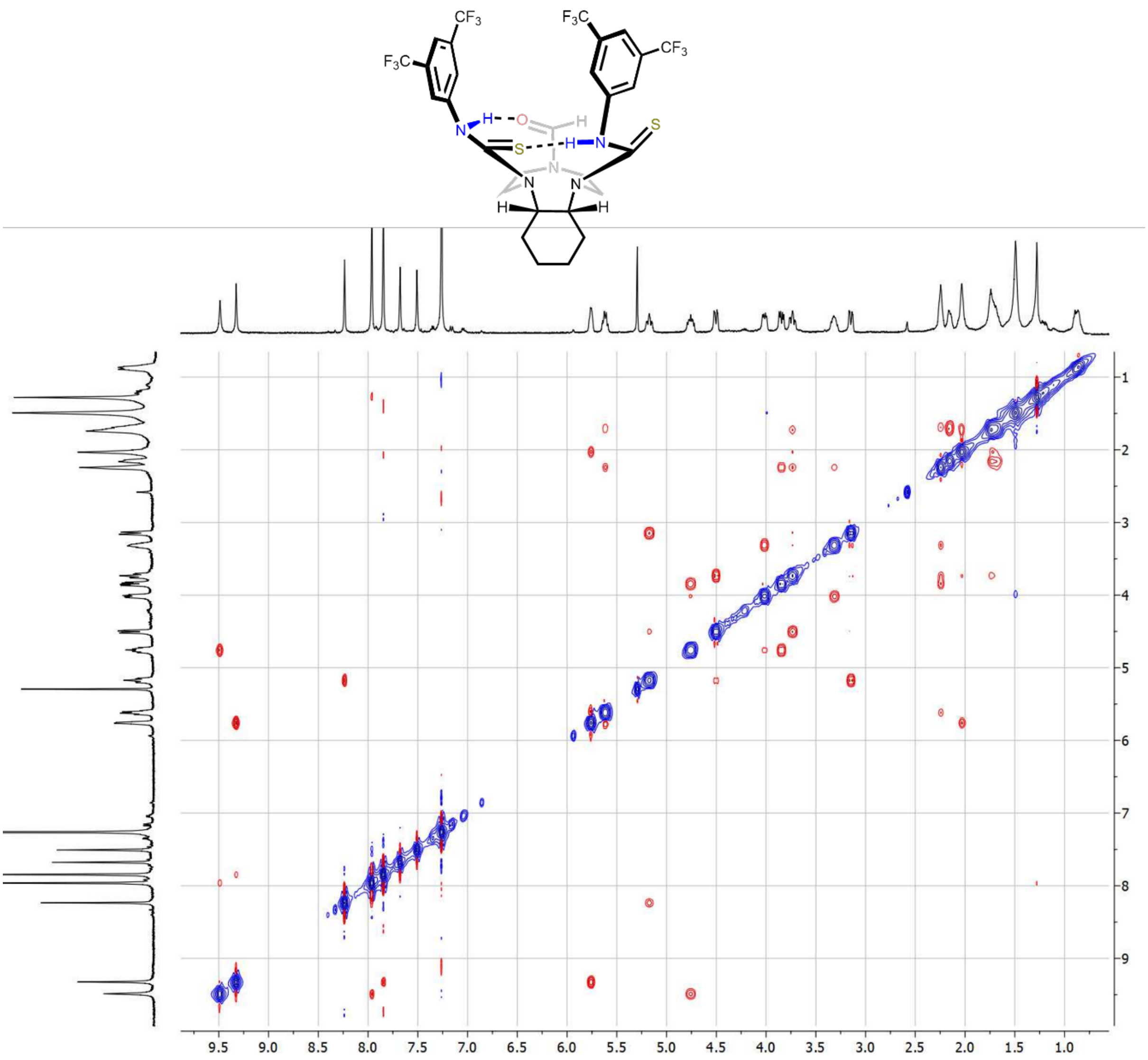

Figure S43 – NOESY spectrum of **8** at a higher temperature and with a longer mixing time (14 mM, 500 MHz, $CDCl_3$, 52 °C, 500 ms mixing time). These modified conditions did not result in the appearance of rotational exchange cross peaks, confirming a higher barrier to enantiomerization than previous analogues.

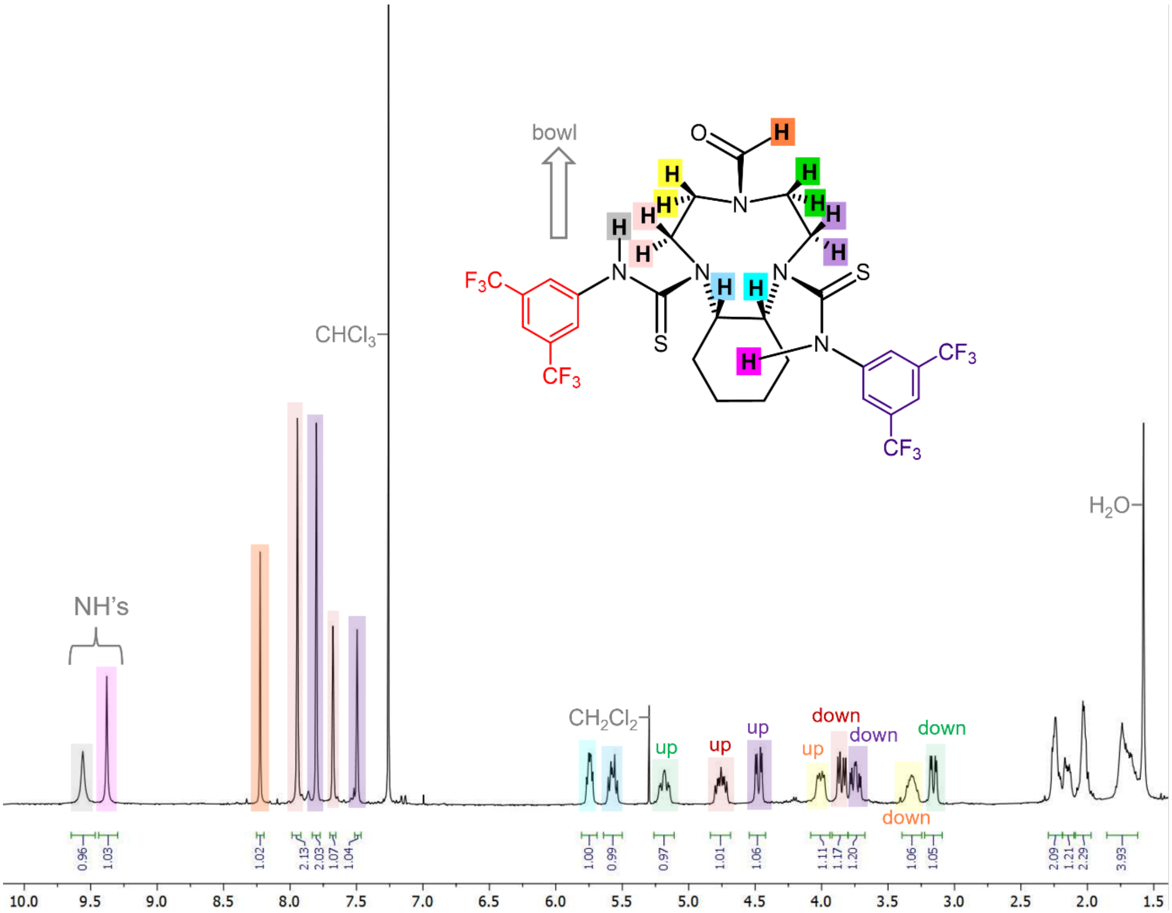

Figure S44 – ^{1}H NMR spectrum of **8** (25 mM, 400 MHz, $CDCl_3$, 25 °C) showing assignments of the TACN ring protons, the BTMP thiourea protons, the formamide proton and the NH protons by color (based on COSY, HSQC and NOESY data). Ethylene bridge protons labelled as 'up' are orientated syn to the hydrogen bond network. Ethylene bridge protons labelled as 'down' are orientated anti to the hydrogen bond network.

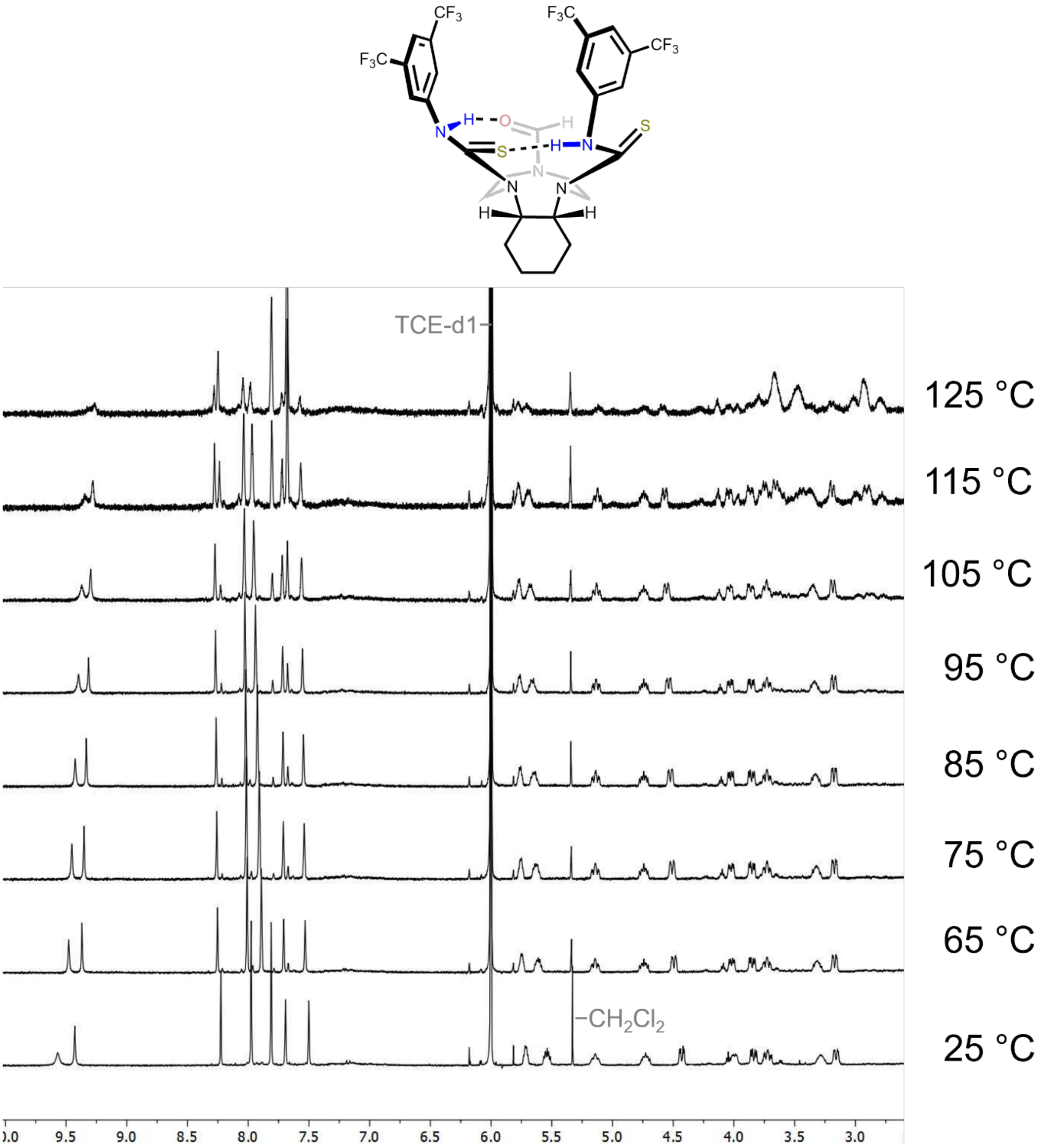

Figure S45 – VT-NMR stack plot for **8** (5 mM, TCE-d_2, 500 MHz). No coalescence of the BTMP urea or TACN proton signals was observed. The peak intensities decrease as the temperature increases due to partial decomposition of the sample; additional signals begin to appear in the spectra as a result.

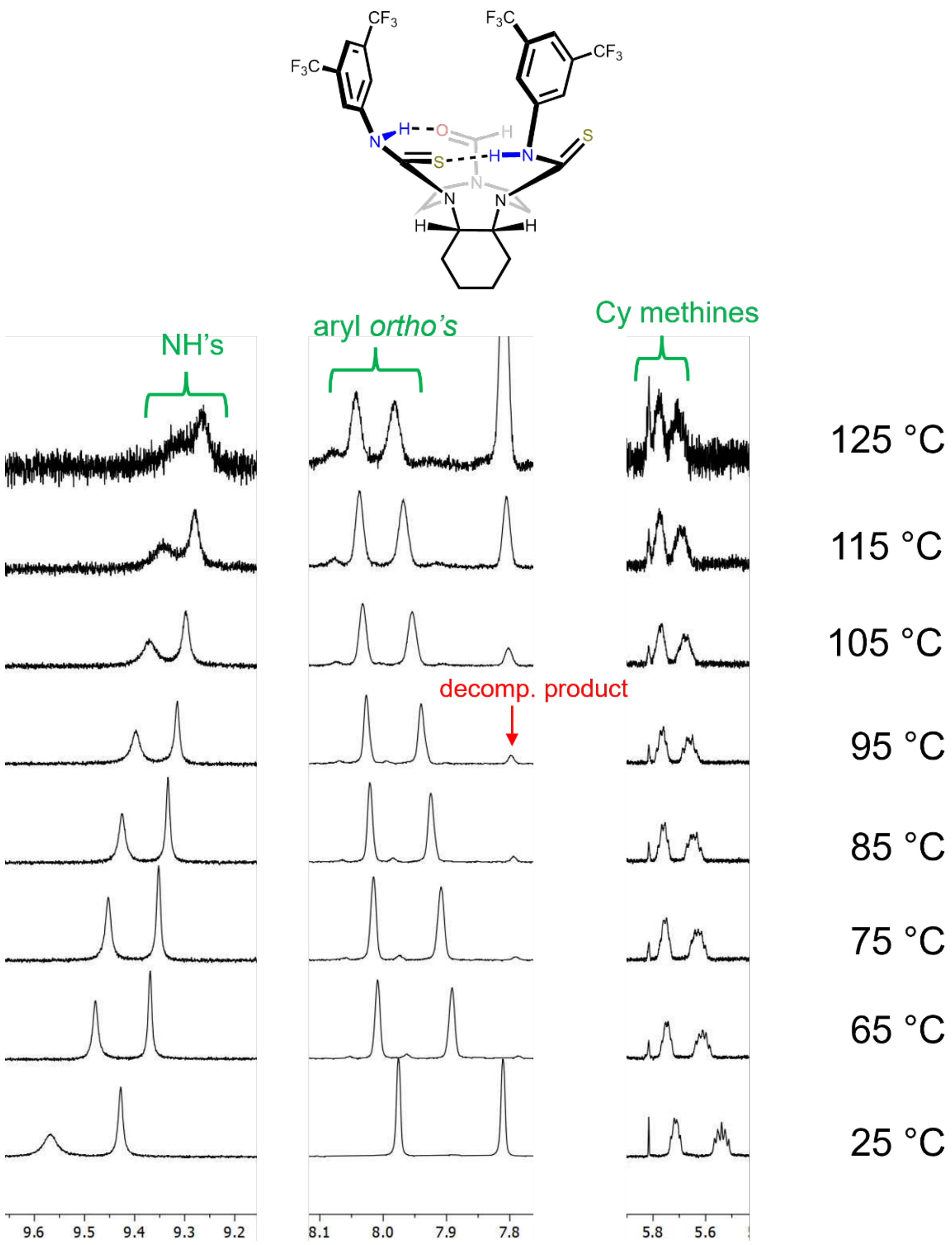

Figure S46 – Selected regions of the VT-NMR stack plot for **8** (5 mM, TCE-d_2, 500 MHz) showing signal broadening upon heating but a lack of coalescence in all cases. The peak intensities decrease as the temperature increases due to partial decomposition of the sample; additional signals begin to appear in the spectra as a result (e.g., a new singlet at ca 7.8 ppm emerges above 85 °C).

Stereochemical Stability of **8** using Circular Dichroism (CD) Spectroscopy

General Notes

All CD spectra of **8** were recorded using a cell with a 10 mm pathlength (as opposed to 1 mm), which was crucial to obtaining a good signal-to-noise ratio ([**8**] = approximately 30 μM). All CD spectra are corrected by subtracting the 'blank' spectrum of the solvent from the spectrum containing the analyte. Kinetic studies were performed by monitoring CD signal decay at the wavelength of maximum signal intensity (solvent dependent), as ascertained from the full CD spectra. The CD kinetic decay plots are corrected by subtracting the CD signal intensity of the solvent at the wavelength in question (obtained from the 'blank' spectrum) from the signal intensity of the analyte at every time point in the kinetic decay plots. Kinetic studies were performed with a CD signal recorded every 5 seconds (data pitch).

The enantiomers of **8** were separated by semi-preparative chiral HPLC immediately prior to every CD time course decay experiment. For each HPLC run, 50 μL of a 2.0 $mgmL^{-1}$ solution of (±)-**8** in 20% *i*-PrOH/hexane was injected, giving enantioenriched fractions of **8** and *ent*-**8** of approximately 30 μM (volume approximately 2−2.5 mL). CD spectra of **8** in 20% isopropanol/hexane (i.e., the eluent for the chiral HPLC) were recorded using enantioenriched fractions directly eluted from the column (Figure S47), with a small amount of additional 20% isopropanol/hexane typically added to the fraction of the first eluting enantiomer to reach the 2 mL required for the 10 mm CD cell. For CD measurements in other solvents, the 20% isopropanol/hexane eluent was immediately 'blown off' the fractions of interest using a stream of N_2 (which keeps the temperature cold during concentration), and the residue was taken up in 2 mL of chloroform or DMSO for immediate analysis by CD. All samples were subjected to CD analysis within 20 minutes of elution from the chiral HPLC column (including those requiring a solvent swap). Small differences in the initial intensities of the CD signals (in the same solvent) are due to slight changes in concentration between samples (which are all approximately 30 μM) and slight variations in the time between elution from the chiral HPLC column and commencing the CD decay experiments (in some cases a full CD spectrum was recorded initially to determine the wavelength of maximum CD signal intensity to be used in the subsequent kinetic experiment).

Rate constants for racemization (k_{rac}) were determined using a first order decay model applied to the CD kinetic data. Enantiomerization barriers ($\Delta G^{\ddagger}_{25\ °C}$) were calculated by plugging the enantiomerization rate constant ($k_{ent} = 0.5k_{rac}$) into the rearranged Eyring equation ($\Delta G^{\ddagger}_{T} = RT\ln(k_{B}T/hk_{ent})$). Values of k_{rac} (and hence $k_{ent} = 0.5k_{rac}$) in 20% isopropanol/hexane and chloroform were determined at three different temperatures, allowing the entropy and enthalpy of activation to be determined from the corresponding Eyring plots. Plugging these entropy and enthalpy of activation constants into the Gibbs free energy of activation equation ($\Delta G^{\ddagger}_{T} = \Delta H^{\ddagger}_{T} - T\Delta S^{\ddagger}_{T}$) gave activation energy values ($\Delta G^{\ddagger}_{25\ °C}$) within 0.1 kJ mol^{-1} of the values determined by direct use of k_{ent} in the rearranged Eyring equation. The values of $\Delta G^{\ddagger}_{25\ °C}$ quoted in the manuscript are those determined by direct use of k_{ent} in the rearranged Eyring equation.

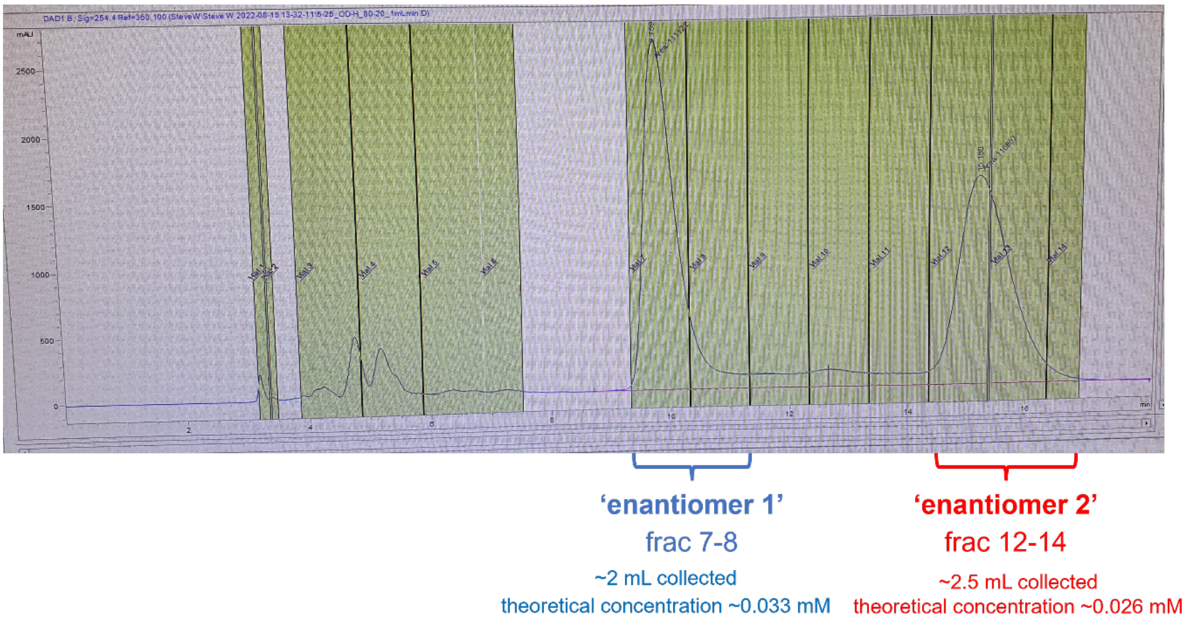

Figure S47 – Representative HPLC chromatogram of (±)-**8** at a wavelength of 254 nm (OD-H chiral stationary phase, 20% *i*-PrOH/hexane, 1 mL min^{-1}) showing the fraction collector trace. The approximate theoretical concentrations listed for each enantiomer are based on the amount of the racemate injected: 50 μL of a 2.0 mgmL^{-1} solution in 20% *i*-PrOH/hexane.

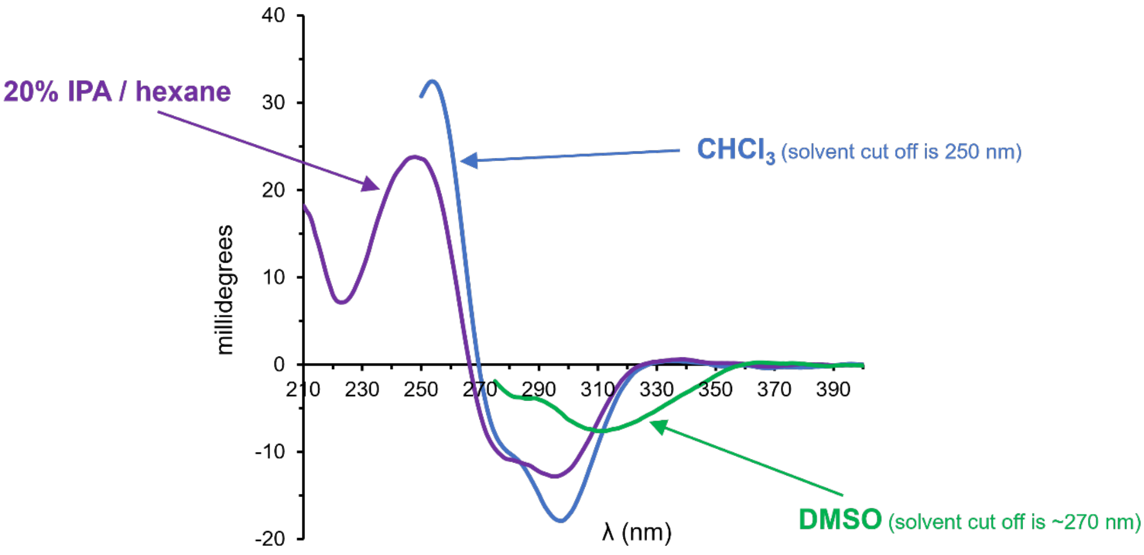

Figure S48 – CD spectra of enantioenriched **8** (first eluting enantiomer only) in 20% *i*-PrOH/hexane, chloroform and DMSO (all: ~33 μM, 25 °C, *l* = 10 mm). The solvent cut offs for chloroform and DMSO are listed on the spectra.

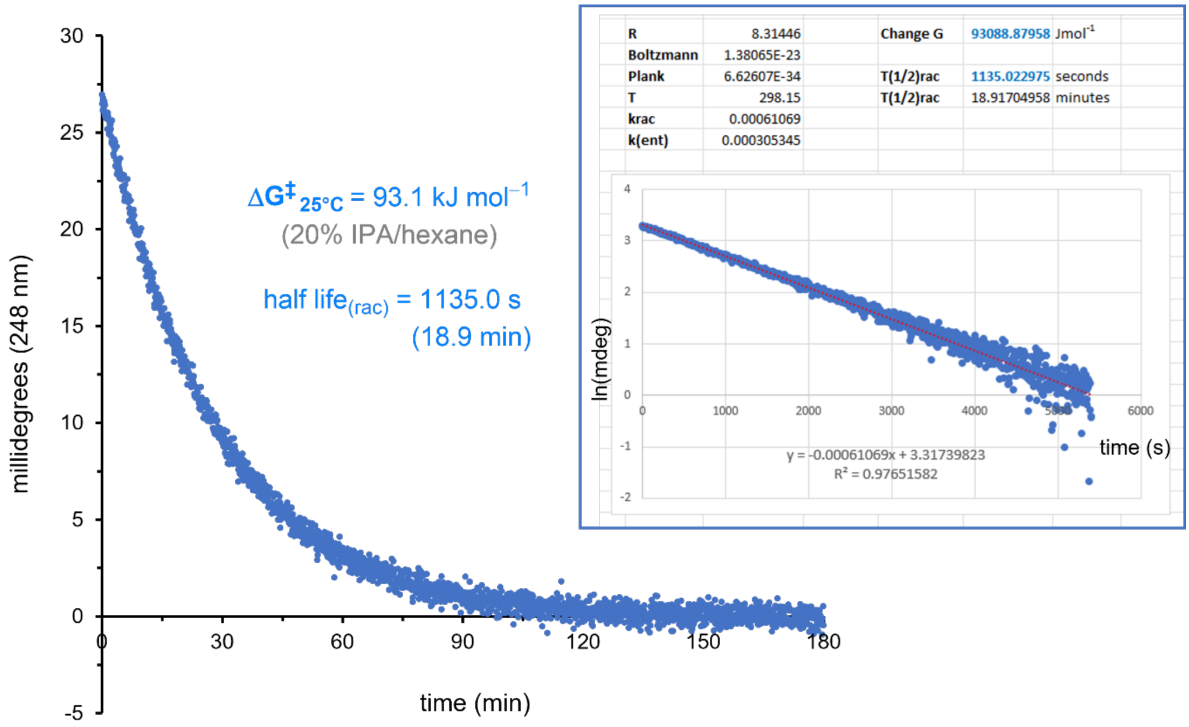

Figure S49 – Time course decay of the CD signal of an enantioenriched sample of **8** at a wavelength of 248 nm in 20% *i*-PrOH/hexane (~33 μM, 25 °C, *l* = 10 mm). The sample is enriched initially in the enantiomer with the shorter HPLC retention time. The enantiomerization barrier and racemization half-life are shown on the graph, as determined from the first order decay rate constant (blue box).

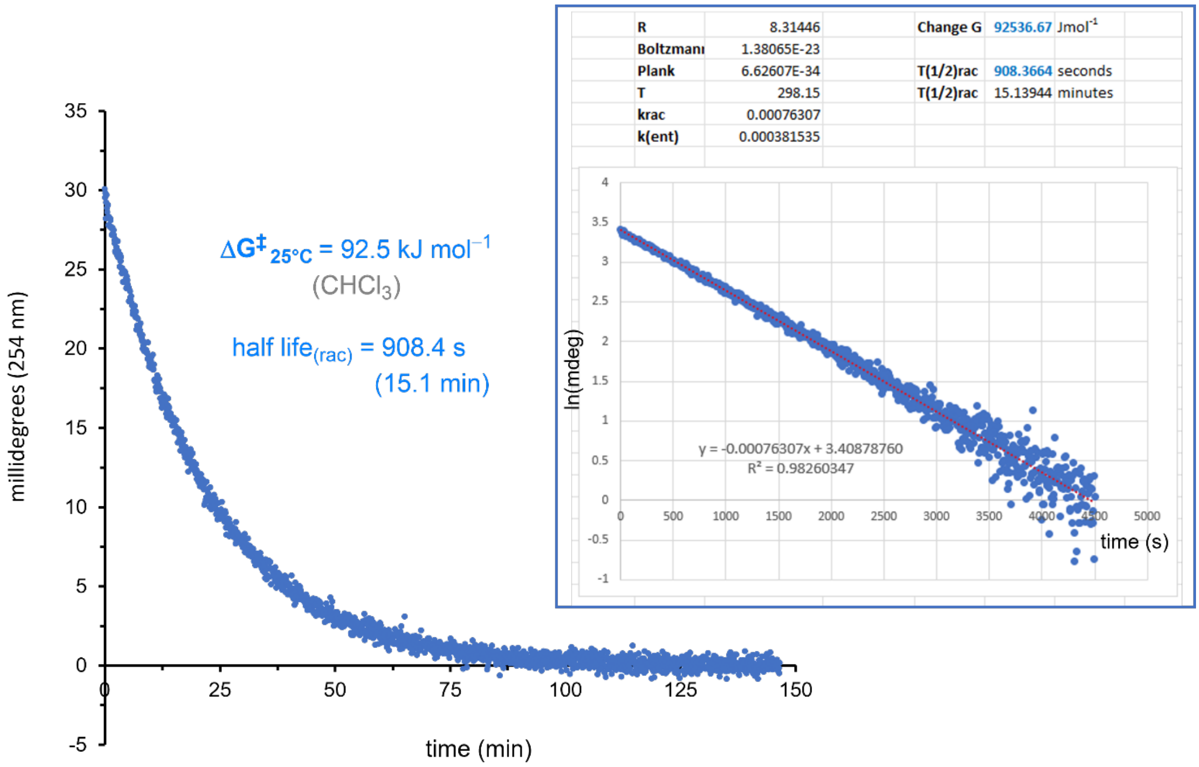

Figure S50 – Time course decay of the CD signal of an enantioenriched sample of **8** at a wavelength of 254 nm in chloroform (~33 µM, 25 °C, *l* = 10 mm). The sample is enriched initially in the enantiomer with the shorter HPLC retention time. The enantiomerization barrier and racemization half-life are shown on the graph, as determined from the first order decay rate constant (blue box).

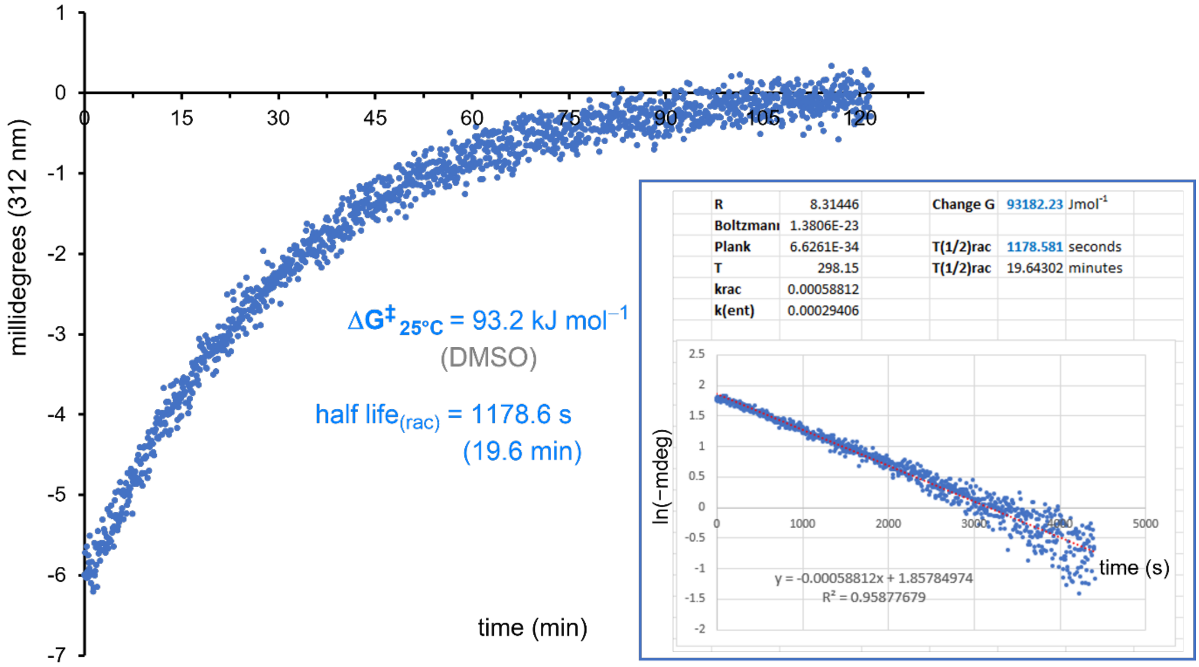

Figure S51 – Time course decay of the CD signal of an enantioenriched sample of **8** at a wavelength of 312 nm in DMSO (~33 µM, 25 °C, *l* = 10 mm). The sample is enriched initially in the enantiomer with the shorter HPLC retention time. The enantiomerization barrier and racemization half-life are shown on the graph, as determined from the first order decay rate constant (blue box).

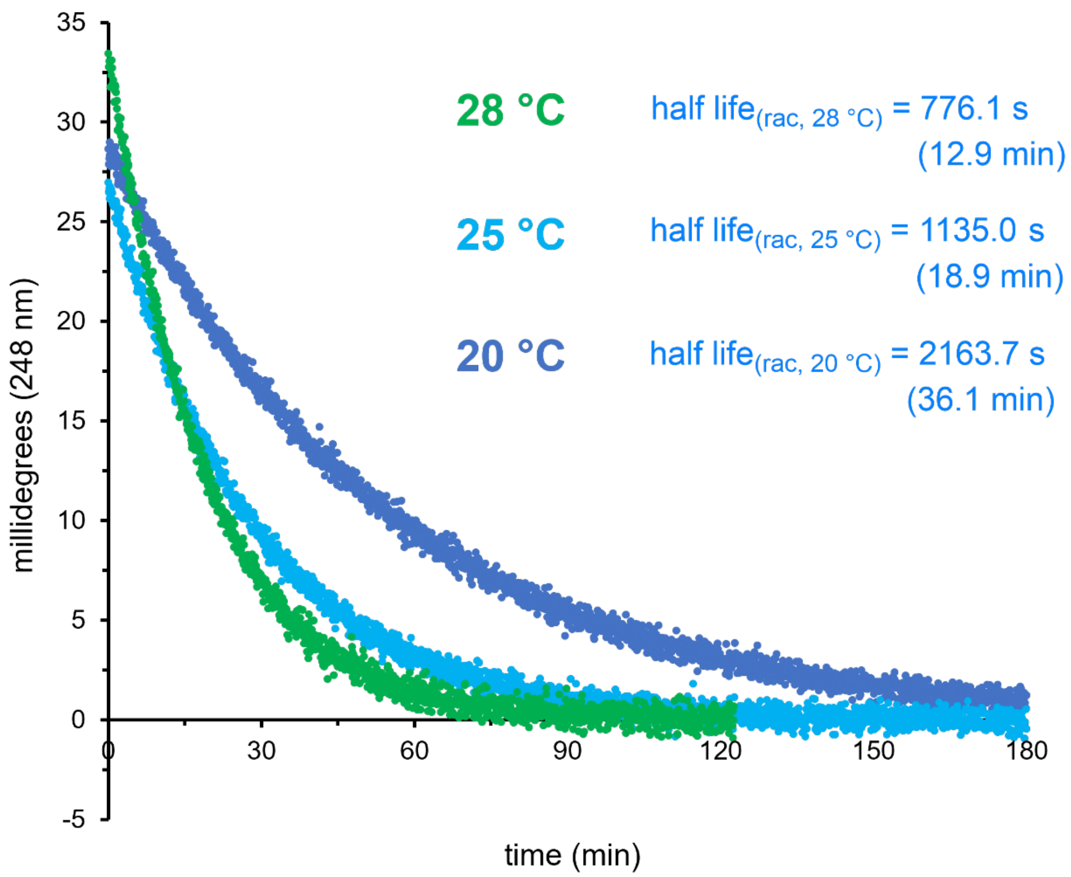

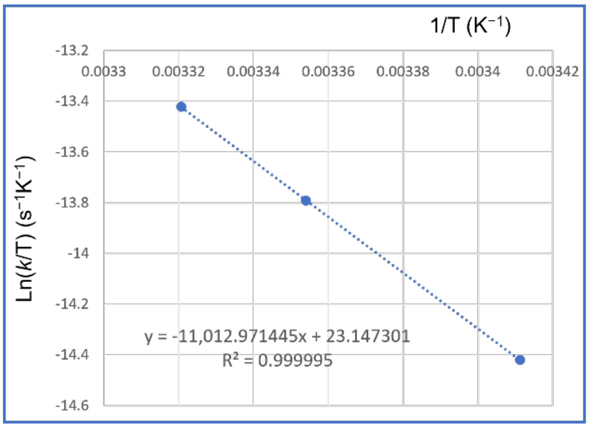

R	8.31446	Y intercept	23.1473	
Boltzmann	1.3806E-23	gradient	-11012.97	
Plank	6.6261E-34			
		enthalpy	91566.91	$Jmol^{-1}$
		entropy	-5.094085	$Jmol^{-1}K^{-1}$

Temperature	k_{ent} (per sec)	k_{rac} (per sec)	1/T	ln(k/T)	ΔH	TΔS	ΔG	
293.15	0.000160175	0.00032035	0.003411223	-14.41992801	91566.91019	-1493.331061	93.06024125	$kJmol^{-1}$
298.15	0.000305345	0.00061069	0.003354016	-13.79166499	91566.91019	-1518.801487	93.08571168	$kJmol^{-1}$
301.15	0.00044653	0.00089306	0.003320604	-13.42161245	91566.91019	-1534.083742	93.10099393	$kJmol^{-1}$

Figure S52 – (Top) Time course decay of the CD signal of an enantioenriched sample of **8** at a wavelength of 248 nm in 20% *i*-PrOH/hexane (~33 µM, l = 10 mm) at 28 °C, 25 °C and 20 °C. Note that the 25 °C kinetic trace is the same as given previously but is repeated here for comparison. Each sample is enriched initially in the enantiomer with the shorter HPLC retention time. (Bottom) Eyring plot using the temperature dependent rate constants to determine the contributions of enthalpy and entropy to the enantiomerization barrier (selected results are also tabulated below).

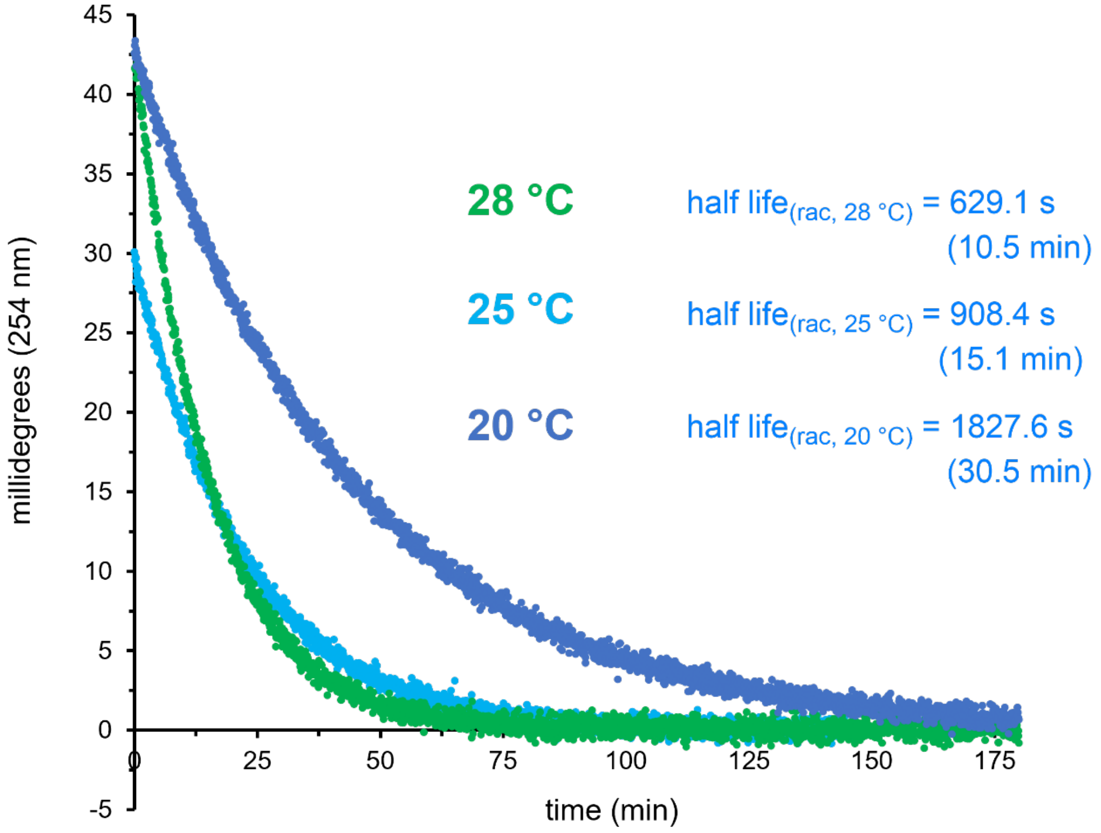

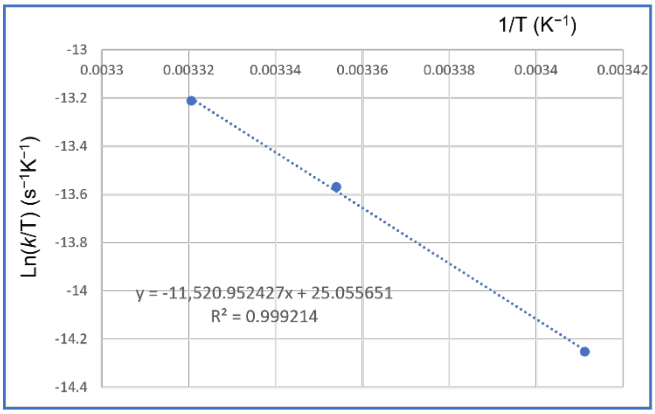

**R**	8.31446		**Y intercept**	25.055651	
**Boltzmann**	1.38E-23		**gradient**	-11520.952	
**Plank**	6.63E-34				
			**enthalpy**	95790.4981	$Jmol^{-1}$
			**entropy**	10.7728229	$Jmol^{-1}K^{-1}$

**Temperature**	**k_{ent} (per sec)**	**1/T**	**ln(k/T)**	**ΔH**	**TΔS**	**ΔG**	
293.15	0.00018963	0.003411223	-14.25112018	95790.49812	3158.053036	92.6324451	$kJmol^{-1}$
298.15	0.000381535	0.003354016	-13.56890468	95790.49812	3211.917151	92.578581	$kJmol^{-1}$
301.15	0.00055091	0.003320604	-13.21154758	95790.49812	3244.235619	92.5462625	$kJmol^{-1}$

Figure S53 – (Top) Time course decay of the CD signal of an enantioenriched sample of **8** at a wavelength of 254 nm in chloroform (~33 µM, *l* = 10 mm) at 28 °C, 25 °C and 20 °C. Note that the 25 °C kinetic trace is the same as given previously but is repeated here for comparison. Each sample is enriched initially in the enantiomer with the shorter HPLC retention time. (Bottom) Eyring plot using the temperature dependent rate constants to determine the contributions of enthalpy and entropy to the enantiomerization barrier (selected results are also tabulated below).

Table S25 – Summary of enantiomerization barriers and their enthalpic and entropic contributions for compound **8**, as determined by Eyring analysis.

**Solvent**	**$\Delta G^{\ddagger}_{298\ K}$** (kJ mol^{-1})	**$\Delta H^{\ddagger}$** (kJ mol^{-1})	**$\Delta S^{\ddagger}$** (J mol^{-1} K^{-1})
20% *i*-PrOH/hexane	93.09	91.57	−5.09
$CHCl_3$	92.58	95.79	10.77

Contrasting Conformations of **1a** and **9**

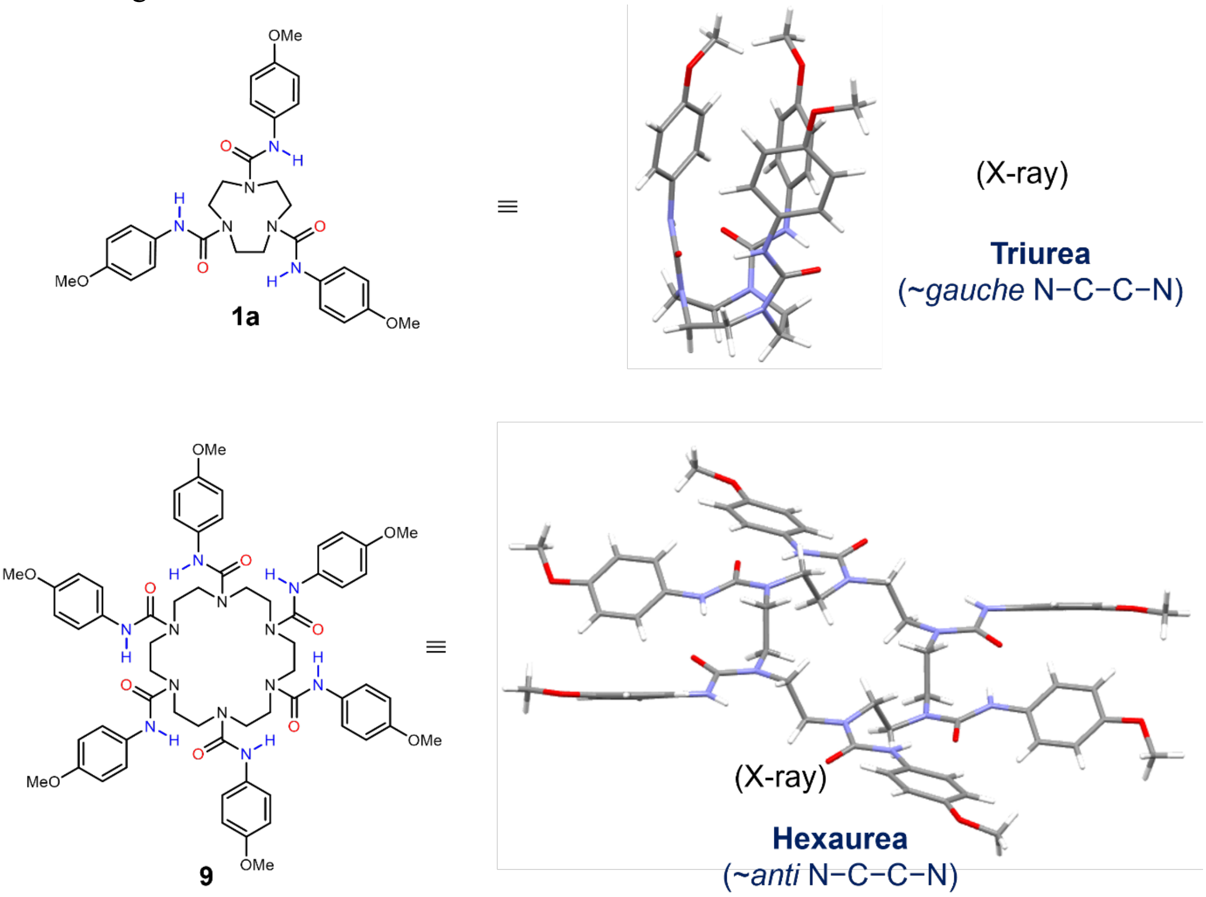

Figure S54 – The gauche conformations of the ethylene bridges (N−C−C−N linkages) of **1a** enforce a folded, bowl-like conformation (top) while the anti conformations of the ethylene bridges in the larger homologue **9** enforce an unfolded conformation (bottom).

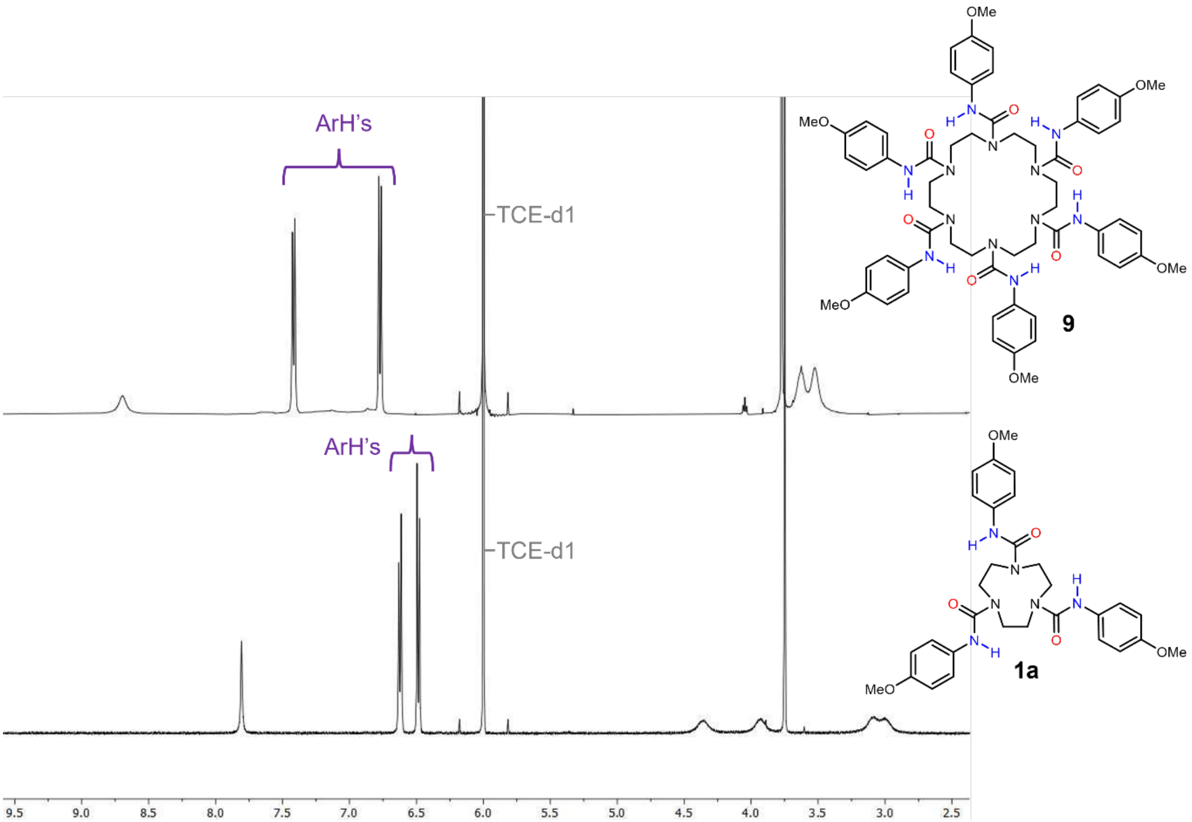

Figure S55 – ^{1}H NMR spectra of **9** (top) and **1a** (bottom) (both: 2 mM, 500 MHz, TCE-d_2, 25 °C). The conformational divergence from folded to extended with increasing macrocycle size has a notable influence on the chemical shifts of the urea aromatic protons which are more shielded in the folded conformation; for example in **1a** (folded; δ_H = 6.49 and 6.62) relative to **9** (unfolded; δ_H = 6.77 and 7.42).

Computational Details

Geometry Optimization

Density functional theory (DFT) calculations were performed with Gaussian 16.[S11] The geometries were optimized using the hybrid B3LYP functional[S12,S13] with D3(BJ) dispersion correction[S14] and the split-valence plus polarization def2-SVP basis set.[S15] All DFT calculations were conducted with the *ultrafine* integration grid. Frequency calculations were performed at the same level of theory as for geometry optimizations to verify the stationary points as minima (no imaginary frequencies) as well as to obtain thermal Gibbs free energy corrections at 298 K. Thermochemical free energy corrections were then recomputed at 298 K and concentration 1.0 $mol.L^{-1}$ using the GoodVibes software package with Grimme's entropy corrections using quasi-rigid rotor harmonic approximation (qRRHO)[S16] applied to all frequencies below 100 cm^{-1}.[S17]

The conformers were generated using GFN2-xTB metadynamics simulation.[S18] Generally, 300 confomers were generated; and 4 lowest energy structures were optimized by DFT.

Single-Point Calculations

To refine the computed energy, single point calculations were performed in Gaussian 16 at B3LYP-D3(BJ)/def2-TZVPP/SMD(MeCN) level of theory. SMD implicit solvation model for dichloromethane was used.[S19]

CD and absorption spectra were calculated with PBE0 hybrid[S20] and CAM-B3LYP range-separated hybrid[S21] DFT functionals with def2-TZVPP basis set, Rijcosx approximation and CPCM solvation model in ORCA 5.0.3 computational software.[S22] VeryTightSCF convergence criteria and Tamm-Dancoff approximation were used. The spectra were plotted in Spectragryph (F. Menges "*Spectragryph - optical spectroscopy software*", Version 1.2.16.1, 2022, http://www.effemm2.de/spectragryph/).

Gibbs Free Energies

The ΔG value is obtained by adding the corresponding free energy corrections at 298 K calculated at the B3LYP-D3(BJ)/def2-SVP level, to ΔE, calculated at the single-point calculation at B3LYP-D3(BJ)/def2-TZVPP/SMD(MeCN) level with SMD solvation correction.

Computed Energies of **3** / **3'**

Table S26 – Computed Gibbs free energies (MeCN, 298 K, 1 M) of **3** (*d1*) and its diastereomer **3'** (*d2*) based on B3LYP-D3(BJ)/def2-TZVPP/SMD single point calculations.

Compound	B3LYP/SVP	G_{corr}	B3LYP/TZVPP/SMD	ΔG_{corr}	ΔGsolv [$kJ\cdot mol^{-1}$]
3-d1	-2099.535257	0.682574	-2101.885148	-2101.202574	0
3'-d2	-2099.532907	0.682425	-2101.881062	-2101.198637	10.3

Other generated conformers of both *cis*-diastereomers were optimized but converged to the structures with rotated MeO groups having identical energy due to symmetrical structure of the molecule. The backbone structure was almost identical in all cases.

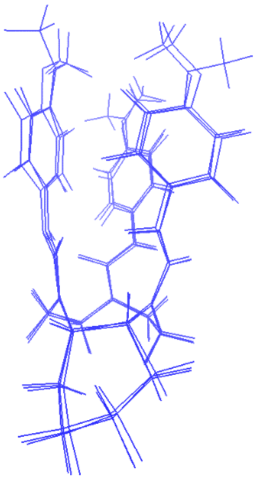

Figure S56 – Overlaid structures of optimized conformers of **3** showing MeO groups rotations but conserved backbone structure.

Computed Geometric Properties of the H-Bonds in **4** / **4'**

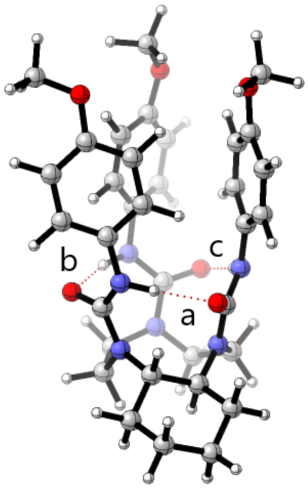

Table S27 – Selected geometrical features of (*S*,*S*)-**4** (*diastereomer 1*).

	a	*b*	*c*
NH···O bond length [Å]	1.89	1.93	1.88
NH···O angle [°]	161.2	160.4	159.6
C=O···H angle [°]	105.8	104.8	104.3

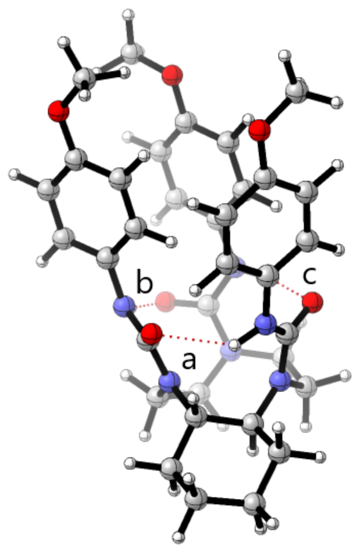

Table S28 – Selected geometrical features of (*S,S*)-**4' (***diastereomer 2*, conf6**)**. The NH···O bond length of the unit *a* is longer than in (*S,S*)-**4**, and the NH···O and C=O···H angles deviate from ideal values (180° and 120°) more than in (*S,S*)-**4**.

	a	*b*	*c*
NH···O bond length [Å]	2.21	1.80	1.92
NH···O angle [°]	133.3	155.4	163.8
C=O···H angle [°]	100.2	102.5	104.3

Computed Energies of **4** / **4'** and UV-Vis/CD Spectra of (*S,S*)-**4**

Table S29 – Computed Gibbs free energies (MeCN, 298 K, 1 M) of (*S,S*)-**4** (denoted *d1*) and its diastereomer (*S,S*)-**4'** (*d2*) based on B3LYP-D3(BJ)/def2-TZVPP/SMD single point calculations.

**Compound**	**B3LYP/SVP**	G_{corr}	**B3LYP/TZVPP/SMD**	ΔG_{corr}	**Δ*G*solv [kJ·mol^{-1}]**
d1_conf1_crystal	-2099.549971	0.682150	-2101.898842	-2101.216692	0.0
d2_conf6	-2099.531926	0.680616	-2101.884620	-2101.204004	33.3
d2_conf33	-2099.530699	0.681180	-2101.883445	-2101.202265	37.9
d2_conf4	-2099.525153	0.679439	-2101.879990	-2101.200551	42.4
d2_conf1	-2099.517173	0.678599	-2101.874472	-2101.195873	54.7

Table S30 – Absorption spectrum via transition electric dipole moments of (*S,S*)-**4** (*d1*).

State	Energy (cm^{-1})	Wavelength (nm)	fosc (au**2)	T2 (au)
**1**	**36032.5**	**277.5**	**0.062**	**0.570**
**2**	**36259.3**	**275.8**	**0.059**	**0.534**
3	36784.0	271.9	0.003	0.031
4	38366.4	260.6	0.020	0.168
5	38998.1	256.4	0.018	0.152
6	39323.3	254.3	0.014	0.116
7	39563.2	252.8	0.003	0.022
8	39983.9	250.1	0.000	0.003
9	40251.5	248.4	0.011	0.092
10	40432.0	247.3	0.011	0.089
11	40793.2	245.1	0.012	0.095
12	41285.2	242.2	0.022	0.174
13	41482.1	241.1	0.024	0.191
14	41569.8	240.6	0.022	0.177
15	41663.4	240.0	0.164	1.292
16	42024.1	238.0	0.008	0.060
17	42299.1	236.4	0.016	0.128
**18**	**42719.9**	**234.1**	**1.290**	**9.944**
19	46490.1	215.1	0.000	0.001
20	46848.3	213.5	0.003	0.023
21	46972.9	212.9	0.001	0.005
22	47568.3	210.2	0.005	0.034
23	48086.8	208.0	0.002	0.012
24	49137.2	203.5	0.003	0.021
25	49242.5	203.1	0.009	0.058

TD-DFT/TDA EXCITED STATES (SINGLETS) of (*S,S*)-**4**:

PBE0/def2-TZVPP/CPCM(MeCN)

STATE 1: E= 0.164176 au 4.467 eV 36032.5 cm**-1 <S**2> = 0.000000

161a -> 172a : 0.014302 (c= 0.11958988)

166a -> 168a : 0.035755 (c= -0.18909077)

167a (HOMO) -> 168a (LUMO): 0.885349 (c= -0.94092997)

167a -> 169a : 0.010493 (c= -0.10243371)

STATE 2: E= 0.165210 au 4.496 eV 36259.3 cm**-1 <S**2> = 0.000000

159a -> 173a : 0.011613 (c= 0.10776570)

166a -> 168a : 0.870142 (c= -0.93281405)

167a -> 168a : 0.037446 (c= 0.19350961)

STATE 18: E= 0.194646 au 5.297 eV 42719.9 cm**-1 <S**2> = 0.000000

159a -> 168a : 0.023918 (c= 0.15465553)

161a -> 169a : 0.014005 (c= -0.11834358)

162a -> 170a : 0.017251 (c= 0.13134180)

165a -> 170a : 0.027748 (c= 0.16657623)

165a -> 171a : 0.089670 (c= 0.29944999)

165a -> 173a : 0.053990 (c= -0.23235764)

166a -> 172a : 0.039388 (c= 0.19846457)

166a -> 173a : 0.503273 (c= 0.70941741)

167a -> 172a : 0.141824 (c= -0.37659486)

167a -> 173a : 0.020858 (c= 0.14442177)

NATURAL TRANSITION ORBITALS FOR STATE 1

E= 0.164176 au 4.467 eV 36032.5 cm**-1

167a -> 168a : n= 0.93921503

166a -> 169a : n= 0.03030682

165a -> 170a : n= 0.01802906

164a -> 171a : n= 0.00807415

NATURAL TRANSITION ORBITALS FOR STATE 2

E= 0.165210 au 4.496 eV 36259.3 cm**-1

167a -> 168a : n= 0.92121376

166a -> 169a : n= 0.04044887

165a -> 170a : n= 0.01375258

164a -> 171a : n= 0.01211821

163a -> 172a : n= 0.00484935

162a -> 173a : n= 0.00346534

NATURAL TRANSITION ORBITALS FOR STATE 18

E= 0.194646 au 5.297 eV 42719.9 cm**-1

167a -> 168a : n= 0.61700100

166a -> 169a : n= 0.17929074

165a -> 170a : n= 0.12401986

164a -> 171a : n= 0.02663160

163a -> 172a : n= 0.01894554

162a -> 173a : n= 0.01445165

161a -> 174a : n= 0.00144768

160a -> 175a : n= 0.00133692

159a -> 176a : n= 0.00105385

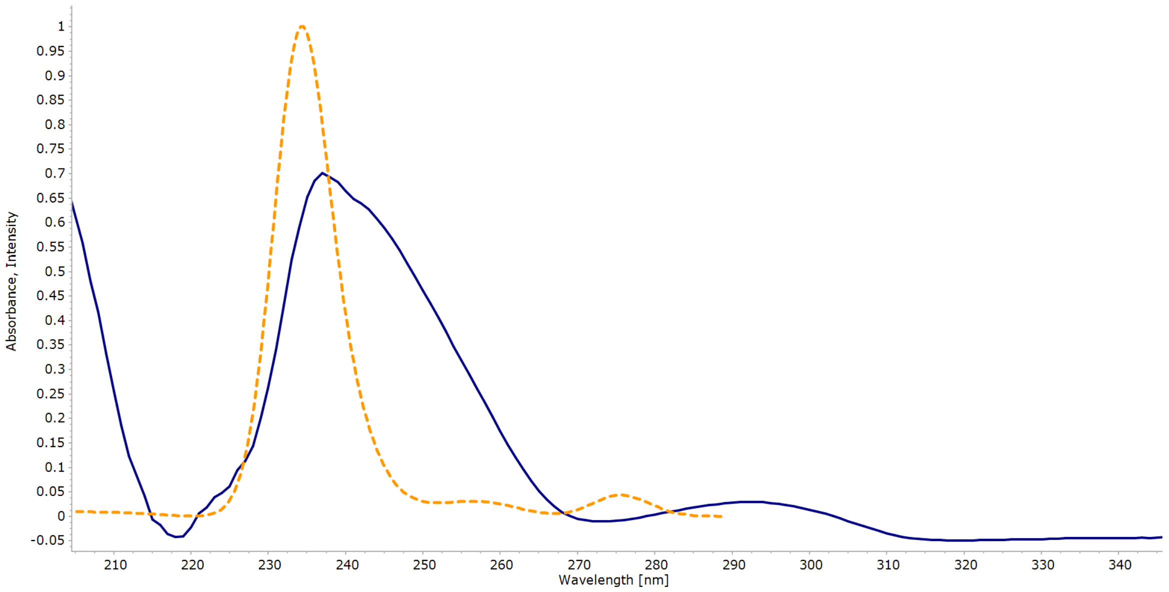

Figure S57 – Comparison of experimental (in MeCN; solid blue line) and computed UV-VIS absorption spectra (PBE0/def2-TZVPP/CPCM(MeCN); unshifted; orange dashed line) for (*S,S*)-**4** (*d1_conf1_crystal)*, showing a good match.

HOMO/LUMO Visualizations for **4**

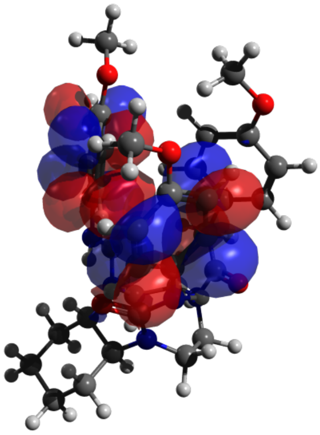

Figure S58 – LUMO+1 orbital of (*S,S*)-**4** (*dl*).

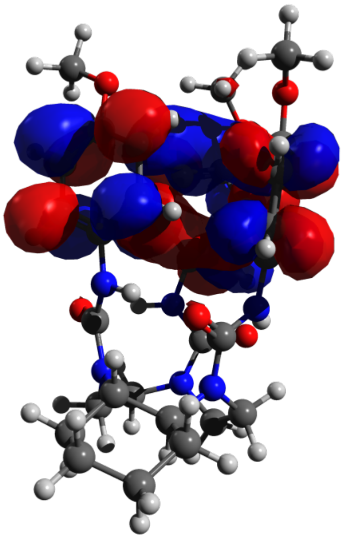

Figure S59 – LUMO orbital of (*S,S*)-**4** (*dl*).

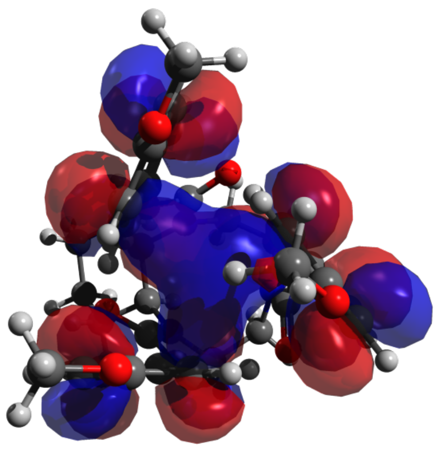

Figure S60 – LUMO orbital of (*S*,*S*)-**4** (*d1*) (viewed from top).

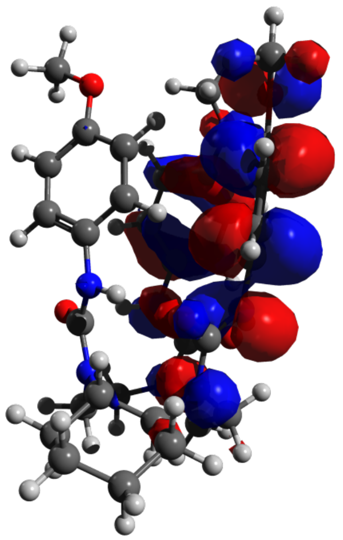

Figure S61 – HOMO orbital of (*S*,*S*)-**4** (*d1*).

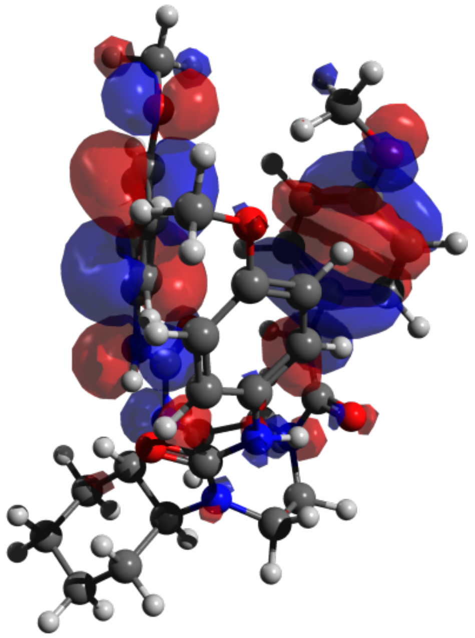

Figure S62 – HOMO-1 orbital of (*S,S*)-**4** (*d1*).

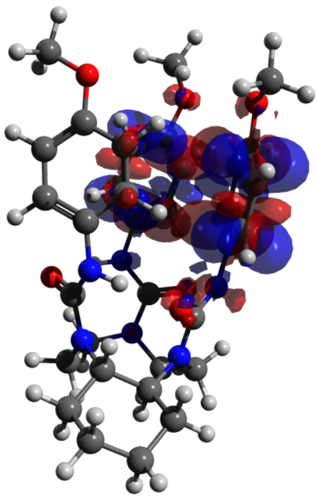

Figure S63 – π-π* Transition density of *State 1* of (*S,S*)-**4** (*d1*).

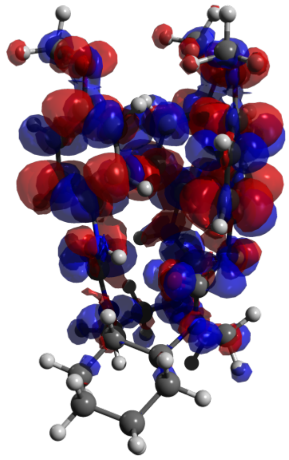

Figure S64 – π-π* Transition density of *State 18* of (*S,S*)-**4** (*d1*).

Computed Energies of **8** / **8'**

Table S31 – Computed Gibbs free energies (MeCN, 298 K, 1 M) of **8** (*d1*) and its diastereomer **8'** (*d2*) based on B3LYP-D3(BJ)/def2-TZVPP/SMD single point calculations.

Compound	B3LYP/SVP	G_{corr}	B3LYP/TZVPP/SMD	ΔG_{corr}	ΔGsolv [kJ·mol^{-1}]
TU_d1_conf3	-3462.909650	0.493053	-3466.272804	-3465.779751	0.0
TU_d1_conf1	-3462.913952	0.495814	-3466.271562	-3465.775748	10.5
TU_d1_conf9	-3462.893019	0.489439	-3466.265158	-3465.775719	10.6
TU_d1_conf21	-3462.903644	0.492257	-3466.266764	-3465.774507	13.8
TU_d2_conf1	-3462.901641	0.492981	-3466.264317	-3465.771336	22.1
TU_d2_conf87	-3462.885632	0.489415	-3466.255654	-3465.766239	35.5
TU_d2_conf13	-3462.875162	0.489362	-3466.250514	-3465.761152	48.8
TU_d2_conf15	-3462.896168	0.494944	-3466.254307	-3465.759363	53.5

Coordinates of Optimized Structures

Method: B3LYP-D3(BJ)/def2-SVP

3 (*d1*)

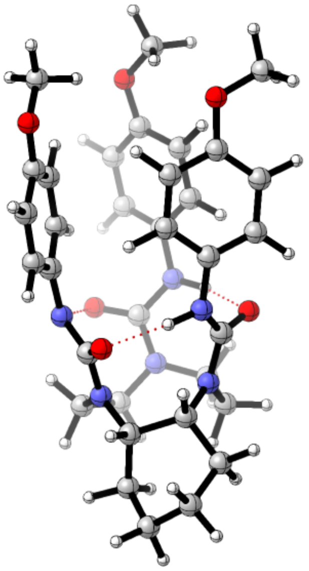

```
C 3.4889120000 -2.2995930000 -0.4249440000
H 2.7532440000 -3.1034030000 -0.3366610000
H 4.4591300000 -2.7661730000 -0.1959350000
C 3.5451760000 -1.8312960000 -1.8954990000
H 4.5239880000 -1.3757780000 -2.0964140000
H 3.4734830000 -2.7359050000 -2.5229170000
C 3.0256980000 0.4144970000 -2.8082490000
H 2.2047300000 0.9471650000 -3.2912490000
H 3.7785670000 0.2271220000 -3.5960490000
C 3.6864460000 1.3156590000 -1.7495240000
H 4.6974890000 0.9406580000 -1.5563140000
H 3.7951990000 2.3205710000 -2.1928670000
C 3.7874460000 1.1702380000 0.7605670000
H 3.1437550000 1.4797250000 1.5869120000
C 4.1661420000 -0.3286980000 1.0713920000
H 4.1150070000 -0.3940960000 2.1678750000
C 5.6143090000 -0.6432060000 0.6900840000
H 5.7851740000 -0.4652620000 -0.3833350000
H 5.8338330000 -1.7057370000 0.8648040000
C 6.5831760000 0.2153730000 1.5332120000
H 6.8463540000 -0.3269950000 2.4553570000
H 7.5232280000 0.3423580000 0.9727530000
C 5.9985230000 1.5946930000 1.9007220000
H 5.4618980000 1.5343260000 2.8625760000
H 6.8096730000 2.3239040000 2.0494740000
C 5.0307570000 2.0813730000 0.8232040000
H 5.5582660000 2.0960590000 -0.1434640000
H 4.7025470000 3.1153980000 1.0111490000
C 1.9740270000 -1.2743760000 1.2650940000
C -0.3121080000 -2.1263200000 1.4323830000
C -0.5411720000 -1.9742720000 2.8103710000
H 0.3003130000 -1.8854220000 3.4942910000
C -1.8426610000 -1.8794450000 3.2926440000
H -2.0356750000 -1.7294350000 4.3564670000
C -2.9399500000 -1.9502170000 2.4194090000
C -2.7158180000 -2.1712310000 1.0554100000
```

H -3.5381680000 -2.2377860000 0.3449870000
C -1.4080700000 -2.2556390000 0.5755960000
H -1.2390040000 -2.3884540000 -0.4909670000
C -5.2963780000 -1.8203620000 2.1294970000
H -5.4047500000 -2.8075200000 1.6451100000
H -5.2487140000 -1.0497510000 1.3405310000
H -6.1740330000 -1.6327150000 2.7623580000
C 1.2255050000 -1.2512790000 -2.2786730000
C -1.1079920000 -0.4792890000 -2.4675810000
C -1.8741330000 0.5953890000 -2.0054150000
H -1.3832490000 1.5362450000 -1.7593550000
C -3.2481480000 0.4679710000 -1.8055590000
H -3.7987270000 1.3157480000 -1.4055140000
C -3.8786070000 -0.7546880000 -2.0592700000
C -3.1256090000 -1.8157050000 -2.5858290000
H -3.6348610000 -2.7556270000 -2.8071710000
C -1.7557890000 -1.6818980000 -2.7979280000
H -1.1770460000 -2.5219870000 -3.1766530000
C -5.9917870000 0.0650460000 -1.3367630000
H -6.9987390000 -0.3428310000 -1.1748690000
H -6.0509900000 0.8805380000 -2.0811230000
H -5.6148620000 0.4893850000 -0.3890380000
C 1.7066950000 1.8396400000 -0.4784740000
C -0.3741350000 2.1227280000 0.7845050000
C -1.2123280000 1.2973560000 1.5529100000
H -0.7895360000 0.4443320000 2.0814210000
C -2.5796250000 1.5318350000 1.6132710000
H -3.2317410000 0.8704890000 2.1851410000
C -3.1448950000 2.5974880000 0.8998270000
C -2.3099260000 3.4589020000 0.1768140000
H -2.7158630000 4.3090900000 -0.3707820000
C -0.9306840000 3.2252080000 0.1283360000
H -0.2943380000 3.8818990000 -0.4619560000
C -5.1242510000 3.7759240000 0.2796600000
H -4.9337220000 3.7590000000 -0.8095100000
H -4.7841400000 4.7482240000 0.6794600000
H -6.2044270000 3.6777000000 0.4530440000
N 3.1741830000 -1.2938700000 0.5759870000
N 2.5459010000 -0.8556810000 -2.2978510000
N 3.0095520000 1.3801940000 -0.4600830000
N 0.9879980000 -2.1399710000 0.8660600000
N 0.2902840000 -0.2907360000 -2.5704750000
N 1.0014230000 1.7946020000 0.6997040000
O 1.8211690000 -0.4848810000 2.2085190000
O -4.1704950000 -1.7717830000 2.9747680000
O 0.9389730000 -2.4201310000 -1.9870030000
O -5.1945560000 -1.0016350000 -1.7998390000
O 1.2146270000 2.2460010000 -1.5398760000
O -4.5079650000 2.6954850000 0.9378830000
H 1.0082960000 -2.4693690000 -0.1032560000
H 1.3113950000 1.1154830000 1.4024090000
H 0.5751200000 0.6817720000 -2.4269120000

3' (*d2*)

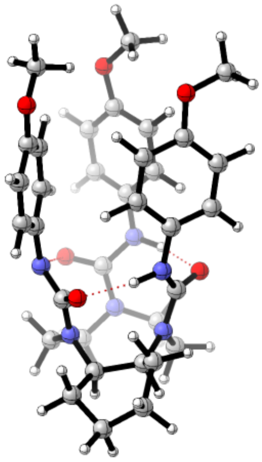

C -3.5237850000 1.5870070000 1.8538560000
H -2.7888240000 1.8264260000 2.6258270000
H -4.5047630000 1.5663710000 2.3612610000
C -3.5639210000 2.7252210000 0.8105310000
H -4.5354580000 2.7149650000 0.2968260000
H -3.5006040000 3.6779890000 1.3638270000
C -2.9965110000 2.6642040000 -1.6108690000
H -2.1494390000 2.8913030000 -2.2613750000
H -3.7234610000 3.4843200000 -1.7564320000
C -3.6742070000 1.3594000000 -2.0755000000
H -4.7195140000 1.3620260000 -1.7392120000
H -3.6882240000 1.3736580000 -3.1787470000
C -4.0216350000 -0.7931920000 -0.9106250000
C -4.3238400000 -0.3942140000 0.5830300000
C -2.0831520000 -0.4310060000 1.5912570000
C 0.1808450000 -0.3640770000 2.5038230000
C 0.3269060000 -1.6903730000 2.9431390000
H -0.5543610000 -2.2974500000 3.1412370000
C 1.5958740000 -2.2449060000 3.0718620000
H 1.7248390000 -3.2834980000 3.3819000000
C 2.7431340000 -1.4876500000 2.7842110000
C 2.6025130000 -0.1457450000 2.4114960000
H 3.4659300000 0.4769270000 2.1825770000
C 1.3258490000 0.4014210000 2.2724060000
H 1.2217220000 1.4327050000 1.9416170000
C 5.1100210000 -1.4239000000 2.5620720000
H 5.2632960000 -0.5698980000 3.2461180000
H 5.0956810000 -1.0426010000 1.5261100000
H 5.9456790000 -2.1276170000 2.6735960000
C -1.2298930000 2.7563240000 0.1649900000
C 1.1097450000 2.5255930000 -0.5703720000
C 1.8560570000 1.6407440000 -1.3547940000
H 1.3570520000 1.0637330000 -2.1321250000
C 3.2170050000 1.4450260000 -1.1245960000
H 3.7492320000 0.7162190000 -1.7307890000
C 3.8541380000 2.1346780000 -0.0875820000
C 3.1254950000 3.0758700000 0.6561010000
H 3.6413940000 3.6294740000 1.4429240000
C 1.7688650000 3.2794370000 0.4156930000
H 1.2071630000 3.9870070000 1.0221090000
C 5.9262950000 1.0375630000 -0.4901280000
H 6.9186720000 0.9986010000 -0.0208930000

H 6.0368960000 1.3882350000 -1.5329110000
H 5.4942070000 0.0211400000 -0.5119270000
C -1.7753230000 -0.1052690000 -1.9638960000
C 0.2492390000 -1.4541740000 -1.6492170000
C 1.0337560000 -1.8893160000 -0.5672700000
H 0.5733330000 -2.0210950000 0.4106310000
C 2.3954180000 -2.1131500000 -0.7216850000
H 3.0074350000 -2.4202970000 0.1271960000
C 3.0093600000 -1.9029780000 -1.9638720000
C 2.2248470000 -1.5278170000 -3.0618380000
H 2.6677390000 -1.3789060000 -4.0463330000
C 0.8506500000 -1.3132300000 -2.9043410000
H 0.2555730000 -0.9892940000 -3.7560170000
C 5.0286840000 -1.9043570000 -3.2338900000
H 4.9072270000 -0.8865220000 -3.6489010000
H 4.6675200000 -2.6326370000 -3.9822760000
H 6.0954500000 -2.0863470000 -3.0456970000
N -3.2389470000 0.2655140000 1.3184440000
N -2.5486430000 2.6659970000 -0.2296850000
N -3.0855240000 0.1149870000 -1.5845120000
N -1.0870750000 0.2271940000 2.2681240000
N -0.2816760000 2.6024080000 -0.8144940000
N -1.1177440000 -1.1725900000 -1.4165380000
O -1.9608790000 -1.6087360000 1.2235500000
O 3.9358230000 -2.1388220000 2.8697090000
O -0.9566150000 2.9467780000 1.3573930000
O 5.1523530000 1.9317570000 0.2775040000
O -1.2362150000 0.6822100000 -2.7560080000
O 4.3682060000 -2.0513210000 -2.0000770000
H -1.0561890000 1.2472200000 2.1818720000
H -1.4751930000 -1.5259870000 -0.5242200000
H -0.5700590000 2.0925960000 -1.6542890000
H -5.1000720000 0.3824200000 0.5159060000
C -4.9459850000 -1.5796260000 1.3449390000
C -3.8265850000 -2.3043790000 -1.0933470000
H -4.9750150000 -0.6015190000 -1.4287570000
H -3.0352090000 -2.6797630000 -0.4401390000
H -3.5332460000 -2.5171640000 -2.1316170000
C -5.1474430000 -3.0225530000 -0.7475290000
H -4.1400780000 -2.2350960000 1.6997010000
C -5.8960560000 -2.3634670000 0.4363400000
H -5.4745450000 -1.1974720000 2.2330770000
H -4.9190720000 -4.0730680000 -0.5092260000
H -5.8086470000 -3.0437730000 -1.6298210000
H -6.6740230000 -1.6774880000 0.0558090000
H -6.4323280000 -3.1279780000 1.0192870000

(*S*,*S*)-4 (*d1*)

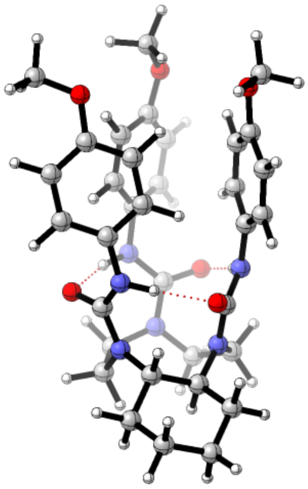

C 3.7446140000 -1.1786570000 1.9263950000
H 3.8608330000 -1.1892440000 3.0233740000
H 4.7551270000 -1.1408880000 1.5008380000
C 3.0790400000 -2.5106460000 1.5230970000
H 3.8321770000 -3.3092250000 1.6546100000
H 2.2664460000 -2.7380080000 2.2161640000
C 3.5356810000 -2.6042810000 -0.9237000000
H 3.4609850000 -3.5616540000 -1.4666790000
H 4.5311180000 -2.5736850000 -0.4590680000
C 3.4182150000 -1.4723590000 -1.9697440000
H 4.3767080000 -1.4226310000 -2.5177200000
H 2.6584170000 -1.7353170000 -2.7095020000
C 4.2034170000 0.5013080000 -0.6921720000
H 4.9585240000 -0.2852070000 -0.5324200000
C 3.8031500000 1.0169610000 0.7121110000
H 3.1488210000 1.8770110000 0.5627130000
C 5.0427760000 1.5488920000 1.4502690000
H 4.7307730000 1.9148840000 2.4412270000
H 5.7668770000 0.7339620000 1.6249140000
C 5.7216510000 2.6611110000 0.6447500000
H 6.6122580000 3.0260890000 1.1813460000
H 5.0313380000 3.5189210000 0.5603030000
C 6.0997790000 2.1786260000 -0.7586640000
H 6.5505190000 2.9994900000 -1.3392930000
H 6.8747790000 1.3942220000 -0.6745790000
C 4.8844850000 1.6166860000 -1.5009300000
H 5.1807110000 1.2140140000 -2.4833200000
H 4.1435400000 2.4087050000 -1.6849850000
C 1.7693410000 0.2046370000 1.9494350000
C -0.2997440000 1.5006980000 1.7682170000
C -1.1595660000 1.8905620000 0.7277950000
H -0.7585870000 2.0370970000 -0.2738770000
C -2.5221460000 2.0323880000 0.9488260000
H -3.1964440000 2.2837750000 0.1294110000
C -3.0621820000 1.7845650000 2.2188980000
C -2.2041290000 1.4510520000 3.2746360000
H -2.5900770000 1.2691470000 4.2772240000
C -0.8287450000 1.3195700000 3.0499450000
H -0.1760970000 1.0233840000 3.8687020000
C -5.0187110000 1.5928880000 3.5668970000
H -6.1032220000 1.6934980000 3.4251110000
H -4.6904000000 2.3133520000 4.3379620000

H -4.7969570000 0.5702430000 3.9233810000
C 1.2412770000 -2.6917900000 -0.1762830000
C -1.0813670000 -2.5520120000 0.5754440000
C -1.8692240000 -1.5876510000 1.2088490000
H -1.4086180000 -0.8913310000 1.9075560000
C -3.2297890000 -1.4669610000 0.9203350000
H -3.7979630000 -0.6682890000 1.3918140000
C -3.8198120000 -2.3187170000 -0.0192780000
C -3.0479660000 -3.3373810000 -0.6014970000
H -3.5282800000 -4.0103240000 -1.3142540000
C -1.6947200000 -3.4598970000 -0.3043330000
H -1.0978750000 -4.2287120000 -0.7918380000
C -5.8619990000 -1.0971190000 -0.0124520000
H -5.3871790000 -0.1435420000 -0.3034370000
H -6.0086560000 -1.1007050000 1.0827090000
H -6.8418020000 -1.1749540000 -0.5022890000
C 1.9373170000 0.5189430000 -1.6335790000
C -0.3864950000 0.3498210000 -2.4193350000
C -1.4749860000 -0.5263600000 -2.2662730000
H -1.2909480000 -1.5753260000 -2.0366330000
C -2.7790920000 -0.0618250000 -2.3600000000
H -3.6223140000 -0.7374040000 -2.2165420000
C -3.0310620000 1.2967950000 -2.5982450000
C -1.9531180000 2.1636670000 -2.8171390000
H -2.1145830000 3.2195350000 -3.0328750000
C -0.6381340000 1.6885090000 -2.7385610000
H 0.1914560000 2.3778240000 -2.8778720000
C -4.6519050000 3.0325370000 -2.7995710000
H -5.7441450000 3.1219120000 -2.7276550000
H -4.3301940000 3.3673350000 -3.8025810000
H -4.1885820000 3.6924670000 -2.0432280000
N 3.0569490000 0.0284000000 1.4887480000
N 2.5703150000 -2.5545270000 0.1641580000
N 3.1185010000 -0.1612150000 -1.4211180000
N 1.0653620000 1.2826290000 1.4641250000
H 1.3444400000 1.6412910000 0.5464040000
N 0.3146490000 -2.5642880000 0.8283390000
H 0.5944620000 -2.0327530000 1.6572430000
N 0.9151270000 -0.1754200000 -2.2310560000
H 0.9336000000 -1.1964630000 -2.1504580000
O 1.2877750000 -0.6049260000 2.7521260000
O -4.4215580000 1.8516510000 2.3189330000
O 0.9347870000 -2.9027180000 -1.3576820000
O -5.1077650000 -2.2087690000 -0.4459370000
O 1.8206490000 1.7008370000 -1.2770830000
O -4.3404390000 1.6790600000 -2.5740000000

(*S*,*S*)-4' (*d2*)

Conf4

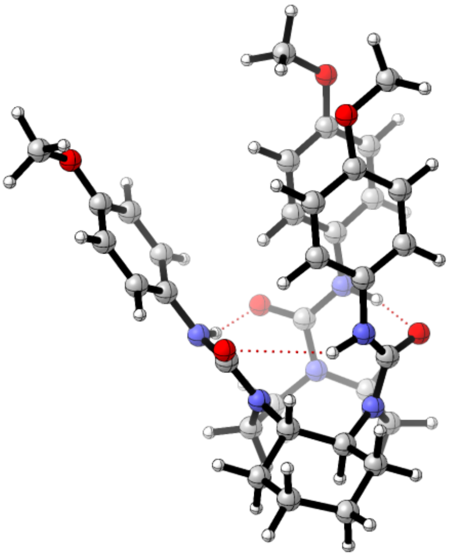

```
C 3.2578250000 1.7152960000 1.5265800000
H 3.1086500000 2.7789770000 1.7774390000
H 4.3388340000 1.5883700000 1.3822830000
C 2.8323170000 0.8829800000 2.7836560000
H 3.7288930000 0.7478600000 3.4092390000
H 2.1159740000 1.4686120000 3.3722230000
C 3.0716110000 -1.6004600000 2.6394340000
H 2.4272450000 -2.4780460000 2.7367370000
H 3.6714960000 -1.5498740000 3.5670540000
C 4.0480710000 -1.8055680000 1.4574580000
H 4.9673970000 -1.2334500000 1.6379250000
H 4.3250630000 -2.8719950000 1.4294890000
C 4.2641800000 -0.3292050000 -0.5546810000
H 5.0892430000 -0.0651620000 0.1226760000
C 3.5098090000 0.9828890000 -0.8487730000
H 2.8407010000 0.8459500000 -1.7053180000
C 4.5068000000 2.0449240000 -1.3428970000
H 3.9540550000 2.9771780000 -1.5320730000
H 5.2583760000 2.2584160000 -0.5617370000
C 5.2022430000 1.5644110000 -2.6213400000
H 5.9344620000 2.3135610000 -2.9629320000
H 4.4471280000 1.4784480000 -3.4226340000
C 5.8838060000 0.2068660000 -2.4209260000
H 6.3116570000 -0.1527830000 -3.3704550000
H 6.7329830000 0.3276860000 -1.7236410000
C 4.9213200000 -0.8399860000 -1.8484160000
H 5.4531480000 -1.7791440000 -1.6297470000
H 4.1372910000 -1.0841920000 -2.5849440000
C 1.4454820000 2.0138640000 -0.2001680000
C -0.6678350000 3.0346360000 0.5311880000
C -1.6746370000 2.8385540000 1.4958210000
H -1.4541500000 2.2410160000 2.3813700000
C -2.9451840000 3.3704130000 1.3143360000
H -3.7292660000 3.2134840000 2.0573080000
C -3.2500890000 4.1124410000 0.1627660000
C -2.2500840000 4.3163890000 -0.7966410000
H -2.4498500000 4.8886950000 -1.7023170000
C -0.9697970000 3.7832040000 -0.6124490000
H -0.2096780000 3.9349370000 -1.3746570000
```

C -4.8797700000 5.3365030000 -1.0673580000
H -5.9355560000 5.6138020000 -0.9443220000
H -4.7696960000 4.7542570000 -2.0009730000
H -4.2766940000 6.2588320000 -1.1579750000
C 0.8483930000 -0.4422990000 2.4692110000
C -1.1220130000 -1.7306950000 1.7676650000
C -1.8412940000 -0.7181510000 1.1279370000
H -1.3574730000 0.2224270000 0.8827380000
C -3.1767310000 -0.9051040000 0.7736020000
H -3.6960200000 -0.0951750000 0.2638670000
C -3.8070770000 -2.1288300000 1.0308130000
C -3.0881530000 -3.1506800000 1.6693950000
H -3.5919920000 -4.0983810000 1.8682090000
C -1.7630680000 -2.9473020000 2.0411910000
H -1.2063700000 -3.7432390000 2.5410080000
C -5.8442180000 -1.4249690000 0.0173180000
H -5.3783370000 -1.1421960000 -0.9416800000
H -5.9655150000 -0.5219360000 0.6420050000
H -6.8343140000 -1.8606960000 -0.1735160000
C 2.3706270000 -2.0052720000 -0.2341330000
C 0.3094660000 -1.7490390000 -1.5879790000
C -0.4772000000 -0.7036910000 -2.1049770000
H -0.0466530000 0.2942410000 -2.2206400000
C -1.8132130000 -0.9165130000 -2.4169730000
H -2.4326030000 -0.1031780000 -2.7988240000
C -2.4024940000 -2.1713700000 -2.2038800000
C -1.6191800000 -3.2145240000 -1.6992370000
H -2.0499340000 -4.1963650000 -1.5087760000
C -0.2689860000 -3.0077830000 -1.4007280000
H 0.3261630000 -3.8196660000 -0.9916460000
C -4.3528030000 -3.5355840000 -2.3570930000
H -5.3908920000 -3.4070750000 -2.6921160000
H -4.3600530000 -3.8759360000 -1.3083840000
H -3.8644620000 -4.2988830000 -2.9889720000
N 2.6389470000 1.4278520000 0.2374710000
N 2.2375340000 -0.4169910000 2.5491760000
N 3.5234780000 -1.3629260000 0.1709590000
N 0.6087720000 2.4735640000 0.7829890000
H 0.6469940000 1.9726290000 1.6776040000
N 0.2616100000 -1.6068150000 2.0740990000
H 0.8506790000 -2.3262470000 1.6443450000
N 1.6593490000 -1.4497870000 -1.2642290000
H 1.9409300000 -0.5260390000 -1.5608550000
O 1.1935080000 2.0933850000 -1.4023170000
O -4.5236020000 4.5848250000 0.0658950000
O 0.2002060000 0.5754900000 2.7385250000
O -5.0908000000 -2.4179470000 0.6808280000
O 1.9927960000 -3.0169840000 0.3687830000
O -3.7301620000 -2.2765280000 -2.4924580000

(*S*,*S*)-4' (*d2*)

Conf6

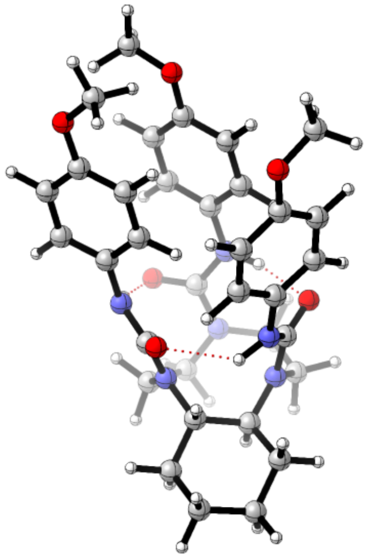

C 3.1452450000 -2.4724870000 0.5594260000
H 2.4014880000 -3.2231030000 0.8401460000
H 4.0695890000 -2.7580080000 1.0894050000
C 3.4409140000 -2.6056830000 -0.9577590000
H 4.4422910000 -2.2082390000 -1.1719330000
H 3.4737540000 -3.6867550000 -1.1831220000
C 3.0694320000 -1.0908240000 -2.9023110000
H 2.2478800000 -0.7675590000 -3.5478210000
H 3.7391910000 -1.7048120000 -3.5340880000
C 3.8613390000 0.1428220000 -2.4111710000
H 4.9183750000 -0.1177050000 -2.2720410000
H 3.8156660000 0.9106030000 -3.1973460000
C 4.2712670000 0.5312310000 0.0158180000
H 5.0412630000 -0.1698480000 -0.3361280000
C 3.6654600000 -0.1142590000 1.2866420000
H 3.0947350000 0.6439940000 1.8333070000
C 4.8116730000 -0.4907530000 2.2390700000
H 4.3860630000 -0.9848120000 3.1263500000
H 5.4972450000 -1.2131280000 1.7608880000
C 5.5930840000 0.7648490000 2.6445370000
H 6.4258190000 0.4960900000 3.3142560000
H 4.9254610000 1.4254780000 3.2251680000
C 6.1198440000 1.5249550000 1.4226910000
H 6.6088330000 2.4616700000 1.7349150000
H 6.8998420000 0.9171090000 0.9286930000
C 5.0112000000 1.8217250000 0.4064400000
H 5.4317170000 2.2830930000 -0.5011230000
H 4.2893050000 2.5443770000 0.8225520000
C 1.4163340000 -0.9413070000 1.5689410000
C -0.9169770000 -1.6963090000 1.6238740000
C -1.6814700000 -2.7883600000 2.0558470000
H -1.1902840000 -3.7475840000 2.2342850000
C -3.0552460000 -2.6612130000 2.2447900000
H -3.6615880000 -3.5081280000 2.5713340000
C -3.6878960000 -1.4290320000 2.0228110000
C -2.9248560000 -0.3313640000 1.6051400000
H -3.3824130000 0.6346650000 1.3967630000
C -1.5554720000 -0.4763360000 1.3942630000
H -0.9881640000 0.3710740000 1.0251100000

C -5.7016020000 -0.1569640000 2.0572790000
H -6.7617440000 -0.3432360000 2.2765080000
H -5.6050810000 0.2289640000 1.0286300000
H -5.3166110000 0.5997190000 2.7641210000
C 1.1741390000 -2.1781610000 -1.6822230000
C -1.0888330000 -1.1983810000 -1.9575850000
C -1.8666480000 -2.3164910000 -1.6367400000
H -1.3949100000 -3.2897040000 -1.5336420000
C -3.2389350000 -2.1799620000 -1.4085280000
H -3.8086090000 -3.0647600000 -1.1283860000
C -3.8555260000 -0.9278760000 -1.5010570000
C -3.0798530000 0.1901650000 -1.8401920000
H -3.5604700000 1.1683890000 -1.8974210000
C -1.7163770000 0.0563080000 -2.0632530000
H -1.1158430000 0.9379390000 -2.2910360000
C -6.0108080000 -1.8086840000 -0.9858430000
H -5.7264040000 -2.3093510000 -0.0451710000
H -5.9930010000 -2.5424320000 -1.8115670000
H -7.0308480000 -1.4141740000 -0.8827340000
C 2.1790700000 1.3485250000 -1.2279040000
C 0.4200080000 2.6102240000 -0.0336710000
C -0.2237630000 2.6664270000 1.2164080000
H 0.2426210000 2.1907940000 2.0793430000
C -1.4760600000 3.2522880000 1.3453830000
H -1.9867960000 3.2702870000 2.3096650000
C -2.1256100000 3.8013920000 0.2295380000
C -1.4683830000 3.7929960000 -1.0069670000
H -1.9305550000 4.2299010000 -1.8918380000
C -0.2028660000 3.2098920000 -1.1352020000
H 0.2861900000 3.1949900000 -2.1050250000
C -4.0845950000 4.8235140000 -0.6548310000
H -5.0617640000 5.1472370000 -0.2719580000
H -4.2421430000 4.0610840000 -1.4398350000
H -3.5729910000 5.6926380000 -1.1074620000
N 2.6969100000 -1.1753640000 1.0418340000
N 2.5209510000 -1.9071440000 -1.8378240000
N 3.3873360000 0.6909140000 -1.1437170000
N 0.4644110000 -1.8913330000 1.3401340000
H 0.6321340000 -2.5960220000 0.6154380000
N 0.3096410000 -1.2352190000 -2.1630850000
H 0.7110580000 -0.3242400000 -2.4001100000
N 1.6739600000 1.9506000000 -0.1026050000
H 1.9872400000 1.6007640000 0.7967010000
O 1.1854810000 0.0908340000 2.2018170000
O -5.0372740000 -1.3937310000 2.2118650000
O 0.8225860000 -3.1968690000 -1.0738170000
O -5.1791730000 -0.7016600000 -1.2595650000
O 1.5906150000 1.3833140000 -2.3179440000
O -3.3714090000 4.3054060000 0.4415320000

(*S*,*S*)-4' (*d2*)

Conf33

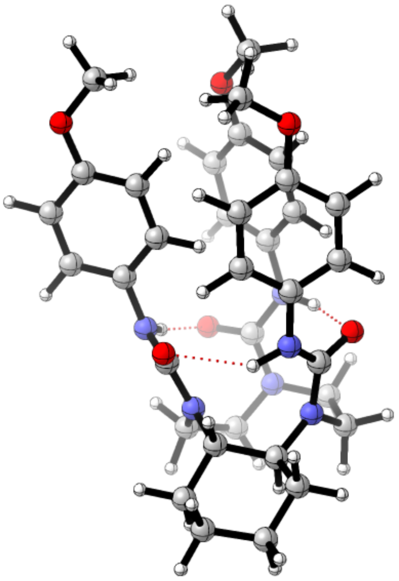

C -3.2438100000 -2.3995930000 0.1034880000
H -2.5341370000 -3.2303160000 0.0763200000
H -4.1703670000 -2.7820740000 -0.3580080000
C -3.5846580000 -2.0824390000 1.5832060000
H -4.5801570000 -1.6211750000 1.6373950000
H -3.6572990000 -3.0527030000 2.1067570000
C -3.2518650000 -0.0811480000 3.0232590000
H -2.4607630000 0.3822210000 3.6195520000
H -3.9901110000 -0.4771220000 3.7454750000
C -3.9495220000 0.9931000000 2.1532700000
H -5.0130380000 0.7496560000 2.0348340000
H -3.8911470000 1.9531170000 2.6861300000
C -4.2322920000 0.6690500000 -0.2993660000
H -5.0491520000 0.1253620000 0.1962930000
C -3.6071260000 -0.3313600000 -1.2986150000
H -2.9736000000 0.2172490000 -2.0037730000
C -4.7270560000 -0.9191730000 -2.1729420000
H -4.2863200000 -1.6589650000 -2.8591090000
H -5.4693100000 -1.4516840000 -1.5516890000
C -5.4236540000 0.1969590000 -2.9601000000
H -6.2379290000 -0.2200130000 -3.5743460000
H -4.6971600000 0.6410850000 -3.6630910000
C -5.9648790000 1.2903210000 -2.0331270000
H -6.3902080000 2.1182920000 -2.6227140000
H -6.7966250000 0.8762970000 -1.4341790000
C -4.8888070000 1.8207120000 -1.0791140000
H -5.3249320000 2.5353190000 -0.3631770000
H -4.1114630000 2.3680790000 -1.6384440000
C -1.4261230000 -1.3580800000 -1.2781960000
C 0.8229930000 -2.2765390000 -1.1634340000
C 1.6371670000 -1.1755220000 -0.9003800000
H 1.2150540000 -0.2914220000 -0.4291860000
C 2.9980210000 -1.2003910000 -1.2009580000
H 3.6016820000 -0.3274870000 -0.9623820000
C 3.5685410000 -2.3549430000 -1.7547570000
C 2.7515520000 -3.4639510000 -2.0301390000
H 3.2114970000 -4.3528500000 -2.4655060000
C 1.3906940000 -3.4195460000 -1.7402450000
H 0.7532340000 -4.2810610000 -1.9496080000

C 5.7688790000 -1.4654420000 -1.6296370000
H 5.5727730000 -0.5148020000 -2.1609870000
H 6.7832220000 -1.8038660000 -1.8809650000
H 5.6972840000 -1.2848580000 -0.5431100000
C -1.3316470000 -1.4929570000 2.2602560000
C 0.9275970000 -0.4723030000 2.3983990000
C 1.5345550000 0.7681610000 2.1631610000
H 0.9170740000 1.6654510000 2.1229780000
C 2.9058600000 0.8710170000 1.9315080000
H 3.3258310000 1.8526930000 1.7176290000
C 3.6978250000 -0.2829760000 1.9380460000
C 3.1003360000 -1.5221160000 2.2068680000
H 3.7293090000 -2.4139430000 2.2043800000
C 1.7308750000 -1.6250020000 2.4356430000
H 1.2743030000 -2.5963310000 2.6088610000
C 5.6797980000 0.9501730000 1.4725570000
H 5.2549100000 1.5145410000 0.6231610000
H 6.7361190000 0.7302800000 1.2666970000
H 5.6130190000 1.5856660000 2.3739040000
C -2.1541820000 1.7323820000 0.7700480000
C -0.2233440000 2.4338180000 -0.5814460000
C 0.2916730000 3.4434360000 0.2516490000
H -0.3385430000 3.8860430000 1.0192680000
C 1.6181380000 3.8423650000 0.1279350000
H 2.0347380000 4.6056240000 0.7879760000
C 2.4569160000 3.2617450000 -0.8361350000
C 1.9257870000 2.3135640000 -1.7161150000
H 2.5378390000 1.8454090000 -2.4858620000
C 0.5964780000 1.9036970000 -1.5827980000
H 0.2088710000 1.1151340000 -2.2283730000
C 4.6111250000 3.1830760000 -1.8464530000
H 5.5905650000 3.6537630000 -1.6869210000
H 4.2416150000 3.4511520000 -2.8525370000
H 4.7313120000 2.0848370000 -1.7999750000
N -2.7093770000 -1.3189050000 -0.7114590000
N -2.6775280000 -1.1792730000 2.2699750000
N -3.3906890000 1.1272410000 0.8114030000
N -0.5529740000 -2.2793250000 -0.7770220000
H -0.7183900000 -2.6398830000 0.1700240000
N -0.4764790000 -0.4671320000 2.5592430000
H -0.8720720000 0.4755580000 2.5162130000
N -1.5456170000 1.9282440000 -0.4470730000
H -1.8256410000 1.3285660000 -1.2163300000
O -1.1236340000 -0.5856800000 -2.1901120000
O 4.8892190000 -2.4867380000 -2.0434730000
O -0.9648060000 -2.6308270000 1.9444630000
O 5.0368630000 -0.2887750000 1.6621670000
O -1.6246320000 2.0866050000 1.8328140000
O 3.7609500000 3.6632460000 -0.8322430000

(*S*,*S*)-4' (*d2*)

Conf1

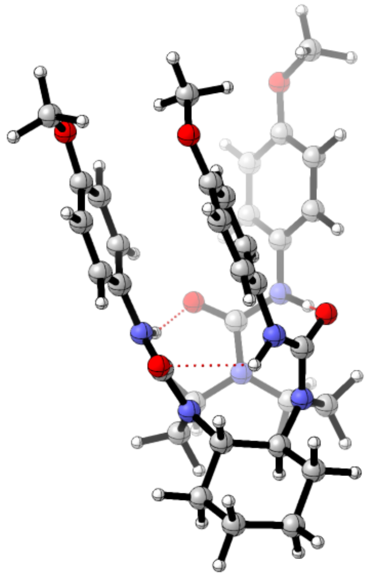

C -3.3739500000 0.1114110000 2.2603120000
H -3.4320740000 -0.5132950000 3.1711550000
H -4.4045050000 0.4317180000 2.0574880000
C -2.5478690000 1.3593420000 2.6296760000
H -3.1974870000 2.0032970000 3.2499310000
H -1.7009360000 1.0835660000 3.2652600000
C -2.8945410000 3.0382740000 0.8175320000
H -2.4682920000 4.0550380000 0.8246290000
H -3.8170290000 3.0821990000 1.4155140000
C -3.2704690000 2.6865070000 -0.6595020000
H -4.3161270000 2.9854930000 -0.8295120000
H -2.6544530000 3.2745010000 -1.3471450000
C -4.2523420000 0.4123410000 -0.7302470000
H -4.9026250000 1.0216670000 -0.0817570000
C -3.9594450000 -0.8818480000 0.0598920000
H -3.5170510000 -1.6345850000 -0.6003250000
C -5.2836960000 -1.5274540000 0.4981810000
H -5.0531730000 -2.4350590000 1.0767260000
H -5.8487130000 -0.8504690000 1.1633580000
C -6.1354630000 -1.8685280000 -0.7299530000
H -7.0938220000 -2.3140390000 -0.4184290000
H -5.6115790000 -2.6388390000 -1.3231770000
C -6.3825230000 -0.6359890000 -1.6063800000
H -6.9378070000 -0.9155750000 -2.5160310000
H -7.0257070000 0.0751560000 -1.0564070000
C -5.0767300000 0.0729850000 -1.9830130000
H -5.2842350000 0.9998260000 -2.5409860000
H -4.4704360000 -0.5657680000 -2.6489260000
C -1.8732940000 -1.5538310000 1.0547460000
C 0.4808370000 -1.8509880000 1.7471080000
C 1.5922060000 -1.1246400000 2.2136550000
H 1.4465180000 -0.1179740000 2.6065900000
C 2.8737660000 -1.6517160000 2.1185010000
H 3.7383890000 -1.0795710000 2.4603610000
C 3.0828460000 -2.9171450000 1.5511270000
C 1.9787870000 -3.6527250000 1.1112320000
H 2.1033210000 -4.6356770000 0.6589340000
C 0.6875600000 -3.1271220000 1.2106720000
H -0.1553420000 -3.6947810000 0.8308670000
C 4.6339670000 -4.5541270000 0.7898080000
H 5.7255880000 -4.6752230000 0.7645250000

H 4.2426300000 -4.5473780000 -0.2425710000
H 4.1941500000 -5.4099860000 1.3336840000
C -0.6104950000 2.2387180000 1.5053130000
C 1.3192070000 3.1361130000 0.3088770000
C 1.8298720000 3.0645440000 -0.9914950000
H 1.1466310000 2.8867780000 -1.8233670000
C 3.2029310000 3.1729860000 -1.2327240000
H 3.5620330000 3.1018840000 -2.2588620000
C 4.0887010000 3.3494400000 -0.1621880000
C 3.5750320000 3.4324430000 1.1424710000
H 4.2767540000 3.5754350000 1.9662680000
C 2.2094790000 3.3316220000 1.3784460000
H 1.8289770000 3.3845630000 2.3967030000
C 6.0092470000 3.3333500000 -1.5670950000
H 5.6594980000 4.1297440000 -2.2495090000
H 5.7857330000 2.3540270000 -2.0296950000
H 7.0961980000 3.4270580000 -1.4398290000
C -1.8757660000 0.9638790000 -1.5529810000
C -0.2459970000 -0.8596390000 -1.8547400000
C -0.0278390000 -2.0802990000 -2.5082240000
H -0.8637850000 -2.5877010000 -2.9951220000
C 1.2372380000 -2.6590160000 -2.5252100000
H 1.4121260000 -3.6141710000 -3.0234010000
C 2.3200550000 -2.0172710000 -1.9050000000
C 2.1066330000 -0.7976200000 -1.2536150000
H 2.9112250000 -0.2821430000 -0.7325980000
C 0.8302050000 -0.2384210000 -1.2170540000
H 0.6858300000 0.6873570000 -0.6723810000
C 4.6742950000 -1.9337970000 -1.5534640000
H 5.5396610000 -2.5610040000 -1.8075340000
H 4.6734420000 -1.7582310000 -0.4660110000
H 4.7632420000 -0.9661890000 -2.0793350000
N -2.9737280000 -0.6873340000 1.1136670000
N -1.9990340000 2.1344510000 1.5282790000
N -3.1112360000 1.2874450000 -1.0131500000
N -0.7894340000 -1.2193580000 1.8216710000
H -0.7321920000 -0.2713770000 2.1867370000
N -0.0820200000 3.0126930000 0.5002880000
H -0.5938560000 2.9551220000 -0.3857240000
N -1.5745640000 -0.3500110000 -1.7785340000
H -2.2655900000 -1.0535490000 -1.5599920000
O -1.8968370000 -2.5291080000 0.3081320000
O 4.3787280000 -3.3324460000 1.4449140000
O 0.0781260000 1.6612370000 2.3475960000
O 5.4399070000 3.4496840000 -0.2866990000
O -1.0616340000 1.8606920000 -1.8000090000
O 3.5280000000 -2.6435250000 -1.9756350000

8 (*d1*)

conf1

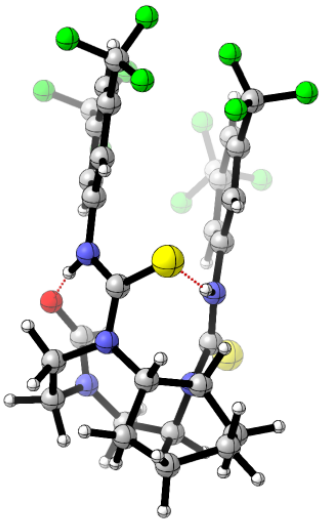

C 3.4449980000 -1.0712720000 -2.3520700000
H 2.5775240000 -1.2008970000 -3.0024670000
H 4.1718000000 -1.8353450000 -2.6721630000
C 4.0997710000 0.2911600000 -2.6495210000
H 5.1494200000 0.2748510000 -2.3333290000
H 4.1007960000 0.3983050000 -3.7483230000
C 4.3354250000 2.3261190000 -1.2092520000
H 3.7820010000 3.2513610000 -1.0174060000
H 5.2623740000 2.5924290000 -1.7449200000
C 4.7131070000 1.6714820000 0.1306030000
H 5.5456140000 0.9799670000 -0.0346400000
H 5.0692150000 2.4667750000 0.8061690000
C 3.9491890000 -0.4682230000 1.1952440000
H 3.1165880000 -0.7849550000 1.8312000000
C 4.0370830000 -1.5619930000 0.0761900000
H 3.7167330000 -2.4849550000 0.5802180000
C 5.4715980000 -1.8241130000 -0.3846510000
H 5.9512320000 -0.9102780000 -0.7664070000
H 5.4811790000 -2.5480400000 -1.2114480000
C 6.2888890000 -2.3947990000 0.8007570000
H 6.2762240000 -3.4952490000 0.7606160000
H 7.3420080000 -2.0942620000 0.6826700000
C 5.7632730000 -1.9308180000 2.1743930000
H 4.9729930000 -2.6130580000 2.5293550000
H 6.5689070000 -1.9826520000 2.9223880000
C 5.2021570000 -0.5106520000 2.0978610000
H 5.9881430000 0.1551580000 1.7105300000
H 4.9372040000 -0.1252470000 3.0939890000
C 1.7282580000 -1.6938380000 -0.6806770000
C -0.6205680000 -1.3079090000 -1.4203210000
C -1.2995750000 -2.4582850000 -1.0042760000
H -0.7499830000 -3.3600790000 -0.7517540000
C -2.6902330000 -2.4300930000 -0.8826510000
C -3.4164710000 -3.6825850000 -0.4570720000
C -3.4190960000 -1.2809690000 -1.1873580000
C -2.7357890000 -0.1501170000 -1.6395780000
C -3.5186930000 1.0755550000 -2.0354430000
C -1.3476620000 -0.1555170000 -1.7561250000
H -0.8184580000 0.7351420000 -2.0955120000
C 2.2156080000 1.7925230000 -2.2830660000

C 2.4629820000 1.5568700000 1.0684980000
C 0.0003060000 1.0635630000 1.3798090000
C -0.8336780000 0.0252740000 1.8338350000
H -0.4006790000 -0.9371760000 2.1037020000
C -2.2136700000 0.1982900000 1.8857590000
C -3.0827740000 -0.9146620000 2.4147390000
C -2.7998900000 1.4079560000 1.5044870000
C -1.9696550000 2.4359910000 1.0629880000
C -2.5576580000 3.7780560000 0.7000550000
C -0.5838920000 2.2763010000 0.9896580000
H 0.0392160000 3.0896490000 0.6314350000
N 3.0326570000 -1.3553520000 -0.9799520000
N 3.5056460000 1.4769500000 -2.0487100000
N 3.6361250000 0.8983600000 0.7417430000
N 0.7848400000 -1.2319330000 -1.5457420000
N 1.3719870000 0.7568180000 1.2672730000
S 1.3358570000 -2.5809630000 0.7135370000
O 1.4446710000 1.0962290000 -2.9418760000
S 2.4225550000 3.2337440000 1.2059650000
H 1.0739140000 -0.4664760000 -2.1692720000
H 1.5224300000 -0.2534520000 1.2406510000
H 1.8986940000 2.7427260000 -1.8070580000
H -4.5003970000 -1.2614410000 -1.0578840000
H -3.8807070000 1.5366570000 1.5295310000
F -4.5536780000 -3.4014070000 0.1976750000
F -2.6621730000 -4.4538920000 0.3402660000
F -3.7533440000 -4.4336220000 -1.5258900000
F -2.5276700000 -2.1239430000 2.2177550000
F -4.2927440000 -0.9217760000 1.8280410000
F -3.2904460000 -0.7893420000 3.7385680000
F -1.8816750000 4.3659210000 -0.3010890000
F -2.5156370000 4.6228190000 1.7490950000
F -3.8426350000 3.6796030000 0.3259440000
F -2.7591240000 2.1846580000 -2.0186870000
F -4.5626750000 1.2817900000 -1.2130690000
F -4.0187290000 0.9583600000 -3.2792840000

8 (*d1*)

Conf3

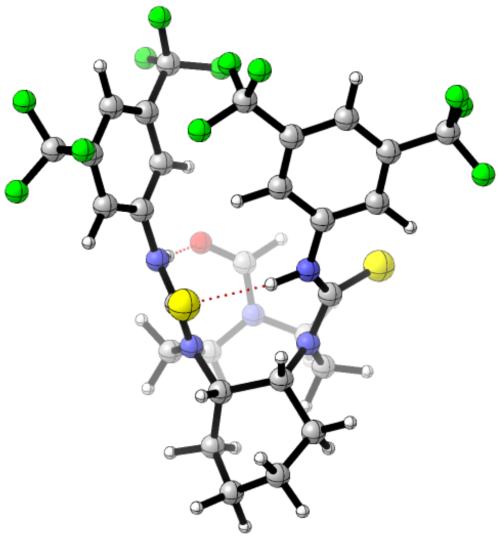

C -3.2684110000 2.8398140000 -1.0418560000

H -2.4173460000 3.3800050000 -1.4614250000
H -3.9660660000 3.6163520000 -0.6930900000
C -3.9940670000 2.0815840000 -2.1705320000
H -5.0404340000 1.9119580000 -1.8891560000
H -4.0037370000 2.7574770000 -3.0431010000
C -4.3201880000 -0.3780770000 -2.4828480000
H -3.8119420000 -1.2094520000 -2.9816680000
H -5.2633820000 -0.1838180000 -3.0220750000
C -4.6623430000 -0.7851550000 -1.0431170000
H -5.4361410000 -0.1116360000 -0.6600330000
H -5.0906840000 -1.8008280000 -1.0791470000
C -3.7768680000 -0.0173950000 1.1709480000
H -2.9412540000 -0.2794930000 1.8240370000
C -3.7528570000 1.5515690000 1.1021920000
H -3.3294590000 1.8508340000 2.0718300000
C -5.1576360000 2.1569240000 1.0561430000
H -5.7271580000 1.7906490000 0.1879300000
H -5.1009090000 3.2493110000 0.9522840000
C -5.9083170000 1.8159920000 2.3653310000
H -5.7544530000 2.6243500000 3.0972910000
H -6.9894870000 1.7911060000 2.1562700000
C -5.4611650000 0.4760130000 2.9838150000
H -4.6117370000 0.6380100000 3.6682900000
H -6.2711490000 0.0538130000 3.5976380000
C -5.0447870000 -0.5150110000 1.8974120000
H -5.8831390000 -0.6392690000 1.1950440000
H -4.8425250000 -1.5129690000 2.3152500000
C -1.4582600000 2.0395760000 0.4338440000
C 0.8192340000 2.3451880000 -0.5020770000
C 1.5322360000 2.9814040000 0.5221140000
H 1.0035420000 3.5030860000 1.3156440000
C 2.9256280000 2.9339700000 0.5207770000
C 3.6815550000 3.5408680000 1.6758090000
C 3.6250330000 2.2788860000 -0.4958680000
C 2.9082170000 1.6690890000 -1.5226460000
C 3.6199200000 0.9659480000 -2.6510570000
C 1.5118760000 1.6981970000 -1.5319310000
H 0.9512990000 1.2188950000 -2.3360560000
C -2.1981720000 0.6777500000 -3.0334970000
C -2.4823100000 -1.5869920000 -0.2854640000
C -0.1782250000 -2.0640770000 0.5673890000
C 0.9956070000 -1.3227930000 0.7749780000
H 0.9408740000 -0.2380450000 0.8706220000
C 2.2240980000 -1.9735960000 0.8907790000
C 3.4833790000 -1.1669950000 1.0821370000
C 2.3082120000 -3.3636670000 0.7939880000
C 1.1354380000 -4.0950110000 0.5979450000
C 1.2026900000 -5.5958470000 0.4617500000
C -0.1042760000 -3.4618840000 0.4956670000
H -1.0075560000 -4.0508530000 0.3657810000
N -2.7958540000 2.0729850000 0.1091800000
N -3.4574160000 0.7877260000 -2.5643880000
N -3.5483660000 -0.7292900000 -0.0996920000
N -0.5924500000 2.3536100000 -0.5689790000
N -1.3922390000 -1.3510340000 0.5082060000
S -0.9158560000 1.5985720000 1.9833750000
O -1.4074620000 1.6172960000 -3.1088920000
S -2.5186620000 -2.8169480000 -1.4344680000
H -0.9400110000 2.2508610000 -1.5325920000
H -1.3355240000 -0.4466330000 0.9874900000
H -1.9259300000 -0.3550780000 -3.3329170000
H 4.7125920000 2.2333870000 -0.4798960000
H 3.2676420000 -3.8704520000 0.8919850000
F 4.9146540000 3.9334350000 1.3125630000
F 3.8262850000 2.6639230000 2.6839760000
F 3.0447030000 4.6150890000 2.1767060000
F 4.0792560000 -0.8912210000 -0.0975190000
F 4.3871750000 -1.8259200000 1.8257740000

F 3.2406780000 0.0093290000 1.6832790000
F 0.1031870000 -6.1909110000 0.9564850000
F 2.2656150000 -6.1078260000 1.1064590000
F 1.3057500000 -5.9691710000 -0.8265490000
F 3.0787460000 -0.2399040000 -2.8947140000
F 4.9233890000 0.7867390000 -2.3945400000
F 3.5304400000 1.6707100000 -3.7964360000

8 (*d1*)

Conf9

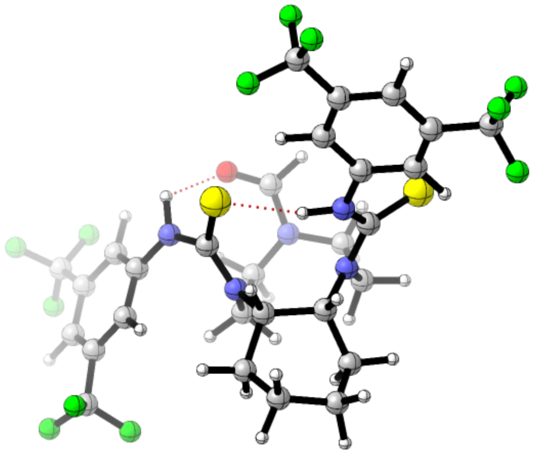

C 2.2098260000 -1.3367110000 0.7914980000
H 3.2346020000 -1.4264010000 0.3948760000
H 1.8667790000 -2.3639460000 0.9407160000
C 2.2980460000 -0.6210260000 2.1562120000
H 2.7788880000 -1.3305310000 2.8524310000
H 2.9470800000 0.2584670000 2.0937060000
C 0.1598780000 -1.1331980000 3.3617020000
H -0.6829360000 -0.5796040000 3.7942720000
H 0.6942440000 -1.6249530000 4.1960140000
C -0.3957460000 -2.2387150000 2.4603860000
H 0.4064740000 -2.9256070000 2.1804040000
H -1.1210210000 -2.8102050000 3.0629610000
C -0.6878930000 -2.3982890000 -0.0474430000
H -1.6048510000 -2.3316220000 -0.6463470000
C 0.4050500000 -1.6636110000 -0.9127520000
H -0.1416250000 -1.0156060000 -1.6058830000
C 1.2109970000 -2.6586510000 -1.7661840000
H 1.9826120000 -3.1668070000 -1.1651850000
H 1.7480390000 -2.0988120000 -2.5480230000
C 0.2744240000 -3.6946350000 -2.3904100000
H -0.4841680000 -3.1608600000 -2.9874680000
H 0.8287190000 -4.3270450000 -3.0996910000
C -0.4160530000 -4.5645460000 -1.3130340000
H -1.4587980000 -4.7611340000 -1.6089400000
H 0.0738340000 -5.5480560000 -1.2414630000
C -0.3944150000 -3.9028050000 0.0749110000
H 0.5863180000 -4.0794720000 0.5427210000
H -1.1426050000 -4.3703010000 0.7321390000
C 1.1915800000 0.5945800000 -0.4010410000
C 3.6374510000 1.0753710000 -0.2654800000

C 4.0724850000 0.1349020000 -1.2090430000
H 3.3490920000 -0.3742560000 -1.8444340000
C 5.4308800000 -0.1598720000 -1.3248410000
C 5.8290370000 -1.2790360000 -2.2530180000
C 6.3814910000 0.5069310000 -0.5522850000
C 5.9450160000 1.4771280000 0.3568200000
C 6.9619480000 2.2547210000 1.1585590000
C 4.5884990000 1.7526420000 0.5179040000
H 4.2526380000 2.4738620000 1.2646820000
C 0.7877580000 1.1653820000 2.8345520000
C -2.2394610000 -1.0911410000 1.3637330000
C -3.8794810000 0.1030840000 -0.0713340000
C -3.9330470000 1.3039890000 -0.7914790000
H -3.0088370000 1.8045530000 -1.0802160000
C -5.1653240000 1.8575470000 -1.1467280000
C -5.1931630000 3.1755830000 -1.8791070000
C -6.3587890000 1.2336100000 -0.7860330000
C -6.2993750000 0.0355760000 -0.0669930000
C -7.5807750000 -0.6330430000 0.3646050000
C -5.0782230000 -0.5349940000 0.2855700000
H -5.0541550000 -1.4752310000 0.8307560000
N 1.2918380000 -0.7498140000 -0.1888290000
N 1.0378440000 -0.1751060000 2.7143250000
N -1.0372860000 -1.7624040000 1.2380540000
N 2.2886470000 1.3635080000 -0.0887340000
N -2.6246850000 -0.4581650000 0.2040280000
S -0.1761790000 1.3840660000 -0.9783720000
O 1.5411410000 2.0599190000 2.4944500000
S -3.1186200000 -1.0399840000 2.7875680000
H 2.0559810000 2.2606750000 0.3278380000
H -1.8445310000 -0.0246120000 -0.3162590000
H -0.2079820000 1.3629820000 3.2900860000
H 7.4403700000 0.2722390000 -0.6497740000
H -7.3181770000 1.6625630000 -1.0726540000
F 5.2116310000 -1.1814500000 -3.4425010000
F 5.4721380000 -2.4737600000 -1.7299310000
F 7.1482950000 -1.3171660000 -2.4743580000
F -6.3266870000 3.3326330000 -2.5831770000
F -4.1629410000 3.2889950000 -2.7360330000
F -5.1098990000 4.2139160000 -1.0269590000
F -8.5688060000 -0.4326630000 -0.5256510000
F -8.0131540000 -0.1543930000 1.5449130000
F -7.4293280000 -1.9611380000 0.5090120000
F 7.3726860000 3.3472520000 0.4921870000
F 8.0527920000 1.5156790000 1.4200540000
F 6.4578810000 2.6692380000 2.3316240000

8 (*d1*)

Conf21

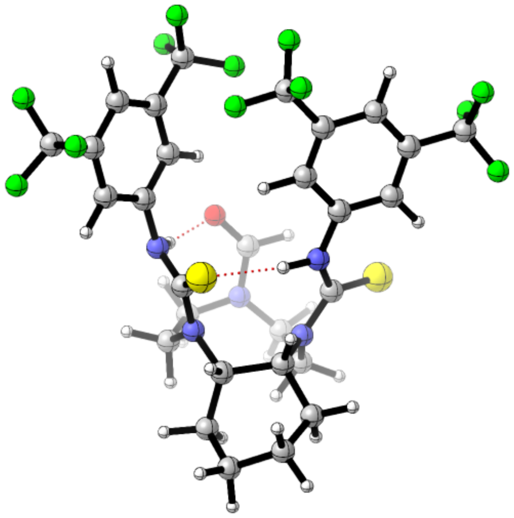

```
C 2.5989660000 3.3476120000 1.2991860000
H 1.9086450000 4.1958870000 1.4374160000
H 3.5809150000 3.7755670000 1.0872080000
C 2.6847100000 2.6122680000 2.6714690000
H 3.5120870000 3.0748920000 3.2332000000
H 1.7673230000 2.7997870000 3.2442070000
C 4.1967280000 0.5884650000 2.5157690000
H 4.1674400000 -0.4186110000 2.9467750000
H 4.9310520000 1.1778880000 3.0893240000
C 4.6812270000 0.4887800000 1.0568840000
H 5.0939470000 1.4561420000 0.7503180000
H 5.5015390000 -0.2485550000 1.0297330000
C 3.6228550000 0.9350190000 -1.1539890000
H 2.8744310000 0.4692030000 -1.8017470000
C 3.1667690000 2.4228360000 -0.9990230000
H 2.5802990000 2.6237650000 -1.9060790000
C 4.3370850000 3.4084340000 -1.0432070000
H 5.0750520000 3.2063550000 -0.2499990000
H 3.9733110000 4.4336520000 -0.8790550000
C 5.0249620000 3.3259210000 -2.4239110000
H 4.5248350000 4.0149480000 -3.1224130000
H 6.0603280000 3.6872150000 -2.3219330000
C 5.0146460000 1.8990170000 -3.0140310000
H 4.1371880000 1.7677550000 -3.6694280000
H 5.8977560000 1.7448780000 -3.6521660000
C 4.9639630000 0.8446330000 -1.9083420000
H 5.8111580000 0.9995680000 -1.2216710000
H 5.0744450000 -0.1727870000 -2.3132800000
C 0.8836080000 2.3343300000 -0.1640950000
C -1.3806520000 2.2480860000 0.7397140000
C -1.9647000000 1.1766790000 1.4175420000
H -1.3455650000 0.5344570000 2.0441480000
C -3.3316010000 0.9257470000 1.2662860000
C -3.9294780000 -0.2491140000 1.9980270000
C -4.1188650000 1.7323110000 0.4494710000
C -3.5254330000 2.8108610000 -0.2158500000
C -4.3577830000 3.6584310000 -1.1442460000
C -2.1651460000 3.0747490000 -0.0720580000
H -1.7081200000 3.9127470000 -0.5959770000
C 1.8009780000 0.3821100000 2.9055930000
```

C 2.9528240000 -1.0352650000 0.2162930000
C 0.7963830000 -2.0860590000 -0.5651630000
C -0.5281810000 -1.6732580000 -0.8060230000
H -0.7582570000 -0.6095620000 -0.8864060000
C -1.5401000000 -2.6165530000 -0.9565770000
C -2.9468480000 -2.1846920000 -1.2865530000
C -1.2653880000 -3.9838520000 -0.8560910000
C 0.0495490000 -4.3856460000 -0.6296250000
C 0.3740150000 -5.8515600000 -0.4807070000
C 1.0841250000 -3.4550010000 -0.4955420000
H 2.1030810000 -3.7921030000 -0.3322800000
N 2.1975380000 2.6168070000 0.0989460000
N 2.8788670000 1.1766610000 2.6780130000
N 3.6399650000 0.1572140000 0.0923680000
N 0.0135530000 2.4913640000 0.8837170000
N 1.7593120000 -1.0659330000 -0.4528000000
S 0.3484850000 1.8076460000 -1.6865390000
O 0.6549100000 0.8054220000 3.0142980000
S 3.5389740000 -2.2940800000 1.1633050000
H 0.3568980000 2.1887810000 1.8044290000
H 1.4123260000 -0.1692740000 -0.8067340000
H 2.0473100000 -0.6950160000 2.9784890000
H -5.1802020000 1.5215450000 0.3241610000
H -2.0603450000 -4.7214330000 -0.9637810000
F -3.1361040000 -1.3331670000 1.9072890000
F -5.1355470000 -0.5839910000 1.5178420000
F -4.0790440000 0.0133270000 3.3102670000
F -3.8518430000 -2.8047640000 -0.5120660000
F -3.2571960000 -2.4828570000 -2.5627400000
F -3.1217830000 -0.8599190000 -1.1374590000
F -0.5368260000 -6.6301180000 -1.0897050000
F 0.4061370000 -6.2188430000 0.8132850000
F 1.5765270000 -6.1497730000 -1.0028910000
F -4.4242440000 3.1222240000 -2.3755790000
F -3.8499920000 4.8966560000 -1.2798520000
F -5.6209110000 3.7842660000 -0.7015060000

8' (*d2*)

conf1

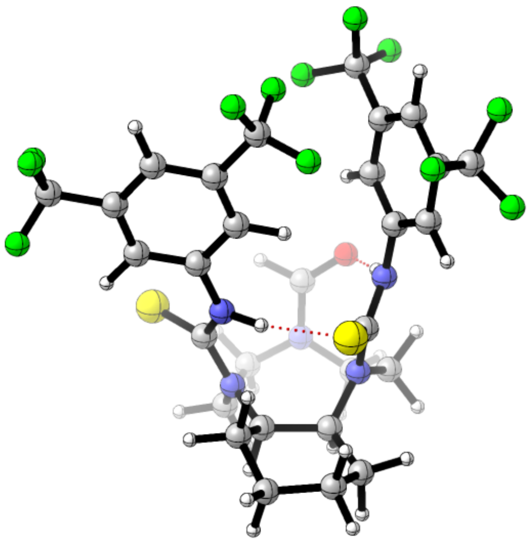

C 4.8954270000 -0.0091990000 1.2059890000
H 5.4500250000 0.9392450000 1.3026640000
H 5.5883350000 -0.7617160000 0.8067520000
C 4.4457550000 -0.4238940000 2.6162670000
H 5.3191410000 -0.8209410000 3.1614600000
H 4.0938660000 0.4654570000 3.1498780000
C 3.6425420000 -2.7732950000 2.2278540000
H 3.5331400000 -3.4465960000 3.0957630000
H 4.7011940000 -2.8044720000 1.9379630000
C 2.7702900000 -3.3650860000 1.0994280000
H 3.3130020000 -4.2451170000 0.7101960000
H 1.8387550000 -3.7476930000 1.5195710000
C 3.6456500000 -2.1560580000 -0.8564600000
C 4.0217120000 -0.6452900000 -1.0160520000
C 2.8814070000 1.1216830000 0.4277060000
C 0.6879850000 1.9484940000 -0.4375540000
C -0.6004450000 1.4280400000 -0.6288820000
H -0.7467180000 0.3498160000 -0.6835640000
C -1.6877320000 2.2889120000 -0.7863880000
C -3.0715200000 1.7145490000 -0.9556100000
C -1.5148510000 3.6728550000 -0.7476700000
C -0.2277110000 4.1839700000 -0.5660290000
C 0.8723670000 3.3383190000 -0.4221940000
H 1.8680650000 3.7559260000 -0.3026250000
C 2.1458070000 -1.0552120000 3.0919910000
C 1.1604970000 -2.2262540000 -0.3790620000
C -1.1495050000 -2.1859740000 0.5387890000
C -1.7468410000 -1.4661340000 1.5805540000
H -1.1263980000 -1.0861670000 2.3938220000
C -3.1238720000 -1.2352390000 1.5694360000
C -3.7386460000 -0.4692650000 2.7138440000
C -3.9158070000 -1.7113290000 0.5271280000
C -3.3129560000 -2.4383810000 -0.5023270000
C -4.1440810000 -2.8975680000 -1.6736040000
C -1.9415370000 -2.6887260000 -0.5014680000
H -1.4905910000 -3.2634050000 -1.3061540000
N 3.8193970000 0.1340200000 0.2248610000
N 3.3674550000 -1.4012600000 2.6341640000
N 2.4611100000 -2.4811820000 -0.0292750000
N 1.7546760000 1.0339360000 -0.3365860000
H 1.5734530000 0.1537960000 -0.8265080000
N 0.2440130000 -2.4016300000 0.6151340000
H 0.5967950000 -2.3516630000 1.5808820000
S 3.0983180000 2.3256890000 1.5875750000
O 1.1880800000 -1.8248560000 3.1464400000
S 0.7142350000 -1.6914870000 -1.9168980000
H -2.3641610000 4.3425800000 -0.8781230000
H 2.0740530000 0.0070310000 3.4028240000
C -0.0207790000 5.6763180000 -0.4914820000
C 3.8343850000 -2.9562550000 -2.1791160000
H 4.4455230000 -2.5288150000 -0.2070620000
C 3.6557250000 0.0358200000 -2.3422650000
H 5.1194820000 -0.7000660000 -1.0995440000
H 2.5799560000 0.1489580000 -2.4960960000
C 4.2287210000 -0.8016130000 -3.4913130000
H 4.1004430000 1.0438860000 -2.3351290000
C 3.6311020000 -2.2107660000 -3.5060610000
H 5.3297670000 -0.8564870000 -3.3971000000
H 4.0253660000 -0.2989550000 -4.4496920000
H 4.0943230000 -2.8055890000 -4.3103980000
H 2.5598090000 -2.1419330000 -3.7290150000
H 3.1900130000 -3.8474570000 -2.1401940000
H 4.8731270000 -3.3288680000 -2.1625500000
F -0.9457270000 6.3430130000 -1.2041760000
F 1.1875910000 6.0366230000 -0.9577530000
F -0.1031590000 6.1222560000 0.7750130000

F -3.0464060000 0.5189550000 -1.5677960000
F -3.8628550000 2.5251210000 -1.6767600000
F -3.6814200000 1.5340410000 0.2350480000
F -3.6471420000 -4.0177560000 -2.2286010000
F -4.1877040000 -1.9644790000 -2.6401380000
F -5.4137490000 -3.1525490000 -1.3129710000
F -4.9654340000 -0.0167790000 2.4172840000
F -2.9830800000 0.5862270000 3.0606400000
F -3.8537080000 -1.2435070000 3.8113110000
H -4.9852070000 -1.5073640000 0.5089310000

8' (*d2*)

Conf87

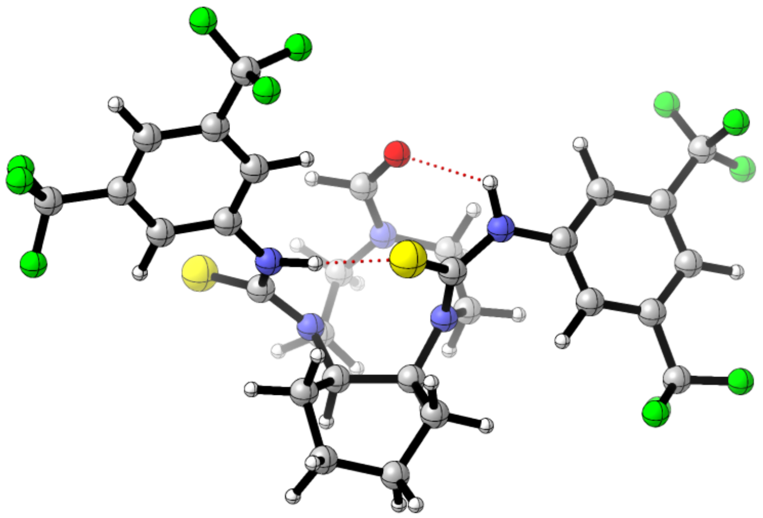

C 0.5379260000 -2.6990030000 2.4746070000
H 1.2416010000 -3.3695830000 2.9971170000
H -0.3565020000 -3.2857660000 2.2332100000
C 0.1802460000 -1.5414670000 3.4306770000
H -0.4854810000 -1.9224950000 4.2230110000
H 1.1044410000 -1.2006500000 3.9150080000
C -1.8415900000 -0.4158410000 2.4455530000
H -2.1221660000 0.6125220000 2.1943870000
H -2.4311280000 -0.7196130000 3.3281310000
C -2.2252820000 -1.3780840000 1.3122980000
H -2.1294430000 -2.4071670000 1.6703320000
H -3.2969060000 -1.2426250000 1.0939670000
C -0.8658960000 -2.5830550000 -0.3997220000
C 0.6195150000 -2.9457450000 0.0183640000
C 2.3342600000 -1.5834220000 1.2795530000
C 3.6506450000 0.0831550000 -0.0225090000
C 3.4230430000 1.3938720000 -0.4657990000
H 2.4017910000 1.7747730000 -0.5157610000
C 4.5003170000 2.2089680000 -0.8176070000
C 4.2343150000 3.5907290000 -1.3600450000
C 5.8109820000 1.7421170000 -0.7121280000
C 6.0292990000 0.4373490000 -0.2604040000
C 4.9640990000 -0.3971890000 0.0766980000
H 5.1488370000 -1.4128320000 0.4172270000
C 0.3211980000 0.6882860000 2.4279090000
C -1.1914090000 -0.1030200000 -0.5300210000
C -3.4849050000 0.8562250000 -0.2803510000

C -4.1746460000 -0.2063150000 -0.8842130000
H -3.6225940000 -1.0090490000 -1.3706110000
C -5.5685060000 -0.2336970000 -0.8666420000
C -6.2701780000 -1.4505340000 -1.4168470000
C -6.3036170000 0.8045580000 -0.2929730000
C -5.6124160000 1.8784350000 0.2764700000
C -6.3886250000 3.0441050000 0.8427420000
C -4.2190260000 1.9050370000 0.2990730000
H -3.6942420000 2.7358040000 0.7736640000
N 1.1060260000 -2.2333240000 1.2140820000
N -0.4360980000 -0.3935520000 2.7895720000
N -1.4112230000 -1.2963260000 0.0844950000
N 2.5275650000 -0.7060870000 0.2615950000
H 1.6855920000 -0.4547850000 -0.2793960000
N -2.0949230000 0.9090930000 -0.2559000000
H -1.6606490000 1.8258510000 -0.2065170000
S 3.4082740000 -1.7928250000 2.5491960000
O -0.0876130000 1.6524410000 1.8059510000
S 0.0847150000 0.2732420000 -1.5554950000
H 6.6503680000 2.3838940000 -0.9775370000
H 1.3740340000 0.5980050000 2.7740170000
C 7.4421130000 -0.0631600000 -0.0912070000
C -1.1095570000 -2.8373030000 -1.8946180000
H -1.4941010000 -3.3211430000 0.1152920000
C 1.5562290000 -2.9883650000 -1.1922140000
H 0.5507340000 -4.0009910000 0.3405350000
H 1.5637960000 -2.0196030000 -1.7041170000
C 1.0934430000 -4.0855640000 -2.1679280000
H 2.5844370000 -3.1908460000 -0.8596490000
C -0.4458210000 -4.1531650000 -2.3062730000
H 1.4749450000 -5.0655590000 -1.8380460000
H 1.5537820000 -3.8903710000 -3.1486070000
H -0.8469050000 -4.9674340000 -1.6773760000
H -0.7277760000 -4.4049800000 -3.3395000000
H -0.7098900000 -2.0116450000 -2.4967830000
H -2.1963210000 -2.8758300000 -2.0741610000
F 7.9227030000 0.2225320000 1.1323500000
F 8.2780360000 0.4986980000 -0.9823670000
F 7.5217980000 -1.3960980000 -0.2450280000
F 5.2641670000 4.4234610000 -1.1325590000
F 3.1373600000 4.1389740000 -0.8124450000
F 4.0368570000 3.5633510000 -2.6922820000
F -6.6662370000 3.9504610000 -0.1098990000
F -5.7009690000 3.6772910000 1.8069230000
F -7.5592450000 2.6477790000 1.3675550000
F -5.6212980000 -1.9604940000 -2.4782750000
F -7.5278440000 -1.1836020000 -1.7916470000
F -6.3265570000 -2.4277760000 -0.4887610000
H -7.3924860000 0.7786460000 -0.2883410000

8' (*d2*)

Conf13

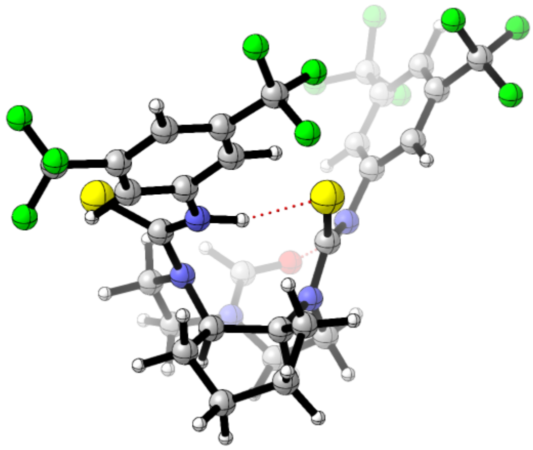

C -4.5627690000 -1.1254330000 -1.8853480000
H -4.0722470000 -1.5794880000 -2.7533530000
H -5.4822410000 -0.6387890000 -2.2529060000
C -4.9746050000 -2.2127270000 -0.8722640000
H -5.7836090000 -1.8454610000 -0.2274790000
H -5.3986130000 -3.0600770000 -1.4399400000
C -4.1206620000 -2.6804610000 1.4623480000
H -3.8354180000 -3.6681930000 1.8570580000
H -5.1998170000 -2.5692490000 1.6277890000
C -3.3348010000 -1.6166100000 2.2689380000
H -3.9278710000 -1.3753910000 3.1708860000
H -2.3974690000 -2.0508470000 2.6247300000
C -4.2197410000 0.3735840000 1.1180180000
C -4.2609150000 0.8117570000 -0.3962640000
C -2.3699630000 -0.0585460000 -1.9012850000
C -0.1264830000 0.9764290000 -1.4568700000
C 0.4002400000 2.2355690000 -1.1473500000
H -0.2704500000 3.0485270000 -0.8697870000
C 1.7799910000 2.4486610000 -1.1597000000
C 2.3006680000 3.8271170000 -0.8402050000
C 2.6537200000 1.4182250000 -1.5006760000
C 2.1217690000 0.1648130000 -1.8162830000
C 0.7484430000 -0.0698660000 -1.7821490000
H 0.3579770000 -1.0598790000 -1.9966890000
C -2.6860870000 -3.0173300000 -0.4412900000
C -1.7392340000 0.1342090000 1.5904070000
C 0.6132770000 -0.8204380000 1.4555010000
C 1.2202900000 -2.0462030000 1.1249100000
H 0.5970190000 -2.8990730000 0.8517310000
C 2.6067950000 -2.1602940000 1.0966600000
C 3.2399000000 -3.4936210000 0.7828480000
C 3.4193530000 -1.0621500000 1.3821250000
C 2.8106750000 0.1506880000 1.7044380000
C 3.6866810000 1.3405860000 2.0092590000
C 1.4229190000 0.2851690000 1.7533010000
H 0.9732140000 1.2465640000 1.9763910000
N -3.6448130000 -0.1213400000 -1.3620120000
N -3.9061080000 -2.6625960000 0.0202600000
N -3.0314270000 -0.3757460000 1.5525160000

```
N -1.5201200000 0.8163490000 -1.3021710000
H -1.8743300000 1.3721860000 -0.5279000000
N -0.7929690000 -0.8261280000 1.4199730000
H -1.1543000000 -1.7346830000 1.1030110000
S -1.8927230000 -0.9779630000 -3.2340360000
O -1.7397810000 -3.2669190000 0.3044580000
S -1.3926550000 1.7716780000 1.8014430000
H 3.7302340000 1.5803840000 -1.4949800000
H -2.5922060000 -3.0431580000 -1.5446890000
C 3.0485320000 -0.9441920000 -2.2467730000
C -4.6712630000 1.5362540000 2.0297690000
H -5.0164620000 -0.3754830000 1.1932830000
C -4.0078550000 2.3192010000 -0.5778940000
H -5.3274220000 0.7157640000 -0.6433710000
H -3.0738430000 2.6328950000 -0.0970960000
C -5.1700470000 3.0968040000 0.0797840000
H -3.9346110000 2.5583330000 -1.6490560000
C -5.7364190000 2.3835240000 1.3300260000
H -5.9802430000 3.2502310000 -0.6517010000
H -4.8072030000 4.0991490000 0.3540990000
H -6.5894010000 1.7407810000 1.0461010000
H -6.1459900000 3.1261880000 2.0312220000
H -3.8046270000 2.1570740000 2.2830790000
H -5.0556370000 1.1186310000 2.9741300000
F 4.2379040000 -0.8719080000 -1.6223720000
F 3.2971770000 -0.8888470000 -3.5673970000
F 2.5300010000 -2.1600560000 -1.9954890000
F 2.1184830000 4.6687180000 -1.8781130000
F 3.6114400000 3.8200010000 -0.5607630000
F 1.6567270000 4.3655980000 0.2101520000
F 4.2768270000 1.2233470000 3.2126840000
F 2.9961370000 2.4915320000 2.0132870000
F 4.6750680000 1.4635650000 1.1021950000
F 4.3899410000 -3.3505040000 0.1061300000
F 2.4290820000 -4.2783850000 0.0579330000
F 3.5322700000 -4.1662860000 1.9148500000
H 4.5039510000 -1.1441300000 1.3195680000
```

8' (*d2*)

Conf15

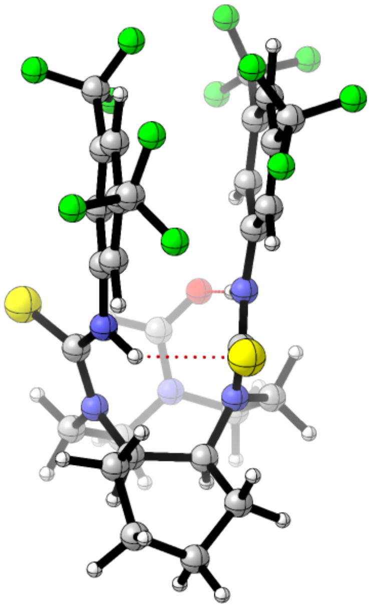

C -4.5627690000 -1.1254330000 -1.8853480000
H -4.0722470000 -1.5794880000 -2.7533530000
H -5.4822410000 -0.6387890000 -2.2529060000
C -4.9746050000 -2.2127270000 -0.8722640000
H -5.7836090000 -1.8454610000 -0.2274790000
H -5.3986130000 -3.0600770000 -1.4399400000
C -4.1206620000 -2.6804610000 1.4623480000
H -3.8354180000 -3.6681930000 1.8570580000
H -5.1998170000 -2.5692490000 1.6277890000
C -3.3348010000 -1.6166100000 2.2689380000
H -3.9278710000 -1.3753910000 3.1708860000
H -2.3974690000 -2.0508470000 2.6247300000
C -4.2197410000 0.3735840000 1.1180180000
C -4.2609150000 0.8117570000 -0.3962640000
C -2.3699630000 -0.0585460000 -1.9012850000
C -0.1264830000 0.9764290000 -1.4568700000
C 0.4002400000 2.2355690000 -1.1473500000
H -0.2704500000 3.0485270000 -0.8697870000
C 1.7799910000 2.4486610000 -1.1597000000
C 2.3006680000 3.8271170000 -0.8402050000
C 2.6537200000 1.4182250000 -1.5006760000
C 2.1217690000 0.1648130000 -1.8162830000
C 0.7484430000 -0.0698660000 -1.7821490000
H 0.3579770000 -1.0598790000 -1.9966890000
C -2.6860870000 -3.0173300000 -0.4412900000
C -1.7392340000 0.1342090000 1.5904070000
C 0.6132770000 -0.8204380000 1.4555010000
C 1.2202900000 -2.0462030000 1.1249100000
H 0.5970190000 -2.8990730000 0.8517310000
C 2.6067950000 -2.1602940000 1.0966600000
C 3.2399000000 -3.4936210000 0.7828480000
C 3.4193530000 -1.0621500000 1.3821250000
C 2.8106750000 0.1506880000 1.7044380000
C 3.6866810000 1.3405860000 2.0092590000
C 1.4229190000 0.2851690000 1.7533010000
H 0.9732140000 1.2465640000 1.9763910000
N -3.6448130000 -0.1213400000 -1.3620120000
N -3.9061080000 -2.6625960000 0.0202600000
N -3.0314270000 -0.3757460000 1.5525160000
N -1.5201200000 0.8163490000 -1.3021710000
H -1.8743300000 1.3721860000 -0.5279000000
N -0.7929690000 -0.8261280000 1.4199730000
H -1.1543000000 -1.7346830000 1.1030110000
S -1.8927230000 -0.9779630000 -3.2340360000
O -1.7397810000 -3.2669190000 0.3044580000
S -1.3926550000 1.7716780000 1.8014430000
H 3.7302340000 1.5803840000 -1.4949800000
H -2.5922060000 -3.0431580000 -1.5446890000
C 3.0485320000 -0.9441920000 -2.2467730000
C -4.6712630000 1.5362540000 2.0297690000
H -5.0164620000 -0.3754830000 1.1932830000
C -4.0078550000 2.3192010000 -0.5778940000
H -5.3274220000 0.7157640000 -0.6433710000
H -3.0738430000 2.6328950000 -0.0970960000
C -5.1700470000 3.0968040000 0.0797840000
H -3.9346110000 2.5583330000 -1.6490560000
C -5.7364190000 2.3835240000 1.3300260000
H -5.9802430000 3.2502310000 -0.6517010000
H -4.8072030000 4.0991490000 0.3540990000
H -6.5894010000 1.7407810000 1.0461010000
H -6.1459900000 3.1261880000 2.0312220000
H -3.8046270000 2.1570740000 2.2830790000
H -5.0556370000 1.1186310000 2.9741300000
F 4.2379040000 -0.8719080000 -1.6223720000
F 3.2971770000 -0.8888470000 -3.5673970000
F 2.5300010000 -2.1600560000 -1.9954890000
F 2.1184830000 4.6687180000 -1.8781130000
F 3.6114400000 3.8200010000 -0.5607630000

F 1.6567270000 4.3655980000 0.2101520000
F 4.2768270000 1.2233470000 3.2126840000
F 2.9961370000 2.4915320000 2.0132870000
F 4.6750680000 1.4635650000 1.1021950000
F 4.3899410000 -3.3505040000 0.1061300000
F 2.4290820000 -4.2783850000 0.0579330000
F 3.5322700000 -4.1662860000 1.9148500000
H 4.5039510000 -1.1441300000 1.3195680000

Crystallography

X-ray diffraction experiments were carried out at 100(2) K. **1a** was run on a Bruker APEX II diffractometer using Mo-K_α radiation (λ = 0.71073 Å) and a CCD area detector. While **2a** and **2b** were run on a Bruker D8 Venture diffractometer using Mo-K_α radiation (λ = 0.71073 Å) and a Bruker CPAD detector and **3**, **4** and **9** were run on a Bruker D8 Venture diffractometer using Cu-K_α radiation (λ = 1.54178 Å) and a Bruker CPAD detector. Intensities were integrated in SAINT[S23] and absorption corrections based on equivalent reflections were applied using SADABS.[S24] Structure **1a** was solved using Superflip[S25,S26] while structures **2a**, **2b**, **3**, **4** and **9** were solved using ShelXT[S27] and refined by full matrix least squares against *F*[S24] in ShelXL[S28,S29] using Olex2.[S30] All of the non-hydrogen atoms were refined anisotropically. While all of the hydrogen atoms in **2a**, **2b**, **3**, **4** and **9** were located geometrically and refined using a riding model, apart from the N-H protons in all structures and the full occupancy water molecule protons in **9** which were located in the difference map.

In the case of **1a**, distances between some intramolecular hydrogen atoms appear short, giving rise to one A level and five B level checkCIF alerts. To check this the hydrogen atoms, on all non-disordered atoms, were found by difference map, after all other atoms had been refined. The short hydrogen-hydrogen distances appear to be due to the intramolecular hydrogen bonding, exhibited by the complex, which forces the molecule to fold in on itself. All hydrogen atoms, on non-disordered atoms, have been found by difference map and refined. Hydrogens on disordered atoms were calculated and refined using a riding model.

1a, **2b** and **4** contain some disorder in the main molecule and **3** and **9** contain disordered solvent molecules, that have been modelled in two or three positions and the occupancies of the fragments were determined by refining them against a free variable with the sum of the sites set to equal 1. SADI, SIMU and ISOR were used, as necessary, to maintain sensible geometries and thermal parameters. For **9**, due to the low occupancy of the minor component, it has been modelled as an idealised fixed geometry fragment. In the case of **1a, 2a** and **2b**, the solvent molecule disorder was resolved using a solvent mask, leading to the apparent presence of solvent accessible voids in these structures.

The crystal structure and refinement data are given in Table S32. Crystallographic data for compounds **1a**, **2a**, **2b**, **3**, **4** and **9** has been deposited with the Cambridge Crystallographic Data Centre as supplementary publications CCDC 2262692-2262697. Copies of the data can be obtained free of charge on application to CCDC, 12 Union Road, Cambridge CB2 1EZ, UK [fax(+44) 1223 336033, e-mail: deposit@ccdc.cam.ac.uk].

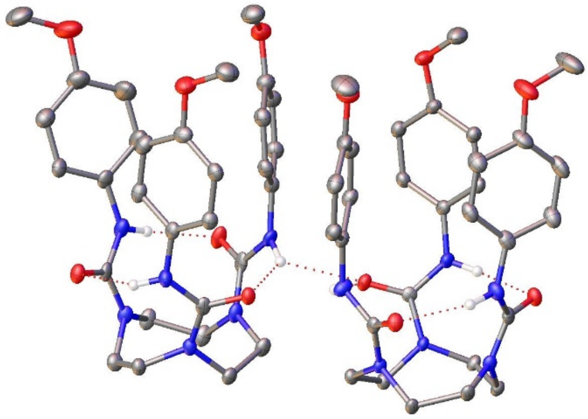

Figure S65 − Crystal structure of **1a**, showing two molecules present in the asymmetric unit, with the anisotropic displacement parameters depicted at the 50% probability level. Disorder and hydrogens, except the N-H protons, omitted for clarity.

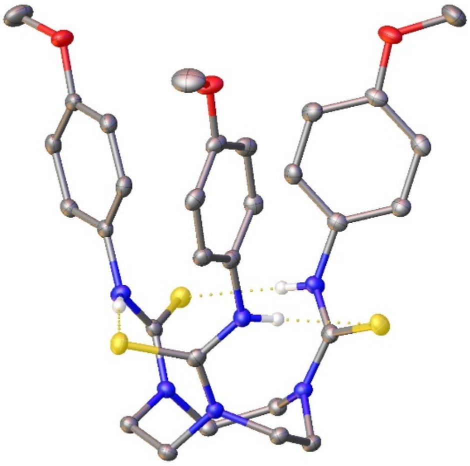

Figure S66 − Crystal structure of **2a**, with the anisotropic displacement parameters depicted at the 50% probability level. Hydrogens, except the N-H protons, omitted for clarity. Note only one third of the molecule is in the asymmetric unit, the rest is symmetry generated.

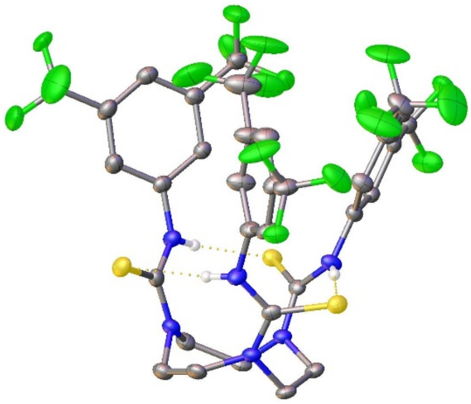

Figure S67 − Crystal structure of **2b**, with the anisotropic displacement parameters depicted at the 50% probability level. Disorder and hydrogens, except the N-H protons, omitted for clarity.

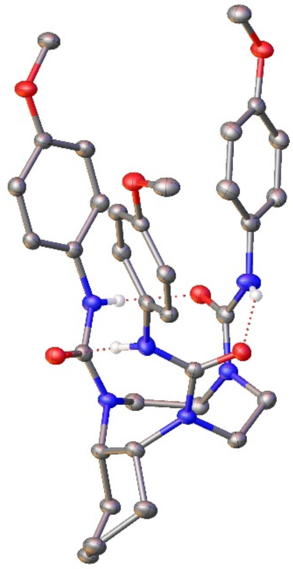

Figure S68 − Crystal structure of **3**, with the anisotropic displacement parameters depicted at the 50% probability level. Solvent molecules and hydrogens, except the N-H protons, omitted for clarity.

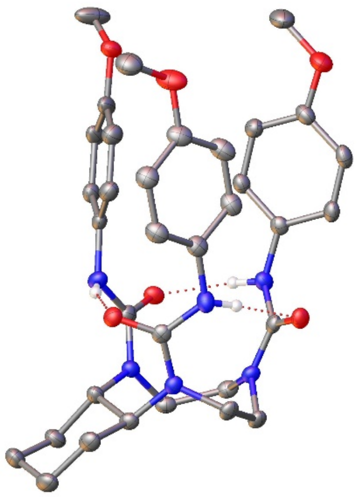

Figure S69 − Crystal structure of **4**, with the anisotropic displacement parameters depicted at the 50% probability level. Disorder and hydrogens, except the N-H protons, omitted for clarity.

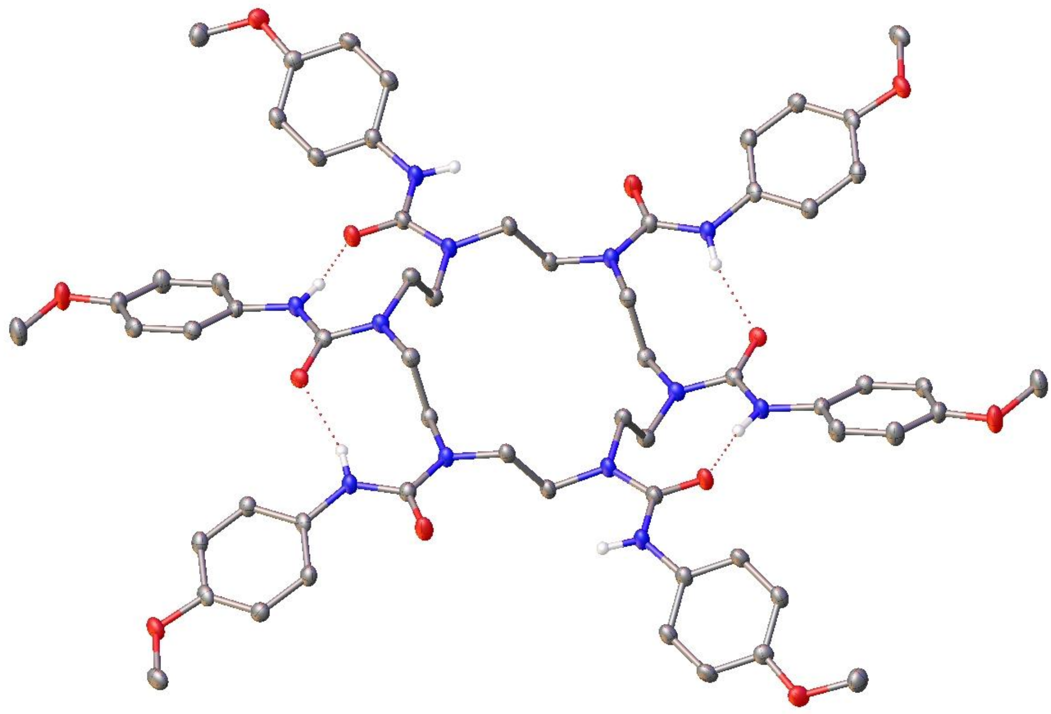

Figure S70 − Crystal structure of **9**, with the anisotropic displacement parameters depicted at the 50% probability level. Solvent molecules and hydrogens, except the N-H protons, omitted for clarity.

Table S32 − Crystal data and structure refinement.

Complex	**1a**	**2a**	**2b**	**3**	**4**	**9**
CCDC number	2262692	2262693	2262694	2262695	2262696	2262697
Empirical formula	$C_{30}H_{36}N_6O_6$	$C_{30}H_{36}N_6O_3S_3$	$C_{33}H_{24}F_{18}N_6S_3$	$C_{35}H_{44}ClN_6O_6$	$C_{34}H_{42}N_6O_6$	$C_{63}H_{83.19}N_{13}O_{15.1}$
Formula weight	576.65	624.83	942.76	680.21	630.73	1264.22
Temperature/K	100.00	102.00	100.00	100.00	100(2)	100(2)
Crystal system	monoclinic	trigonal	monoclinic	monoclinic	orthorhombic	triclinic
Space group	$P2_1/c$	P-3c1	$P2_1/c$	$P2_1/n$	Aea2	P-1
a/Å	12.6236(4)	13.5368(8)	12.1715(7)	8.4273(3)	30.2008(7)	11.3729(3)
b/Å	14.4638(4)	13.5368(8)	17.2634(9)	31.6869(13)	14.9213(4)	15.4366(4)
c/Å	34.9564(9)	20.8264(16)	20.6545(11)	12.1622(5)	14.2365(4)	19.9382(6)
α/°	90	90	90	90	90	72.8470(10)
β/°	92.5613(17)	90	95.540(2)	93.591(2)	90	84.3640(10)
γ/°	90	120	90	90	90	73.0650(10)
Volume/$Å^3$	6376.1(3)	3305.0(5)	4319.7(4)	3241.4(2)	6415.5(3)	3199.31(15)
Z	8	4	4	4	8	2
$\rho_{calc}g/cm^3$	1.201	1.256	1.450	1.394	1.306	1.312
μ/mm^{-1}	0.085	0.264	0.281	1.515	0.741	0.785
F(000)	2448.0	1320.0	1896.0	1444.0	2688.0	1346.0
Crystal size/mm^3	0.47 × 0.309 × 0.192	0.401 × 0.212 × 0.12	0.299 × 0.22 × 0.16	0.423 × 0.065 × 0.02	0.534 × 0.347 × 0.02	0.328 × 0.262 × 0.12
Radiation	MoKα (λ = 0.71073)	MoKα (λ = 0.71073)	MoKα (λ = 0.71073)	CuKα (λ = 1.54178)	CuKα (λ = 1.54178)	CuKα (λ = 1.54178)
2Θ range for data collection/°	3.048 to 52.744	3.912 to 54.34	3.962 to 54.256	5.578 to 137.29	9.07 to 137.062	4.638 to 137.232
Index ranges	-15 ≤ h ≤ 15, -14 ≤ k ≤ 18,-43 ≤ l ≤ 43	-17 ≤ h ≤ 17, -17 ≤ k ≤ 17, -26 ≤ l ≤ 26	-15 ≤ h ≤ 15, -22 ≤ k ≤ 22, -26 ≤ l ≤ 26	-10 ≤ h ≤ 10, -38 ≤ k ≤ 38, -14 ≤ l ≤ 14	-36 ≤ h ≤ 36, -17 ≤ k ≤ 17, -16 ≤ l ≤ 17	-13 ≤ h ≤ 13, -18 ≤ k ≤ 18, -24 ≤ l ≤ 24
Reflections collected	102110	76758	117704	49761	27928	54962
Independent reflections	13032 [R_{int} = 0.0706, R_{sigma} = 0.0399]	2461 [R_{int} = 0.0632, R_{sigma} = 0.0205]	9546 [R_{int} = 0.0595, R_{sigma} = 0.0340]	5989 [R_{int} = 0.0400, R_{sigma} = 0.0218]	5544 [R_{int} = 0.0259, R_{sigma} = 0.0204]	11693 [R_{int} = 0.0421, R_{sigma} = 0.0338]
Data/restraints/parameters	13032/697/1042	2461/0/132	9546/997/739	5989/37/467	5544/27/454	11693/138/919
Goodness-of-fit on F^2	1.045	1.161	1.055	1.075	1.063	1.034
Final R indexes [I>=2σ (I)]	R_1=0.0623, wR_2 = 0.1274	R_1 = 0.0455, wR_2 = 0.0969	R_1 = 0.0402, wR_2 = 0.0945	R_1 = 0.0359, wR_2 = 0.0879	R_1 = 0.0293, wR_2 = 0.0769	R_1 = 0.0406, wR_2 = 0.1049
Final R indexes [all data]	R_1 = 0.0887, wR_2 = 0.1393	R_1 = 0.0508, wR_2 = 0.0988	R_1 = 0.0591, wR_2 = 0.1017	R_1 = 0.0376, wR_2 = 0.0889	R_1 = 0.0298, wR_2 = 0.0773	R_1 = 0.0444, wR_2 = 0.1073
Largest diff. peak/hole / e $Å^{-3}$	0.41/-0.47	0.28/-0.23	0.33/-0.30	0.26/-0.20	0.21/-0.18	0.32/-0.30

NMR Spectra of Novel Compounds

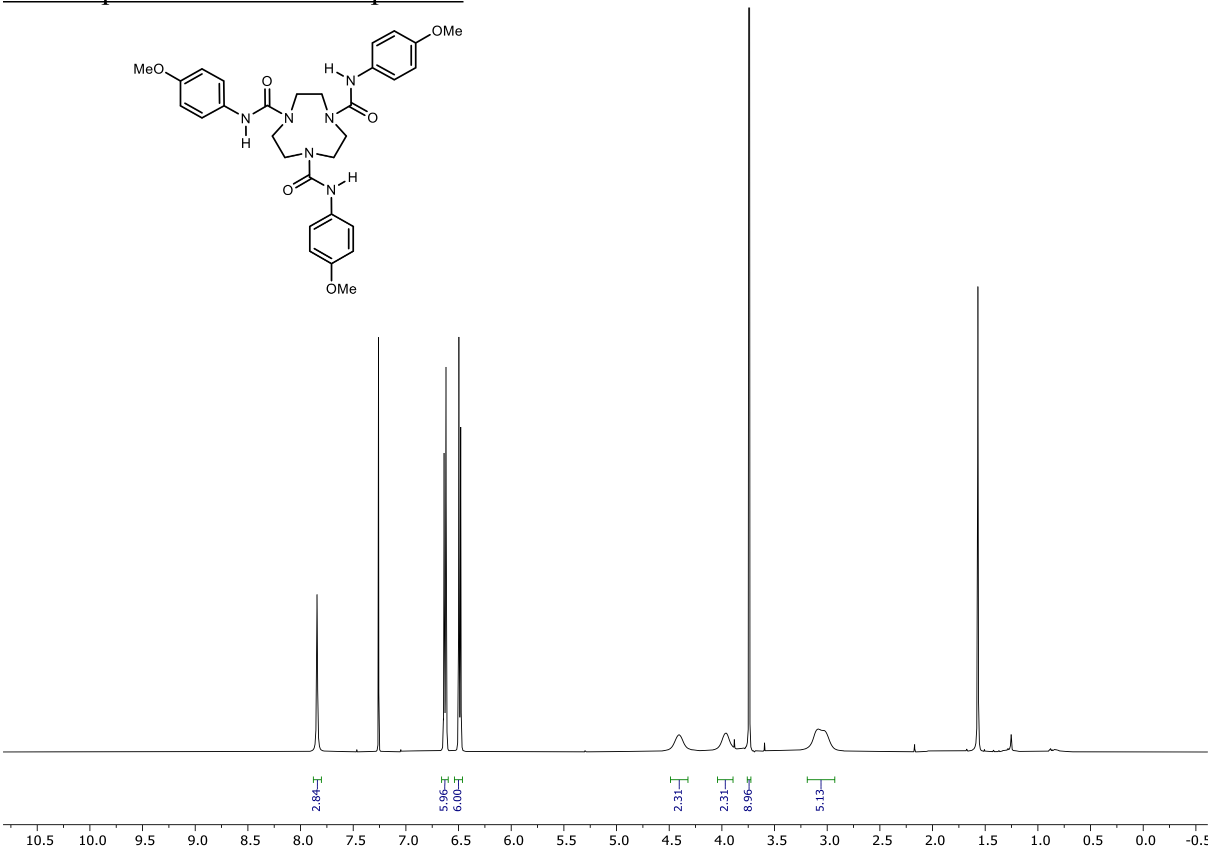

Figure S71 – ^{1}H NMR spectrum of **1a** ($CDCl_3$, 500 MHz).

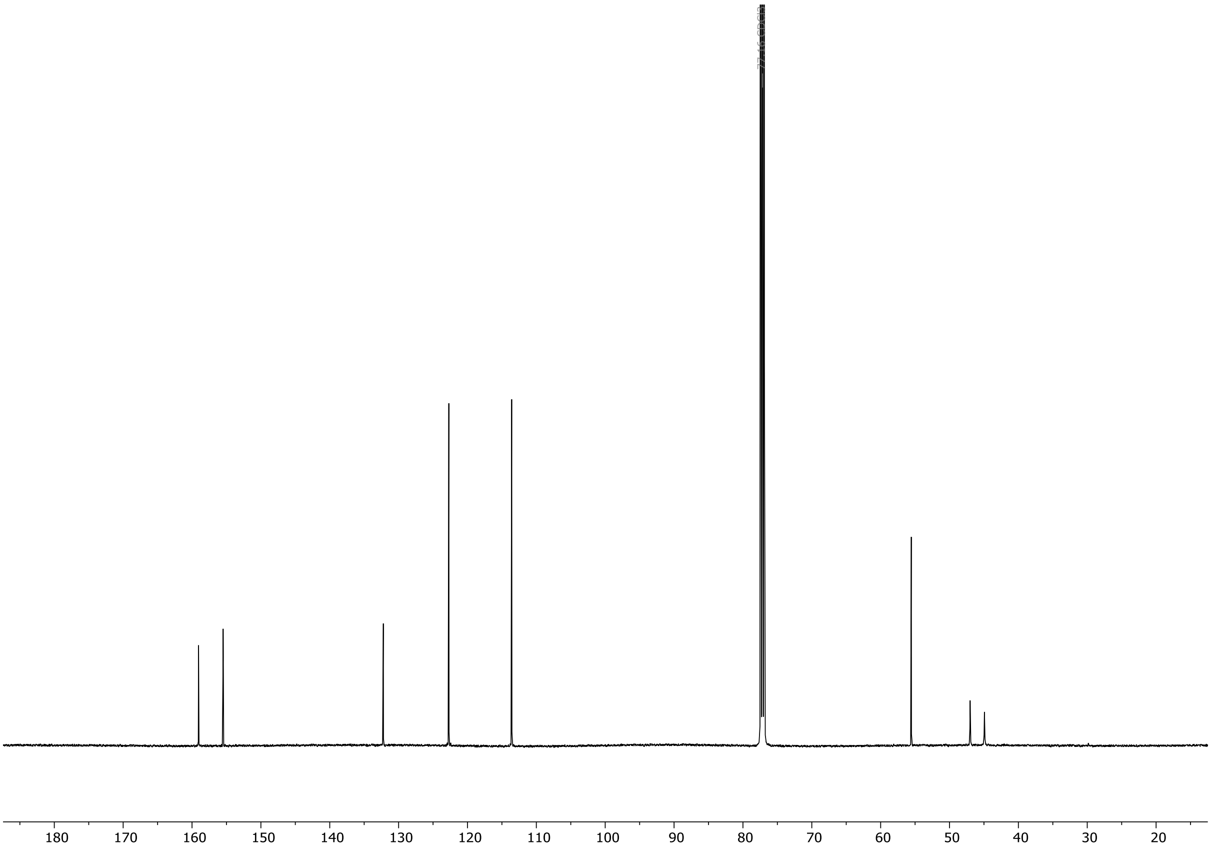

Figure S72 – ^{13}C NMR spectrum of **1a** ($CDCl_3$, 126 MHz).

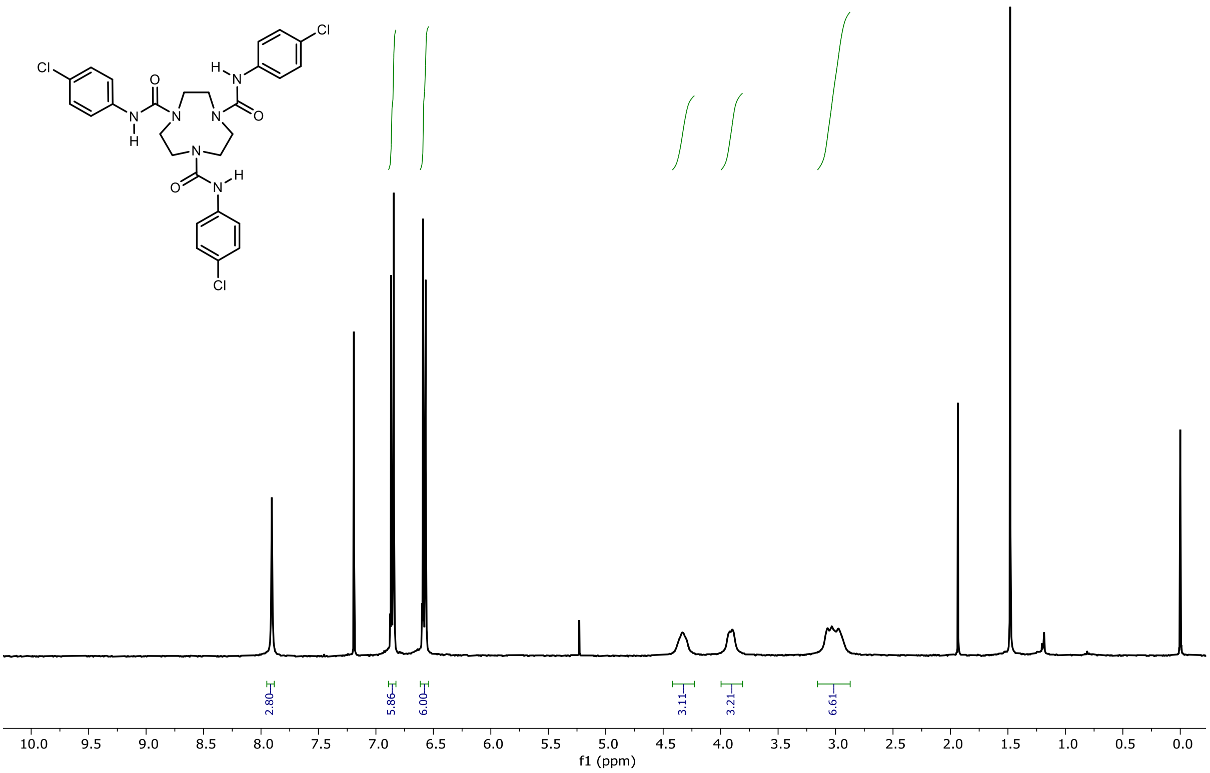

Figure S73 – ^{1}H NMR spectrum of **1b** ($CDCl_3$, 400 MHz).

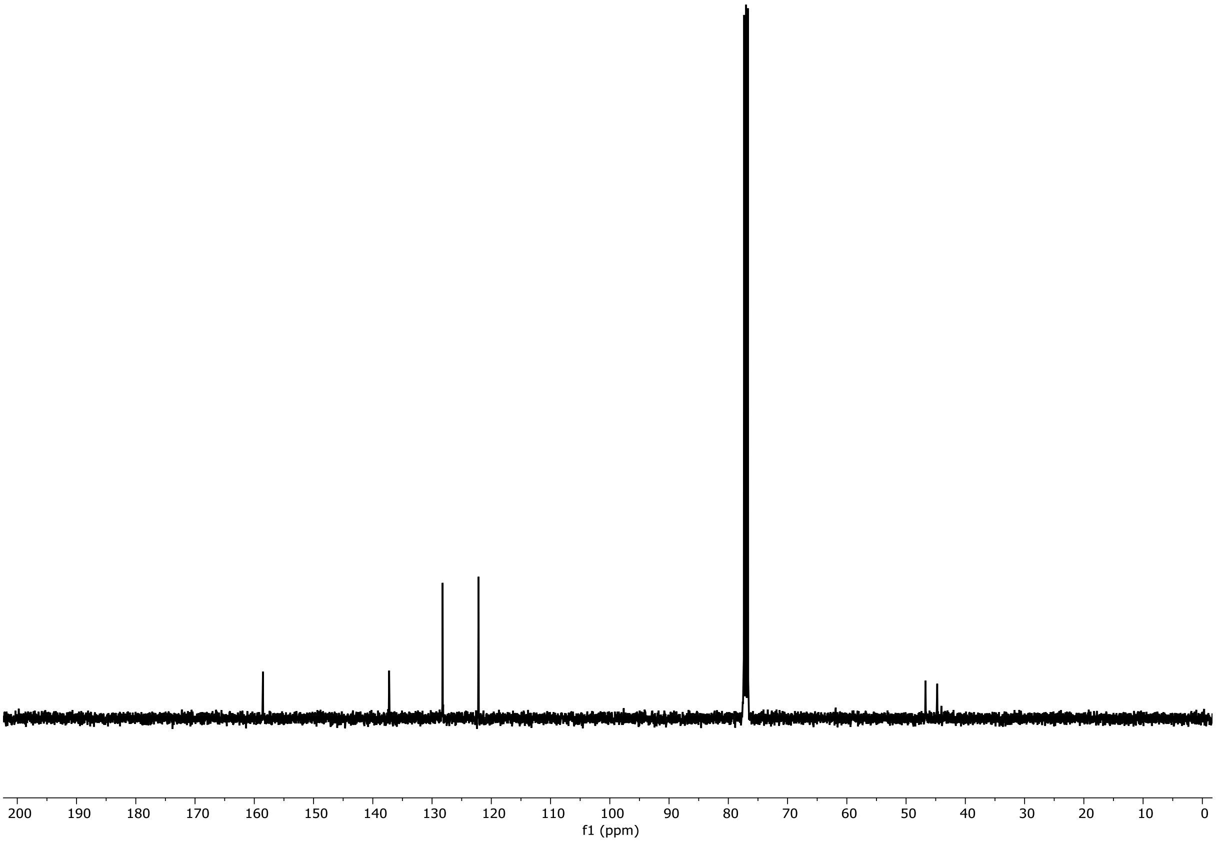

Figure S74 – ^{13}C NMR spectrum of **1b** ($CDCl_3$, 101 MHz).

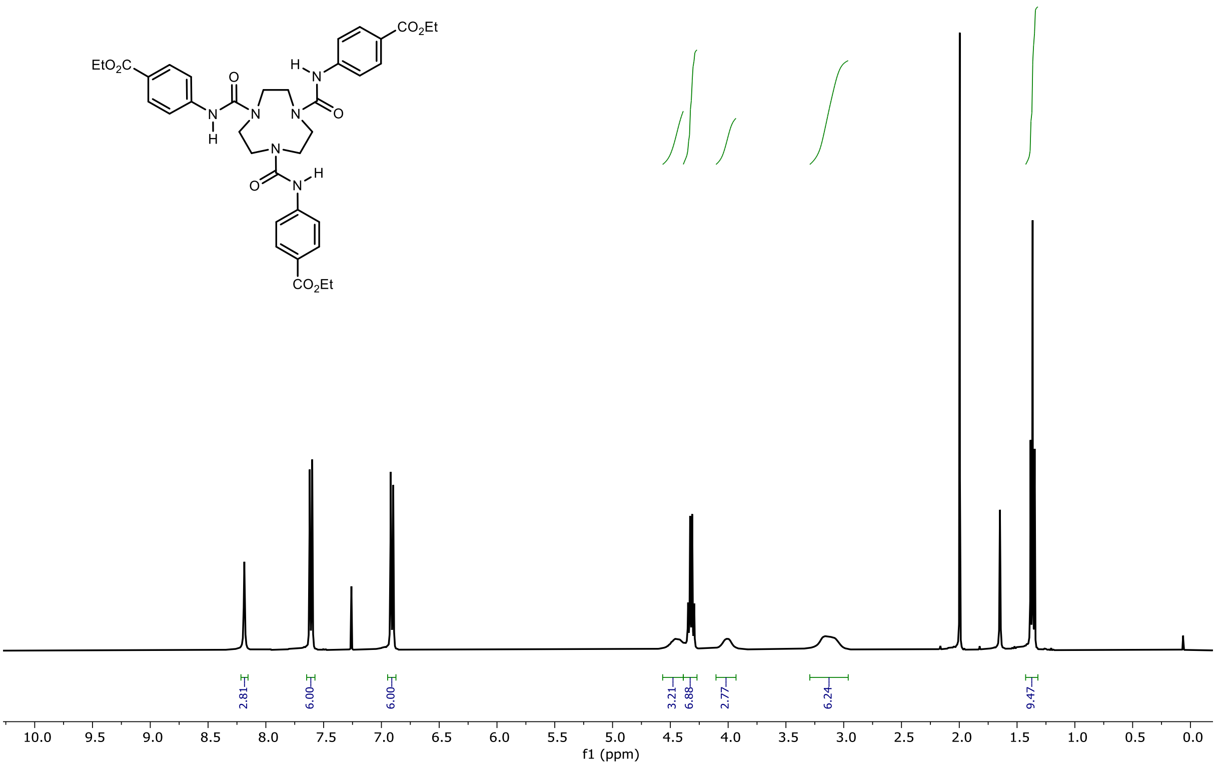

Figure S75 – ^{1}H NMR spectrum of **1c** ($CDCl_3$, 400 MHz).

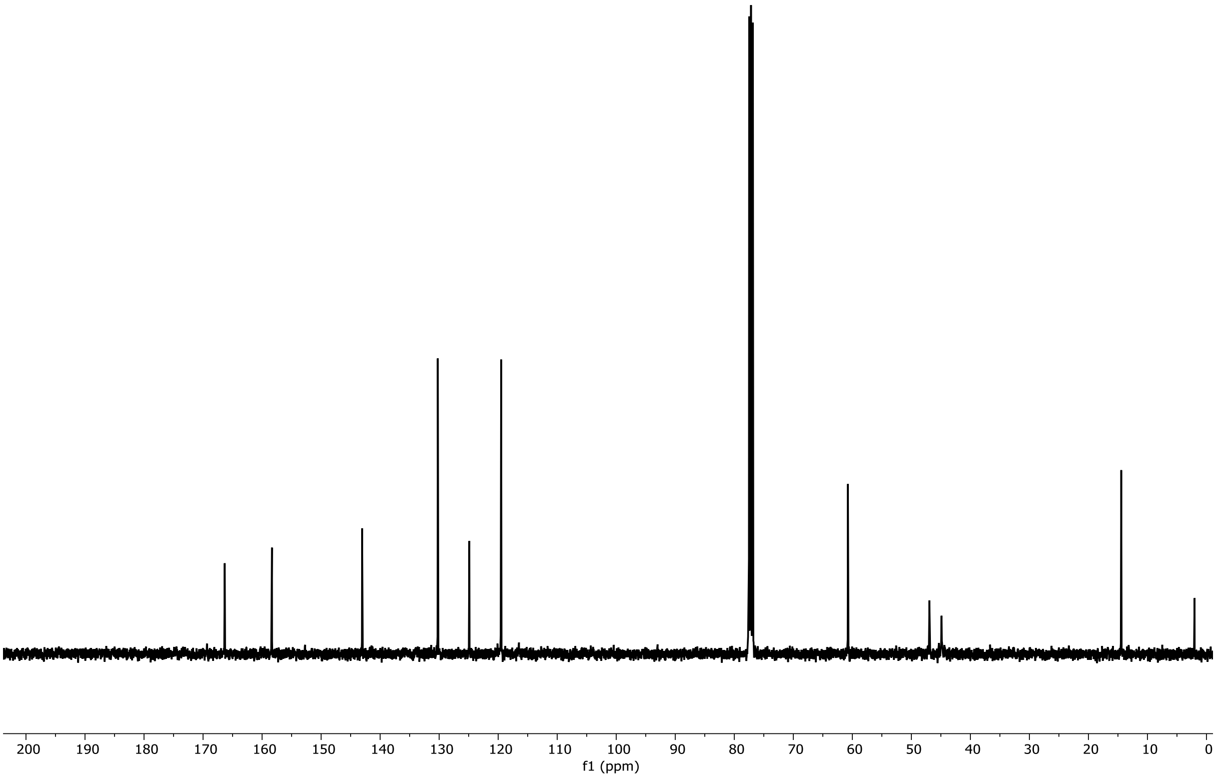

Figure S76 – ^{13}C NMR spectrum of **1c** ($CDCl_3$, 101 MHz).

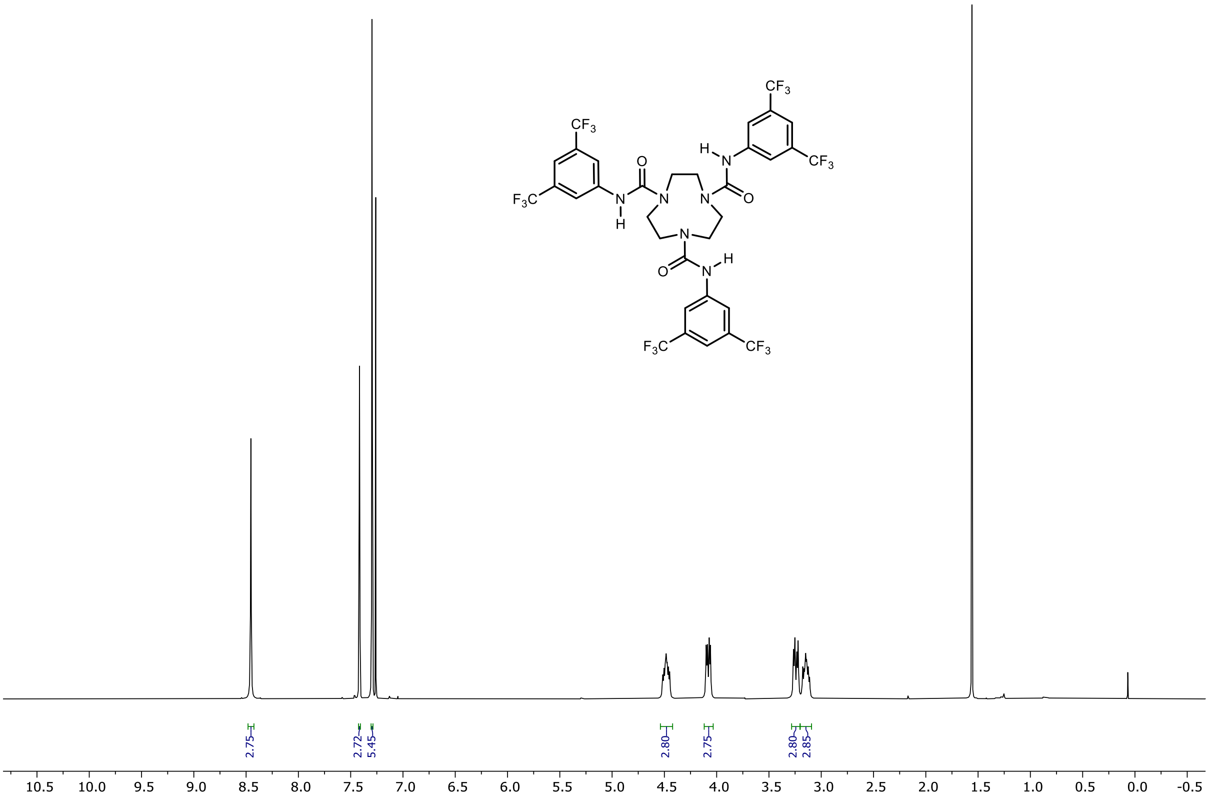

Figure S77 – ^{1}H NMR spectrum of **1d** ($CDCl_3$, 500 MHz).

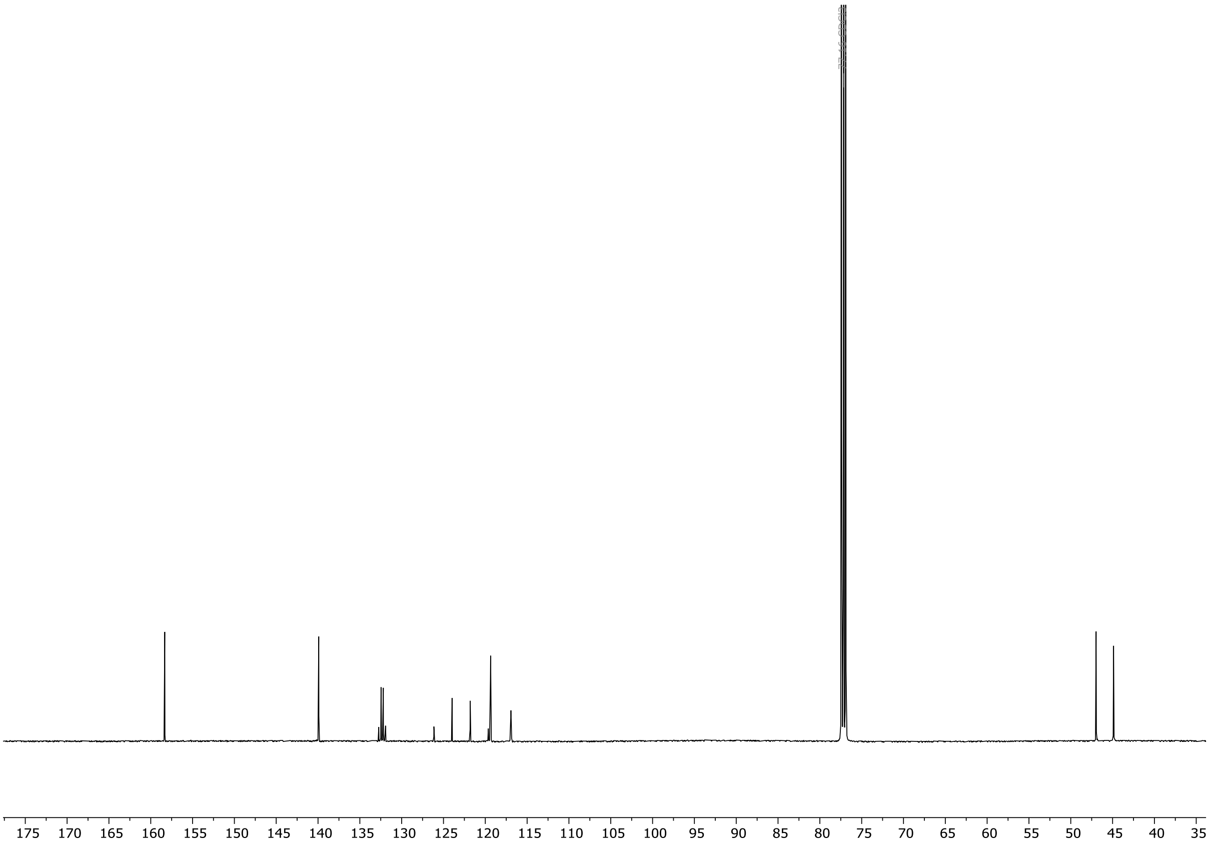

Figure S78 – ^{13}C NMR spectrum of **1d** ($CDCl_3$, 126 MHz).

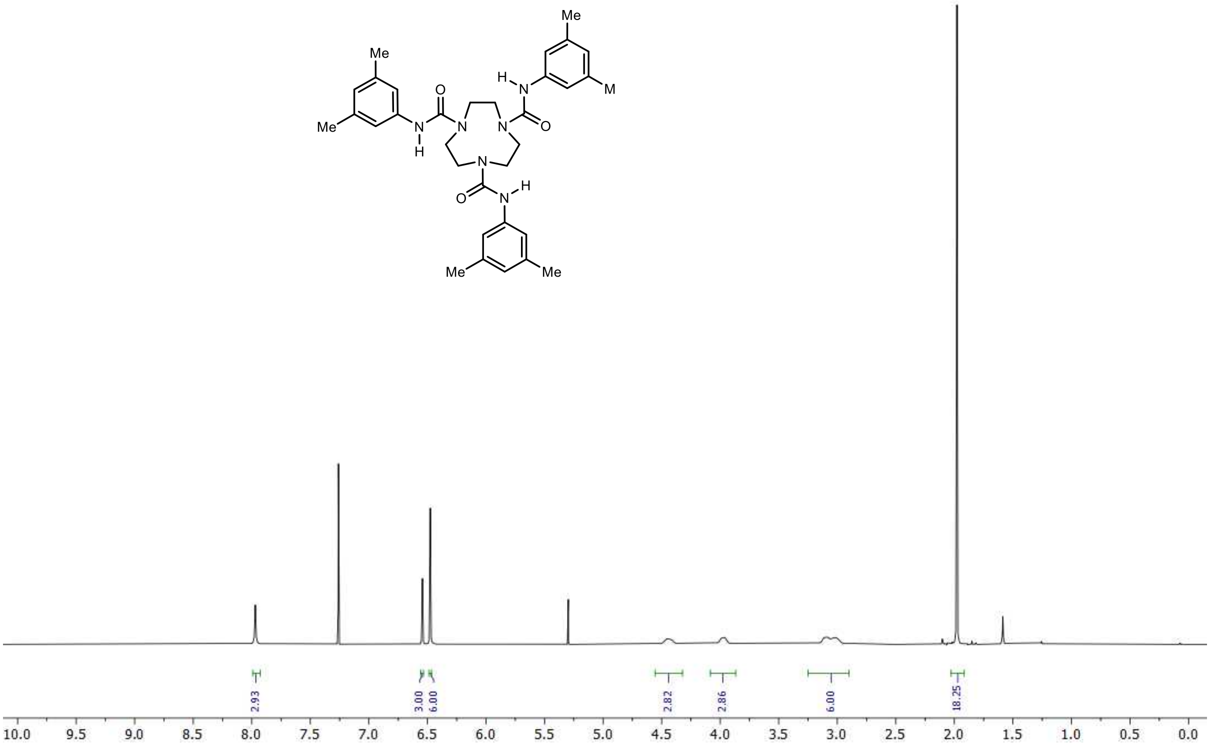

Figure S79 – ^{1}H NMR spectrum of **1e** ($CDCl_3$, 500 MHz).

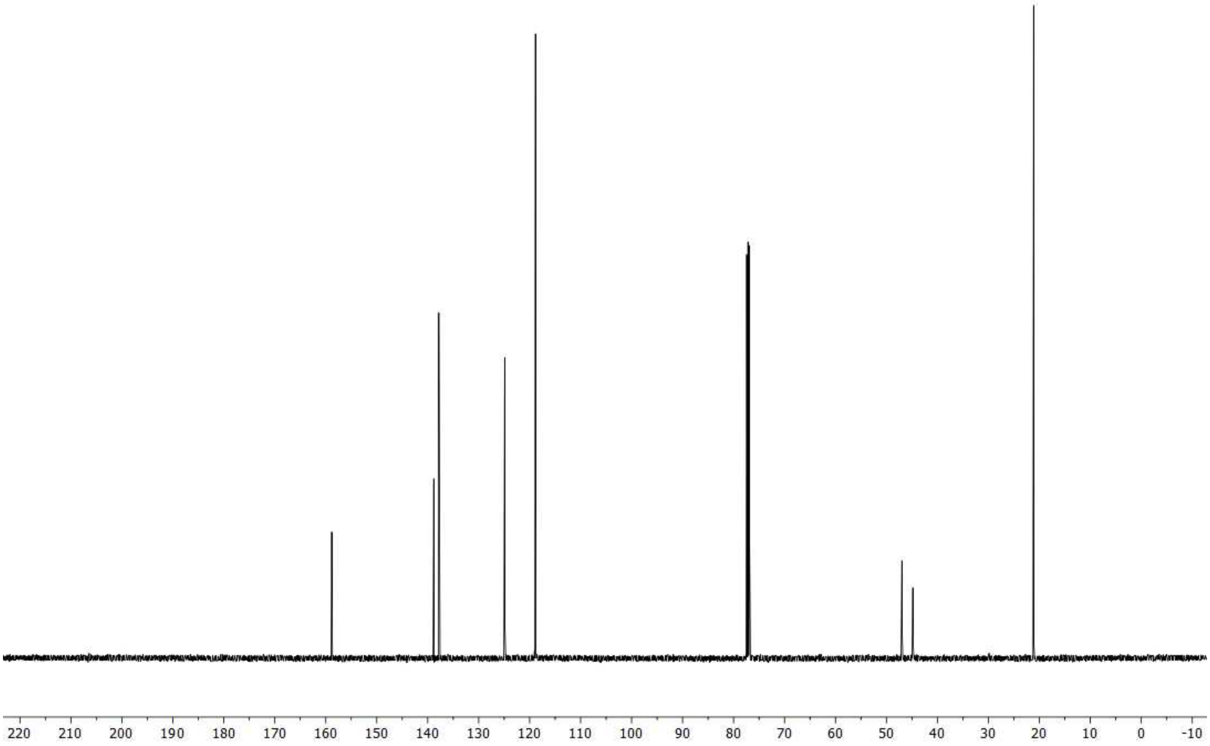

Figure S80 – ^{13}C NMR spectrum of **1e** ($CDCl_3$, 126 MHz).

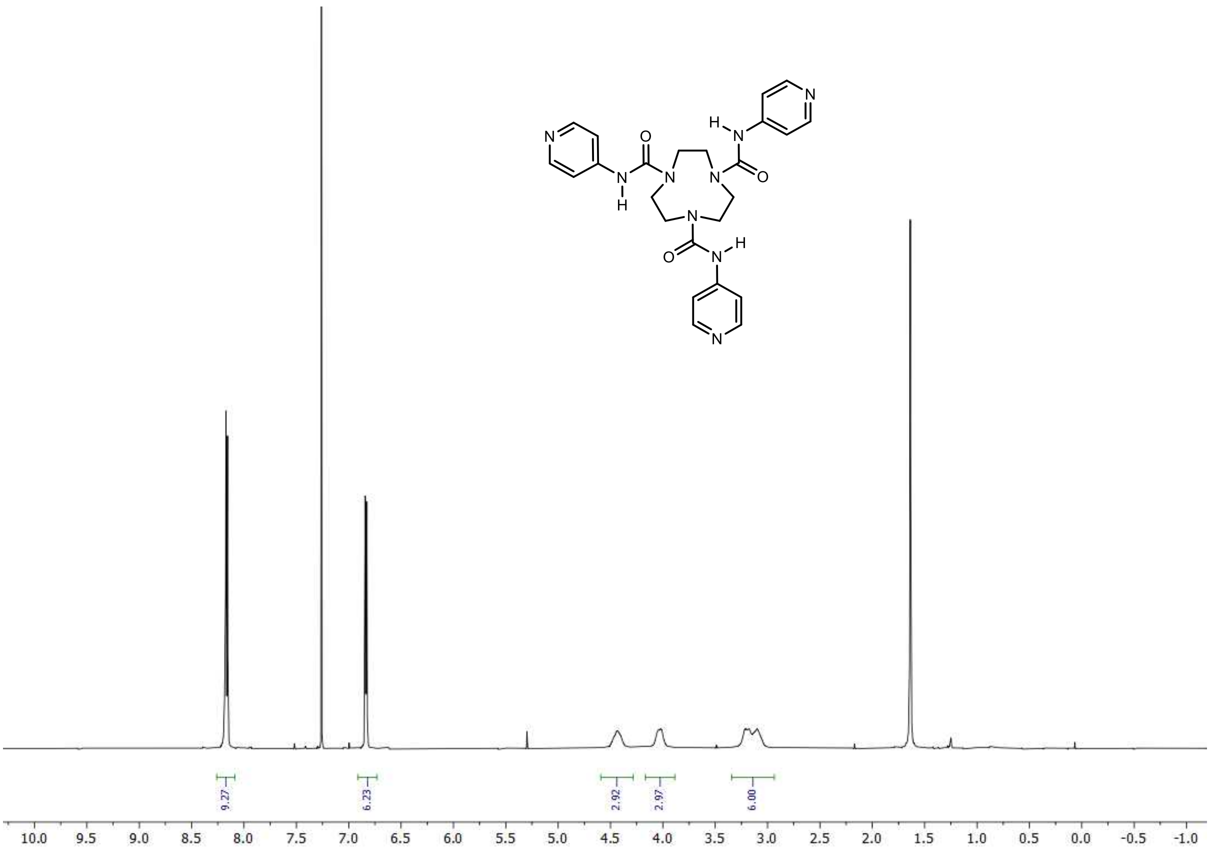

Figure S81 – ^{1}H NMR spectrum of **1f** ($CDCl_3$, 400 MHz).

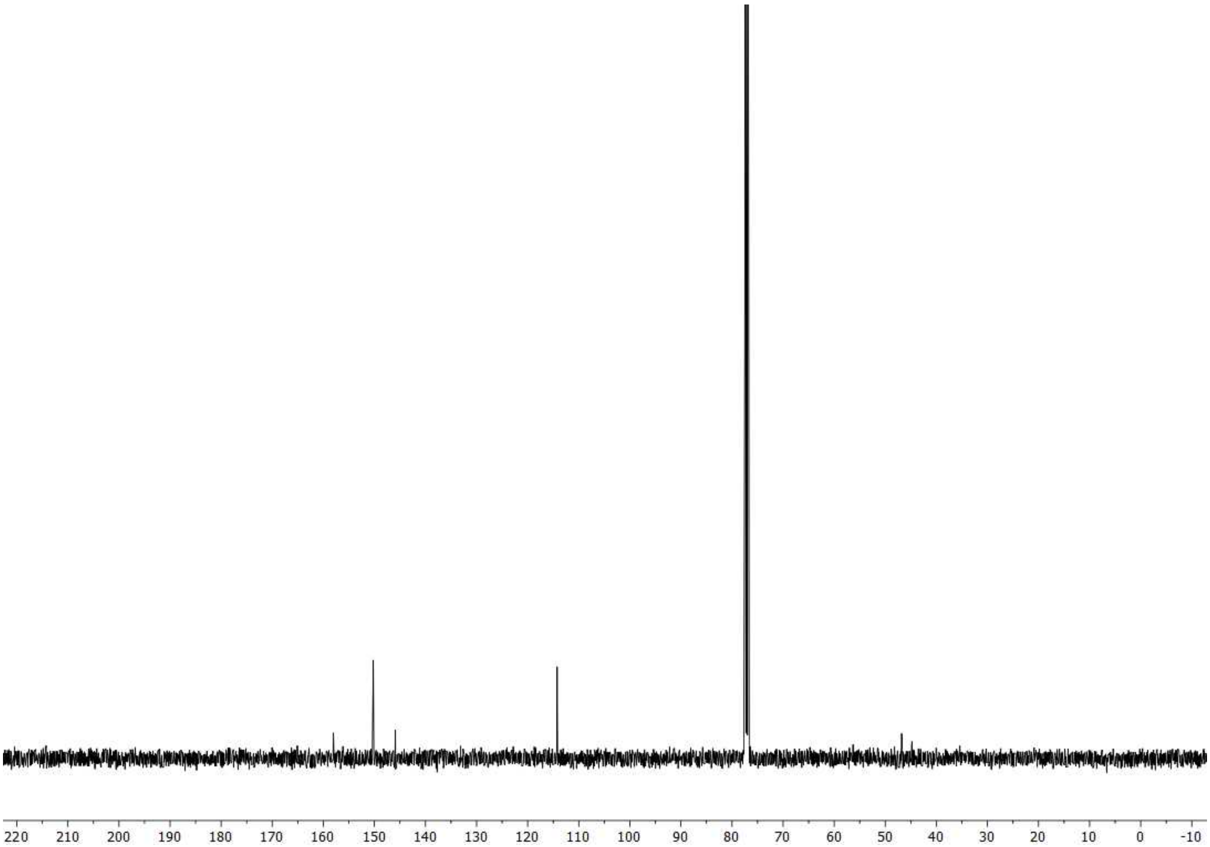

Figure S82 – ^{13}C NMR spectrum of **1f** ($CDCl_3$, 101 MHz).

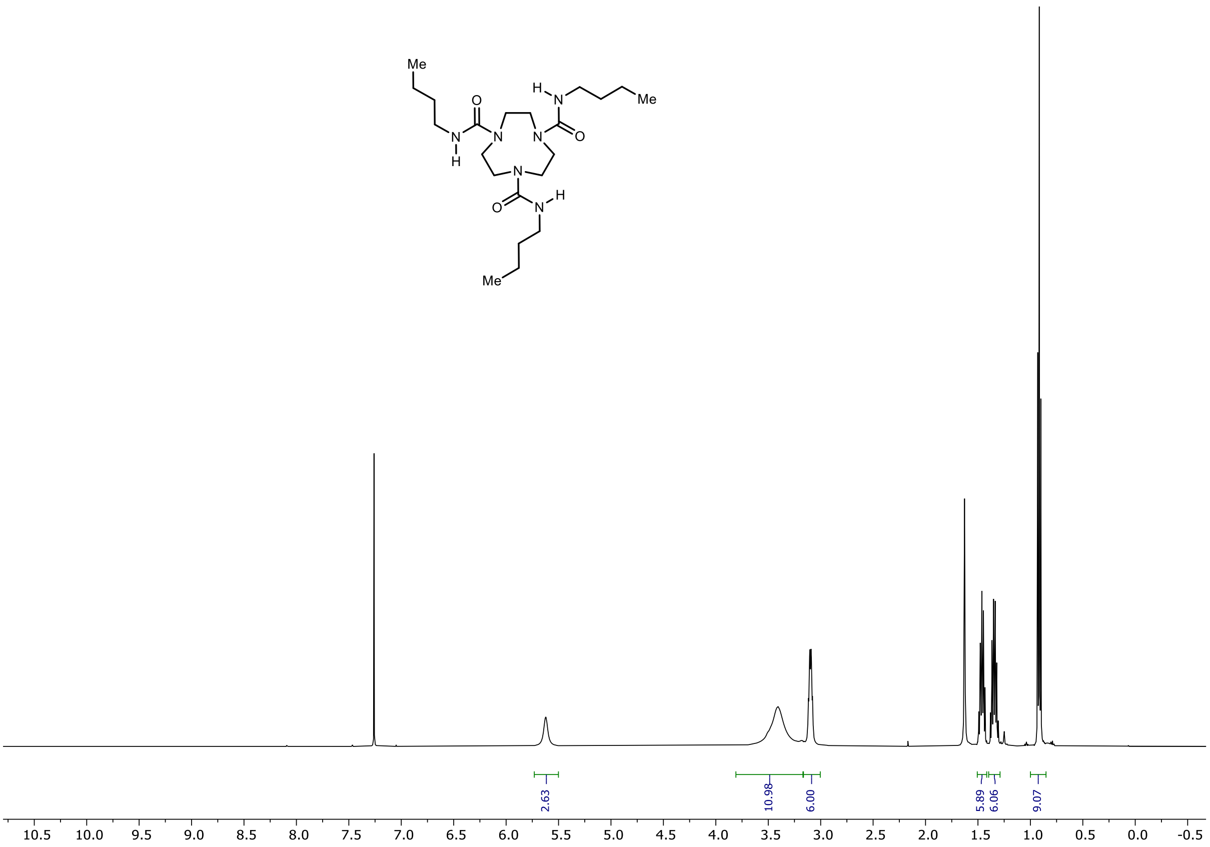

Figure S83 – ^{1}H NMR spectrum of **1g** ($CDCl_3$, 500 MHz).

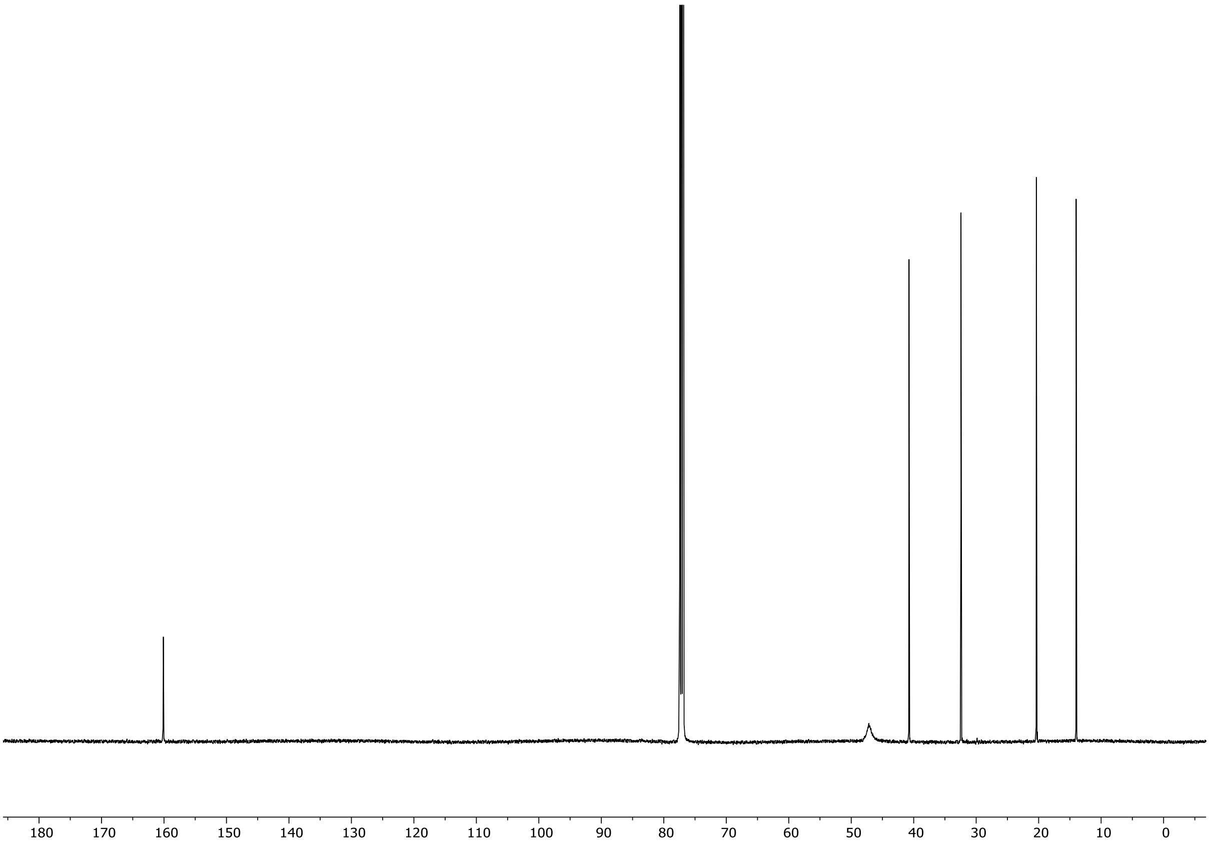

Figure S84 – ^{13}C NMR spectrum of **1g** ($CDCl_3$, 126 MHz).

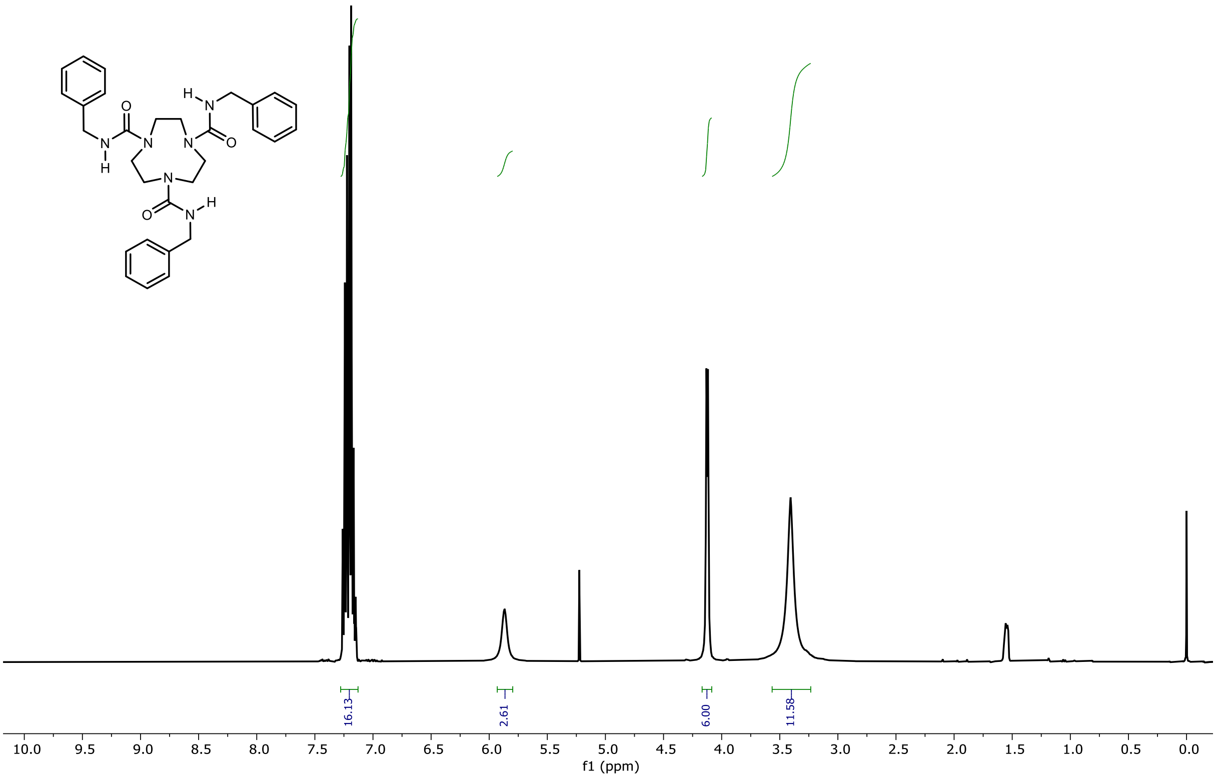

Figure S85 – ^{1}H NMR spectrum of **1h** ($CDCl_3$, 400 MHz).

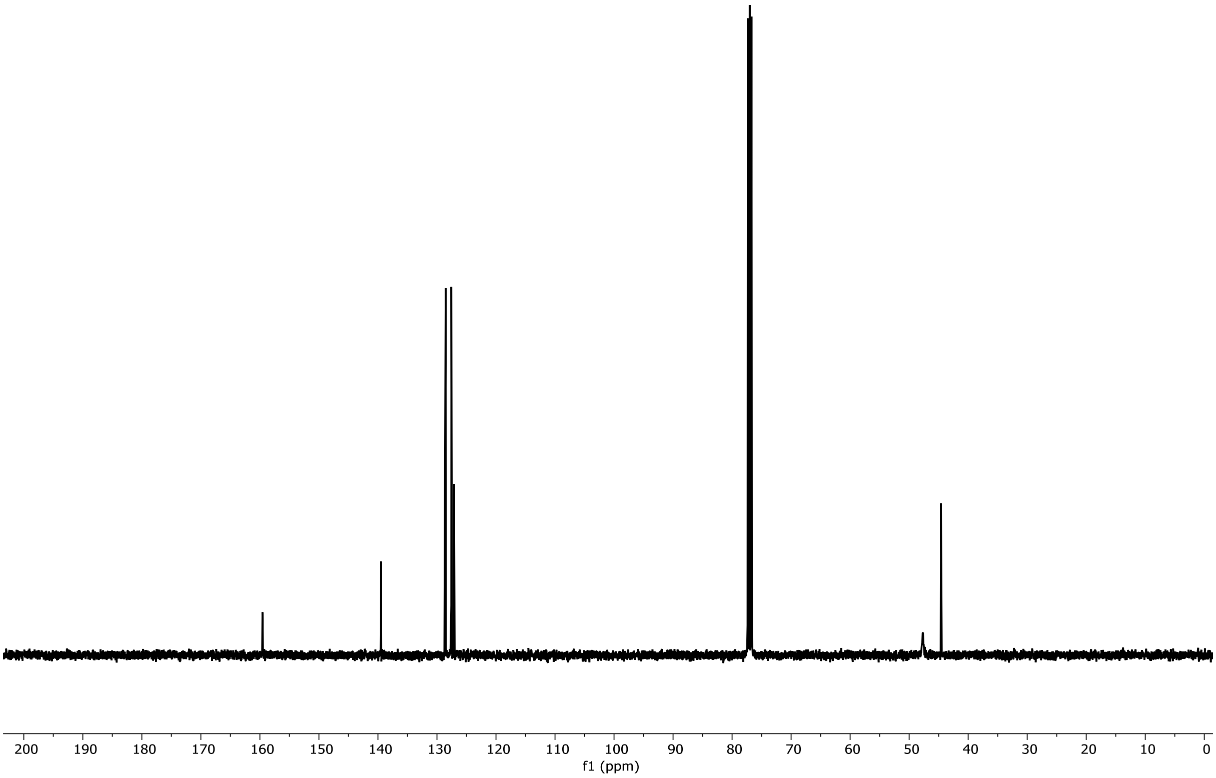

Figure S86 – ^{13}C NMR spectrum of **1h** ($CDCl_3$, 101 MHz).

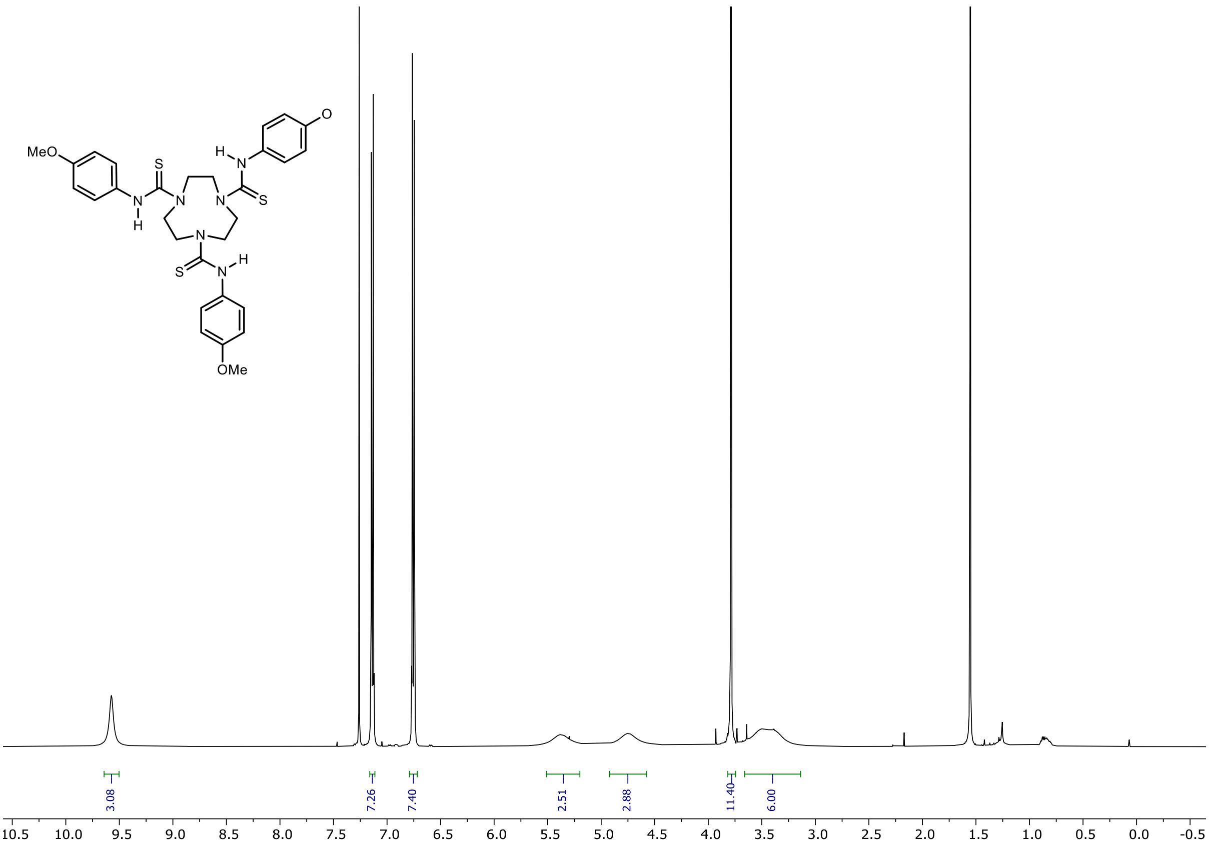

Figure S87 – ^{1}H NMR spectrum of **2a** ($CDCl_3$, 500 MHz).

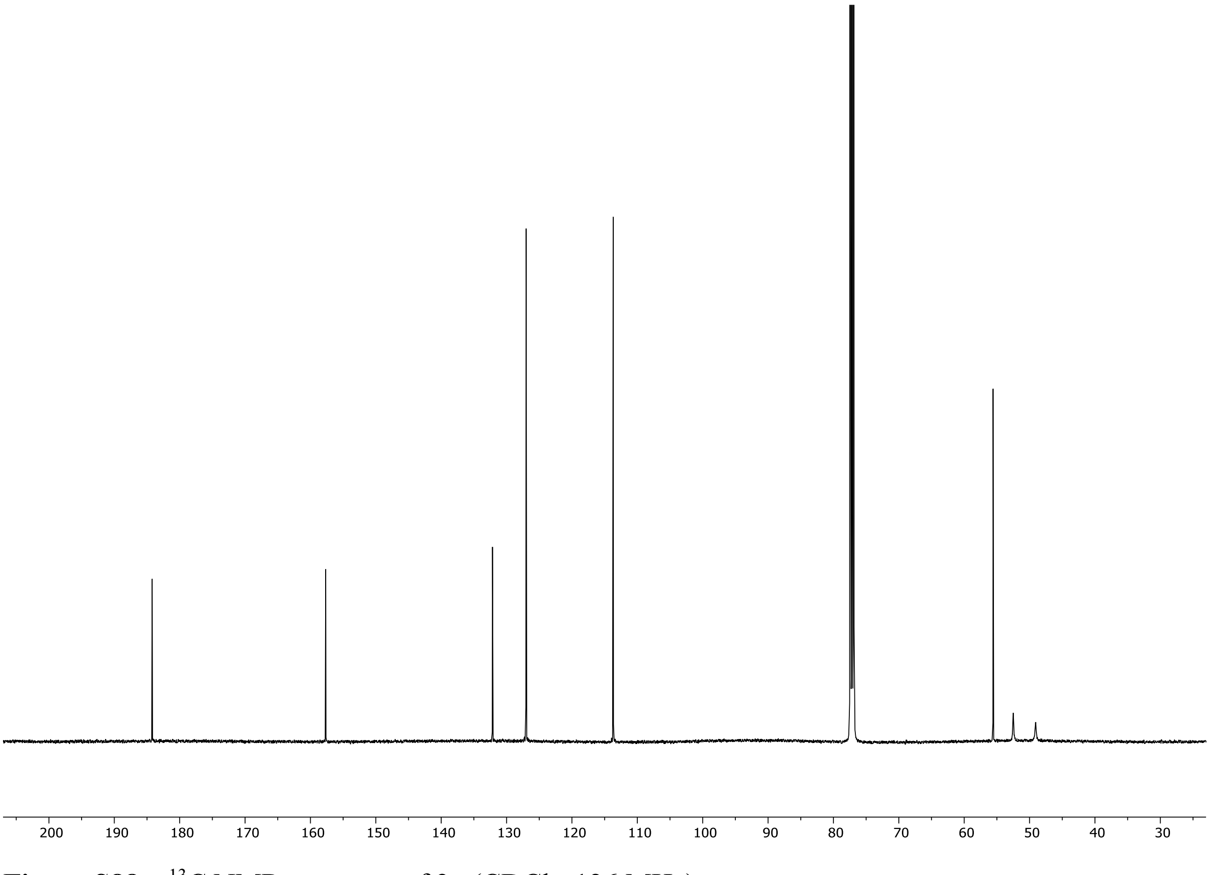

Figure S88 – ^{13}C NMR spectrum of **2a** ($CDCl_3$, 126 MHz).

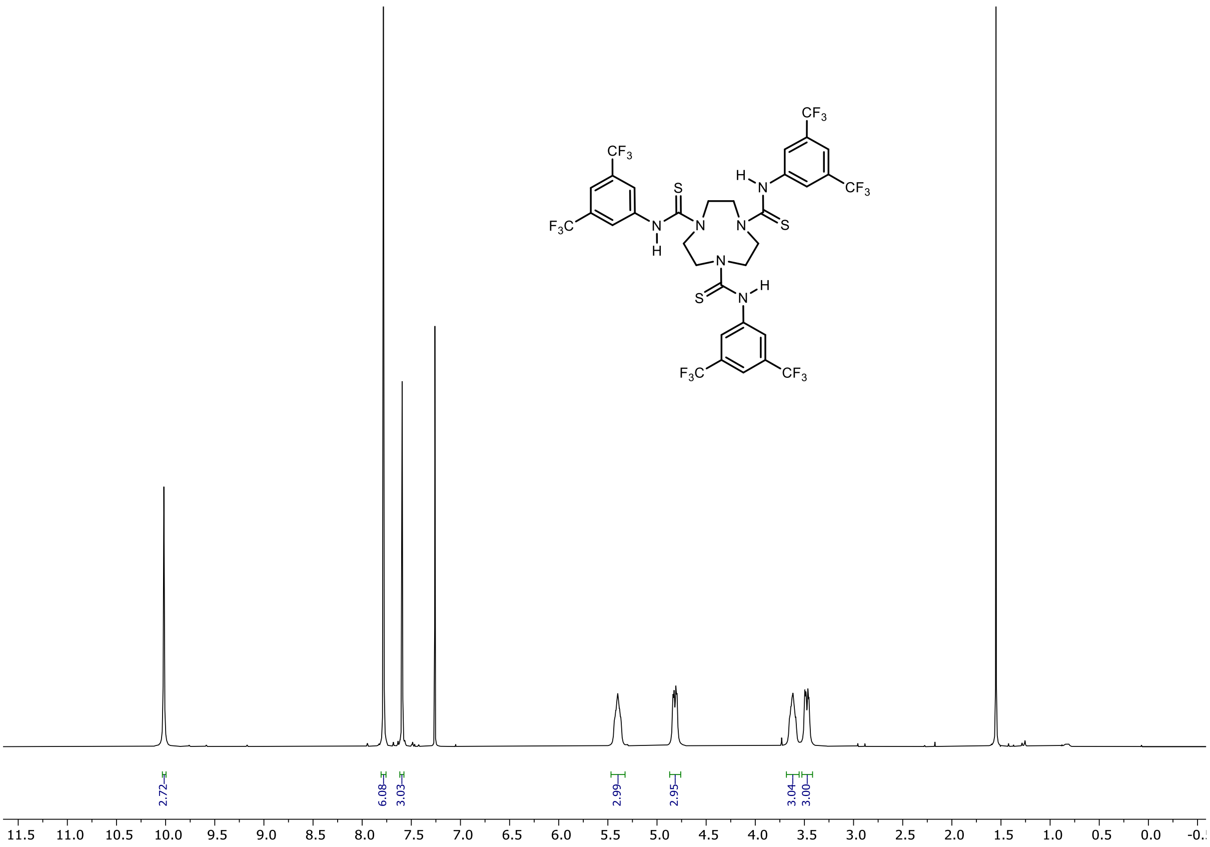

Figure S89 – ^{1}H NMR spectrum of **2b** ($CDCl_3$, 500 MHz).

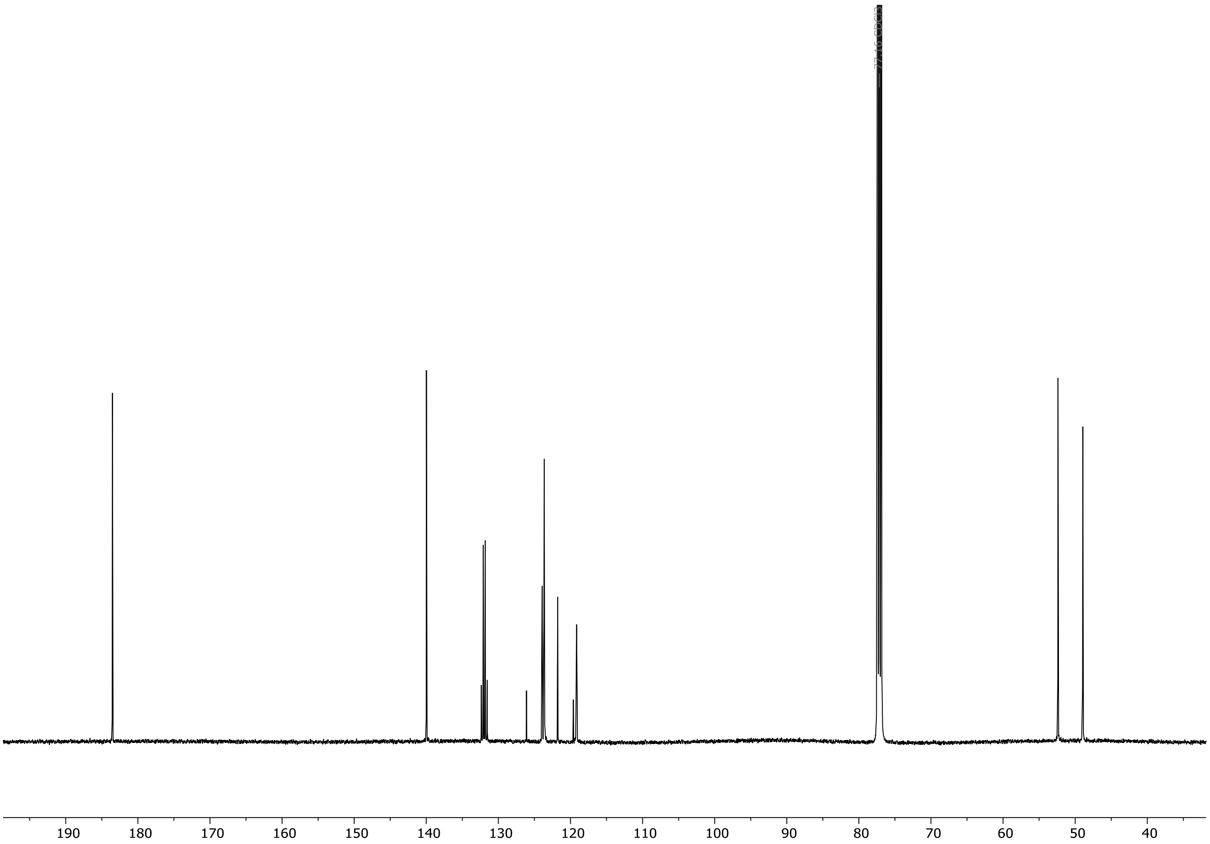

Figure S90 – ^{13}C NMR spectrum of **2b** ($CDCl_3$, 126 MHz).

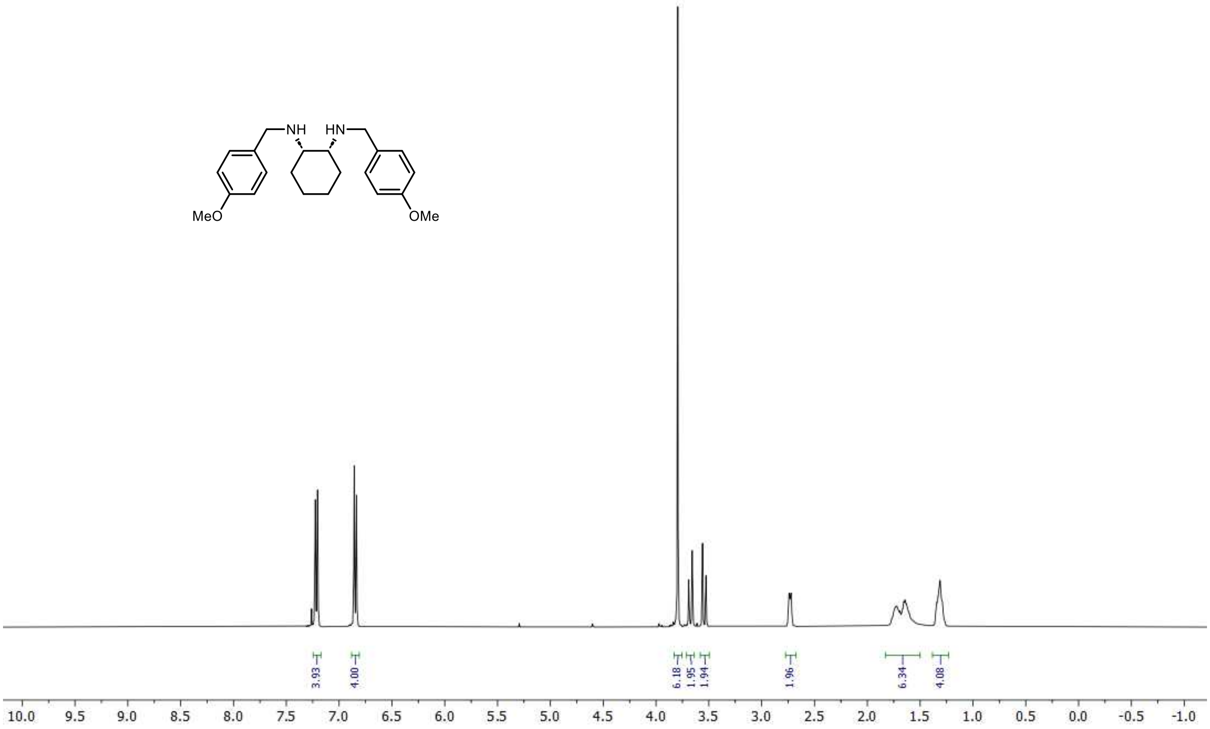

Figure S91 – ^{1}H NMR spectrum of **3-1** ($CDCl_3$, 400 MHz).

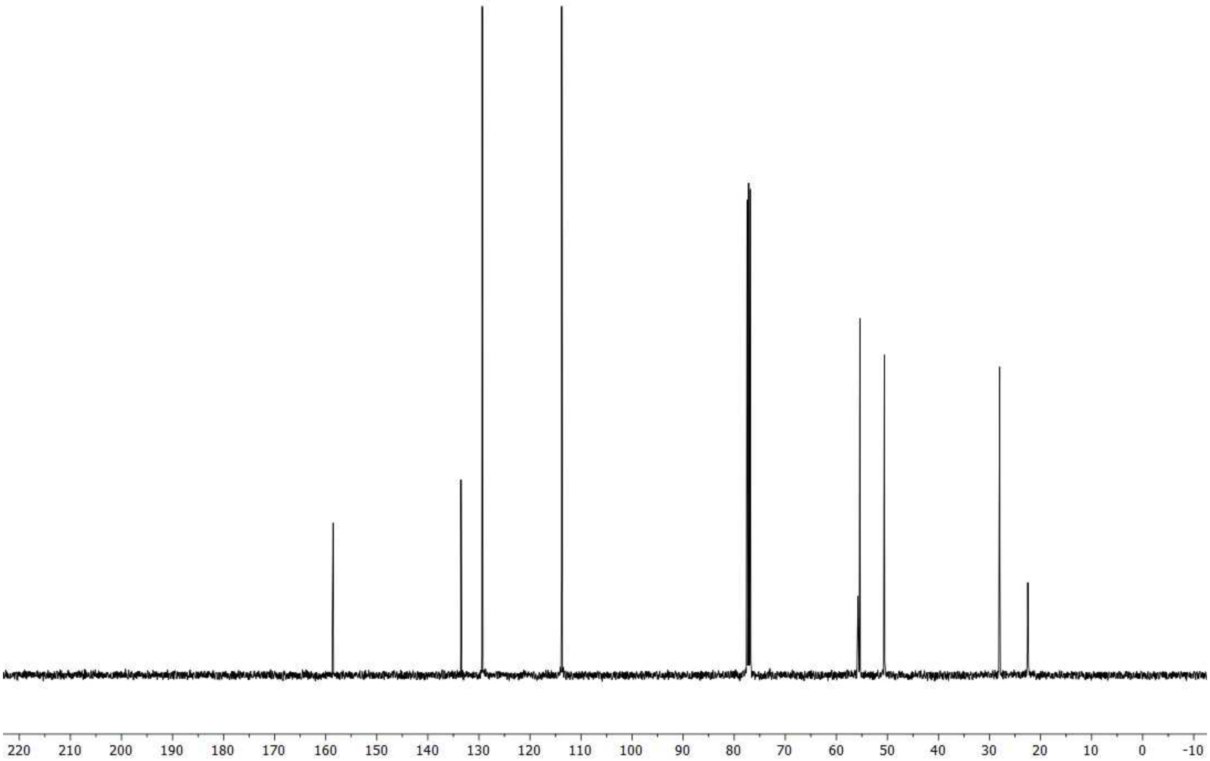

Figure S92 – ^{13}C NMR spectrum of **3-1** ($CDCl_3$, 101 MHz).

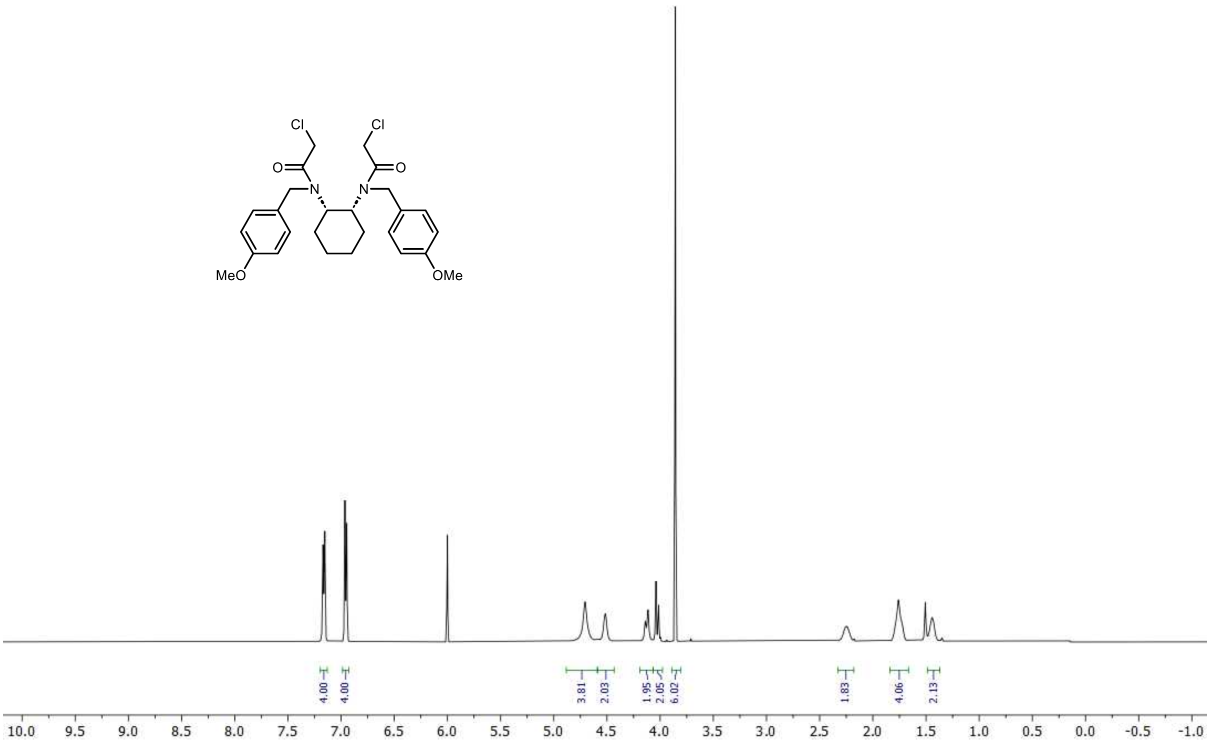

Figure S93 – ^{1}H NMR spectrum of **3-2** ($C_2D_2Cl_4$, 500 MHz, 110 °C).

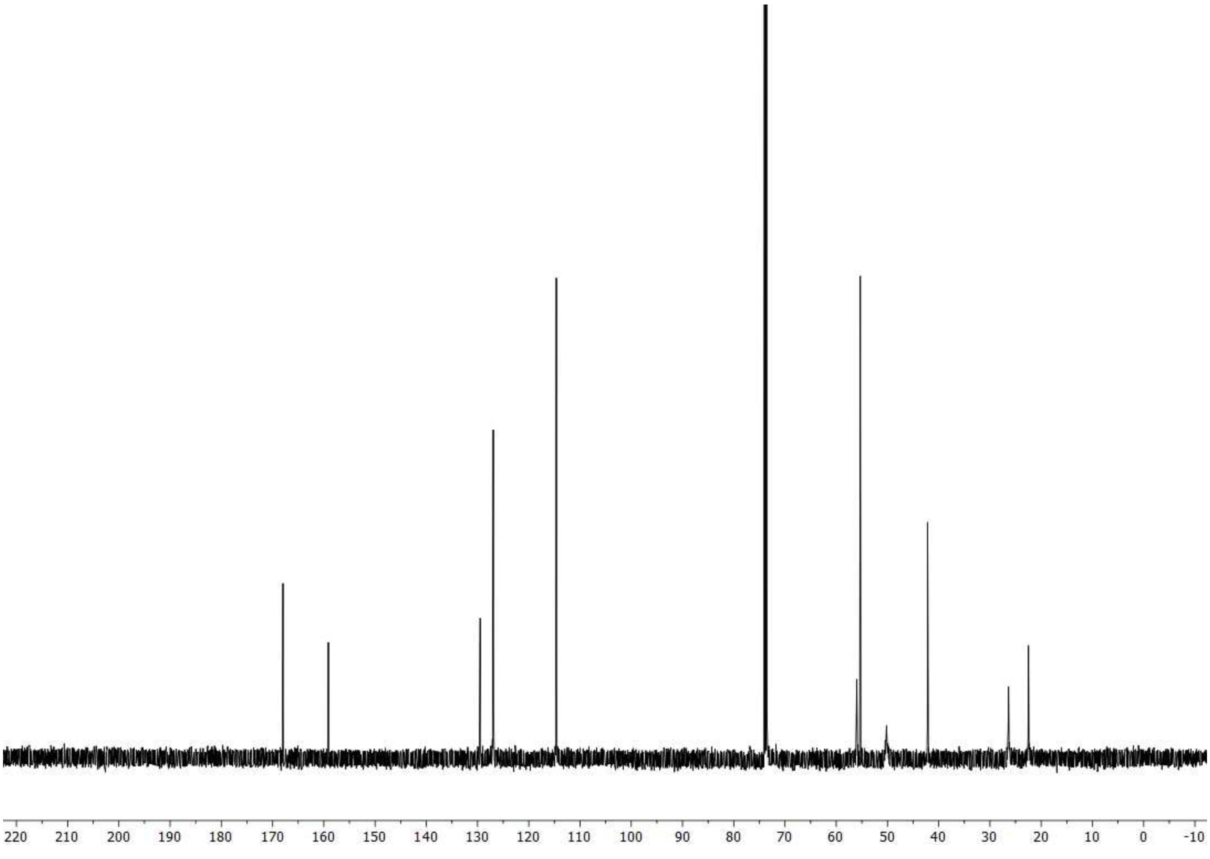

Figure S94 – ^{13}C NMR spectrum of **3-2** ($C_2D_2Cl_4$, 126 MHz, 110 °C).

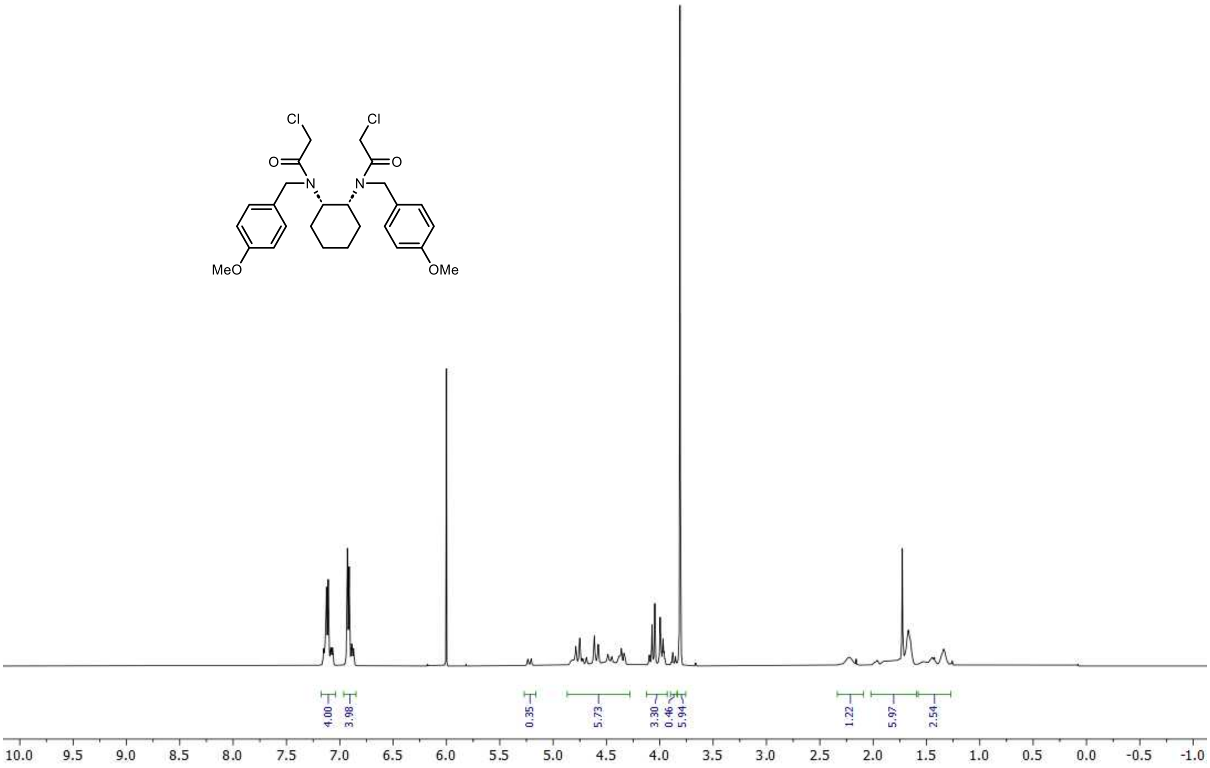

Figure S95 – ^{1}H NMR spectrum of **3-2** ($C_2D_2Cl_4$, 500 MHz, 25 °C).

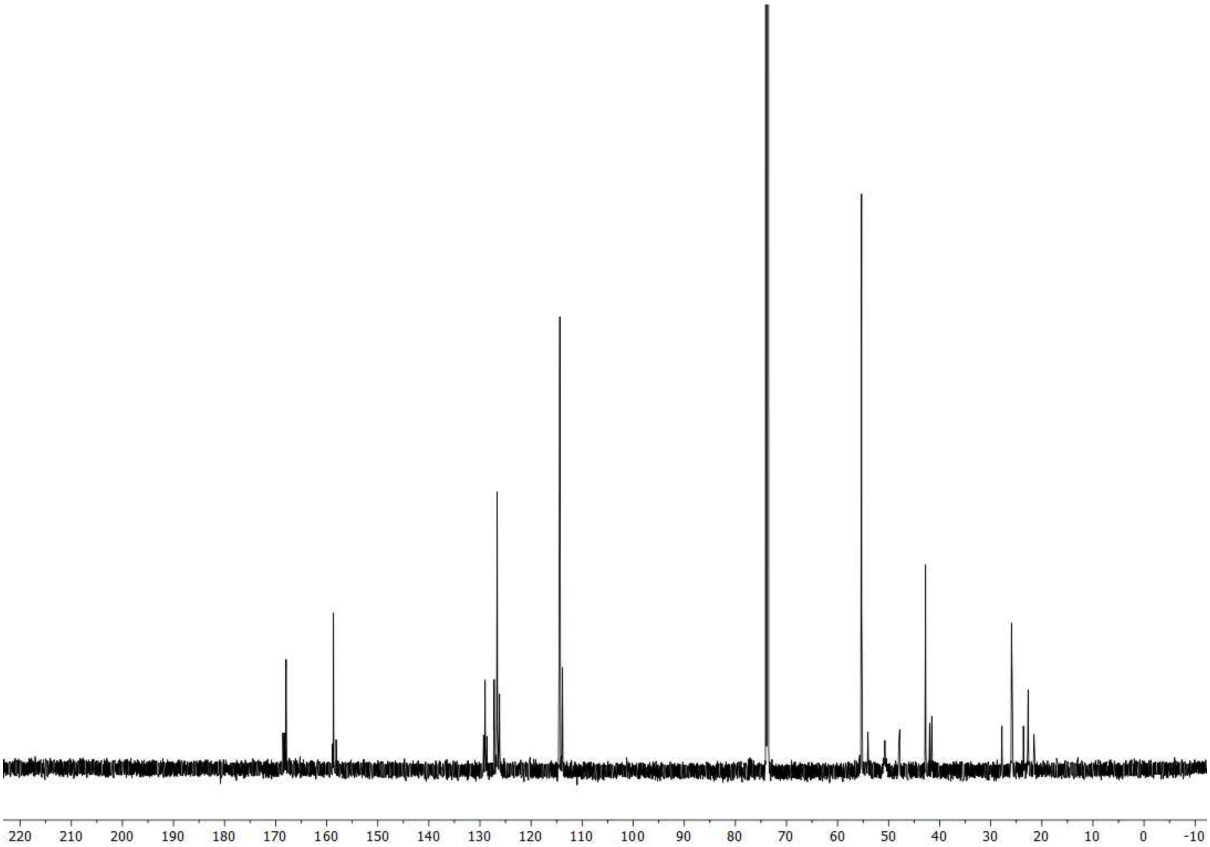

Figure S96 – ^{13}C NMR spectrum of **3-2** ($C_2D_2Cl_4$, 126 MHz, 25 °C).

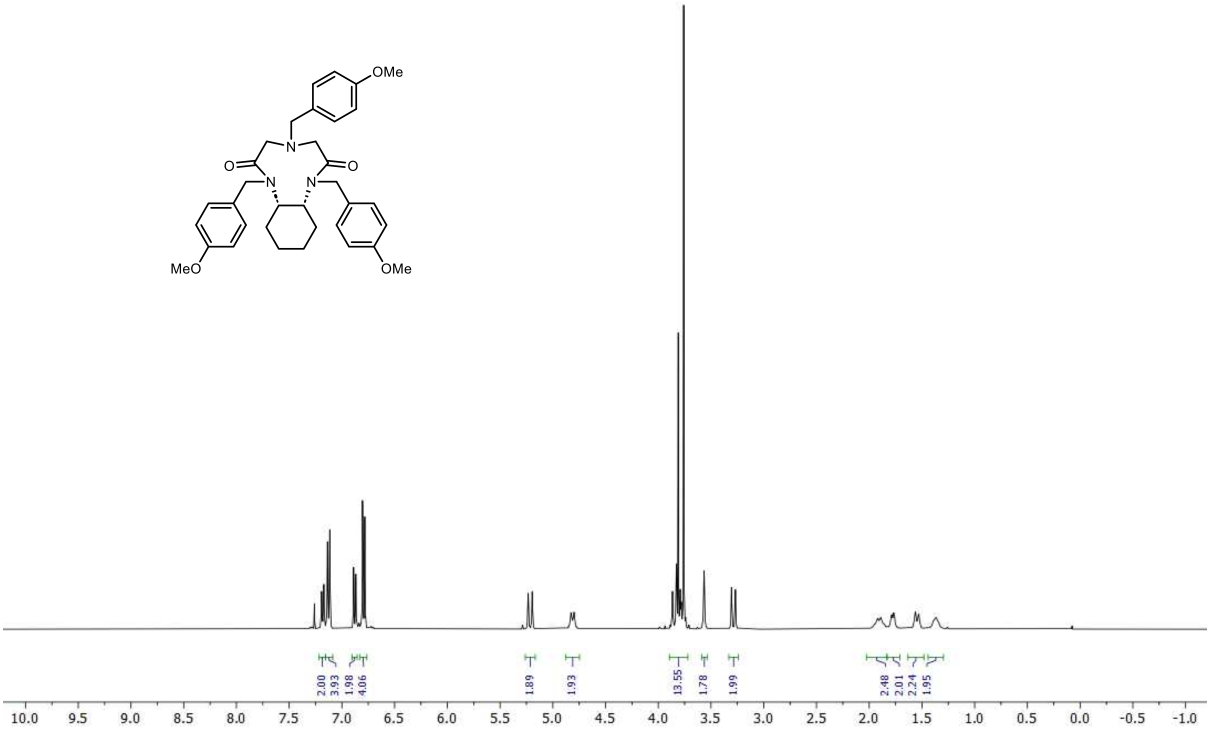

Figure S97 – ^{1}H NMR spectrum of **3-3** ($CDCl_3$, 400 MHz).

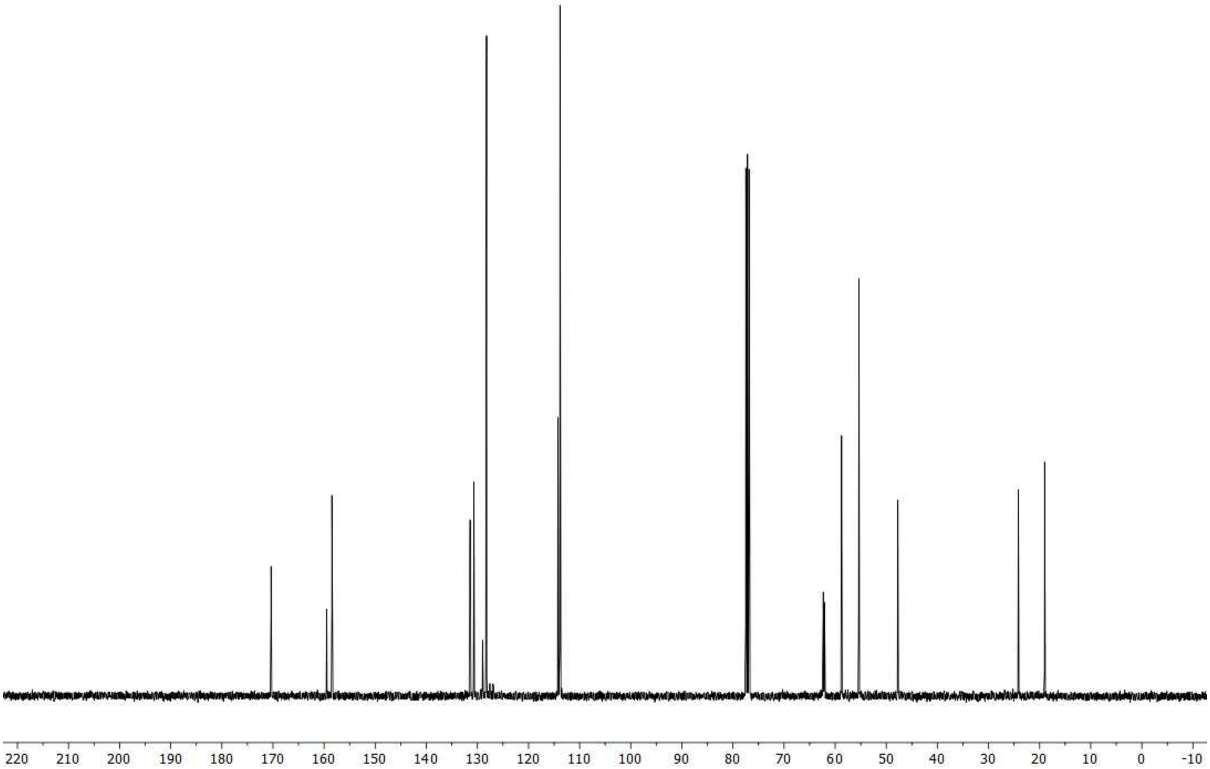

Figure S98 – ^{13}C NMR spectrum of **3-3** ($CDCl_3$, 101 MHz).

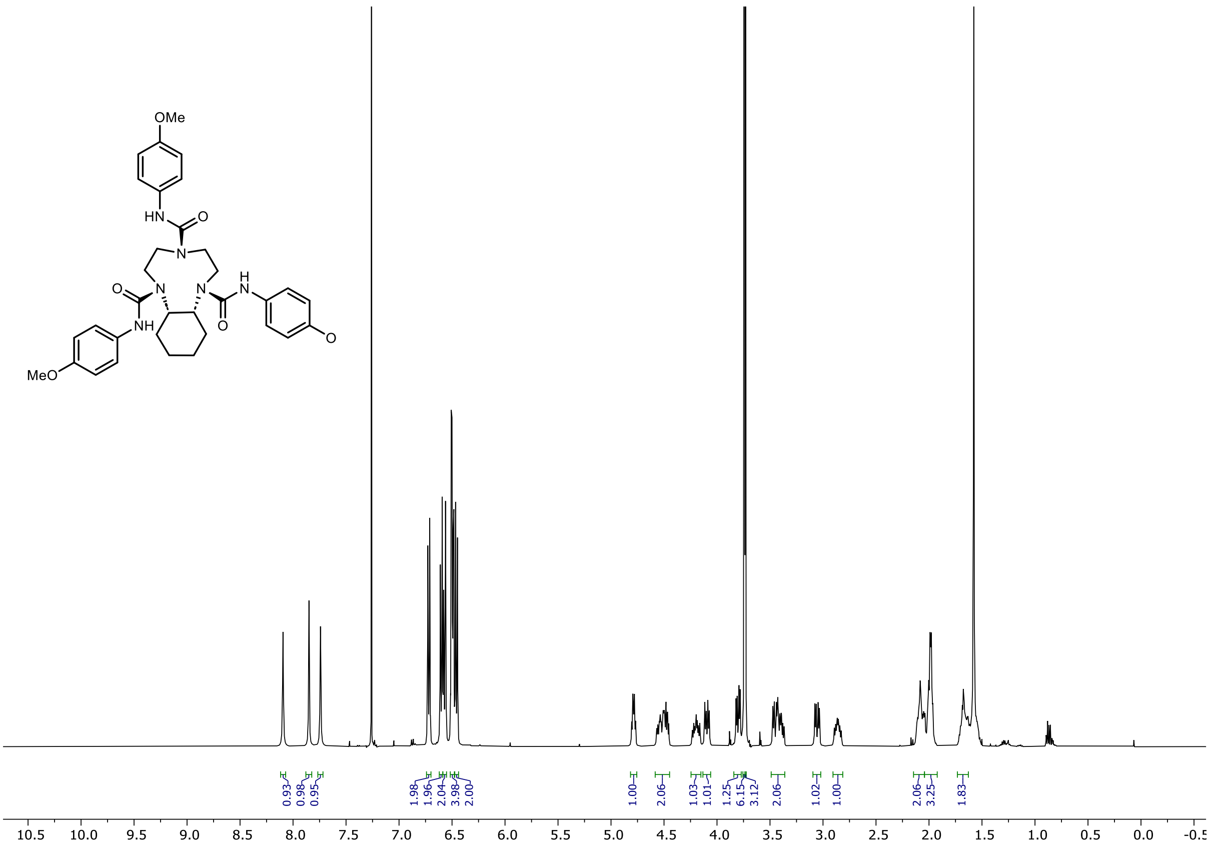

Figure S99 – ^{1}H NMR spectrum of **3** ($CDCl_3$, 500 MHz).

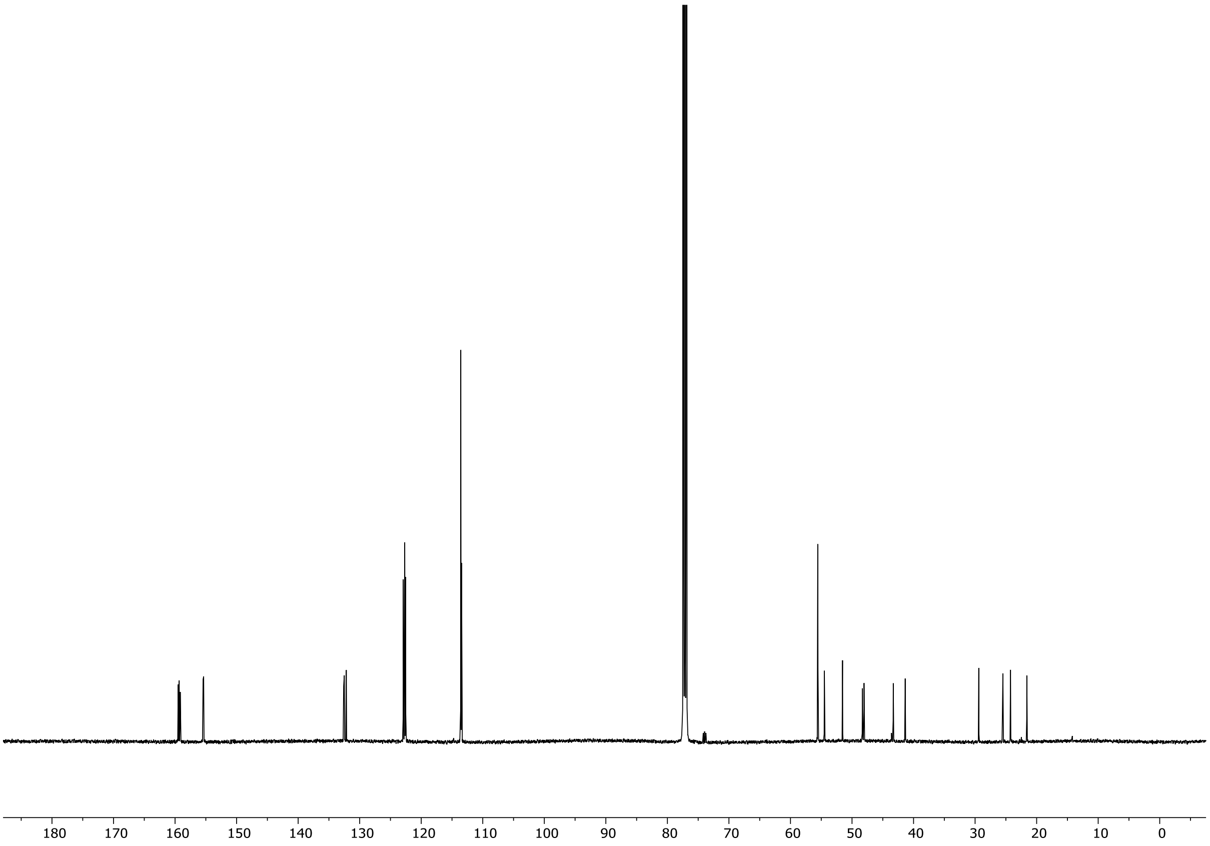

Figure S100 – ^{13}C NMR spectrum of **3** ($CDCl_3$, 126 MHz).

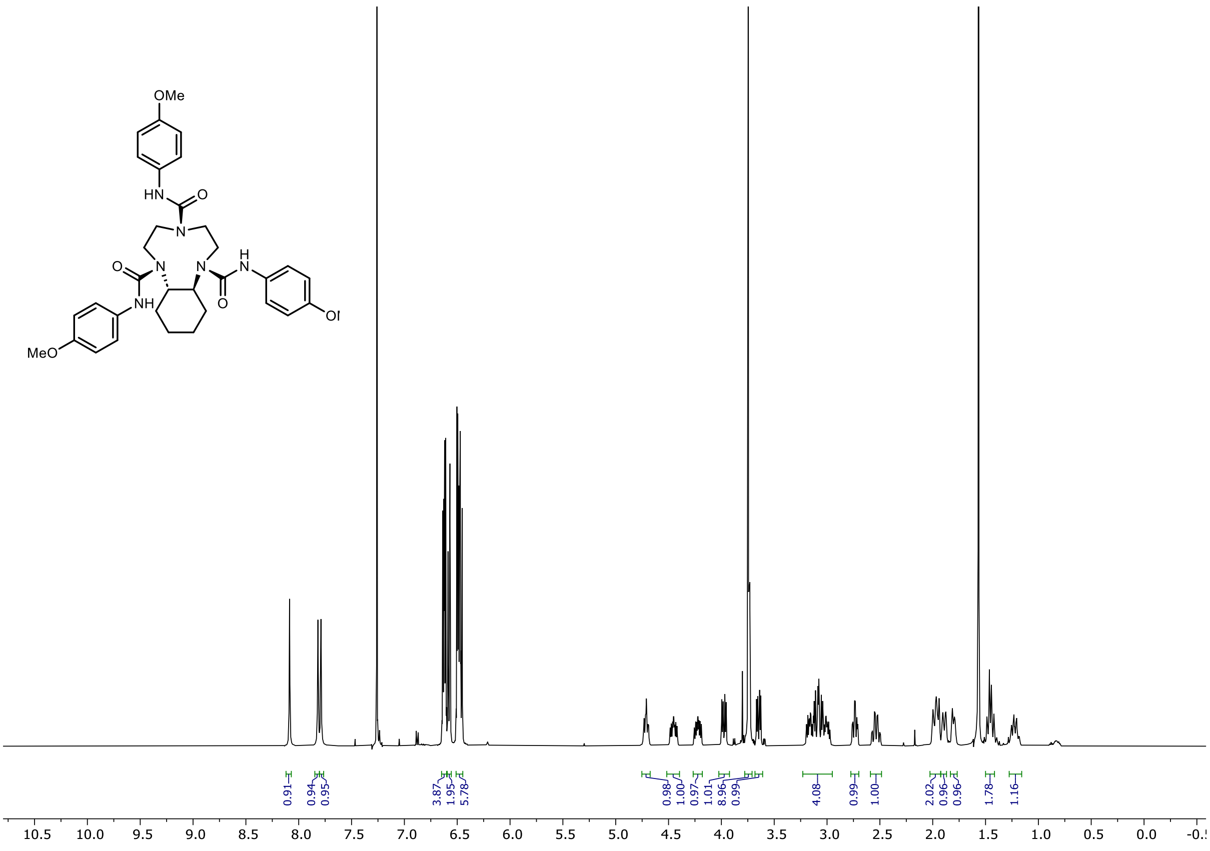

Figure S101 – ^{1}H NMR spectrum of (±)-**4** ($CDCl_3$, 500 MHz).

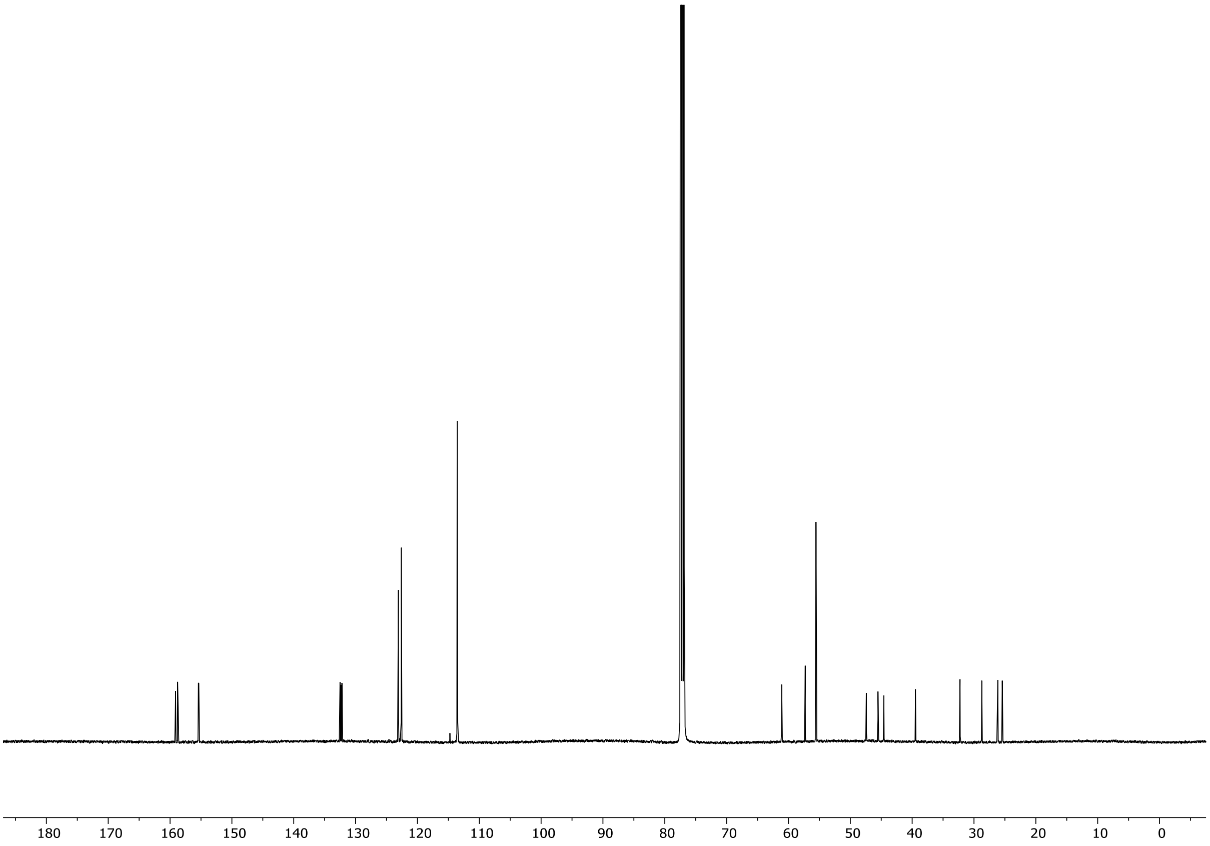

Figure S102 – ^{13}C NMR spectrum of (±)-**4** ($CDCl_3$, 126 MHz).

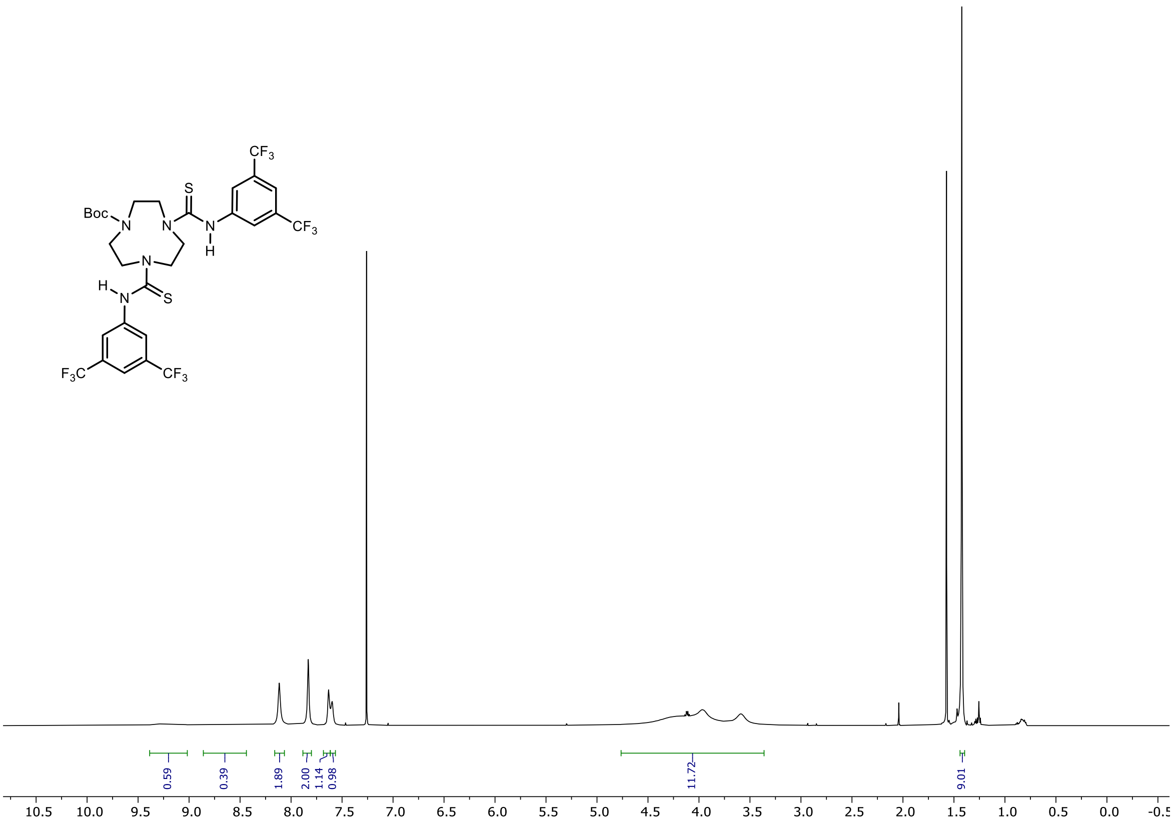

Figure S103 – ^{1}H NMR spectrum of **6** ($CDCl_3$, 500 MHz).

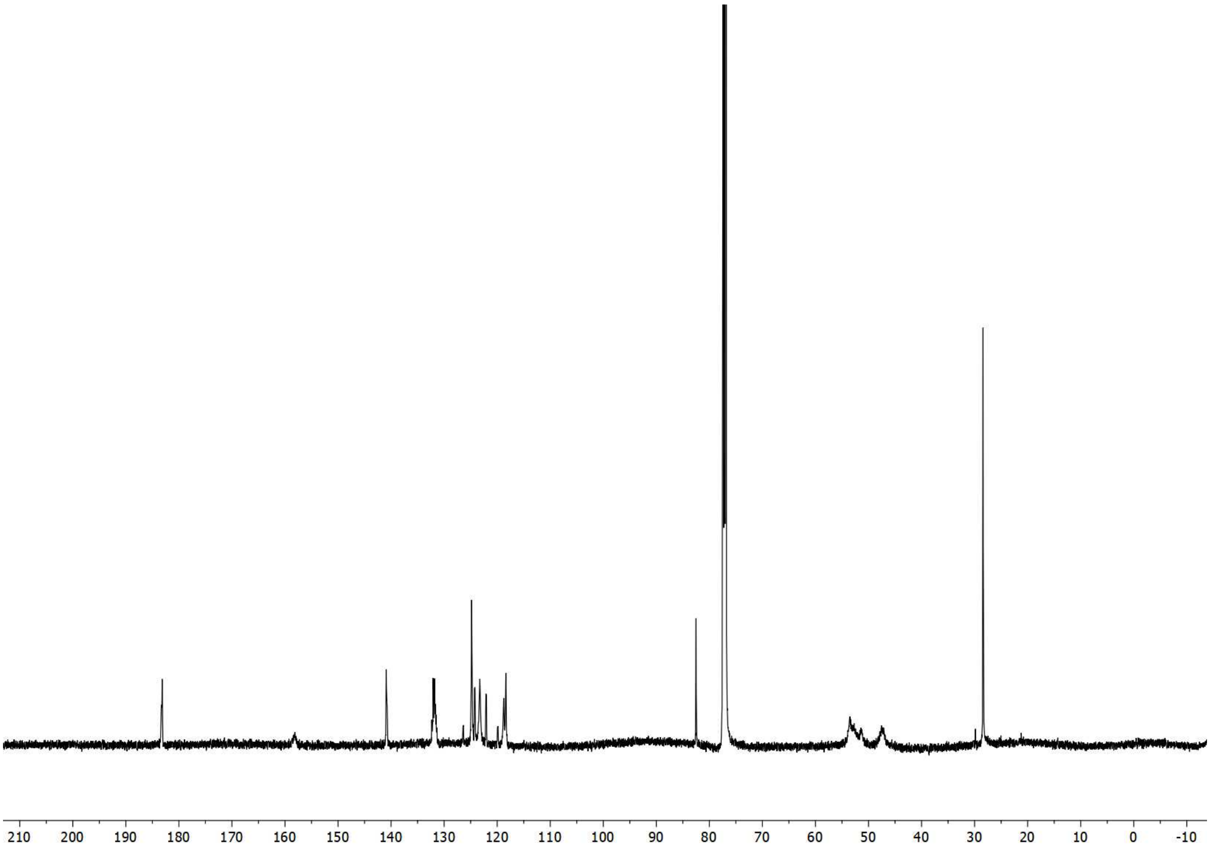

Figure S104 – ^{13}C NMR spectrum of **6** ($CDCl_3$, 126 MHz).

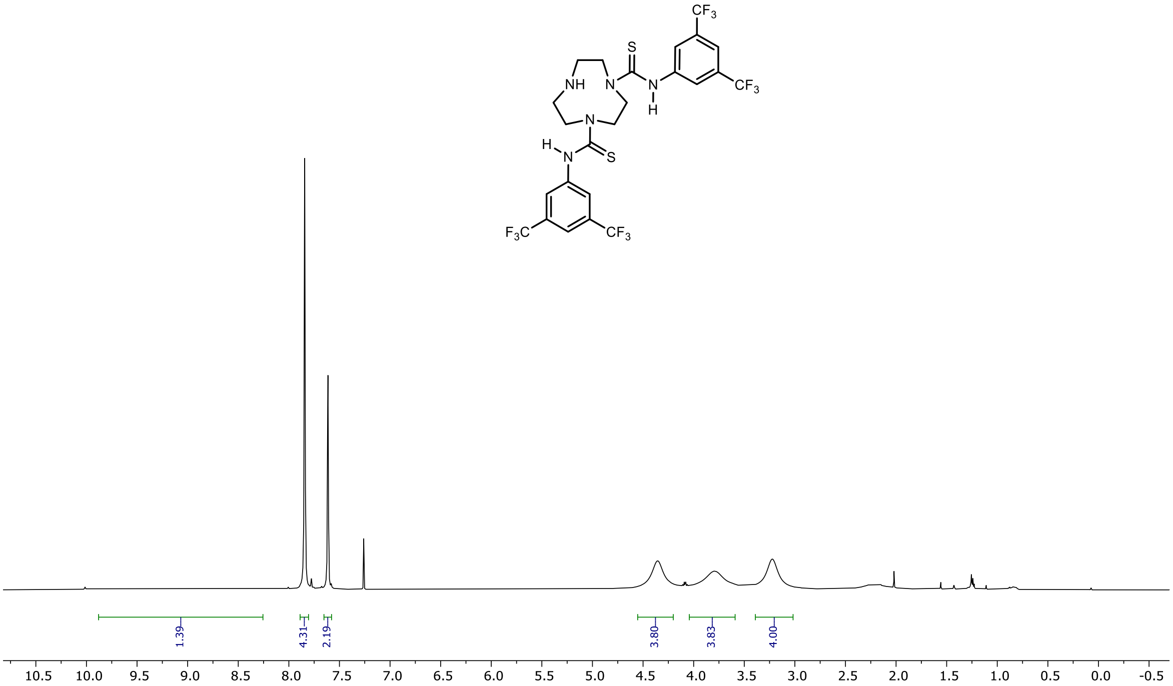

Figure S105 – ^{1}H NMR spectrum of **5-2** ($CDCl_3$, 500 MHz).

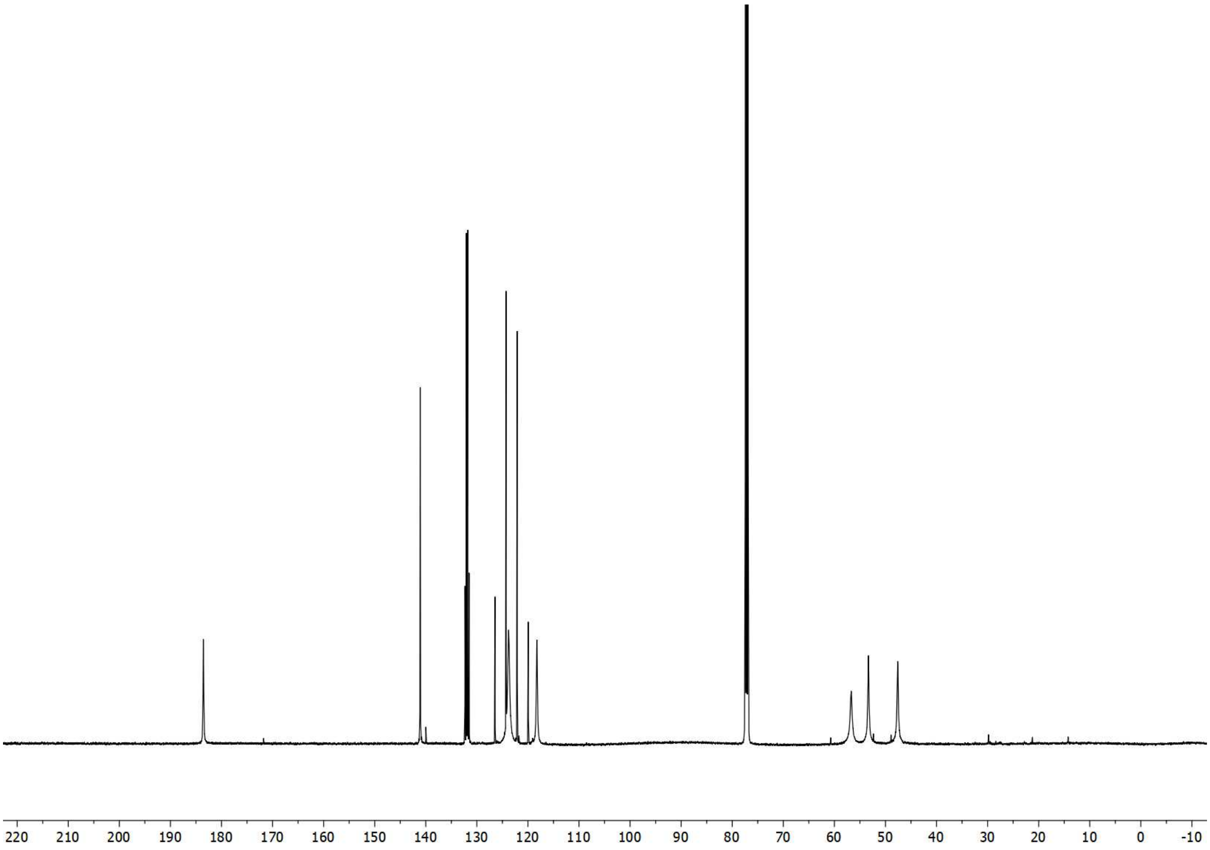

Figure S106 – ^{13}C NMR spectrum of **5-2** ($CDCl_3$, 126 MHz).

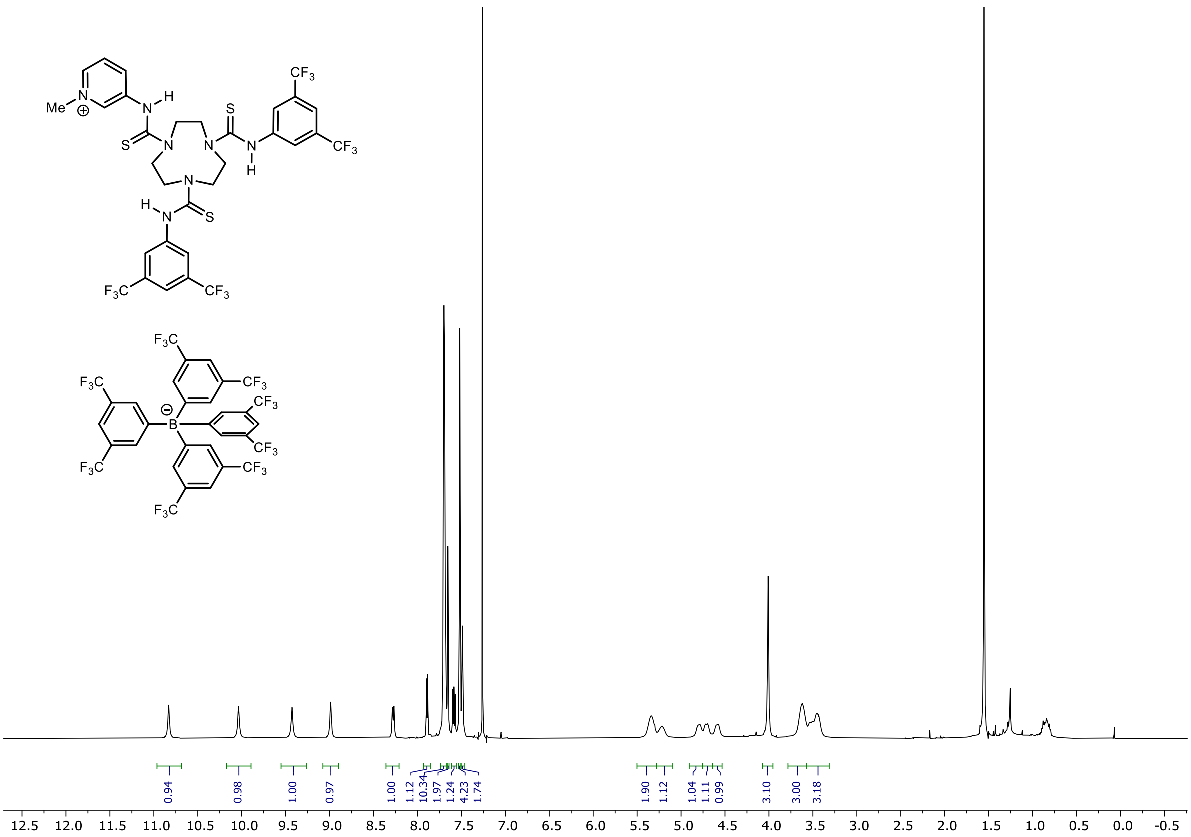

Figure S107 – ^{1}H NMR spectrum of **5** ($CDCl_3$, 500 MHz).

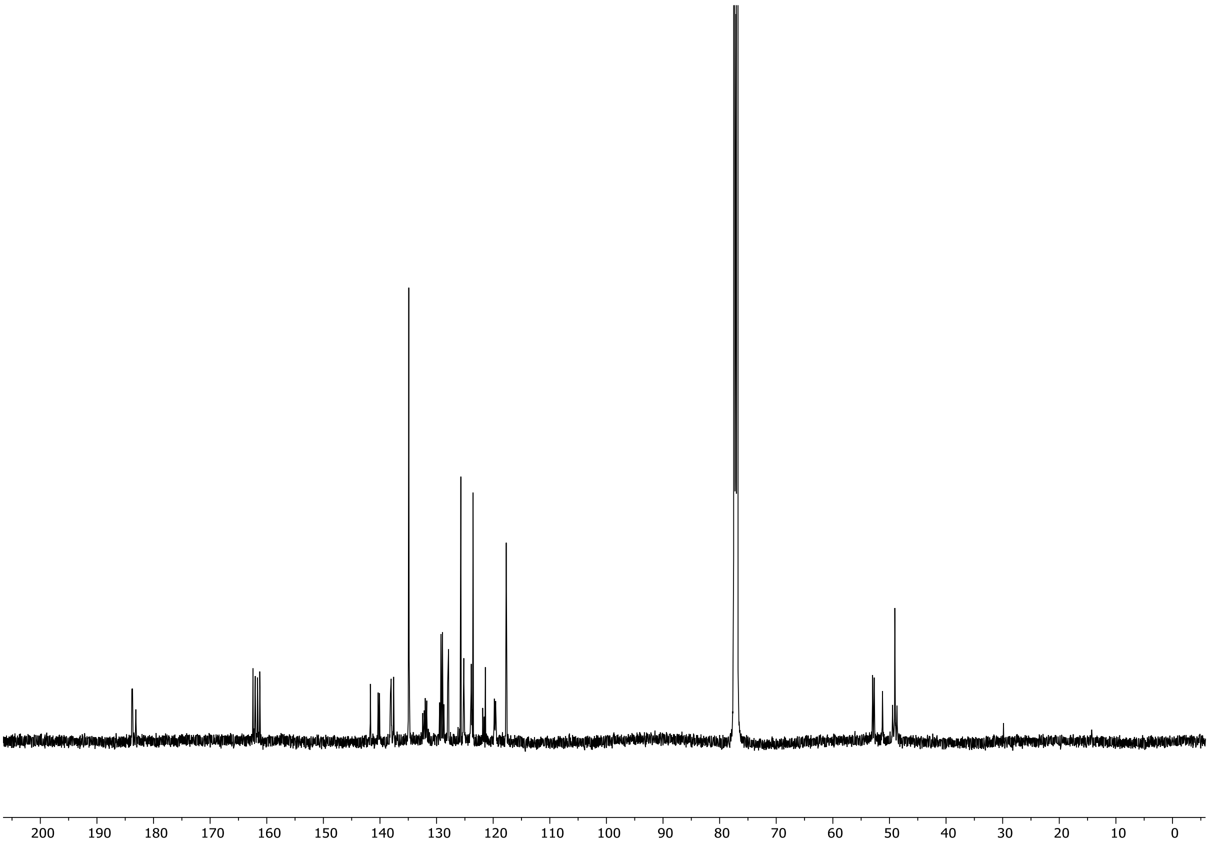

Figure S108 – ^{13}C NMR spectrum of **5** ($CDCl_3$, 126 MHz).

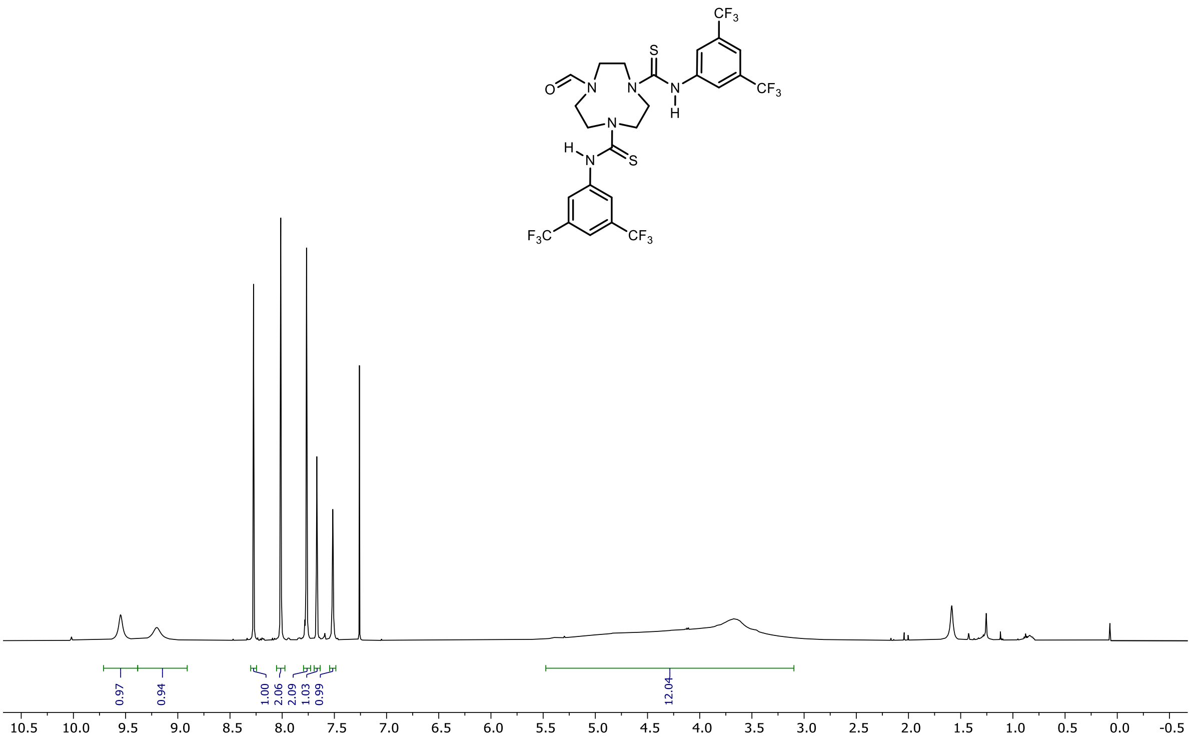

Figure S109 – ^{1}H NMR spectrum of **7** ($CDCl_3$, 500 MHz).

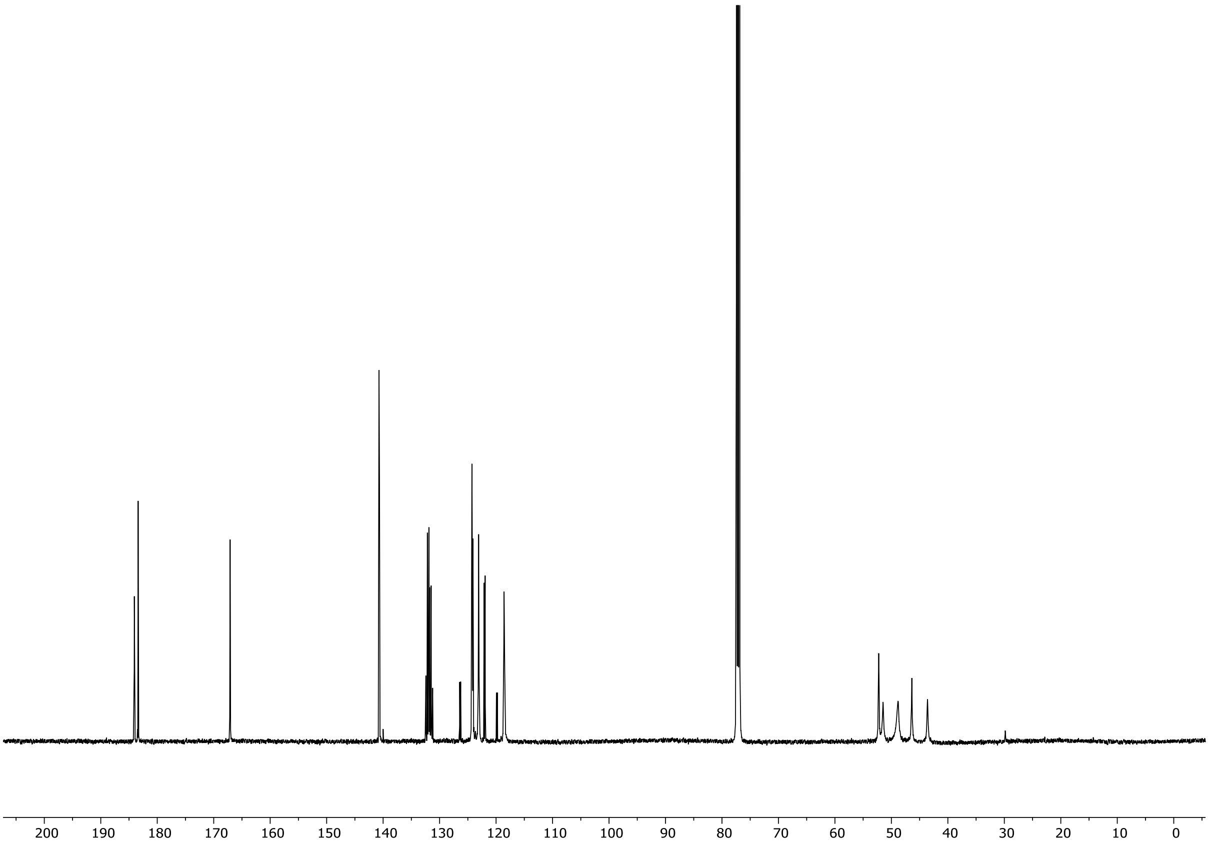

Figure S110 – ^{13}C NMR spectrum of **7** ($CDCl_3$, 126 MHz).

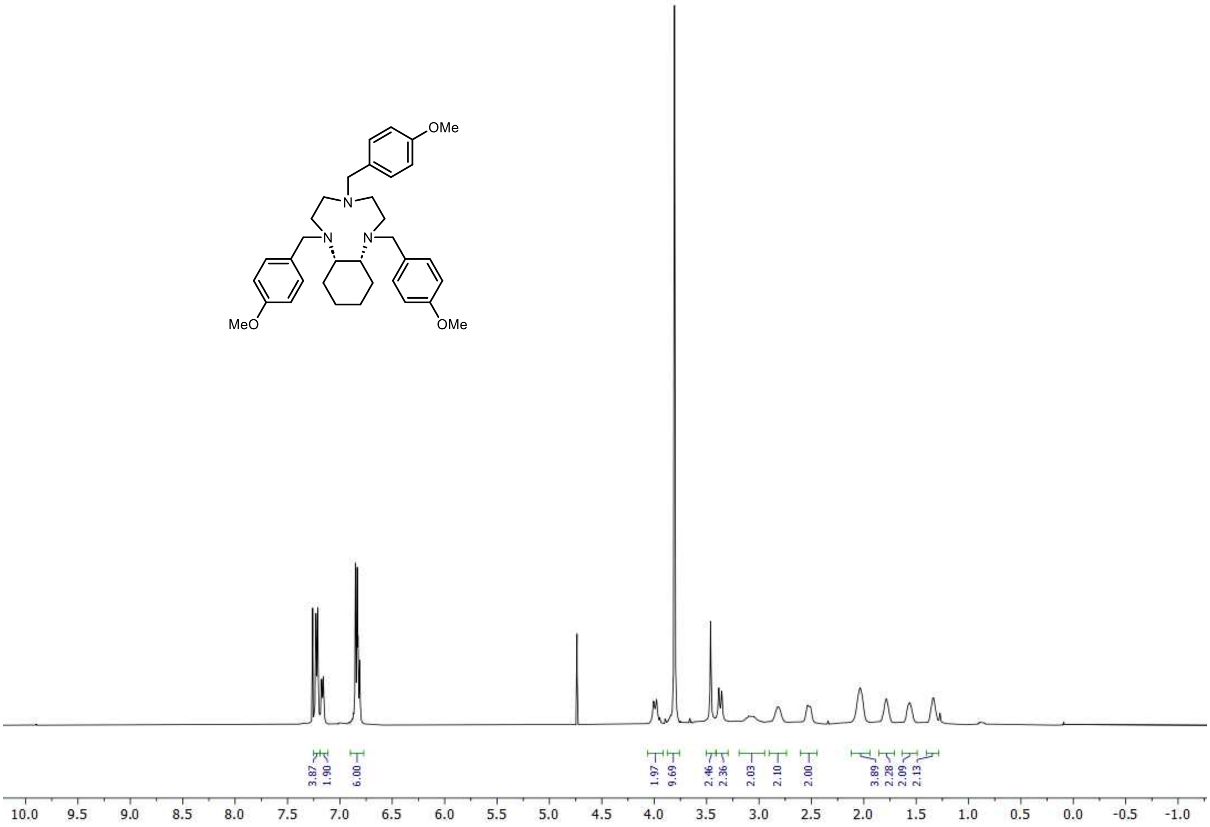

Figure S111 – ^{1}H NMR spectrum of **8-1** ($CDCl_3$, 500 MHz).

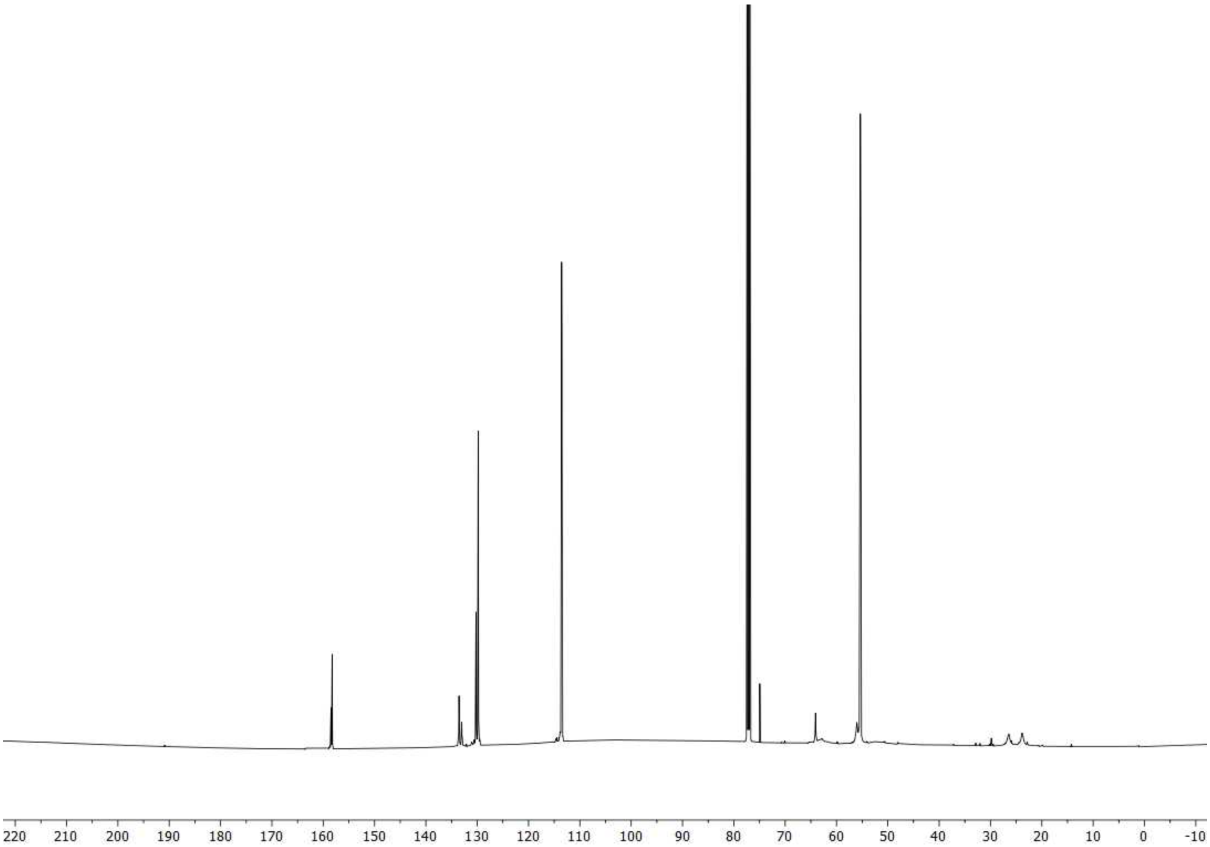

Figure S112 – ^{13}C NMR spectrum of **8-1** ($CDCl_3$, 126 MHz).

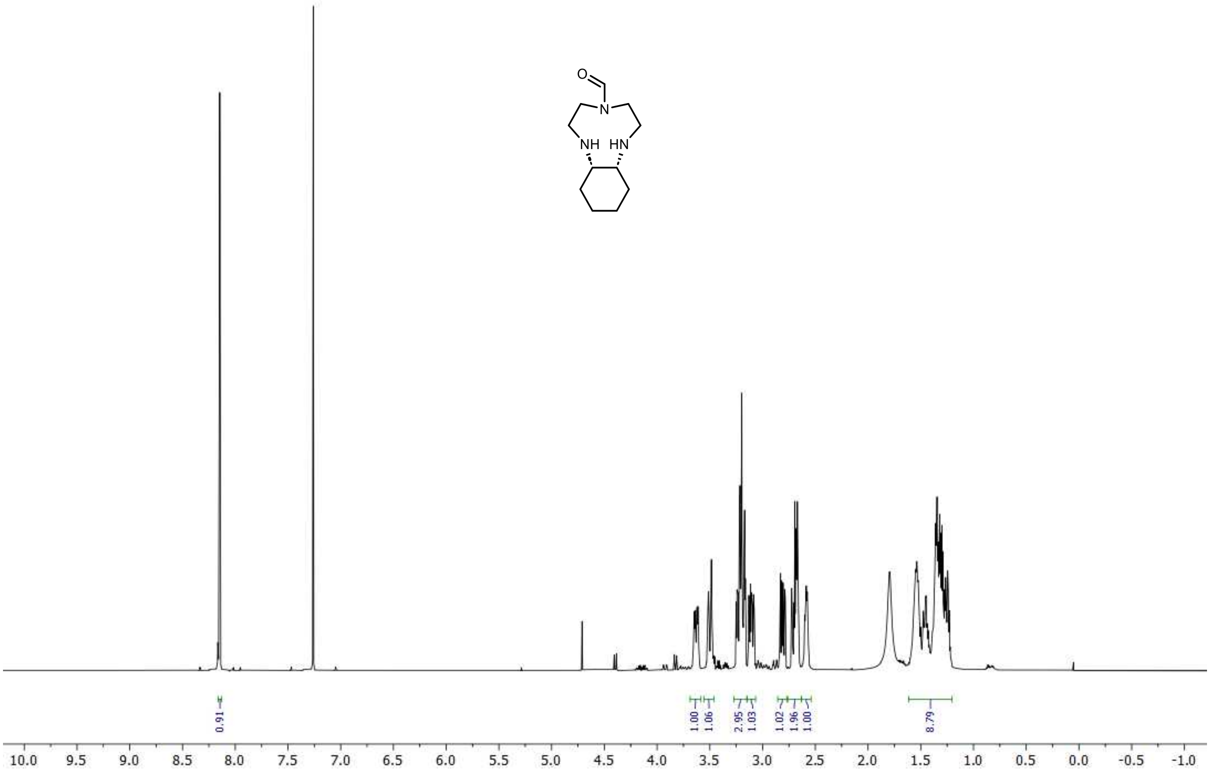

Figure S113 – ^{1}H NMR spectrum of **8-2** ($CDCl_3$, 500 MHz).

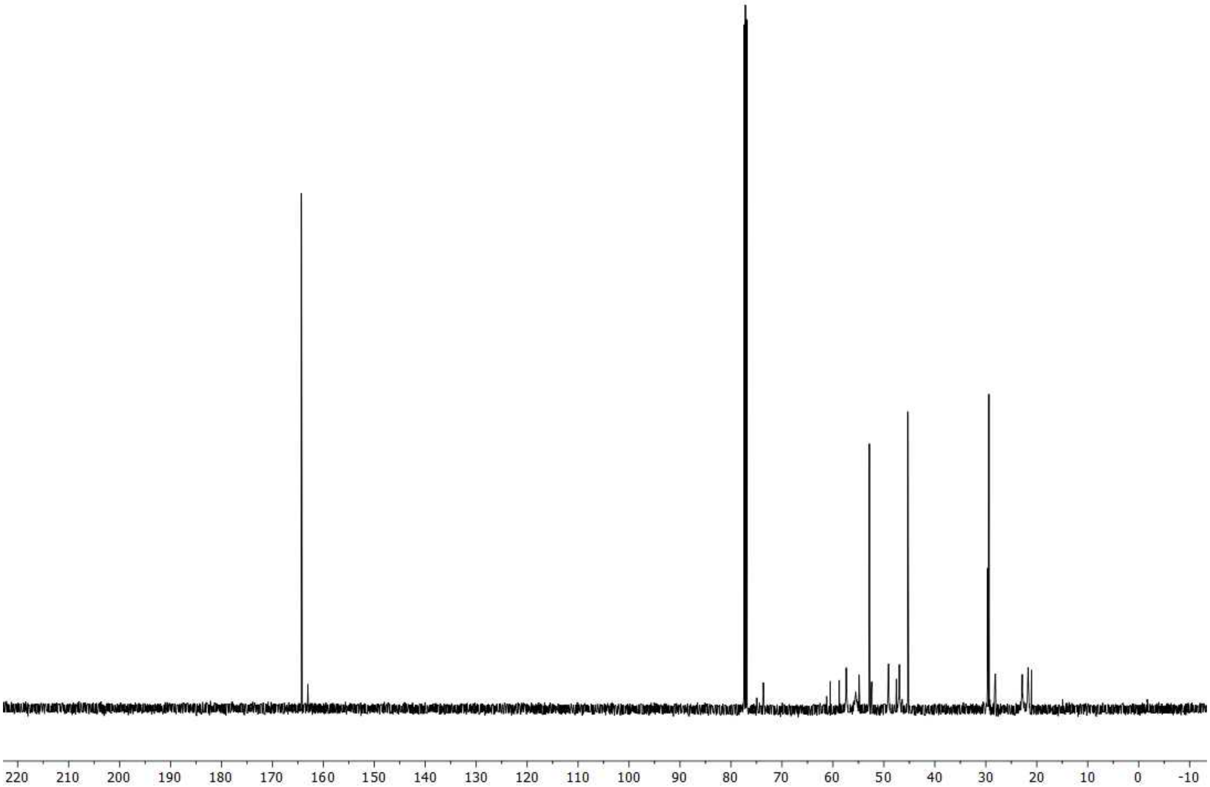

Figure S114 – ^{13}C NMR spectrum of **8-2** ($CDCl_3$, 126 MHz).

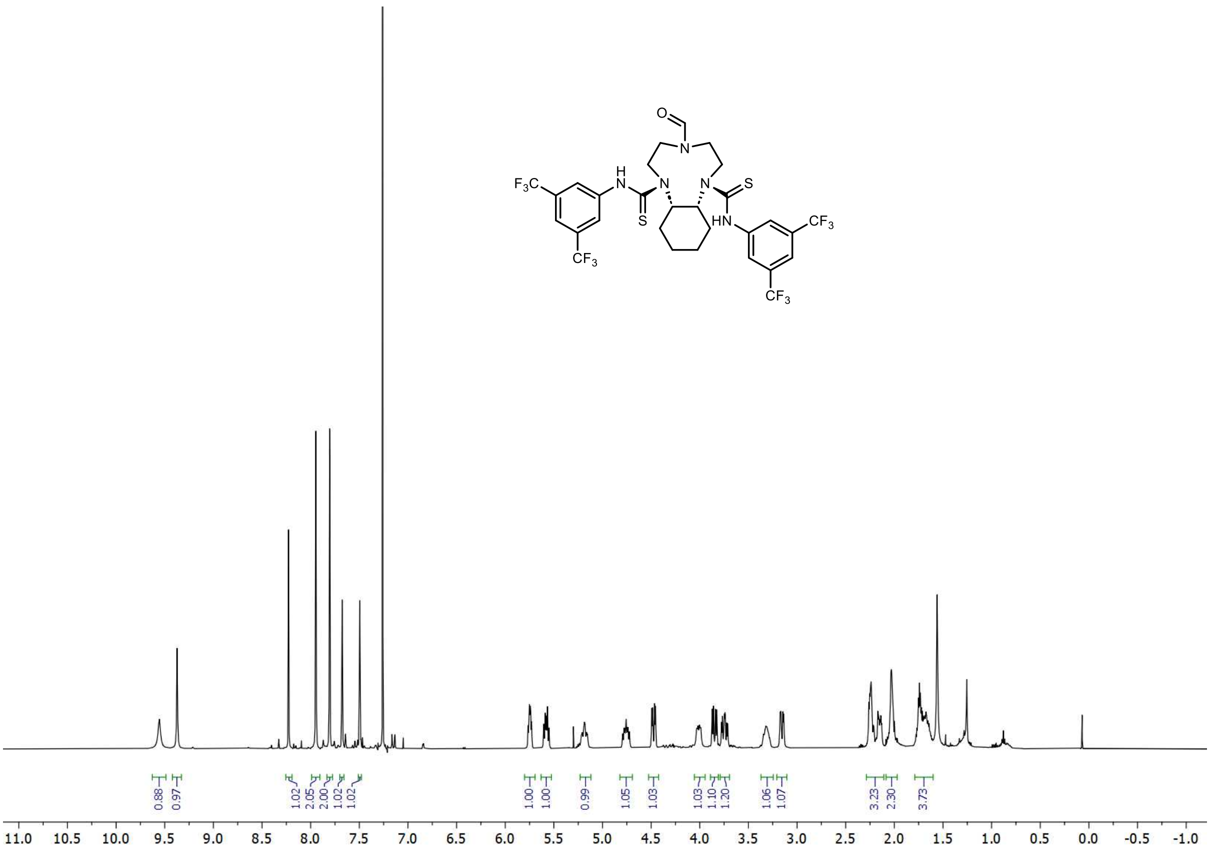

Figure S115 – ^{1}H NMR spectrum of **8** ($CDCl_3$, 500 MHz).

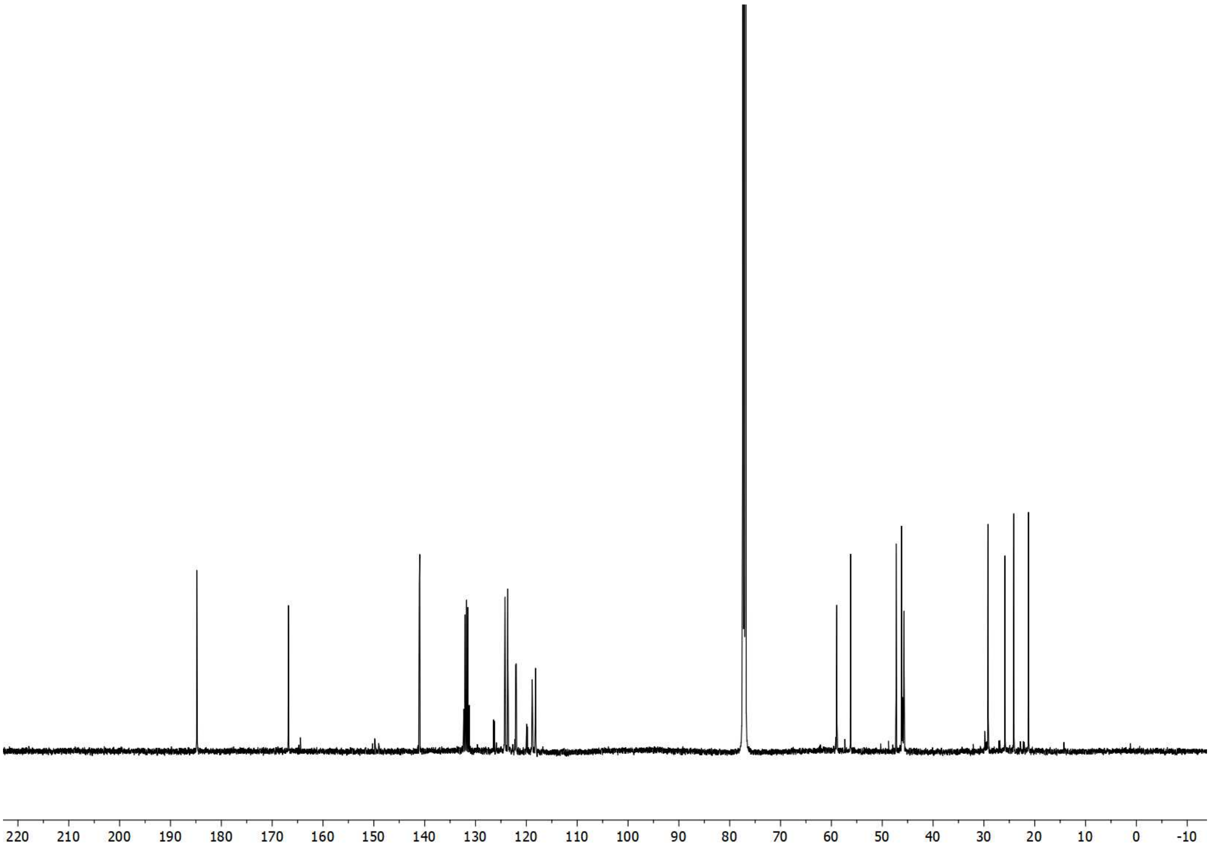

Figure S116 – ^{13}C NMR spectrum of **8** ($CDCl_3$, 126 MHz).

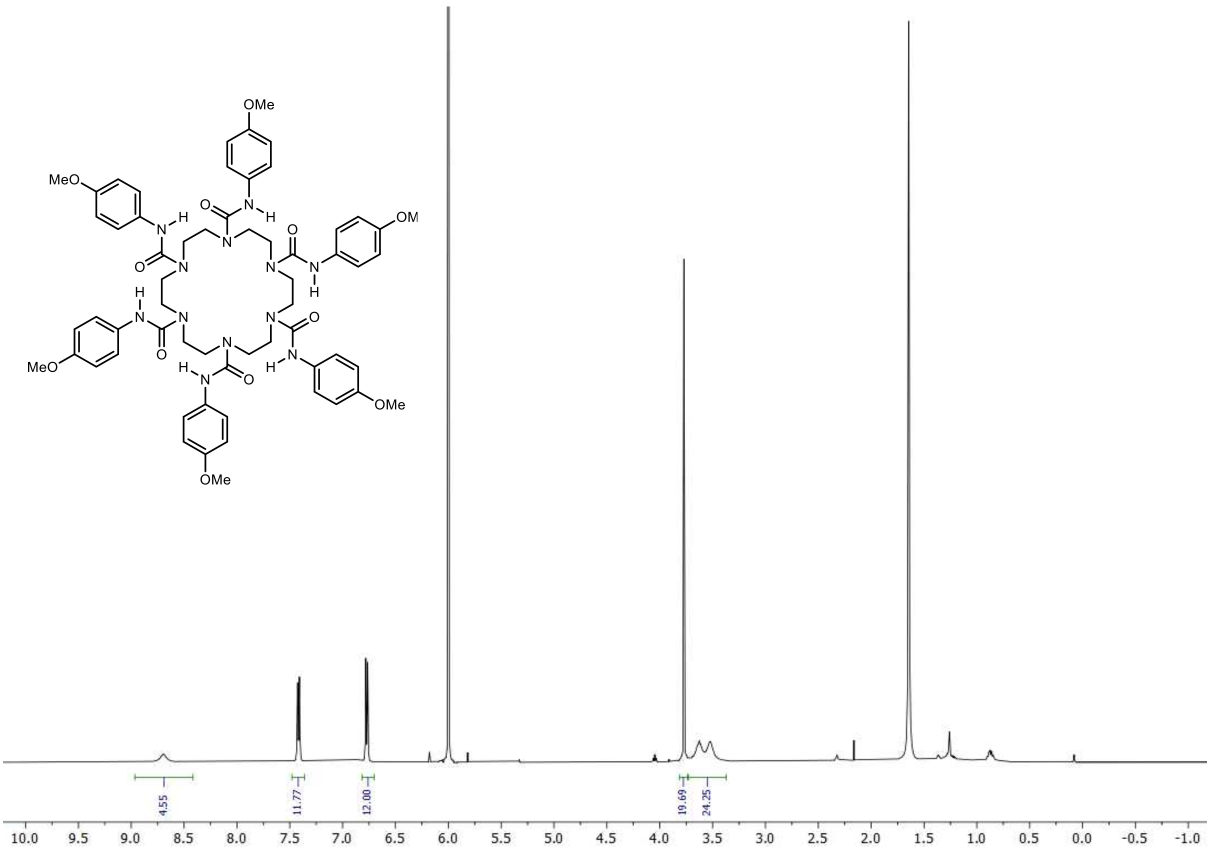

Figure S117 – ^{1}H NMR spectrum of **9** ($C_2D_2Cl_4$, 500 MHz).

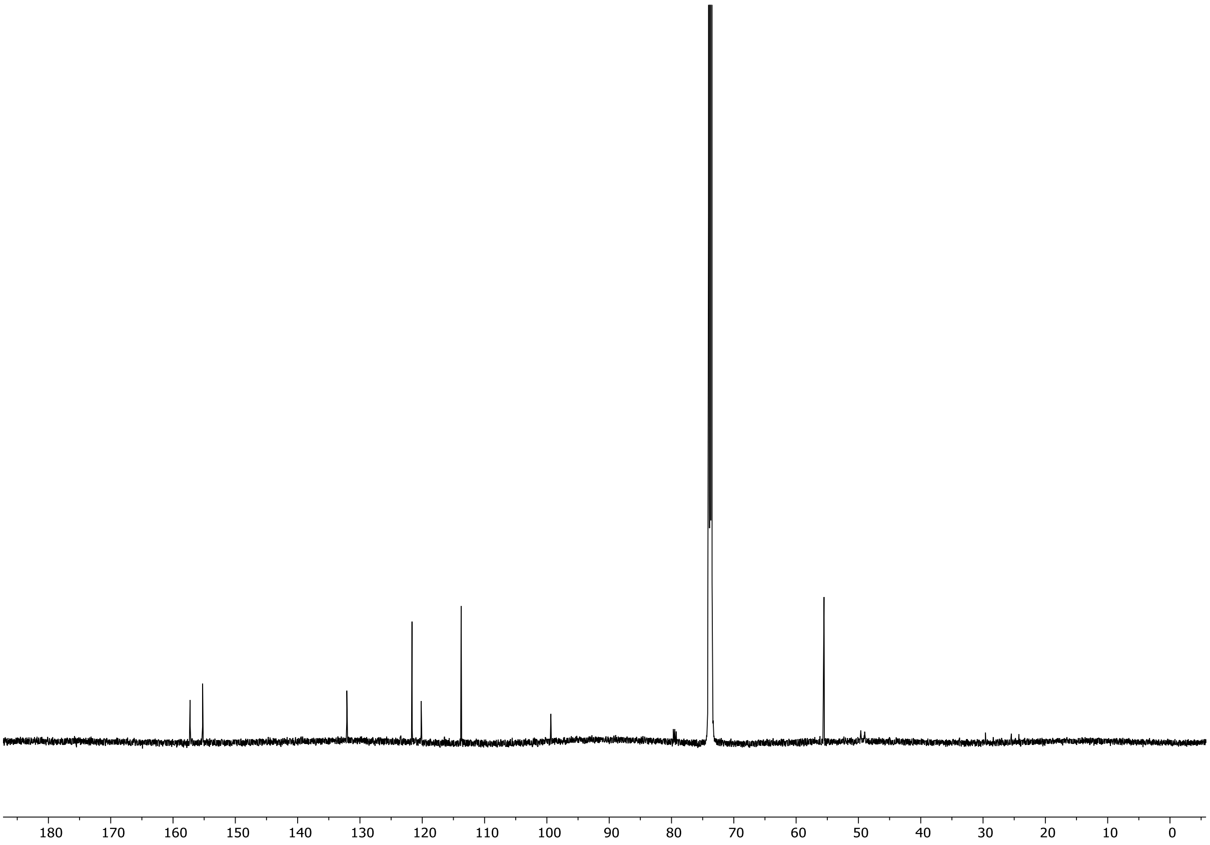

Figure S118 – ^{13}C NMR spectrum of **9** ($C_2D_2Cl_4$, 126 MHz)

References

(S1) Fan, Y.; Kass, S. R. *Org. Lett.* **2016**, *18*, 188–191.

(S2) Hron, R.; Jursic, B. S. *Tetrahedron Lett.* **2014**, *55*, 1540–1543.

(S3) Pulacchini, S.; Sibbons, K. F.; Shastri, K.; Motevalli, M.; Watkinson, M.; Wan, H.; Whiting, A.; Lightfoot, A. P. *Dalt. Trans.* **2003**, 2043–2052.

(S4) Golding, S. W.; Hambley, T. W.; Lawrance, G. A.; Luther, S. M.; Maeder, M.; Turner, P. *J. Chem. Soc. - Dalt. Trans.* **1999**, 1975–1980.

(S5) Roger, M.; Patinec, V.; Bourgeois, M.; Tripier, R.; Triki, S.; Handel, H. *Tetrahedron* **2012**, *68*, 5637–5643.

(S6) Gai, Y.; Sun, L.; Lan, X.; Zeng, D.; Xiang, G.; Ma, X. *Bioconjug. Chem.* **2018**, *29*, 3483–3494.

(S7) Nikitin, K.; O'Gara, R. *Chem. – A Eur. J.* **2019**, *25*, 4551–4589.

(S8) Paquette, L. A.; Wang, T. Z.; Luo, J.; Cottrell, C. E.; Clough, A. E.; Anderson, L. B. *J. Am. Chem. Soc.* **1990**, *112*, 239–253.

(S9) Du, J.-Y.; Balan, T.; Claridge, T. D. W.; Smith, M. D. *J. Am. Chem. Soc.* **2022**, *144*, 14790–14797.

(S10) Steffel, L. R.; Cashman, T. J.; Reutershan, M. H.; Linton, B. R. *J. Am. Chem. Soc.* **2007**, *129*, 12956–12957.

(S11) Gaussian. *Gaussian 16, Revis. C.01, M. J. Frisch, G. W. Truck. H. B. Schlegel, G. E. Scuseria, M. A. Robb, J. R. Cheeseman, G. Scalmani, V. Barone, G. A. Petersson, H. Nakatsuji, X. Li, M. Caricato, A. V. Marenich, J. Bloino, B. G. Janesko, R. Gomperts, B. Mennu.*

(S12) Becke, A. D. *J. Chem. Phys.* **1993**, *98*, 5648–5652.

(S13) Lee, C.; Yang, W.; Parr, R. G. *Phys. Rev. B* **1988**, *37*, 785–789.

(S14) Grimme, S. *Wiley Interdiscip. Rev. Comput. Mol. Sci.* **2011**, *1*, 211–228.

(S15) Weigend, F.; Ahlrichs, R. *Phys. Chem. Chem. Phys.* **2005**, *7*, 3297–3305.

(S16) Grimme, S. *Chem. - A Eur. J.* **2012**, *18*, 9955–9964.

(S17) Luchini, G.; Alegre-Requena, J. V.; Funes-Ardoiz, I.; Paton, R. S. *F1000Research* **2020**, *9*, 291.

(S18) Grimme, S. *J. Chem. Theory Comput.* **2019**, *15*, 2847–2862.

(S19) Marenich, A. V.; Cramer, C. J.; Truhlar, D. G. *J. Phys. Chem. B* **2009**, *113*, 6378–6396.

(S20) Adamo, C.; Barone, V. *J. Chem. Phys.* **1999**, *110*, 6158–6170.

(S21) Yanai, T.; Tew, D. P.; Handy, N. C. *Chem. Phys. Lett.* **2004**, *393*, 51–57.

(S22) Neese, F.; Wennmohs, F.; Becker, U.; Riplinger, C. *J. Chem. Phys.* **2020**, *152*, 224108.

(S23) *Bruker, SAINT+ v8.39.0 Integration Engine, Data Reduction Software, Bruker Analytical X-ray Instruments Inc., Madison, WI, USA,* **2018**.

(S24) *Bruker, SADABS 2018, Bruker AXS area detector scaling and absorption correction, Bruker Analytical X-ray Instruments Inc., Madison, Wisconsin, USA,* **2018**.

(S25) Palatinus, L.; Chapuis, *G. J. Appl. Crystallogr.* **2007**, *40,* 786−790.

(S26) Palatinus, L.; Prathapa, S. J.; van Smaalen, S. *J. Appl. Crystallogr.* **2012**, *45*, 575−580.

(S27) Sheldrick, G. M. *Acta Crystallographica a-Foundation and Advances* **2015**, *71*, 3−8.

(S28) Sheldrick, G. M. *Acta Crystallogr., Sect. A: Found. Crystallogr.* **2008**, *64*, 112−122.

(S29) Sheldrick, G. M. *Acta Crystallogr. C* **2015**, *71*, 3−8.

(S30) Dolomanov, O. V.; Bourhis, L. J.; Gildea, R. J.; Howard, J. A. K.; Puschmann, H. *J. Appl. Crystallogr.* **2009**, *42*, 339−341.
